# Evidence of synergistic mechanisms of hepatoprotective botanical herbal preparation of *Pueraria montana var. lobata* and *Schisandra sphenanthera*

**DOI:** 10.3389/fphar.2024.1412816

**Published:** 2024-06-24

**Authors:** Yang Lv, Huan Li, Bing-Tao Zhai, Jing Sun, Jiang-Xue Cheng, Xiao-Fei Zhang, Dong-Yan Guo

**Affiliations:** State Key Laboratory of Research and Development of Characteristic Qin Medicine Resources (Cultivation), and Shaanxi Province Key Laboratory of New Drugs and Chinese Medicine Foundation Research, College of Pharmacy, Shaanxi University of Chinese Medicine, Xi’an, China

**Keywords:** *Pueraria montana* var.var. lobata (Willd.) Maesen & S.M.Almeida ex Sanjappa & Predeep, *Schisandra sphenanthera* Rehder & E.H. Wilson, acute liver injury, symptomoriented network pharmacology, multi-omics

## Abstract

**Background:**

*Pueraria montana var. lobata* (Willd.) Maesen & S.M.Almeida ex Sanjappa & Predeep (syn. *Pueraria lobata* (Willd.) Ohwi) and *Schisandra sphenanthera* Rehder & E.H. Wilson are traditional edible and medicinal hepatoprotective botanical drugs. Studies have shown that the combination of two botanical drugs enhanced the effects of treating acute liver injury (ALI), but the synergistic effect and its action mechanisms remain unclear. This study aimed to investigate the synergistic effect and its mechanism of the combination of *Pueraria montana var. lobata* (Willd.) Maesen & S.M.Almeida ex Sanjappa & Predeep (syn. *Pueraria lobata* (Willd.) Ohwi) (PM) and *Schisandra sphenanthera* Rehder & E.H. Wilson (SS) in the treatment of ALI.

**Methods:**

High performance liquid chromatography (HPLC) were utilized to conduct the chemical interaction analysis. Then the synergistic effects of botanical hybrid preparation of PM-SS (BHP PM-SS) against ALI were comprehensively evaluated by the CCl_4_ induced ALI mice model. Afterwards, symptom-oriented network pharmacology, transcriptomics and metabolomics were applied to reveal the underlying mechanism of action. Finally, the key target genes were experimentally by RT-qPCR.

**Results:**

Chemical analysis and pharmacodynamic experiments revealed that BHP PM-SS was superior to the single botanical drug, especially at 2:3 ratio, with a better dissolution rate of active ingredients and synergistic anti-ALI effect. Integrated symptom-oriented network pharmacology combined with transcriptomics and metabolomics analyses showed that the active ingredients of BHP PM-SS could regulate Glutathione metabolism, Pyrimidine metabolism, Arginine biosynthesis and Amino acid sugar and nucleotide sugar metabolism, by acting on the targets of AKT1, TNF, EGFR, JUN, HSP90AA1 and STAT3, which could be responsible for the PI3K-AKT signaling pathway, MAPK signaling pathway and Pathway in cancer to against ALI.

**Conclusion:**

Our study has provided compelling evidence for the synergistic effect and its mechanism of the combination of BHP PM-SS, and has contributed to the development and utilization of BHP PM-SS dietary supplements.

## 1 Introduction

In recent years, due to environmental pollution, drug abuse, changes in dietary habits and autoimmunity, acute liver injury (ALI) has been increasing in frequency. Currently, about 2 million people worldwide die from liver injury each year, accounting for 3.5 per cent of all global deaths, so much so that it has become one of the global public problems contributing to morbidity and mortality (Yu et al., 2021). Studies have shown that the main pathogenesis of ALI includes inflammation, redox imbalance, Kupffer cell activation, and ROS damage associated with CYP2E1 metabolism, mainly caused by alcohol, chemical toxins, drugs, and metabolic disorders (Yu et al., 2014; Zhang M Q et al., 2020; Li R et al., 2021). Although currently approved drugs such as acetaminophen (APAP) is therapeutically effective of hepatitis, but they target only single target and some even have serious side effects such as neurological abnormalities, gastrointestinal reactions and endocrine system disorders (Lefkowitch, 2016). With the global interest in Chinese medicine, traditional medicinal and food plant sources of hepatoprotective agents have attracted extensive attention owing to their low toxicity and therapeutic efficacy.

*Pueraria montana var. lobata* (Willd.) Maesen & S.M.Almeida ex Sanjappa & Predeep, the synonym of *Pueraria lobata* (Willd.) Ohwi, also known as kudzu, is a leguminous vine, one of the earliest plants used in nutritious foods and botanical drugs. It is widely distributed in Asia, Europe, and the Americas, and the formulations containing PM are sold around the world as nutraceuticals (He et al., 2022). According to the Chinese Pharmacopoeia 2020 edition, PM is cool and sweet in taste, and has the efficacy of antipyretic muscle, promoting fluid production to quench thirst, promoting meridian and blood and detoxifying alcohol. In particular, it has thousand years of practical experience on alcoholic intoxication, nausea and vomiting and painful and full abdominal mass and plumpness injury caused by drinking alcohol. Chemical composition studies have shown that flavonoids, triterpenoids, organic acids and volatile oils are the main bioactive components of PM, which have hepatoprotective (He et al., 2022), hypoglycemic (Hou et al., 2020), anti-inflammatory (Tan et al., 2022), antioxidant (Zhou et al., 2022) and anti-tumor effects (Chen et al., 2016). Previous studies have shown that puerarin, as one of the main components of PM, has been shown to contribute to the suppression of inflammatory responses, regulation of lipid metabolism and inhibition of oxidative stress to alleviate liver injury (Liu et al., 2021). Schisandra fruits are the dried ripe fruits of *Schisandra sphenanthera* Rehder & E.H. Wilson, which were used as traditional botanical drugs and dietary supplements over thousand years, and have been recorded in the China, Japan, South Korea, the United States and Russia Pharmacopoeias (Yang et al., 2022). According to TCM theory, SS is warm and acid, and can be used for the treatment of liver and kidney deficiency of Yin or Yang Syndrome. Modern medical research shows that SS has therapeutic effects such as hepatoprotection, neuroprotection, cardiovascular protection, regulation of blood glucose and blood lipids, and anti-cancer (Qiao et al., 2020; Huang et al., 2021; Li et al., 2023). Compositional studies have found that SS contains active ingredients such as lignans, polysaccharides, essential oils and organic acids (Lu and Chen, 2009). Recent studies have found that Schisandrin A and Schisandrin B alleviate hepatic inflammation and oxidative damage *in vivo* by increasing hepatic β-oxidation and fatty acid oxidation, inhibiting lipid peroxidation, and regulating the NF-κB/p38/ERK MAPK and Nrf2/HO-1 signaling pathways (Kwon et al., 2018; Wang HQ et al., 2022; Yan et al., 2022). According to the “Synopsis of Prescriptions of the Golden Chamber”, for liver disease, acid is used for tonic and sweet is used for enhance tonic. PM-SS is the core botanical hybrid preparation of the approach, and the combination of two botanical drugs could enhance each other to strengthen the hepatoprotective effect and provide better efficacy in ALI. A national patent-based report on the frequency pattern of herbal compounding for the treatment of chemical liver injury noted that PM and SS occur together most frequently (FAN et al., 2020). In addition, many dietary supplements based on the BHP PM-SS have been developed and successfully registered as functional foods in China (approval numbers G20070297, G20130739 and G20190463), all claiming to have “complementary functions for the prevention of chemical liver injury”. The above evidence suggests that BHP PM-SS has beneficial effects on ALI. However, due to the complex modification of chemical composition during the compounding process, which usually has a synergistic effect on the disease. Thus, it is necessary to study the changes and potentiation of the main active ingredients in BHP PM-SS and the mechanism of action to be further elucidated.

Symptoms are the key to the theoretical practice of TCM, manifesting as subjective abnormal sensations and pathological changes in the body of the patient in illness states. Clinical practice suggests that symptom-oriented clinical decision-making is both clinically relevant and reduces overdose, and is a concrete practice of the patient’s “real world”. Network pharmacology approach transforms the traditional one-gene/one-drug/one-disease drug development strategy into a bio-network-targeted therapeutic strategy, which can establish a network correlation between macroscopic disease symptoms and microscopic biomolecules, and help to analysis the nature of symptoms in a disease state (Jiang et al., 2022). Shi et al. using the symptom-oriented pharmacological network analysis of “qi”, “blood”, “pain”, and “inflammation”, finally identified MMP2 and DRD2 and AKR1B1 as key targets of Jinhong tablets against chronic superficial gastritis (Shi et al., 2022). Clinical findings show that patients with ALI frequently exhibit jaundice, abdominal distension, fatigue, nausea and vomiting, and poor appetite. Therefore, a symptom-oriented pharmacological network of jaundice, abdominal distension, fatigue, Chemotherapy-induced nausea and vomiting (CINV) and poor appetite are harnessed for excavating the core targets. Transcriptomics is a technique that reveals the relationship between genes associated with disease development and physiological function, enabling determination of potential therapeutic targets for drugs on disease protection (Pan et al., 2022). Metabolomics technologies can observe the dynamics of endogenous metabolites within organs, systems or organisms during pathological or physiological processes through qualitative and quantitative techniques (Wang R et al., 2022). Undoubtedly, the integrated analysis of network pharmacology, transcriptomics and metabolomics can help to gain insights into the complex regulatory networks in disease states.

In this study, we performed HPLC analysis combined with pharmacodynamic experiments to screen the optimal proportion of BHP PM-SS against ALI for the first time. In addition, we emphasized on using symptom-oriented network pharmacology, transcriptomics and metabolomics approaches to reveal the underlying mechanism of action, thus exploring how BHP PM-SS contributes to ALI prevention. These findings provided compelling evidence for the development and utilization of BHP PM-SS dietary supplements.

## 2 Materials and methods

### 2.1 Reagents and chemicals

Schisantherin A (batch No. 11529-200604, purity 99.3%), Puerarin (batch No. 110752–200041, purity 99.3%) were purchased from National Institutes for food and Drug Control (Beijing, China). 3′-hydroxy Puerarin (batch No. HR219W6, purity >98%), Puerarin apigenin (batch No. HS514368W1, purity >98%), Daidzin (batch No. HR12121B2, purity >98%), Daidzein (batch No. HR14626B1, purity >98%), Schisandrol A (batch No. HS19910B1, purity >98%), Schisandrol B (batch No. HS19911B1, purity >98%), Schisandrin A (batch No. HS19906B1, purity >98%), Schisandrin B (batch No. HR2104W7, purity >98%), Schisandrin C (batch No. HS19912B1, purity >98%) were purchased from Chenguang Biotechnology Co., Ltd (Baoji, China). HPLC-grade acetonitrile was obtained from Thermofisher Scientific Co. Ltd. (Waltham, MA, United States). CCl_4_ from Tianjin Tianli Chemical Reagent Co., Ltd. (Tianjin, China). Silymarin was obtained from Sigma Chemical Company (Milan, Italy). The diagnostic kits specific for aspartate aminotransferase (AST), alanine aminotransferase (ALT), triglycerides (TG), total cholesterol (TC), superoxide dismutase (SOD), catalase (CAT), malondialdehyde (MDA) and glutathione (GSH) were all purchased from Nanjing Jiancheng Institute of Biotechnology (Nanjing, China). Enhanced Bicinchoninic Acid (BCA) Protein Assay Kit was supplied by Beyotime Institute of Biotechnology (Jiangsu, China). Mouse interleukin-1β (IL-1β), interleukin-6 (IL-6) and TNF-α enzyme-linked immunosorbent assay (ELISA) kits were purchased from MeiMian Co., Ltd. (Jiangsu, China).

### 2.2 Preparation and detection of the BHP PM–SS

The decoction pieces of roots of *P. montana var. lobata (Willd.)* (PM) and the fruits of *S. sphenanthera* (SS) were offered by the Kangchao Kangjian Pharmaceutical Co., Ltd. (Shaanxi, China), and authenticated by senior experimentalist Wang Jitao of Shaanxi University of Chinese Medicine. The amounts of crude botanical drugs of PM and SS were weighed according to a ratio of 0:1, 1:0, 1:1, 1:2, 2:1, 2:3 and 3:2, each of which was 60 g in total, and then smashed to a powder. Then, the powders were immersed in 10 times the volume of distilled water at room temperature overnight. The next day, the mixture was boiled at 100°C for 2 h to obtain the water extraction. The extraction procedure was repeated. The extracts was freeze-dried, producing a powder and stored at 4 °C.

Then, the main active ingredients were analyzed by HPLC, and the contents were calculated by a standard curve method. The HPLC analysis was carried out on an Agilent 1260 Infinity II HPLC system with a Cosmosil 5C_18_-MS-Ⅱ HPLC column (250 mm × 4.6 mm, 5 μm) at 30 °C. The mobile phase was acetonitrile (A) and 0.1% phosphoric acid water (B) at a flow rate of 1.0 mL/min. The gradient elution program was as follows: 0–3 min, 5%–11% A; 3–15 min, 11% A; 15–25 min, 11%–15% A; 25–38 min, 15%–35% A; 38–43 min, 35%–65% A; 43–50 min, 65%–75% A; 50–60 min, 75%–80% A; 60–70 min, 80%–85% A. The sample volume was 10 μL. DAD detection wavelength was 254 nm.

### 2.3 Calculation of the optimal proportion of BHP PM-SS

Overall desirability (OD) can be used in multi-indicator optimization experiments to avoid mutual contradictions between indicators. Usually, the OD value is in the range of 0–1, and the closer to 1 means that the optimisation conditions are favourable to the overall effect of multiple indicators (Chen et al., 2020). Using the phytochemical hybrid preparation (PHP) which is composed of 11 phytochemicals 3′-hydroxy Puerarin, Puerarin, Puerarin apigenin, Daidzin, Daidzein, Schisandrol A, Schisandrol B, Schisantherin A, Schisandrin A, Schisandrin B and Schisandrin C (abbreviated PHP-11) in BHP PM-SS as indicators, the data were processed by using the normalization. Among them, Dissolution rate of single ingredient (Y) = amount of the ingredient in the total extracts/sample amount of the corresponding proportion of raw material for the ingredient×100%. Then, Hassan method by mathematical transformation was used to calculate the normalized values of PHP-11 in different ratios of BHP PM-SS. The formula is shown in Equation 1:

$$d_{i}=\left(Y_{i}-Y_{\min}\right)/\left(Y_{\max}-Y_{\min}\right)$$ (1)

Where d_i_ represents the normalized value of the dissolution rate of single component, Y_i_ represents the dissolution rate of single component, Y_max_ represents the maximum dissolution rate of single component in different ratios, Y_min_ represents the minimum dissolution rate of single component in different ratios.

Then, the data processing based on Eq. 1 was carried out for the normalized values of the PHP-11 in different ratios of BHP PM-SS to obtain the overall desirability (OD). The formula is shown in Equation 2:

OD=(d1×d2×d3×•••×dn)^1/n (2)

Where OD represents the normalized value of the overall desirability of PHP-11 in different ratios, d_n_ represents the normalized value of the dissolutions rate of single component in different ratios, and n represents the number of components.

### 2.4 Symptom-oriented network pharmacology

Firstly, the 2D structure files of the 11 components were downloaded from the PubChem database (https://pubchem.ncbi.nlm.nih.gov/) and exported in SMILES format. All SMILES were uploaded to the SwissTargetPrediction server (http://www.swisstarget prediction.ch/) for target prediction (Relevance score>0). As a complement, PharmMapper (http://www.lilab-ecust.cn/pharmmapper/) was used to identify component targets (Relevance score>0.5), and after removal duplicates, a database 1 of “*Homo sapiens* species” was obtained. We selected the ALI clinical symptoms “jaundice”, “abdominal distension”, “abdominal distension”, “fatigue”, “CINV”, “poor appetite” and “acute liver injury” as keywords in the GeneCards database (https://genealacart.genecards.org/) to track symptom and disease targets. The median of the key parameters of network pharmacology was used to set the necessary screening criteria. For example, “acute liver injury” screened all targets with a median score >16.12. The ALI targets were intersected with the five symptom targets to obtain database 2, and then database 1 was intersected with database 2 to obtain an overlapping database containing potential targets for BHP PM-SS anti-ALI.

The String database (https://string-db.org/) was used to analyze known and predicted protein-protein interactions. The overlapping database and the five symptom-based target datasets were imported separately into the String database for PPI analysis. Species classification was restricted to “*Homo sapiens*” and a “medium confidence score >0.4″was considered significant. The results were visualized using Cytoscape 3.7.1 software. Based on the “degree value”, the top 3 major symptom targets were selected as the core targets for BHP PM-SS anti-ALI, and the compounds corresponding to the core targets were obtained. A component-target-symptom network was finally constructed in Cytoscape 3.7.1 software. GO and KEGG analyses were performed using the David database (https://david.ncifcrf.gov/) to analysis the top 5 in terms of molecular function, cellular composition, and biological processes of symptom-related targets, and to obtain the top 10 signaling pathways for mapping. Finally, GO and KEGG enrichment bubble maps were created using a bioinformatics platform (http://www.bioinformatics.com.cn/).

Based on the targets on the key targets, we downloaded the crystal structures of AKT1 (PDB ID: 3cqu), TNF (PDB ID: 7jra), JUN (PDB ID: 5t01), HSP90AA1 (PDB ID: 4bqg), EGFR (PDB ID: 6jxt) and STAT3 (PDB ID: 6njs) in the PDB database (www.rcsb.org), with resolution less than 3 Å and *homo sapiens*. Molecular docking was performed on AutoDock Vina 1.5.6 and Pymol 2.4 for ligand preparation, receptor preparation, docking method validation, docking calculations and result visualization.

### 2.5 Animals and experimental procedures

Eighty male c57BL/6 mice (20–22 g) were purchased from Chengdu Dashuo Experimental Animal Co., Ltd. (Chengdu, China, licensed ID: SCXK (Sichuan) 2020-030). All animals were acclimatised for 7 days before the experiment (temperature: 21°C ± 2°C, relative humidity: 45% ± 10%, 12 h light-dark cycle). All animal experiments were performed in accordance with the National Institutes of Health Guide for the Care and Use of Laboratory Animals and were approved by the Institutional Animal Ethics Committee of Shaanxi University of Chinese Medicine (approval number SUCMDL20211214001). The mice were randomly divided into the control group, model group, silymarin group, BHP PM–SS (0 : 1), BHP PM–SS (1 : 0), BHP PM–SS (1 : 1), BHP PM–SS (1 : 2), BHP PM–SS (2 : 1), BHP PM–SS (2 : 3) and BHP PM–SS (3 : 2) groups, each group contained 8 animals. Based on pre-experiments and our previous work (Guo et al., 2023), mice in the control group and model group were given normal saline by oral administration, the oral dose of 10 g raw material/kg was used for the BHP PM-SS groups, which was is equivalent to a daily human dose of 1.2 g/kg, and the oral dose of silymarin was 200 mg/kg. On the 10th day, except for the control group, all groups were injected intraperitoneally with CCl_4_ (2% CCl_4,_ dissolved in olive oil) at a dose of 0.1 mL/10 g. After intraperitoneal injection of CCl_4_ solution, all experimental mice were fasted for 24 h. After CCl_4_ treatment, the mice were sacrificed under ether anesthesia. Blood serum was collected by centrifugation at 12,000 x g for 10 min at 4°C and stored at −80°C. Intact livers were washed and weighed in saline and stored in 4% paraformaldehyde or liquid nitrogen.

### 2.6 Histopathological analysis

Paraffin embedded sections of the livers were cut with 4 μm thickness and examined after staining with H&E using a light microscope (BX43, Olympus, Tokyo, Japan) and then photographed at 200×magnification. Liver pathological scoring was performed by pathologists according to the injury grading scale (0-4) and histopathological standard scale.

### 2.7 Determination of AST, ALT, TG, and TC serum levels in mice

The activities of ALT, AST, TC and TG in serum were measured by detection kits according to the manufacturer’s instructions.

### 2.8 Assay for antioxidant markers in liver tissue

The levels of SOD, CAT, GSH and MDA in liver tissues were measured based on the instructions of the kits.

### 2.9 Cytokine activities by ELISA

Serum IL-1β, IL-6 and TNF-α were assayed according to the manufacturer’s instructions.

### 2.10 Transcriptomic analysis

In this study, eight mice livers of control, model and BHP PM-SS (2:3) groups were randomly mixed and four replicate experiments per group were designed. Total RNA extraction, cDNA library construction and sequencing of each sample were performed as described by Li C et al. (2021). Differently, a total of 12 cDNA libraries were constructed for liver tissue using the Illumina Novaseq6000 from Gene Denovo Biotechnology Co. After obtaining high-quality clean data, differentially expressed genes (Differential genes (DEGs) were identified by DESeq2 (threshold: FC ≥ 2 and *p* < 0.05). GO and KEGG analysis were used for functional analysis of DEGs, *p* < 0.05 was considered statistically significant.

### 2.11 Non-targeted metabolome analysis

Based on the biochemical results of this study, the BHP PM-SS (2:3) group was selected for metabolomic analysis. Liver samples (80 mg) were extracted with 800 μL of extraction solvent (methanol-acetonitrile, 1:1). The supernatant was extracted, vacuum dried and redissolved in 100 μL of solvent (acetonitrile-water, 1:1) and analyzed for LC-MS/MS. The LC-MS/MS analysis platform included an UPLC (1290 Infinity LC, Agilent Technologies), quadrupole time-sensitive LC-MS/MS system (AB Sciex TripleTOF 6600), and an ACQUIY UPLC BEH column (1.7 μm 2.1 × 100 mm, Waters). The mass spectrometry parameters were shown in Supplementary Tables S1–S3. OSI-SMMS (Dalian, China) was used for peak annotation after data processing. SIMCA-P 14.1 software (Umetrics, Umea, Sweden) was used to pattern recognition of mass spectrometry data. In this study, metabolites with variable importance for projection (VIP) > 1.00 and *t*-test *p*-value<0.05 were selected as selection criteria for potential biomarkers of liver injury.

### 2.12 Real-time quantitative PCR (RT-qPCR) analysis

Total RNA was extracted from 15 mg of liver tissue using Trizol reagent per manufacturer’s instructions (Takara, Japan) and the ratio of RNA A260/280 extracted from liver tissue was 1.9–2.0. Reverse transcription of 1 μg total RNA into complementary DNA (cDNA) was performed by PrimeScriptTM RT reagent Kit (Takara, Japan). The levels of mRNA expression were measured by real-time PCR with TB Green^®^ Premix Ex Taq™ II (Tli RNaseH Plus) and CFX connect Real Time PCR System (Bio-rad, United States). GAPDH was amplified as reference genes. The primer sequences used for PCR are shown in Supplementary Table S4. The 2^−ΔΔCT^ method was used to analyze the real-time PCR data.

### 2.13 Statistical analysis

Except for the analysis software used for metabolomics and transcriptomics functional annotation, data was statistically analysed and plotted using GraphPad Prism (9.4.1) and expressed as mean ± standard deviation (SD). T-tests and one-way analysis of variance (ANOVA) were employed for comparisons between two or more groups accordingly. Statistical significance was defined as *p*-value<0.05.

## 3 Results

### 3.1 The analysis of the main chemical contents of BHP PM–SS

**FIGURE 1:**
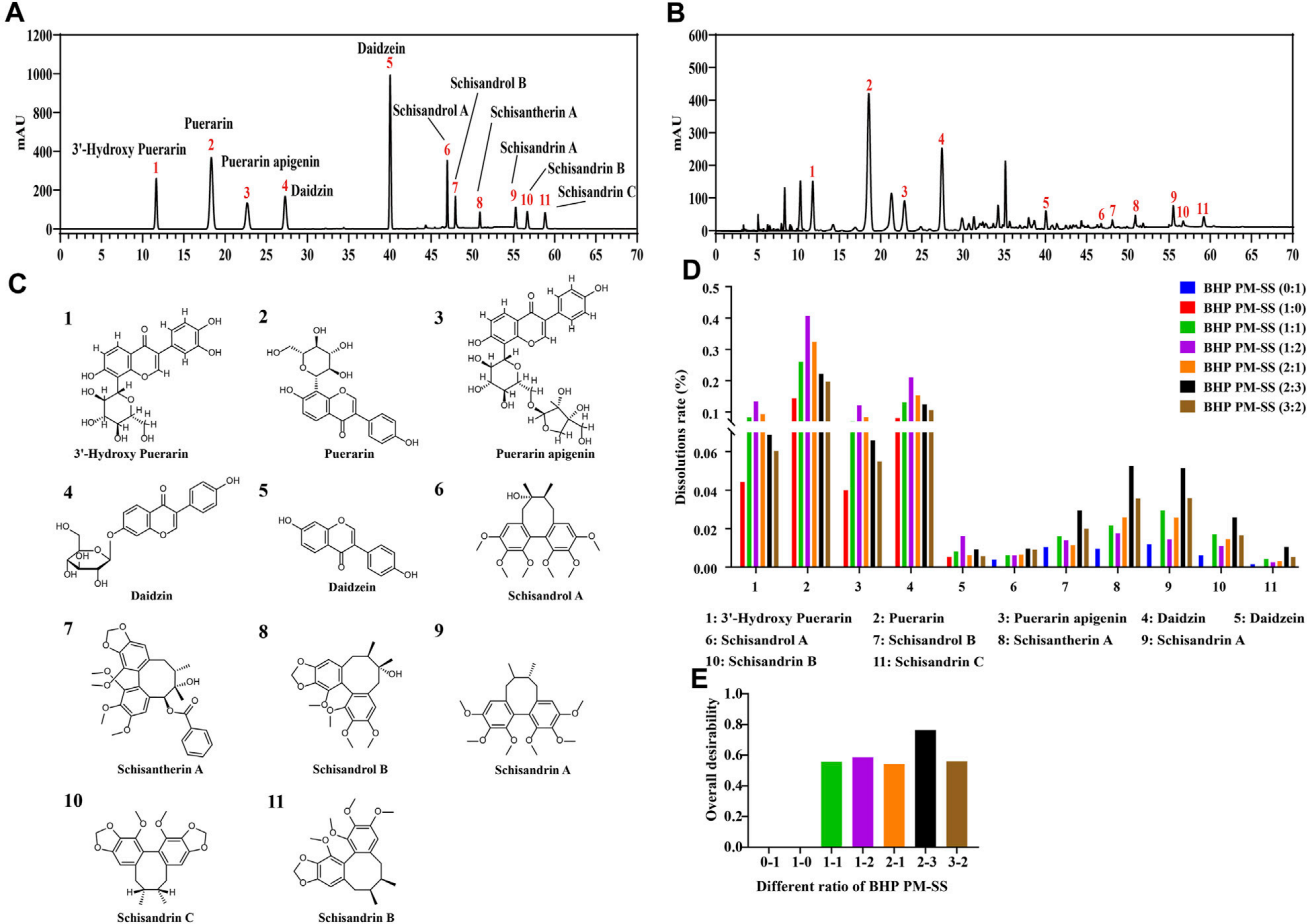
Determination of the content of PHP-11 in BHP PM-SS by HPLC. **(A)** reference substance; **(B)** BHP PM-SS sample; **(C)** Structures of identification compounds from BHP PM-SS; **(D)** The dissolution rates of PHP-11; **(E)** The overall desirability value of different ratios of BHP PM-SS.

In this study, the contents of the PHP-11 in PM, SS and BHP PM-SS (1:1, 1:2, 2:1, 2:3, 3:2) extracts were analyzed at the same wavelengths under the same preparative conditions by HPLC. The linear relationships of the PHP-11 were analysed as shown in Supplementary Table S5. The results of the content of the PHP-11 are shown in Supplementary Table S6. The standard spectra and the representative spectra of BHP PM-SS (2:3) were shown in Figures 1A,B. The 11 chemical components include 3′-hydroxy Puerarin, Puerarin, Puerarin apigenin, Daidzin, Daidzein, Schisandrol A, Schisandrol B, Schisantherin A, Schisandrin A, Schisandrin B and Schisandrin C, as shown in Figure 1C. Figure 1D visually showed that with increased SS proportion, 3′-hydroxy Puerarin, Puerarin, Puerarin apigenin, Daidzin and Daidzein in BHP PM-SS (1:1, 1:2) showed an increased dissolution rates compared with PM (1:0). In addition, when the BHP PM-SS ratio was increased from 2:1 to 2:3, the dissolution rate of the ingredients in PM decreased, in contrast to that in SS, which might be related to the fact that the SS decoction contained polysaccharides and other components that made the decoction viscous during decoction, leading to a decreased dissolution ratio of the ingredients in PM. The overall desirability of the dissolution ratios of the PHP-11 under different ratios was calculated. The results showed that the dissolution rate of the PHP-11 was greatest and most favourable for the dissolution at BHP PM-SS (2:3) (Figure 1E).

### 3.2 Effect of BHP PM-SS on serum and liver biochemical indices in ALI mice

**FIGURE 2:**
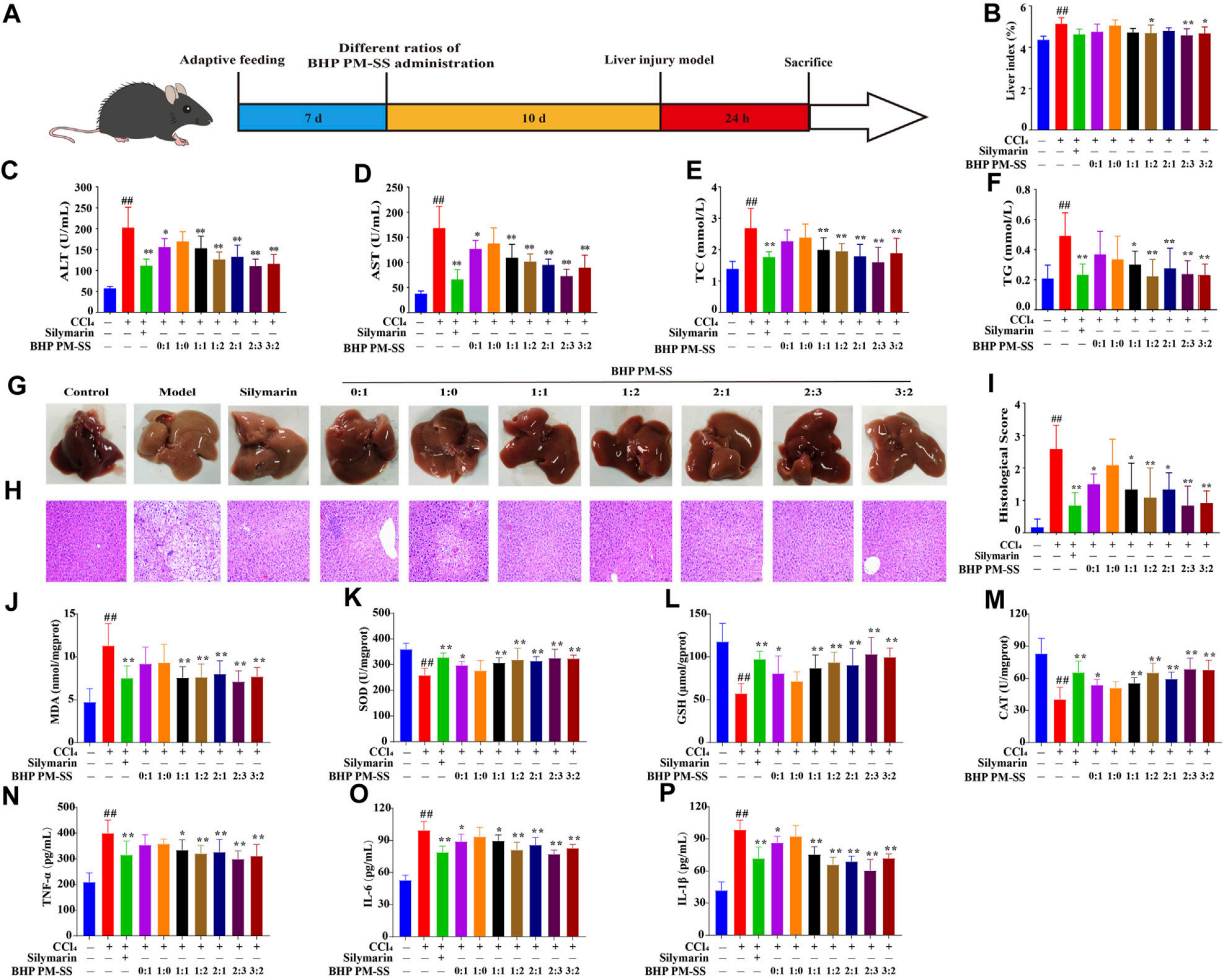
The effect of treatment with BHP PM-SS on CCl_4_-induced ALI in mice. **(A)** Experimental diagram; **(B)** The liver index of mice in each group; **(C–F)** ALT, AST, TC and TG levels in serum; **(G)** Hepatic morphology; **(H)** Histological evaluation (×200, final magnification); **(I)** Histological scores; **(J–M)** MDA, SOD, GSH and CAT in liver tissues; **(N–P)** TNF-α, IL-1β and IL-6 levels in serum. All data are presented as the mean ± SD. ##*p* < 0.01, compared to the control group; *p < 0.05 or **p < 0.01 compared to the Model group (n = 8).

As shown in Figure 2A, ALI mice were induced by CCl_4_ after BHP PM-SS preadministration intervention for 10 days. BHP PM-SS intervention reduced the CCl_4_-induced increase in liver index. In particular, BHP PM-SS (1:2, 2:3 and 3:2) performed better (Figure 2B). In addition, As shown in Figures 2C,D, compared to the control group, ALT and AST were significantly released from serum in the model group (*p* < 0.01). In contrast, the BHP PM-SS (0:1, 1:1, 1:2, 2:1, 2:3 and 3:2) intervention reduced serum ALT and AST significantly (*p* < 0.05). In particular, the BHP PM-SS (2:3) intervention significantly reduced serum ALT and AST by 45.49% and 56.90%, respectively (*p* < 0.01). As shown in Figures 2E,F, serum TC and TG levels were significantly higher in the CCl_4_ group compared with the control group (*p* < 0.01). Encouragingly, the BHP PM-SS (1:1, 1:2, 2:1, 2:3, and 3:2) intervention significantly reduced TC and TG levels (*p* < 0.05). CCl_4_ can cause significant features of acute liver injury with marked hepatic swelling and granular lesions of the liver nodules (Figure 2G). H&E staining for histological analysis revealed intact liver lobules and well-aligned cords in the control group, as shown in Figure 2H. The CCl_4_ group showed impaired and disorganized hepatic lobule structure, ballooning-like degeneration and swelling of hepatocytes around the central venous area, and necrosis of some hepatocytes with inflammatory infiltration and steatosis. In contrast, only partial lesions were observed after pre-administration of BHP PM-SS (0:1, 1:1, 1:2, 2:1, 2:3 and 3:2), indicating the effectiveness of the hepatoprotective effect of BHP PM-SS, especially at the 2:3 ratio (Figure 2I). The effect of BHP PM-SS on the oxidative and inflammation status of the liver was investigated. MDA was significantly increased (*p* < 0.01) and SOD, GSH and CAT activities were significantly decreased (*p* < 0.01) in the model mice compared with control mice. However, BHP PM-SS (1:1, 1:2, 2:1, 2:3, and 3:2) intervention mice had significantly lower (*p* < 0.01) MDA levels and significantly higher (*p* < 0.05) SOD, GSH, and CAT activities in the liver (Figure 2J–M). Compared with the control group, serum TNF-α, IL-1β, and IL-6 concentrations were significantly higher in the model group (*p* < 0.01, Figure 2N–P). Compared with the model group, serum TNF-α, IL-1β, and IL-6 concentrations were significantly lower (*p* < 0.05) in mice after BHP PM-SS (1:1, 1:2, 2:1, 2:3 and 3:2) interventions. These results suggest that the BHP PM-SS was superior to PM or SS alone in preventing liver injury and was more effective at 2:3 ratio. The results further validated the accuracy of the chemical analyses.

### 3.3 Symptom-oriented network pharmacology analysis

#### 3.3.1 Construction of an overlapping database of BHP PM-SS component targets and ALI symptom targets

A total of 832 targets for BHP PM-SS components were obtained from the SwissTargetPrediction webpage and PharmMapper webpage (Database 1). The five symptom targets obtained from the GeneCards database were further screened by intersection with disease to obtain a dataset of targets for ALI-related symptoms. Ultimately, 305 targets for jaundice, 319 targets for CINV, 368 targets for poor appetite, 361 targets for fatigue, and 268 targets for abdominal distension were obtained (Database 2). Based on the relevant targets of BHP PM-SS compounds, the symptom targets were further screened, and 115 potential anti-ALI targets of BHP PM-SS were obtained (overlapping database), including 26 targets for jaundice, 62 targets for CINV, 54 targets for poor appetite, 45 targets for fatigue, and 46 targets for abdominal distension (Supplementary Figure S1).

#### 3.3.2 Protein-protein interaction analysis of targets

**FIGURE 3:**
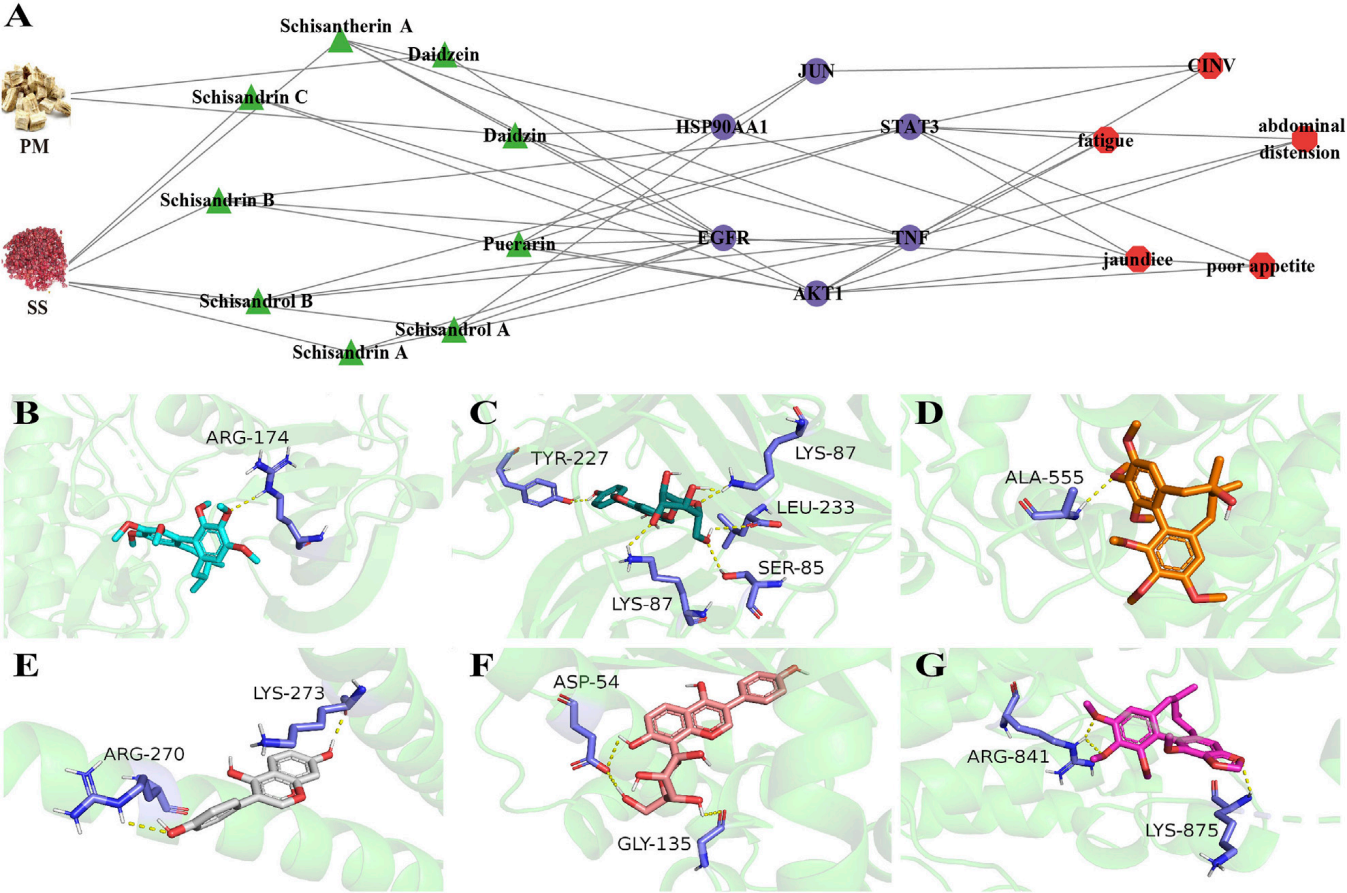
Symptom-oriented network pharmacological analyses and molecular docking. **(A)** A pharmacological network between representative chemical components, core targets and six symptoms. Green triangles for active ingredients, purple circles for core targets, red hexagons for symptoms. (For interpretation of the references to color in this figure legend, the reader is referred to the Web version of this article.); **(B)** Scisandrin A and AKT1; **(C)** Daidzin and TNF; **(D)** Schisendrol A and STAT3; **(E)** Daidzein and JUN; **(F)** Puerarin and HSP90AA1; **(G)** Schisandrin B and EGFR.

The 115 overlapping genes were input into the STRING online database, respectively, to obtain a map of the interactions between the genes (Supplementary Figure S2). Degree indicates the extent to which a gene is linked to other genes. The darker the colour, the more genes connecting to the target, indicating that the gene may be necessary for BHP PM-SS against ALI process. Degree values for the core targets of ALI disease and the five symptoms were calculated by the CytoHubba plugin in Cytoscape 3.7.1. AKT1 (Degree: 87), TNF (Degree: 87), and STAT3 (Degree: 84) are the core targets in the “acute Liver Injury”. HSP90AA1 (Degree: 39), AKT1 (Degree: 39) and STAT3 (Degree: 37) are the core targets in the “jaundice” network. AKT1 (Degree: 55), STAT3 (Degree: 53), and JUN (Degree: 51) are core targets in the “CINV” network. AKT1 (Degree: 39), STAT3 (Degree: 39) and TNF (Degree: 37) are core targets in the “fatigue” network. AKT1 (Degree: 41), STAT3 (Degree: 38) and TNF (Degree: 37) are the key targets of “abdominal distension”. AKT1 (Degree: 46), STAT3 (Degree: 46) and EGFR (Degree: 45) are core targets in the " poor appetite” network. The above six core targets (AKT1, STAT3, TNF, JUN, HSP90AA1 and EGFR) are important targets for protein-protein interactions between ALI and the five symptoms. Further pharmacological networks were constructed to more accurately assess the relationship between representative chemical components, core targets and symptoms (Figure 3A). The binding patterns of representative components and core targets were further validated by molecular docking to investigate the possibility of their interactions (Figure 3B). The lower the docking score, the better the binding of the drug ligand to the protein receptor was demonstrated. The heat map of docking scores showed strong affinity between both protein receptors and docked ligands (Supplementary Figure S3).

#### 3.3.3 GO and KEGG enrichment analysis of targets

**FIGURE 4:**
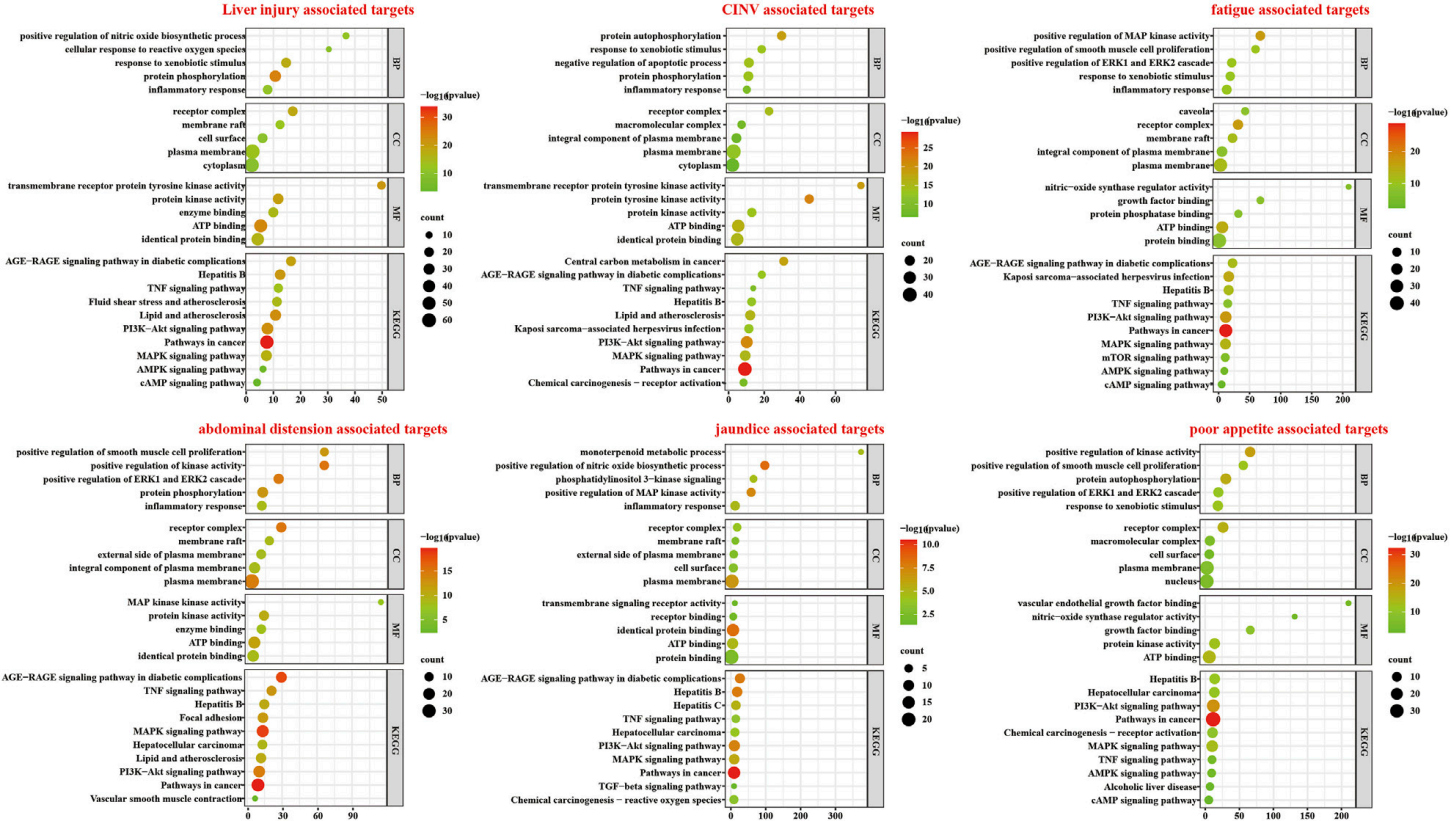
Enrichment analysis of targets related to each symptom.

The results of the GO and KEGG enrichment analyses for 115 potential targets and 5 symptom-associated targets are shown in Figure 4. Abscissa represent the count of genes. For the BP ontology, the main GO term associated with ALI and 5 symptoms was inflammatory response. For CC ontology, the targets associated with “jaundice”, “abdominal distension”, “fatigue”, “nausea and vomiting” and “poor appetite” were distributed in the two most important terms, membrane raft and recepter complax. Enrichment analysis showed significant protein kinase activity and protein binding activity. Moreover, targets related to “jaundice”, “abdominal distension”, “fatigue”, “CINV” and “poor appetite” showed ATP binding. According to KEGG analysis, Pathways in cancer, PI3K-AKT signaling pathway and MAPK signaling pathway were closely associated with the 5 symptom-related targets.

### 3.4 BHP PM-SS preconditioned and CCl_4_-induced changes in gene expression

#### 3.4.1 Transcriptomic data eligibility analysis

In total, 522,119,444 clean reads (Supplementary Table S7), with liver tissue from Control, Model and BHP PM-SS mice generating 166,384,030, 176,154,748 and 179,580,666 clean reads respectively. The percentages of clean data for Q20 and Q30 for all samples were over 97% and 92% respectively. The GC content of clean reads for each sample ranged from 48.35%–49.79%. In addition, the total number of reads mapped to the reference sequence in each liver sample was greater than 95%. These results suggest that the transcriptome data are suitable for analysis.

#### 3.4.2 Analysis of expressed genes and DEGs between samples

**FIGURE 5:**
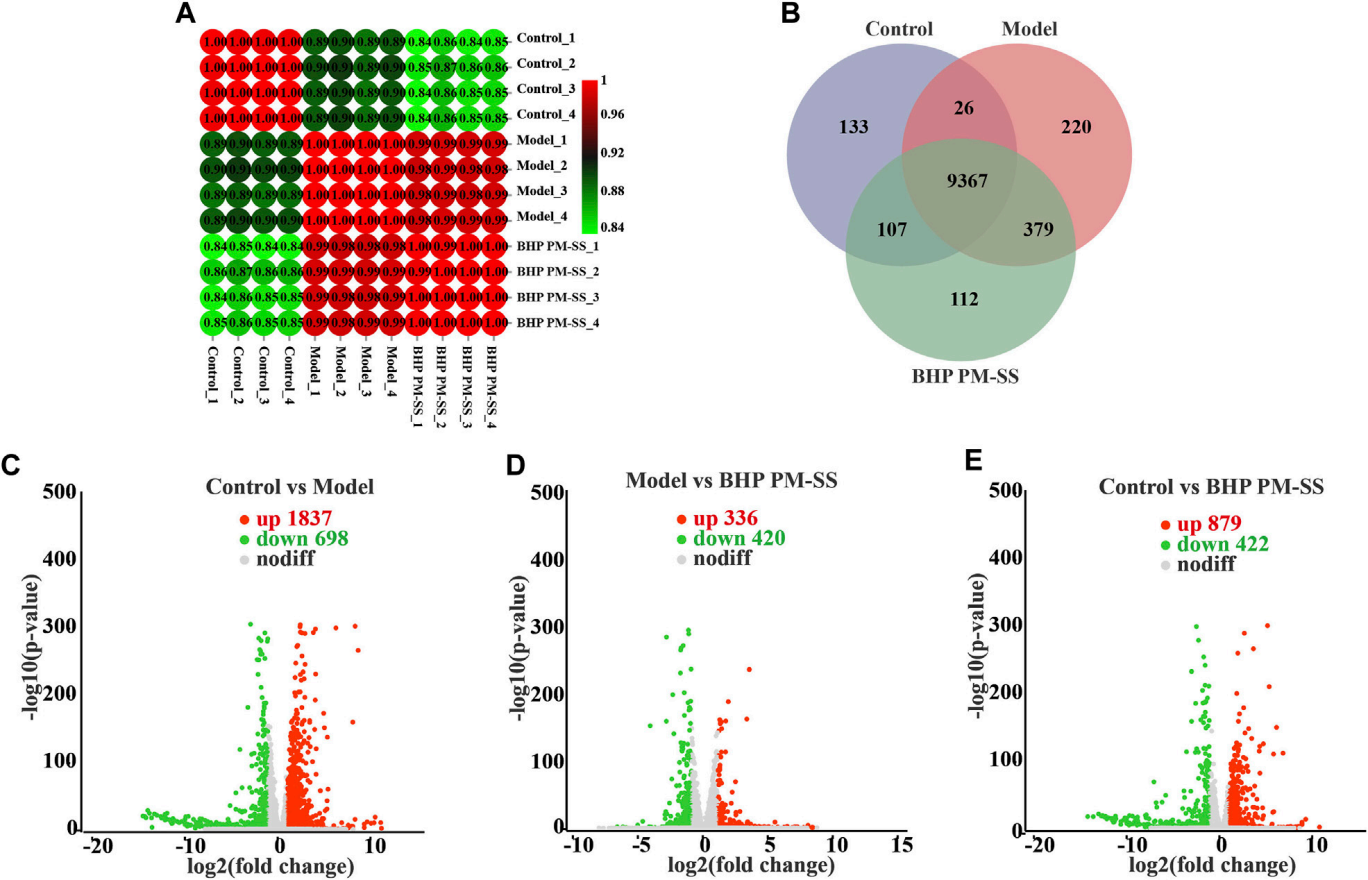
Effects of BHP PM-SS on CCl_4_-induced ALI mice by transcriptomic analysis. **(A)** Correlation analysis of samples from different experimental groups; **(B)** Venn analysis of different experimental groups; **(C–E)** Volcano plot of gene expression difference between different experimental groups, the red dots indicate significantly upregulated genes, the green dots indicate significantly downregulated genes.

The expressed genes between samples were analyzed by correlation analysis and Venn analysis. Figure 5A showed that correlation coefficients for all groups were above 0.8. Venn analysis in Figure 5B showed a total of 9367 genes in common and 133, 220 and 112 unique genes in the control, model and BHP PM-SS groups respectively. Genes meeting the criteria of fold change >2 and *p* < 0.05 were considered DEGs in each condition. Specifically, a large number of DEGs in the Control/Model, Model/BHP PM-SS and Control/BHP PM-SS comparisons showed a significant transcriptome profile caused by the injection of CCl_4_ and oral BHP PM-SS interventions (Control/Model: 1837 upregulated and 698 downregulated; Model/BHP PM-SS: 336 DEGs upregulated and 420 DEGs downregulated; Control/BHP PM-SS: 879 DEGs upregulated and 422 DEGs downregulated) (Supplementary Tables S8-S10). As clearly shown in the volcano plot, the points on the left and right are the down- and upregulated DEGs, respectively (Figure 5C–E). Next, STEM analysis was performed on these 4592 DEGs to analyse gene expression patterns. Three significant clusters were identified, which included 1206, 697 and 387 DEGs (Supplementary Figure S4A). Three significant clusters of DEGs showed almost opposite trends to the gene profile of the CCl_4_-induced ALI model group (Supplementary Figure S4D), suggesting that greater genetic changes occurred following CCl_4_ exploration, and that such changes could be prevented to some extent by early BHP PM-SS feeding. These data imply that pre-administration of BHP PM-SS prevented abnormal changes in the expression levels of some genes during the ALI process. These are consistent with our biochemical results.

#### 3.4.3 Functional annotation and enrichment analysis of Gene Set

**FIGURE 6:**
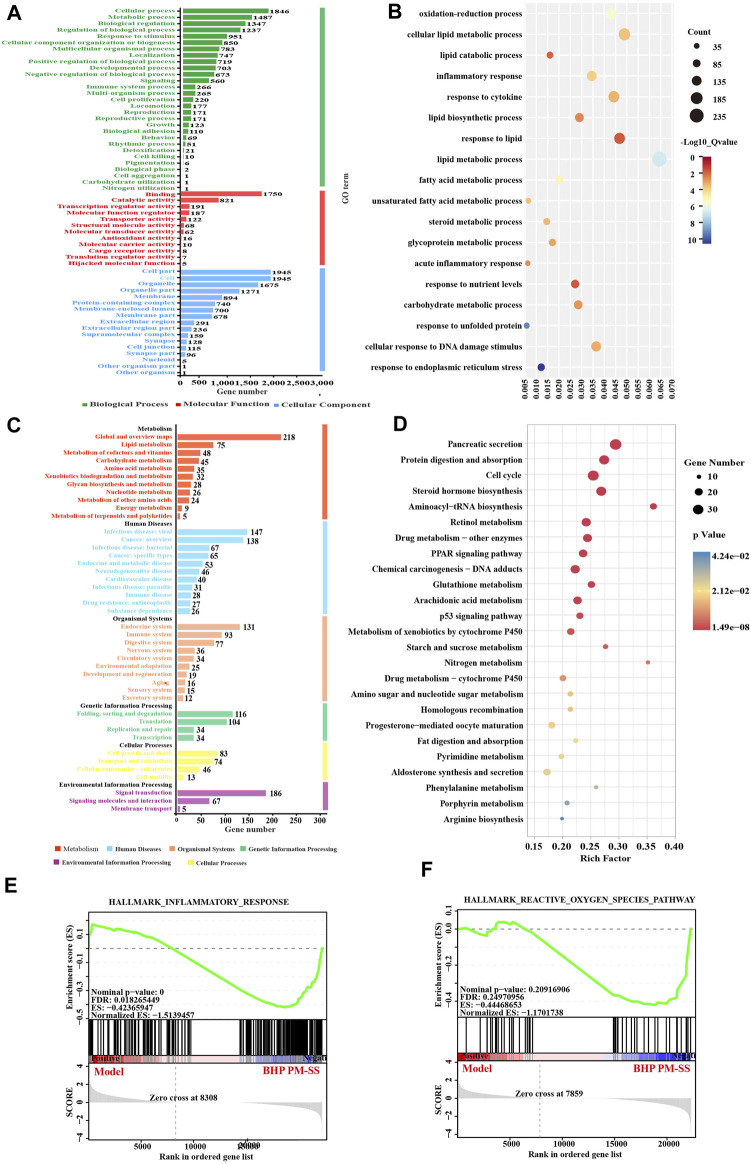
Pathway-enriched analysis of DEGs of STEM analysis. **(A)** GO annotation of Gene Set; **(B)** KEGG pathway annotation of Gene Set; **(C)** GO enrichment of Gene Set, and **(D)** KEGG pathway enrichment of Gene Set; **(E,F)** GSEA pathway enrichment analysis of pathways related to inflammation and Reactive oxygen species.

We constructed Gene Sets based on STEM analysis of DEGs. Gene Ontology (GO) classified Gene Set into three main types, including biological processes, cellular components, and molecular functions (Figure 6A). Similarly, KEGG pathway analysis divided Gene Set into six major types: metabolism, human diseases, organic systems, genetic information processing, environmental information processing, and cellular processes (Figure 6B).

The results of the GO enrichment analysis of Gene Set showed that the main functions of these genes were related to oxidative stress, inflammation, and lipid metabolism (Figure 6C), for instance, oxidation-reduction process, lipid catabolic process, inflammatory response, response to cytokine, lipid metabolic process, lipid biosynthetic process, and acute inflammatory response. Interestingly, KEGG analysis significantly enriched 39 metabolic pathways (*p* < 0.05), including PPAR signaling pathway, p53 signaling pathway, Aminoacyl-tRNA biosynthesis, Glutathione metabolism, Starch and sucrose metabolism, Arachidonic acid metabolism, Pyrimidine metabolism, Phenylalanine metabolism, Arginine biosynthesis, also associated with inflammation, oxidative stress and lipid metabolism (Figure 6D). GESA enrichment analysis showed an overall downregulation of inflammation and reactive oxygen species responses after BHP PM-SS intervention (Figures 6E,F).

### 3.5 BHP PM-SS-preconditioned and CCl_4_-induced changes in metabolomic profiles

**FIGURE 7:**
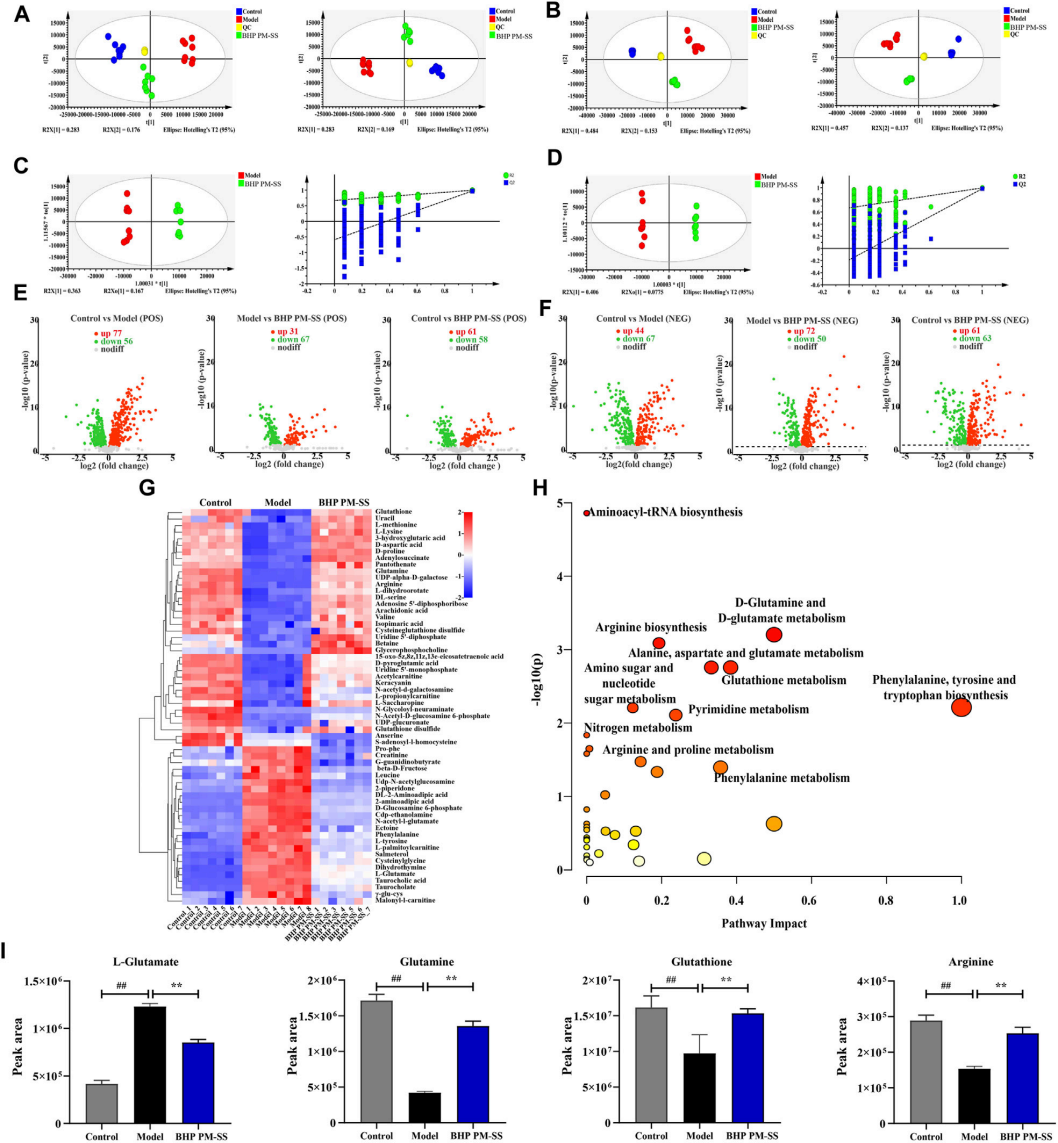
Effects of BHP PM-SS on the liver metabonomic profiling. **(A)** PCA score plot of the different groups in the positive ion (left) and negative ion (right); **(B)** PLS-DA score plot of the different groups in the positive ion (left) and negative ion (right); **(C,D)** OPLS-DA score diagrams of the samples from Model and BHP PM-SS in the positive mode and negative mode; **(E,F)** Volcano plot of the DMs between control group, model group and BHP PM-SS group in the positive ion and the negative ion; **(G)** Heatmap visualization of the intensities of 60 metabolic biomarkers in different groups; **(H)** The metabolic pathway impact prediction between groups based on the KEGG online database; **(I)** Metabolites involved in relevant metabolic pathways showed significantly changed relative peak areas between Control, Model and BHP PM-SS.

To further reveal the physiological and molecular mechanisms of the BHP PM-SS response to ALI, BHP PM-SS (2:3) group samples were selected for liver metabolic profiling and transcriptomic analysis based on physiological and biochemical indicators. A total of 20,589 peaks (9590 and 10,999 ESI+ and ESI- ions, respectively) were detected in the metabolomics data. PCA and PLS-DA were used to differentiate between control, model and BHP PM-SS intervention mice for potential liver biomarkers. As shown in Figures 7A,B, the closely distributed QC samples illustrate instrument stability and result reliability. Samples from different groups in positive and negative ion mode were distinctly separated, indicating a clear distinction between the metabolite profiles of the liver of three groups. OPLS-DA was used to explore differences between BHP PM-SS and model groups. The explanatory power parameter (cumulative explanatory rate: R^2^X, R^2^Y) was close to 1 and the model’s predictive power parameter (Q^2^) was greater than 0.5, indicating superior reliability and predictive power. The values of R^2^X, R^2^Y and Q^2^ for the positive ion model were 0.78, 0.995 and 0.967 respectively, while the values for the negative ion model were 0.699, 0.993 and 0.972, indicating the excellent fitness and predictive power of the proposed model. The permutation test with 200 iterations showed that the OPLS-DA models were valid (Figures 7C,D).

For the OPLS-DA model, one principal component VIP (threshold >1) and a *t*-test P (threshold 0.05) were used to identify differential metabolites (DMs). Based on the above thresholds, volcano plots visually showed differential metabolite expression under the positive ion model (181 downregulated and 169 upregulated) and under the negative ion model (180 downregulated and 177 upregulated), as shown in Figures 7E,F. In addition, a total of 60 DMs in the liver were identified as biomarkers under the positive/negative model between the control, model and BHP PM-SS (2:3) groups (Supplementary Table S11), as shown in the heat map (Figure 7G). These results suggested to some extent that BHP PM-SS pretreatment could reverse certain biological processes that were interrupted by excess CCl_4_. These metabolites can be classified as amino acids, organic acids and derivatives, nucleotides and analogues, lipids, etc.

To achieve a deeper understanding of endogenous metabolite changes in liver upon BHP PM-SS intervention/non-intervention, the MetaboAnalyst platform was used for topological analysis of KEGG metabolic pathways, amino acid-tRNA biosynthesis, amino acid sugar and nucleotide sugar metabolism, d-glutamine and d-glutamate metabolism, arginine biosynthesis, glutathione metabolism, pyrimidine metabolism, and nitrogen metabolism. Phenylalanine metabolism, pantothenic acid and CoA biosynthesis, alanine, aspartate and glutamate metabolism were the main metabolic pathways of BHP PM-SS intervention in liver injury (Figure 7H). We found that BHP PM-SS significantly modulated the disruption of some key metabolites in ALI states (Figure 7I).

### 3.6 Integrated analyses of the metabolomics and transcriptomics data

**FIGURE 8:**
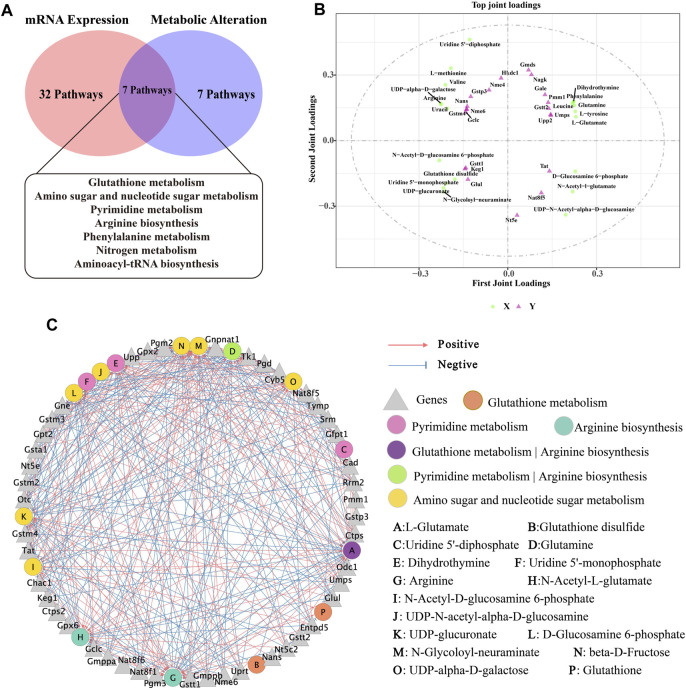
Correlation analysis of metabolomics and transcriptomics. **(A)** Common pathways of metabolomics and transcriptomics; **(B)** Correlation analysis of DMs and DEGs. In the picture, the green dots represent DMs, and the purple triangles represent DEGs. The top 20 DEGs and DMs with high correlation were marked in the figure; **(C)** Interaction network of DMs and DEGs involved in Glutathione metabolism, Pyrimidine metabolism, Arginine biosynthesis and Amino sugar and nucleotide sugar metabolism and based on spearman correlation.

Next, based on transcriptomic and metabolomic data, common enrichment pathways were obtained between DEGs and DMs (Figure 8A), mainly comprised of Glutathione metabolism, Pyrimidine metabolism, Arginine biosynthesis, Amino sugar and nucleotide sugar metabolism, Aminoacyl-tRNA biosynthesis, Nitrogen metabolism and Phenylalanine metabolism. Similar to the metabolic pathway analysis, pathways with Impact value >0.1 included Glutathione metabolism, Pyrimidine metabolism, Arginine biosynthesis and Amino sugar and nucleotide sugar metabolism were highly significantly enriched (*p* < 0.01). O2PLS is an unsupervised model that allows for bidirectional modelling and prediction in two data matrices and was used to mine the internal links between the two histologies and determine the degree of association between the two histological data (Li et al., 2013). The top 20 DEGs and DMs were predicted using the O2PLS model and contributed more to the degree of association between the two omics (Figure 8B). The analysis showed that the DMs and DEGs with a greater degree of association were mainly enriched in the Glutathione metabolism, Pyrimidine metabolism, Arginine biosynthesis and Amino sugar and nucleotide sugar metabolism pathways. These DMs and DEGs were down- or upregulated after oral administration of BHP PM-SS, showing different effects from the Model group. Based on this, we hypothesized that modulation of these major metabolisms could significantly interfere with CCl_4_-induced liver injury. Similar results have been reported in other studies. Some authors have suggested that Glutathione metabolism, Pyrimidine metabolism, Arginine biosynthesis and Amino sugar and nucleotide sugar metabolism pathways were reorganized to reallocate resources to defensive metabolic pathways that this protects the liver from exogenous stimulus.

To further investigate the relationship between DEGs and DMs under CCl_4_ induction and BHP PM-SS pretreatment, |r| >0.85 and *p* < 0.01 were used as screening criteria for co-expression network, which consists of 16 DMs and 45 DEGs (Figure 8C). The results indicated that BHP PM-SS attenuated CCl_4_-induced ALI may be involved in a complex network regulation relationship with the Glutathione metabolism, Pyrimidine metabolism, Arginine biosynthesis and Amino sugar and nucleotide sugar metabolism, which promote accumulation or reduction of these metabolites, thereby increasing hepatic resistance.

### 3.7 Validation of gene expressions by RT-PCR

Subsequently, relative expressions of 8 key unigenes involved in Glutathione metabolism, Pyrimidine metabolism, Arginine biosynthesis and Amino sugar and nucleotide sugar metabolism were verified by RT-PCR (Supplementary Figure S5). On the whole, these results are consistent with those from transcriptome sequencing, the importance of key genes of critical pathways in BHP PM-SS to exert hepatoprotective capacity.

## 4 Discussion

### 4.1 Chemical interactions of BHP PM-SS and its anti-ALI activity evaluation

As a key feature of Chinese medicine, BHP enhances the efficacy through synergistic effect (Panossian et al., 2024). Our results suggested that the BHP PM-SS is superior to PM or SS alone in ALI. Some studies have demonstrated potential mechanisms of synergism related to increased extraction of active ingredients, complex physicochemical reactions between ingredients, multi-targeting behaviour or increased bioavailability (Deng et al., 2008; Xu et al., 2022). In this study, it was found that the dissolution rate of 3′-hydroxy Puerarin, Puerarin, Puerarin apigenin, Daidzin and Daidzein from PM increased after combined decoction with SS when the ratio of SS was greater than 50%, such as 1:1, 1:2 and 2:3. This may be attributed to the acidic nature of SS during the decoction process altered the pH of the solvent environment. It has been reported that the cell membranes of flavonoids are more susceptible to disruption in acidic conditions, resulting in higher dissolution rates (González-de-Peredo et al., 2021). This may further explain the increased dissolution rate of components in PM. The overall desirability (OD) could reflect the integrated effect of the multi-indicator evaluation. When the OD value is closer to 1, the integrated effect is stronger and more favourable to the overall components dissolution. Our results found that BHP PM-SS (2:3) had the highest OD value, indicating that the all ingredients dissolution was favoured at 2:3 ratio and the synergistic effect was optimal. Further, pharmacodynamic experiments confirmed that the preventive effects of BHP PM-SS were superior to PM or SS alone in alleviating pathological injury, improving the abnormalities of liver function indexes, and suppressing the levels of oxidative stress and inflammation in ALI mice. However, the differences effect among different ratios of BHP PM-SS were attributed to that the content of the components during BHP PM-SS varied significantly with the ratio of PM and SS, which may directly contribute to the differences in synergistic effects. In particular, BHP PM-SS (2:3) may be the optimal combination with the preventive effect against CCl_4_-induced ALI.

### 4.2 Symptom-oriented network pharmacology constructs multiple pathways of BHP PM-SS against ALI

Most of the network pharmacology studies conducted search for targets from a disease perspective, whereas this study differed from other studies by searching for targets in a symptom-oriented approach. Network pharmacological results showed that the main active ingredients in BHP PM-SS, such as Puerarin, Daidzin, Daidzein, Schisantherin A, Schisandrin A, Schisandrin C, Schisandrin B, Schisandrol A and Schisandrol B, can regulate the Pathways in cancer, PI3K-AKT signaling pathway and MAPK signaling pathway by acting on the targets of AKT1, TNF, EGFR, JUN, HSP90AA1, and STAT3, thereby ameliorating the “jaundice”, “abdominal distension”, “fatigue”, “CINV” and “poor appetite” associated with ALI.

AKT1 is a serine/threonine kinase that is widely expressed in cells, and is involved in signaling pathways related to cell proliferation, cell growth, metabolism, oxidative stress, and inflammation. Inhibition of AKT1 phosphorylation reduces cytokine production during inflammation, thereby attenuating tissue damage and inflammatory responses (Zhang et al., 2023). We found that AKT1 was inhibited by multiple components in BHP PM-SS to exert anti-hepatic inflammatory effects (Zhang et al., 2015; Wang et al., 2019). Previous studies have found that liver injury leads to dysregulation of intestinal flora and host bile acid (BA) metabolism, causing irritable bowel, and therefore leading to abdominal distension (Lee et al., 2016; Du et al., 2023). Inhibition of the PI3K/AKT signaling pathway can reduce visceral sensitivity in the irritable bowel state, thereby alleviating abdominal distension (Fei and Wang, 2020). The current view suggests that inflammation is one of the important causes of jaundice in liver cell injury (Wang et al., 2021). Signal transducer and activator of transcription 3 (STAT3) is a key signaling molecule in inflammation. Studies have showed that STAT3 activation helped to restore hepatic bile abnormalities in ALI mice (Chen et al., 2015). Oxidative stress-induced activation of EGFR, which impairs mitochondrial oxidative phosphorylation and respiratory capacity, leads to mitochondrial oxidative damage, which results in disturbed and abnormal ingestive behaviours in peptidergic neurons in the arcuate nucleus of the hypothalamus, and may lead to poor appetite (Lindfors et al., 2011). A recent study demonstrated that Schisandrin B attenuates liver injury by downregulating EGFR protein expression (Li et al., 2023). Previous studies have identified HSP90AA1 as a key target gene for chemical toxin-induced liver injury, which is consistent with our results (Jia et al., 2021). HSP90AA1 promotes NLRP3 inflammasome activity during hepatic infections and inflammatory diseases, leading to the release of IL-6 and TNF-α (Choudhury et al., 2020). Thus, inhibition of HSP90AA1 helps to reduce hepatic inflammatory injury. Another pathway closely associated with ALI is the MAPK signaling pathway, which together with the PI3K-Akt signaling pathway, transduces inflammation, differentiation, proliferation or apoptosis. It was found that blockade of p38-MAPK prevented the accumulation of ROS induced by CCl_4_ and inhibited the inflammatory response in order to promote hepatic repair of ALI (Wang et al., 2018). It also found that the components in PL and SS act on the MAPK signaling pathway thereby reducing the inflammatory response (Wu et al., 2016; Chen et al., 2023). Molecular docking of key components with key targets showed that these active components have strong binding activities with AKT1, TNF, EGFR, JUN, HSP90AA1 and STAT3, which may play an important role in the prevention and treatment of ALI. Other compounds, key targets and signaling pathways predicted in this study are also valuable to study for the prevention and treatment of ALI. In addition, although literature and network pharmacological predictions suggested a connection between core targets, representative components and symptoms, there was a lack of relevant experimental to confirm this. These shortcomings need to be further explored.

### 4.3 Combined transcriptomics, metabolomics and symptom-oriented network pharmacology analysis

Through analysis of metabolomics-related networks of DMs and DEGs, the key genes associated with glutathione metabolism, pyrimidine metabolism, arginine biosynthesis, and amino acid sugar and nucleotide sugar metabolism were screened for further attention. This may indicate a stronger effect of ALI on the expression of genes for amino acid, nucleotide and carbohydrate metabolism in the mouse liver, leading to severe metabolic damage in the mouse liver, whereas BHP PM-SS improved amino acid, nucleotide and carbohydrate metabolism. Notably, the various disturbed genes and metabolites are all part of the “glutathione metabolism” pathway. With regard to glutathione metabolism, *Gstt1* and *Gstp3* are known to prevent oxidative stress and DNA damage (Awdishu et al., 2020). In this study, the simultaneous reduction in mRNA levels of these two genes following CCl_4_ intervention may have contributed to the onset of oxidative stress. In addition, other genes involved in biochemical reactions downstream of glutathione metabolism, *Gpx7*, *Ggct*, *Gstp3*, *Gpx2*, *Chac1*, *Gpx6* and *Gclc*, were decreased in expression in the model group, suggesting a deregulation of redox homeostasis (Yadav et al., 2019; He et al., 2021; Tian et al., 2021; Zhou et al., 2021). We found several key genes associated with glutathione metabolism, including *Gclc*, *Gpx7*, *Chac1* and *Gstt1*, which were negatively correlated with L-Glutamate. These genes play an important role in the scavenging of reactive oxygen species. Pyrimidine metabolism plays a key role in maintaining cellular function and energy metabolism, and its uracil and the derivatives also contribute to the reduction of cytotoxicity and inhibition of drug-induced hepatic steatosis (Zhang Y et al., 2020; Xiong et al., 2022). Interestingly, we found that the expression of *Entpd5*, *Upp2*, *Tk1*, *Nme6* and *Tymp*, genes involved in biochemical reactions downstream of deoxycytidine and thymine metabolism, was significantly downregulated and *Cad* and *Rrm2* were significantly upregulated in the CCl_4_ group compared to the control group. Moreover, the reduced levels of some pyrimidine derivatives (Uridine 5′-diphosphate, Uridine 5′-monophosphate and Uracil) further suggest that ALI mice are no longer able to supply deoxycytidine and thymidine involved in cellular DNA and RNA production, possibly leading to an uncontrolled stress response (Ng et al., 2015; Zhao et al., 2018).

Amino sugar and nucleotide sugar metabolism are closely related to ALI. In our study, we observed that although UDP-N-acetylglucosamine levels were increased in ALI mice, their levels were significantly reduced after BHP PM-SS administration. The results also showed a decrease in UDP-alpha-D-galactose and UDP-glucuronate levels, which implies abnormal glycosylation. It was reported to be related to the acute phase response of acidic glycoproteins mediated by CCl_4_ induced inflammatory response (Jamieson et al., 1983). Simultaneously D-glucosamine 6-phosphate levels were increased, suggesting that the liver may release lysosomal glycosidases during inflammation. Further studies revealed significantly higher mRNA levels of Amino sugar and nucleotide sugar metabolism, such as *Gmds*, *Gmppb*, *Nagk*, *Gnpnat1*, *Gfpt1*, *Gne* and *Cyb5r1*; while *Pgm2*, *Pgm3* and *Pmm1* were decreased in expression. The changes in these genes may suggest a potential link between BHP PM-SS protection of the liver and nucleotide metabolism (Stray-Pedersen et al., 2014; Ai et al., 2021). Previous studies have also confirmed that arginine metabolism is closely associated with metabolic disorders in liver-injured mice. Arginine is a multifunctional amino acid in animals and is a precursor to ornithine, urea, nitric oxide and sarcosine. It is considered a candidate biomarker for liver injury and arginine has been found to be reduced in liver injury caused by liver toxins such as carbon tetrachloride. Our results also show induced gene expression of *Gpt2*, an enzyme encoding alanine transaminase, which forms glutamate and pyruvate by activating a reversible ammonification reaction between 2-oxaloglutarate and alanine, with elevated glutamate levels leading to a decrease in arginine due to oxidative stress (Southan et al., 2020). Fortunately, BHP PM-SS contributed to the restoration of arginine levels.

**FIGURE 9:**
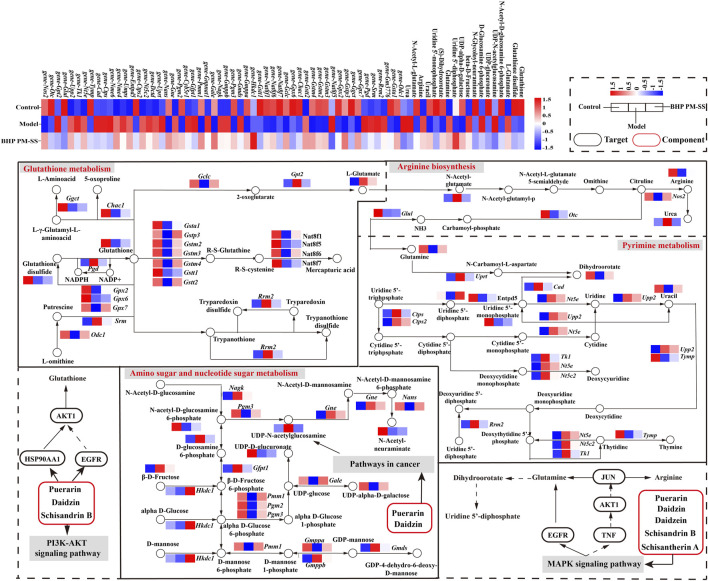
Combining symptom-oriented network pharmacology, transcriptomics and metabolomics analysis of the complex pathways of BHP PM-SS against ALI.

Previous studies have indicated that Schisantherin A can attenuate tissue damage caused by oxidative stress, inflammation and apoptosis through activation of the PI3K/AKT signalling pathway and inhibition of the MAPK signalling pathway (Zheng et al., 2017; Mi et al., 2023). In addition, a previous study found that Schisandrin B inhibits Kupffer cell polarization by downregulating the NF-κB and p38 MAPK signaling pathways for ameliorates CCl_4_ induced hepatic fibrosis (Wang H Q et al., 2022). The other study revealed that Schisandrin B could effectively alleviate CCl_4_ induced hepatic injury and fibrosis in rats by regulating glutathione metabolic pathway as well as metabolic, redox, endoplasmic reticulum stress and apoptosis-related differential genes (Zhang et al., 2019). In conclusion, based on previously reported results, Puerarin, Daidzin, Daidzein, Schisantherin A and Schisandrin B are involved in the regulation of the PI3K-AKT signaling pathway, the MAPK signaling pathway and the Pathway in cancer, thus acting on the AKT1, TNF, EGFR, JUN, HSP90AA1 and STAT3, and indirectly regulate the expression of metabolites and related genes such as Glutathione, Glutamine, Dihydroorotate and Arginine (Li et al., 2014; Leong et al., 2016; Liao et al., 2016; Li S L et al., 2021). These results revealed a multi-component, multi-target and multi-pathway mechanism of action of BHP PM-SS on ALI mice (Figure 9).

## 5 Conclusion

In conclusion, by integrating chemical analysis, pharmacological evaluation, symptom-oriented network pharmacology, transcriptomics and metabolomics, the compatibility characteristics and mechanism for BHP PM-SS against ALI were systematically elucidated from a holistic perspective. After compatibility, the content of some active substances increased, and BHP PM-SS (2:3) showed better anti-ALI effects. To our knowledge, the active ingredients of BHP PM-SS may regulate Glutathione metabolism, Pyrimidine metabolism, Arginine biosynthesis and Amino acid sugar and nucleotide sugar metabolism to ameliorate ALI, by acting on the targets of AKT1, TNF, EGFR, JUN, HSP90AA1 and STAT3, which partly occurred in PI3K-AKT signaling pathway, MAPK signaling pathway and Pathway in cancer.

## Data Availability

The datasets presented in this study can be found in online repositories. The names of the repository/repositories and accession number(s) can be found in the article/Supplementary Material.
