# Supplementary material for: Evidence of synergistic mechanisms of hepatoprotective botanical herbal preparation of Pueraria montana var. lobata and Schisandra sphenanthera
Source: Front Pharmacol. 2024 Jun 24;15:1412816. doi: 10.3389/fphar.2024.1412816 (PMC11228302; doi:10.3389/fphar.2024.1412816)
Supplement: Supplementary file 1 [file DataSheet1.docx]

**Supplementary material**

**Table S1** Parameter conditions of mass spectrometry

| Project | Parameter |
| --- | --- |
| Ion Source Gas1 | 60 |
| Ion Source Gas2 | 60 |
| curtain gas | 30 |
| source temperature | 600 ℃ |
| Scan range | 25-1000 Da |
| IonSpray Voltage Floating | ±5500 V |
| product ion scan accumulation time | 0.05 s/spectra |
| TOF MS scan accumulation time | 0.20 s/spectra |
| collision energy | 35 V ± 15 eV |
| declustering potential | ±60 V |
| candidate ions to monitor per cycle | 10 |

**Table S2** Mobile phase condition parameters in ESI positive mode (Injection volume: 2 μL, flow rate of 0.3 mL/min, and column temperatures at 25 ℃)

| Time (min) | A (0.1% formic acid in water) % | B (0.1% formic acid in acetonitrile)% |
| --- | --- | --- |
| 0 | 99 | 1 |
| 1.5 | 99 | 1 |
| 13 | 1 | 99 |
| 16.5 | 1 | 99 |
| 16.6 | 99 | 1 |
| 20 | 99 | 1 |

**Table S3** Mobile phase condition parameters in ESI negative mode (Injection volume: 2 μL, flow rate of 0.3 mL/min, and column temperatures at 25 ℃.)

| Time (min) | A(0.5 mM ammonium fluoride in water) % | B(acetonitrile) % |
| --- | --- | --- |
| 0 | 99 | 1 |
| 1.5 | 99 | 1 |
| 13 | 1 | 99 |
| 16.5 | 1 | 99 |
| 16.6 | 99 | 1 |
| 20 | 99 | 1 |

**Table S4** The primer sequences used for real-time PCR assay in mice.

| **Gene** | **Forward primer (5’-3’)** | **Reverse primer (5’-3’)** |
| --- | --- | --- |
| Gclc | GCACATCTACCACGCAGTC | TGGTTGGGGTTTGTCCTC |
| Gstt1 | GGCTCGTGCTCGTGTAGA | CCAGTGTTTCAGGAGGTATT |
| Entpd5 | AGACTGTGGGGACCCTTGA | TCCATCAGTCCCTTTTGC |
| Gpx7 | CCGAGCAGGACTTCTACG | AAGGGAAGGCAAGCACAT |
| Cyb5r1 | GTGAGCCACAACACCAGG | TCATTCCCAATTTCTTCG |
| Otc | CCTGGCTGATTACCTTAC | TTCTCATCCTCTTGTCCC |
| Nme6 | CATTGTACGAACGAGGGA | TGTTAGGTTGTTTGTGGC |
| Chac1 | CACTCCGCCTCCTTCACT | GTTGGTCAGGGGTGTCTT |
| GAPDH | GCACATCTACCACGCAGTC | TGGTTGGGGTTTGTCCTC |

**Table S5** Linear relationship of 11 components in BHP PM–SS

| Component | Regression equation | R^2^ | Linear range/μg |
| --- | --- | --- | --- |
| 3'-Hydroxy Puerarin | y = 30012x + 20.024 | 0.9991 | 3.81-244.00 |
| Puerarin | y = 37796x + 128.99 | 0.9991 | 7.78-498.00 |
| Puerarin apigenin | y = 28050x + 37.76 | 0.9992 | 3.94-126.00 |
| Daidzin | y = 35523x + 46.664 | 0.9992 | 3.19-204.00 |
| Daidzein | y = 54974x + 77.816 | 0.9990 | 5.63-360.00 |
| Schisandrol A | y = 18958x + 37.078 | 0.9994 | 4.17-267.00 |
| Schisandrol B | y = 19777x + 29.028 | 0.9993 | 2.13-136.00 |
| Schisantherin A | y = 12191x - 8.9224 | 0.9991 | 1.92-123.00 |
| Schisandrin A | y = 19798x + 8.4511 | 0.9993 | 1.97-126.00 |
| Schisandrin B | y = 16572x - 3.6124 | 0.9992 | 2.28-146.00 |
| Schisandrin C | y = 21290x - 4.3443 | 0.9993 | 1.80-115.50 |

**Table S6** Contents of 11 chemical components of BHP PM-SS in different ratios (mg/g, ‾x±s, n=3)

| Samples | Contents (mg/g) | | | | | | | | | | |
| --- | --- | --- | --- | --- | --- | --- | --- | --- | --- | --- | --- |
|  | 3'-Hydroxy Puerarin | Puerarin | Puerarin apigenin | Daidzin | Daidzein | Schisandrol A | Schisandrol B | Schisantherin A | Schisandrin A | Schisandrin B | Schisandrin C |
| BHP PM-SS (0-1) | / | / | / | / | / | 1.181±0.028 | 3.150±0.049 | 2.868±0.028 | 3.550±0.038 | 1.841±0.016 | 0.434±0.021 |
| BHP PM-SS (1-0) | 13.265±0.053 | 43.201±0.133 | 11.994±0.046 | 24.360±0.043 | 1.609±0.013 | / | / | / | / | / | / |
| BHP PM-SS (1-1) | 12.375±0.086 | 38.918±0.073 | 10.561±0.050 | 19.684±0.041 | 1.224±0.018 | 0.928±0.025 | 2.399±0.031 | 3.254±0.027 | 4.405±0.039 | 2.549±0.038 | 0.651±0.031 |
| BHP PM-SS (1-2) | 13.317±0.034 | 40.630±0.052 | 12.095±0.042 | 21.057±0.053 | 1.610±0.015 | 1.229±0.018 | 2.786±0.028 | 3.527±0.028 | 2.892±0.040 | 2.185±0.014 | 0.503±0.032 |
| BHP PM-SS (2-1) | 11.206±0.093 | 38.786±0.046 | 9.931±0.042 | 18.337±0.049 | 0.747±0.012 | 1.175±0.019 | 2.060±0.022 | 4.657±0.022 | 4.638±0.033 | 2.628±0.012 | 0.557±0.023 |
| BHP PM-SS (2-3) | 13.738±0.054 | 44.251±0.065 | 13.165±0.042 | 24.927±0.053 | 1.848±0.013 | 0.961±0.027 | 2.937±0.026 | 5.259±0.082 | 5.144±0.063 | 2.587±0.022 | 1.058±0.029 |
| BHP PM-SS (3-2) | 10.848±0.114 | 35.353±0.032 | 9.886±0.047 | 19.093±0.039 | 1.017±0.012 | 1.091±0.032 | 2.394±0.032 | 4.270±0.029 | 4.297±0.021 | 1.989±0.029 | 0.637±0.032 |

**Table S7** Summary statistics for quality control of the sequencing data and clean reads was mapped to the reference genome.

| Sample | RawDatas | CleanData (%) | Q20 (%) | Q30 (%) | GC Content (%) | Total reads | Mapped reads (%) | Unique_Mapped(%) | Multiple_Mapped(%) |
| --- | --- | --- | --- | --- | --- | --- | --- | --- | --- |
| Control-1 | 39098848 | 38938718 (99.59%) | 97.50% | 92.89% | 49.18% | 38865474 | 37482589 (96.44%) | 32603824 (83.89%) | 4878765 (12.55%) |
| Control-2 | 38217826 | 38061332 (99.59%) | 97.57% | 93.06% | 48.51% | 37924116 | 36646128 (96.63%) | 31717113 (83.63%) | 4929015 (13.00%) |
| Control-3 | 45000102 | 44823342 (99.61%) | 97.83% | 93.61% | 48.35% | 44733962 | 43422570 (97.07%) | 37476058 (83.78%) | 5946512 (13.29%) |
| Control-4 | 44067254 | 43895706 (99.61%) | 97.51% | 92.78% | 48.43% | 43798178 | 42402832 (96.81%) | 36650121 (83.68%) | 5752711 (13.13%) |
| Model-1 | 42921084 | 42767232 (99.64%) | 97.78% | 93.41% | 49.75% | 42686920 | 41839756 (98.02%) | 38239609 (89.58%) | 3600147 (8.43%) |
| Model-2 | 44965128 | 44783404 (99.60%) | 97.46% | 92.79% | 49.47% | 44702468 | 43549230 (97.42%) | 39730242 (88.88%) | 3818988 (8.54%) |
| Model-3 | 45215408 | 45044080 (99.62%) | 97.59% | 93.08% | 49.56% | 44955080 | 43854976 (97.55%) | 40147304 (89.31%) | 3707672 (8.25%) |
| Model-4 | 43053128 | 42876740 (99.59%) | 97.60% | 93.11% | 49.47% | 42794142 | 41736688 (97.53%) | 38142569 (89.13%) | 3594119 (8.40%) |
| BHP PM-SS-1 | 49782448 | 49578126 (99.59%) | 97.64% | 93.21% | 49.79% | 49478004 | 48342941 (97.71%) | 44202262 (89.34%) | 4140679 (8.37%) |
| BHP PM-SS-2 | 44131344 | 43946990 (99.58%) | 97.54% | 92.99% | 49.26% | 43850834 | 42793200 (97.59%) | 38994396 (88.93%) | 3798804 (8.66%) |
| BHP PM-SS-3 | 45293026 | 45103120 (99.58%) | 97.75% | 93.45% | 49.45% | 45025422 | 44072317 (97.88%) | 40296810 (89.50%) | 3775507 (8.39%) |
| BHP PM-SS-4 | 40373848 | 40211222 (99.60%) | 97.52% | 92.91% | 49.44% | 40137990 | 39216336 (97.70%) | 35735947 (89.03%) | 3480389 (8.67%) |

| **Table S8**. The DEGs between the control and model group | | | |
| --- | --- | --- | --- |
| Symbol | log2(fc) | P Value | FDR |
| Ifrd1 | 4.084 | 0 | 0 |
| Cyp2a5 | 2.962 | 0 | 0 |
| Sgk1 | 3.117 | 0 | 0 |
| Ctsl | 2.520 | 0 | 0 |
| C6 | -1.660 | 0 | 0 |
| Keg1 | -2.523 | 0 | 0 |
| Ddit3 | 5.288 | 0 | 0 |
| Glul | -2.704 | 0 | 0 |
| Apcs | 3.755 | 0 | 0 |
| Atf3 | 6.588 | 0 | 0 |
| Lcn2 | 7.254 | 0 | 0 |
| Car3 | -2.684 | 0 | 0 |
| Plk3 | 3.148 | 0 | 0 |
| Fgl1 | 2.464 | 0 | 0 |
| Mt2 | 7.756 | 0 | 0 |
| Mt1 | 5.731 | 0 | 0 |
| Apoa4 | 3.948 | 0 | 0 |
| Trib3 | 7.625 | 0 | 0 |
| Srxn1 | 3.496 | 0 | 0 |
| Scd1 | -1.863 | 0 | 0 |
| Hsd3b5 | -6.078 | 0 | 0 |
| Elovl3 | -5.007 | 0 | 0 |
| Lifr | -2.092 | 0 | 0 |
| Fabp1 | -1.649 | 0 | 0 |
| Ces1d | -1.822 | 0 | 0 |
| Saa2 | 7.895 | 0 | 0 |
| Orm2 | 6.172 | 0 | 0 |
| Ppp1r3c | -2.738 | 0 | 0 |
| Selenbp2 | -3.399 | 0 | 0 |
| Mup11 | -2.003 | 0 | 0 |
| Saa1 | 6.456 | 0 | 0 |
| Mup19 | -3.352 | 0 | 0 |
| Mup9 | -2.413 | 0 | 0 |
| Cyp4v3 | -1.281 | 0 | 0 |
| Mup15 | -2.679 | 0 | 0 |
| Mup17 | -3.112 | 0 | 0 |
| Mup2 | -2.868 | 1.14E-306 | 5.07E-304 |
| Lrrc59 | 2.493 | 7.73E-306 | 3.34E-303 |
| Fgf21 | 8.401 | 9.81E-304 | 4.13E-301 |
| Hspa5 | 2.463 | 5.05E-303 | 2.07E-300 |
| Nupr1 | 6.334 | 4.48E-301 | 1.80E-298 |
| Ddit4 | 4.130 | 1.54E-299 | 6.02E-297 |
| Npc1 | 2.501 | 6.67E-295 | 2.55E-292 |
| Plpp5 | 3.899 | 2.69E-294 | 1.00E-291 |
| Igfbp1 | 2.674 | 8.39E-294 | 3.07E-291 |
| Aox3 | -1.322 | 9.56E-294 | 3.42E-291 |
| Srm | 2.998 | 7.23E-293 | 2.53E-290 |
| Mup12 | -1.979 | 1.01E-285 | 3.47E-283 |
| Acsl1 | -1.010 | 4.71E-285 | 1.58E-282 |
| Mup7 | -1.727 | 3.41E-282 | 1.12E-279 |
| Glo1 | -1.059 | 2.35E-281 | 7.58E-279 |
| Cln8 | 2.215 | 3.30E-275 | 1.04E-272 |
| Orm1 | 2.120 | 2.37E-273 | 7.35E-271 |
| Mup3 | -1.882 | 6.52E-269 | 1.98E-266 |
| Cyp2b10 | 8.726 | 1.15E-267 | 3.43E-265 |
| Hao1 | -1.789 | 9.09E-267 | 2.67E-264 |
| Cyp2c67 | -1.616 | 4.74E-262 | 1.37E-259 |
| Mup16 | -1.766 | 8.77E-262 | 2.49E-259 |
| Sqstm1 | 2.678 | 2.54E-259 | 7.09E-257 |
| Cyp7b1 | -1.323 | 1.34E-255 | 3.66E-253 |
| Ugt2b1 | -2.155 | 5.37E-254 | 1.45E-251 |
| Srd5a1 | -1.974 | 1.53E-253 | 4.06E-251 |
| Stbd1 | 2.008 | 1.12E-248 | 2.91E-246 |
| Gmppb | 3.009 | 6.23E-247 | 1.60E-244 |
| Ddx28 | 2.790 | 1.04E-235 | 2.63E-233 |
| Gpat3 | 4.150 | 1.19E-232 | 2.97E-230 |
| Tsc22d1 | -2.057 | 4.17E-232 | 1.02E-229 |
| Dnajb9 | 2.500 | 1.05E-231 | 2.54E-229 |
| Gpt2 | 1.932 | 1.61E-227 | 3.84E-225 |
| Serpina3n | 2.908 | 1.48E-225 | 3.47E-223 |
| Slc20a1 | 2.597 | 1.65E-223 | 3.82E-221 |
| Gm4952 | -1.725 | 9.30E-213 | 2.12E-210 |
| Creld2 | 2.484 | 4.35E-206 | 9.79E-204 |
| Gstm3 | 2.298 | 7.56E-206 | 1.68E-203 |
| Trabd | 1.892 | 6.23E-205 | 1.37E-202 |
| Preb | 1.976 | 5.24E-200 | 1.13E-197 |
| Cad | 2.985 | 6.02E-198 | 1.29E-195 |
| Cyp4a12a | -1.621 | 1.34E-197 | 2.82E-195 |
| Asns | 4.105 | 6.87E-194 | 1.43E-191 |
| Nrn1 | -1.213 | 1.33E-189 | 2.74E-187 |
| Mup22 | -1.396 | 2.03E-189 | 4.11E-187 |
| Mup20 | -1.396 | 1.02E-184 | 2.05E-182 |
| Cyp2c40 | -3.156 | 5.03E-183 | 9.95E-181 |
| Tmem62 | 2.665 | 8.86E-183 | 1.73E-180 |
| Ces1f | -1.382 | 5.81E-182 | 1.12E-179 |
| Itih3 | 2.080 | 6.33E-179 | 1.21E-176 |
| Mup14 | -1.490 | 7.41E-177 | 1.40E-174 |
| Cyp2a22 | 5.011 | 3.04E-174 | 5.68E-172 |
| Sec24d | 2.180 | 4.63E-174 | 8.55E-172 |
| Sec61a1 | 2.048 | 7.42E-173 | 1.35E-170 |
| Mup1 | -1.494 | 2.47E-172 | 4.46E-170 |
| Kdelr2 | 1.868 | 4.31E-167 | 7.70E-165 |
| Srprb | 2.210 | 5.66E-166 | 1.00E-163 |
| Lipc | -1.674 | 3.20E-165 | 5.60E-163 |
| Slc22a30 | -1.510 | 3.14E-164 | 5.44E-162 |
| Arfgap3 | 2.918 | 1.35E-163 | 2.31E-161 |
| Abcc4 | 3.505 | 2.76E-163 | 4.68E-161 |
| Arf4 | 1.623 | 6.49E-163 | 1.09E-160 |
| Cdkn1a | 2.736 | 4.16E-161 | 6.90E-159 |
| Bhlha15 | 8.152 | 4.34E-161 | 7.14E-159 |
| Anxa7 | 1.534 | 9.39E-161 | 1.53E-158 |
| Fkbp11 | 3.506 | 9.79E-161 | 1.58E-158 |
| Cyp3a11 | -1.398 | 4.40E-160 | 7.03E-158 |
| Manf | 2.141 | 3.28E-159 | 5.19E-157 |
| Slc3a2 | 2.672 | 8.51E-158 | 1.33E-155 |
| Hsd3b3 | -1.511 | 1.60E-156 | 2.48E-154 |
| Atf4 | 1.735 | 1.24E-153 | 1.89E-151 |
| Nars | 1.779 | 3.17E-153 | 4.74E-151 |
| Cyp1a1 | 5.094 | 9.88E-153 | 1.46E-150 |
| Serpina3m | 1.788 | 6.41E-151 | 9.41E-149 |
| Myc | 4.041 | 3.46E-150 | 5.04E-148 |
| Slc17a2 | -1.226 | 1.21E-149 | 1.75E-147 |
| Cyp2c23 | -1.495 | 2.49E-149 | 3.55E-147 |
| Alas1 | 2.135 | 2.57E-149 | 3.64E-147 |
| Ttc39c | -1.283 | 1.89E-147 | 2.65E-145 |
| Cyp2c70 | -1.003 | 2.49E-147 | 3.47E-145 |
| Gfpt1 | 2.923 | 6.64E-147 | 9.17E-145 |
| Tbrg1 | 1.951 | 9.79E-146 | 1.34E-143 |
| Entpd5 | 1.505 | 1.02E-144 | 1.39E-142 |
| Fndc3b | 2.301 | 6.75E-144 | 9.10E-142 |
| Mup21 | -2.083 | 1.72E-143 | 2.30E-141 |
| Tsku | 2.143 | 1.02E-142 | 1.36E-140 |
| Dio1 | -1.385 | 1.70E-142 | 2.23E-140 |
| Slc35d1 | 1.634 | 6.60E-142 | 8.61E-140 |
| Hyou1 | 1.948 | 1.12E-141 | 1.44E-139 |
| Mknk1 | 2.387 | 7.22E-141 | 9.27E-139 |
| Inmt | -1.517 | 1.11E-140 | 1.42E-138 |
| Epb41 | 1.442 | 7.81E-140 | 9.87E-138 |
| Serpina7 | 2.540 | 2.63E-139 | 3.28E-137 |
| Gtpbp2 | 2.460 | 3.12E-139 | 3.86E-137 |
| Serp1 | 1.679 | 5.95E-139 | 7.30E-137 |
| Psat1 | 5.410 | 1.20E-138 | 1.47E-136 |
| Rbm3 | 2.298 | 2.96E-138 | 3.58E-136 |
| Cyb5r1 | 3.431 | 6.82E-138 | 8.18E-136 |
| Clint1 | 1.841 | 1.35E-135 | 1.59E-133 |
| Derl1 | 1.766 | 4.91E-135 | 5.76E-133 |
| Morf4l2 | 1.503 | 7.63E-135 | 8.89E-133 |
| Yipf5 | 1.916 | 2.59E-134 | 3.00E-132 |
| Tmed3 | 2.906 | 1.88E-133 | 2.16E-131 |
| Armcx3 | 3.288 | 1.28E-129 | 1.46E-127 |
| Hamp | 2.245 | 2.92E-129 | 3.31E-127 |
| Dhdds | 2.274 | 3.08E-129 | 3.47E-127 |
| Arhgef2 | 2.752 | 3.96E-129 | 4.43E-127 |
| Ssr1 | 1.617 | 6.51E-129 | 7.23E-127 |
| Ces1e | -1.232 | 1.47E-128 | 1.62E-126 |
| Cyp4a12b | -1.960 | 3.55E-127 | 3.86E-125 |
| Ugt3a1 | -1.203 | 2.84E-126 | 3.05E-124 |
| Slc17a9 | 3.563 | 9.77E-125 | 1.04E-122 |
| Gnpnat1 | 1.857 | 3.56E-124 | 3.78E-122 |
| Pgm3 | 2.530 | 7.93E-124 | 8.35E-122 |
| Slc30a10 | -1.421 | 1.15E-123 | 1.20E-121 |
| Selenos | 1.808 | 3.52E-123 | 3.66E-121 |
| Mup18 | -1.681 | 1.80E-122 | 1.85E-120 |
| Bcl6 | -1.795 | 3.88E-122 | 3.96E-120 |
| Palld | -1.087 | 9.09E-122 | 9.22E-120 |
| Rgn | -1.094 | 1.22E-121 | 1.23E-119 |
| Cidec | 4.207 | 1.67E-121 | 1.67E-119 |
| Cyp2f2 | -1.623 | 4.67E-121 | 4.65E-119 |
| Slc22a28 | -4.011 | 3.08E-120 | 3.05E-118 |
| Mcfd2 | 1.324 | 4.80E-120 | 4.73E-118 |
| Kyat1 | 1.698 | 3.57E-119 | 3.49E-117 |
| Acat3 | -1.042 | 4.61E-119 | 4.48E-117 |
| Tmem214 | 1.868 | 1.17E-118 | 1.13E-116 |
| Adora1 | 2.246 | 2.54E-118 | 2.42E-116 |
| Iars | 1.447 | 3.74E-118 | 3.55E-116 |
| Extl1 | 3.324 | 2.83E-116 | 2.66E-114 |
| Hsd17b11 | -1.036 | 3.32E-116 | 3.10E-114 |
| Atf5 | 2.085 | 2.88E-115 | 2.67E-113 |
| Etnppl | -1.280 | 3.52E-115 | 3.25E-113 |
| Serpina12 | -2.477 | 8.96E-115 | 8.23E-113 |
| Ces3b | -1.598 | 2.87E-114 | 2.62E-112 |
| Stt3a | 1.509 | 3.70E-114 | 3.36E-112 |
| Arrdc3 | -1.841 | 1.54E-113 | 1.38E-111 |
| Susd4 | -2.759 | 3.88E-113 | 3.47E-111 |
| Septin11 | 2.399 | 1.97E-112 | 1.75E-110 |
| Ces2e | -1.516 | 1.56E-111 | 1.38E-109 |
| C9orf72 | 2.553 | 3.57E-111 | 3.13E-109 |
| Eif4ebp3 | 1.989 | 2.93E-110 | 2.56E-108 |
| 6430548M08Rik | 2.316 | 8.27E-110 | 7.19E-108 |
| Pabpc4 | 1.910 | 1.13E-109 | 9.74E-108 |
| Uba5 | 2.467 | 1.57E-109 | 1.35E-107 |
| Htatip2 | 2.000 | 3.49E-109 | 2.99E-107 |
| Epas1 | -1.012 | 5.41E-109 | 4.60E-107 |
| Erlin1 | 1.729 | 1.35E-107 | 1.14E-105 |
| Hsd3b2 | -2.026 | 7.13E-107 | 5.95E-105 |
| Gars | 1.593 | 2.45E-106 | 2.03E-104 |
| Hsd11b1 | -1.279 | 5.89E-106 | 4.86E-104 |
| Rnf185 | 1.707 | 9.96E-106 | 8.19E-104 |
| Prune1 | 2.133 | 1.93E-105 | 1.57E-103 |
| S100a10 | 1.662 | 4.45E-105 | 3.60E-103 |
| Gosr2 | 1.426 | 1.06E-104 | 8.58E-103 |
| Serpina1e | -1.231 | 1.64E-104 | 1.32E-102 |
| Ank3 | 2.392 | 1.80E-104 | 1.44E-102 |
| Gstm4 | 2.163 | 5.11E-104 | 4.05E-102 |
| Wdr77 | 1.614 | 1.44E-103 | 1.14E-101 |
| Nme6 | 2.742 | 1.58E-103 | 1.25E-101 |
| Lars | 2.055 | 2.87E-103 | 2.25E-101 |
| Rint1 | 2.038 | 1.97E-102 | 1.53E-100 |
| C130074G19Rik | -1.092 | 3.34E-102 | 2.59E-100 |
| Acot2 | 3.813 | 3.37E-102 | 2.60E-100 |
| Ugt1a9 | -1.317 | 3.80E-102 | 2.90E-100 |
| Gstm2 | 1.895 | 1.40E-100 | 1.06E-98 |
| Acot1 | -1.744 | 1.56E-100 | 1.19E-98 |
| Rusc1 | 3.104 | 4.12E-100 | 3.10E-98 |
| Slc38a10 | 1.809 | 2.99E-99 | 2.23E-97 |
| Comt | -1.281 | 3.37E-99 | 2.50E-97 |
| Acaa1b | -1.323 | 5.60E-99 | 4.15E-97 |
| Sucnr1 | -2.243 | 6.22E-98 | 4.59E-96 |
| Cldn1 | -1.033 | 6.19E-97 | 4.52E-95 |
| S1pr1 | -1.079 | 4.28E-96 | 3.11E-94 |
| Coq8a | -1.292 | 9.35E-96 | 6.77E-94 |
| Nxpe2 | -2.727 | 1.19E-95 | 8.54E-94 |
| Sdf2l1 | 1.816 | 2.11E-95 | 1.50E-93 |
| Cyp4a14 | 1.604 | 1.31E-94 | 9.30E-93 |
| Lpin1 | 1.848 | 8.76E-94 | 6.15E-92 |
| Txndc5 | 1.478 | 3.11E-93 | 2.17E-91 |
| Ppp1r15a | 2.081 | 4.14E-93 | 2.88E-91 |
| Otc | -1.113 | 1.13E-92 | 7.81E-91 |
| Prrc1 | 1.577 | 2.51E-92 | 1.73E-90 |
| Leap2 | 2.332 | 5.44E-92 | 3.71E-90 |
| Wfs1 | 2.356 | 1.16E-91 | 7.88E-90 |
| Mlec | 1.244 | 1.72E-91 | 1.16E-89 |
| Krt18 | 1.713 | 1.78E-91 | 1.20E-89 |
| Ces2c | -1.811 | 1.32E-90 | 8.87E-89 |
| Sec22b | 1.472 | 5.48E-90 | 3.66E-88 |
| Marchf5 | 1.393 | 1.51E-89 | 1.00E-87 |
| Sec23b | 1.809 | 1.55E-89 | 1.03E-87 |
| Nbas | 1.982 | 1.81E-89 | 1.20E-87 |
| Hapln4 | 2.539 | 3.66E-88 | 2.41E-86 |
| Slc48a1 | 1.505 | 2.05E-87 | 1.34E-85 |
| Tat | 1.529 | 9.86E-87 | 6.41E-85 |
| Ces3a | -1.103 | 2.02E-86 | 1.30E-84 |
| Tmed9 | 1.790 | 2.50E-86 | 1.61E-84 |
| Srpr | 1.490 | 3.17E-86 | 2.04E-84 |
| Slc25a42 | -1.273 | 4.47E-86 | 2.86E-84 |
| B3galt1 | 2.542 | 5.61E-86 | 3.56E-84 |
| Gmppa | 2.335 | 7.28E-86 | 4.60E-84 |
| Mmadhc | 1.526 | 1.40E-85 | 8.82E-84 |
| Mybbp1a | 2.011 | 3.44E-85 | 2.16E-83 |
| Retreg3 | -1.190 | 1.13E-84 | 6.99E-83 |
| Acot11 | 2.475 | 2.95E-84 | 1.82E-82 |
| Upp2 | -1.059 | 5.20E-84 | 3.20E-82 |
| Rap1gap2 | 4.195 | 2.18E-83 | 1.34E-81 |
| Wipf3 | 2.816 | 2.94E-83 | 1.80E-81 |
| Depp1 | -1.535 | 1.67E-82 | 1.02E-80 |
| Hid1 | 3.194 | 5.66E-82 | 3.41E-80 |
| Trmt61a | 3.035 | 3.30E-81 | 1.98E-79 |
| Ugt2a3 | -1.017 | 6.25E-81 | 3.74E-79 |
| Nop56 | 1.824 | 1.06E-80 | 6.31E-79 |
| Rassf1 | 2.625 | 2.41E-80 | 1.43E-78 |
| Clptm1l | 1.348 | 3.65E-80 | 2.16E-78 |
| Tjp3 | -1.687 | 1.52E-79 | 8.90E-78 |
| Cyp4f14 | -1.271 | 2.67E-79 | 1.56E-77 |
| Ddx56 | 1.974 | 3.05E-79 | 1.77E-77 |
| Agfg2 | -1.297 | 4.62E-79 | 2.66E-77 |
| Alas2 | -1.219 | 1.40E-78 | 8.01E-77 |
| Slc47a1 | -1.157 | 2.15E-78 | 1.22E-76 |
| Dpp3 | 1.633 | 3.01E-78 | 1.71E-76 |
| Col1a1 | 2.418 | 7.37E-78 | 4.16E-76 |
| Psmc2 | 1.246 | 9.26E-78 | 5.21E-76 |
| Hp | 1.755 | 1.57E-77 | 8.81E-76 |
| Rrp12 | 2.153 | 1.86E-76 | 1.03E-74 |
| Stx5a | 1.749 | 3.28E-76 | 1.81E-74 |
| Cyp4a31 | 2.211 | 5.52E-76 | 3.03E-74 |
| Pla2g12a | 2.282 | 1.36E-75 | 7.46E-74 |
| Ppard | -1.968 | 1.82E-75 | 9.90E-74 |
| Hdlbp | 1.139 | 6.44E-75 | 3.47E-73 |
| Car5a | -1.378 | 6.71E-75 | 3.61E-73 |
| Mup8 | -1.413 | 2.56E-74 | 1.37E-72 |
| Ccdc149 | 3.317 | 2.89E-74 | 1.54E-72 |
| Spcs2 | 1.164 | 2.90E-74 | 1.54E-72 |
| Psma7 | 1.312 | 6.09E-74 | 3.22E-72 |
| Hpx | 1.683 | 1.46E-73 | 7.65E-72 |
| Srp68 | 1.398 | 1.53E-73 | 8.01E-72 |
| Osbp | 1.355 | 2.38E-72 | 1.24E-70 |
| Odc1 | 1.334 | 2.73E-72 | 1.42E-70 |
| Nifk | 1.391 | 8.84E-72 | 4.54E-70 |
| Ciapin1 | 1.889 | 1.01E-71 | 5.20E-70 |
| Atf6 | 1.864 | 1.19E-71 | 6.07E-70 |
| Ikbke | 2.116 | 1.29E-71 | 6.56E-70 |
| Ets2 | 1.369 | 1.55E-71 | 7.88E-70 |
| Gadd45a | 3.541 | 1.58E-71 | 7.98E-70 |
| Ftsj3 | 1.613 | 1.59E-71 | 8.00E-70 |
| Hes6 | -1.378 | 2.40E-71 | 1.21E-69 |
| Prg4 | 1.596 | 4.00E-71 | 2.00E-69 |
| Nat8f1 | -1.289 | 6.95E-71 | 3.45E-69 |
| Lss | 1.502 | 2.12E-70 | 1.05E-68 |
| Mafk | 2.056 | 3.18E-70 | 1.57E-68 |
| Slc25a33 | 1.624 | 3.59E-70 | 1.76E-68 |
| Tram1 | 1.200 | 6.14E-70 | 3.01E-68 |
| Por | 1.728 | 2.86E-69 | 1.40E-67 |
| Aldh18a1 | 2.342 | 3.08E-69 | 1.50E-67 |
| 2010003K11Rik | 2.434 | 8.46E-69 | 4.09E-67 |
| Ostc | 1.443 | 1.64E-68 | 7.92E-67 |
| Ddx21 | 1.528 | 1.97E-68 | 9.46E-67 |
| Igsf11 | 1.712 | 8.25E-68 | 3.93E-66 |
| Avpi1 | 1.989 | 1.07E-67 | 5.09E-66 |
| Nr1d2 | 2.504 | 2.20E-67 | 1.04E-65 |
| Slc39a7 | 1.749 | 6.55E-67 | 3.08E-65 |
| Mvp | 1.696 | 2.45E-66 | 1.14E-64 |
| Copb2 | 1.156 | 2.71E-66 | 1.26E-64 |
| Elp3 | 1.559 | 4.10E-66 | 1.89E-64 |
| P4hb | 1.420 | 5.17E-66 | 2.37E-64 |
| Ficd | 1.854 | 1.24E-65 | 5.70E-64 |
| Gramd1c | 2.605 | 1.93E-65 | 8.81E-64 |
| Slc16a5 | 2.435 | 6.87E-65 | 3.12E-63 |
| Nqo2 | -1.161 | 1.94E-64 | 8.77E-63 |
| Gdap2 | 1.427 | 5.67E-64 | 2.55E-62 |
| Atp2a2 | 1.116 | 6.26E-64 | 2.81E-62 |
| Nr4a1 | -2.961 | 1.77E-63 | 7.93E-62 |
| Adgrf1 | -3.779 | 2.06E-63 | 9.19E-62 |
| Cdk2ap2 | 1.616 | 2.27E-63 | 1.01E-61 |
| Cd74 | -1.093 | 2.29E-63 | 1.01E-61 |
| Gm3776 | 2.577 | 5.86E-63 | 2.59E-61 |
| Txnl1 | 1.140 | 1.04E-62 | 4.60E-61 |
| Mrps18b | 1.579 | 1.27E-62 | 5.60E-61 |
| Nqo1 | 2.375 | 1.59E-61 | 6.89E-60 |
| Srp19 | 2.052 | 1.67E-61 | 7.24E-60 |
| Stt3b | 1.152 | 2.55E-61 | 1.09E-59 |
| Cacfd1 | 1.619 | 2.68E-61 | 1.15E-59 |
| Ifrd2 | 1.505 | 3.77E-61 | 1.60E-59 |
| Gpnmb | 5.480 | 3.92E-61 | 1.66E-59 |
| Tsr1 | 1.937 | 8.35E-61 | 3.54E-59 |
| Tppp | -1.708 | 1.09E-60 | 4.59E-59 |
| Syvn1 | 1.399 | 1.63E-60 | 6.80E-59 |
| Srp54c | 3.124 | 4.31E-60 | 1.79E-58 |
| Anxa2 | 1.823 | 4.36E-60 | 1.81E-58 |
| Pmm1 | 2.637 | 8.49E-60 | 3.51E-58 |
| Adrm1 | 1.523 | 1.45E-59 | 5.98E-58 |
| Dynll1 | 2.122 | 2.62E-59 | 1.08E-57 |
| Nrep | -2.236 | 3.80E-59 | 1.55E-57 |
| Srp72 | 1.489 | 4.00E-59 | 1.63E-57 |
| Fibin | 4.646 | 5.67E-59 | 2.31E-57 |
| Nol3 | 4.665 | 7.58E-59 | 3.08E-57 |
| Lmna | 1.674 | 8.18E-59 | 3.30E-57 |
| Ctps | 2.450 | 8.44E-59 | 3.40E-57 |
| Zfp330 | 2.076 | 9.36E-59 | 3.76E-57 |
| Ssr2 | 1.408 | 1.15E-58 | 4.59E-57 |
| Snx10 | 1.992 | 1.47E-58 | 5.86E-57 |
| Lrg1 | 1.818 | 2.29E-58 | 9.09E-57 |
| Cct3 | 1.273 | 2.29E-58 | 9.09E-57 |
| Atad3a | 1.280 | 3.58E-58 | 1.41E-56 |
| Edem2 | 1.486 | 4.67E-58 | 1.83E-56 |
| Mfsd2a | 2.576 | 6.49E-58 | 2.54E-56 |
| Xpot | 1.403 | 1.04E-57 | 4.06E-56 |
| Rpl12 | 1.343 | 1.07E-57 | 4.14E-56 |
| Gnl3 | 1.739 | 1.58E-57 | 6.09E-56 |
| Tspyl2 | 2.287 | 1.74E-57 | 6.70E-56 |
| Rars | 1.494 | 3.33E-57 | 1.28E-55 |
| Slc35b1 | 1.429 | 3.88E-57 | 1.49E-55 |
| Stk40 | 1.308 | 4.08E-57 | 1.56E-55 |
| Col1a2 | 1.581 | 4.34E-57 | 1.65E-55 |
| Gstt1 | -1.032 | 4.39E-57 | 1.67E-55 |
| Mme | -2.719 | 8.47E-57 | 3.21E-55 |
| Ypel5 | 1.344 | 9.12E-57 | 3.45E-55 |
| Ubxn4 | 2.056 | 1.36E-56 | 5.10E-55 |
| Gsta1 | 2.964 | 1.57E-56 | 5.88E-55 |
| Fam149a | -1.021 | 1.58E-56 | 5.92E-55 |
| Rras2 | 1.680 | 3.58E-56 | 1.33E-54 |
| Ltbp4 | -1.254 | 4.05E-56 | 1.50E-54 |
| Gas1 | -1.899 | 5.77E-56 | 2.13E-54 |
| Cyp2c69 | -4.447 | 1.01E-55 | 3.71E-54 |
| Ssr3 | 1.409 | 1.21E-55 | 4.45E-54 |
| Yrdc | 1.771 | 1.43E-55 | 5.25E-54 |
| Nsfl1c | 1.365 | 1.96E-55 | 7.18E-54 |
| Wdr43 | 2.147 | 2.32E-55 | 8.48E-54 |
| Spp2 | -1.081 | 9.74E-55 | 3.52E-53 |
| Slc30a5 | 1.269 | 1.09E-54 | 3.91E-53 |
| Nucb2 | 2.519 | 2.22E-54 | 7.95E-53 |
| Gm45753 | -1.460 | 2.74E-54 | 9.81E-53 |
| Arcn1 | 1.580 | 3.39E-54 | 1.21E-52 |
| Cdcp1 | 2.625 | 6.01E-54 | 2.14E-52 |
| Tfg | 1.309 | 6.06E-54 | 2.16E-52 |
| Actg1 | 1.379 | 1.04E-53 | 3.69E-52 |
| Ltv1 | 1.735 | 1.19E-53 | 4.21E-52 |
| Bfar | 1.601 | 1.30E-53 | 4.59E-52 |
| P3h1 | 1.780 | 1.87E-53 | 6.59E-52 |
| Ankrd33b | -1.110 | 1.92E-53 | 6.75E-52 |
| Uso1 | 1.906 | 2.31E-53 | 8.06E-52 |
| Mat1a | 1.276 | 2.71E-53 | 9.45E-52 |
| Psmd3 | 1.431 | 7.63E-53 | 2.65E-51 |
| Nploc4 | 1.198 | 9.97E-53 | 3.46E-51 |
| Pvr | 2.222 | 1.38E-52 | 4.77E-51 |
| Acacb | -1.008 | 1.55E-52 | 5.30E-51 |
| Lman1 | 1.294 | 2.91E-52 | 9.91E-51 |
| Psmd5 | 1.128 | 4.08E-52 | 1.39E-50 |
| Nnmt | 1.320 | 9.11E-52 | 3.08E-50 |
| Nop58 | 2.777 | 1.17E-51 | 3.93E-50 |
| Vars | 1.666 | 1.34E-51 | 4.50E-50 |
| Abca8a | -1.520 | 2.33E-51 | 7.79E-50 |
| Flt4 | -1.028 | 3.28E-51 | 1.09E-49 |
| Ubiad1 | 2.106 | 5.47E-51 | 1.81E-49 |
| Ddx52 | 2.035 | 5.83E-51 | 1.93E-49 |
| Klkb1 | -1.330 | 1.40E-50 | 4.61E-49 |
| Crls1 | 1.445 | 2.33E-50 | 7.65E-49 |
| Lrrfip2 | 1.452 | 2.85E-50 | 9.36E-49 |
| Chid1 | 1.305 | 3.15E-50 | 1.03E-48 |
| Mgat2 | 1.161 | 3.85E-50 | 1.26E-48 |
| Golga1 | 1.856 | 7.94E-50 | 2.59E-48 |
| Mideas | 1.652 | 8.06E-50 | 2.62E-48 |
| Pomgnt1 | 1.613 | 8.25E-50 | 2.68E-48 |
| Als2cl | 1.414 | 2.65E-49 | 8.55E-48 |
| Trmt6 | 1.931 | 2.77E-49 | 8.93E-48 |
| Pdia4 | 1.220 | 3.70E-49 | 1.19E-47 |
| Naa25 | 1.543 | 4.72E-49 | 1.51E-47 |
| Psmd7 | 1.065 | 5.37E-49 | 1.72E-47 |
| Hmgn2 | 1.796 | 8.41E-49 | 2.67E-47 |
| Srp9 | 1.194 | 1.13E-48 | 3.58E-47 |
| Coq8b | 1.671 | 1.41E-48 | 4.44E-47 |
| Sec31a | 1.126 | 2.92E-48 | 9.18E-47 |
| Tsen2 | 2.293 | 3.48E-48 | 1.09E-46 |
| Plec | 1.307 | 5.67E-48 | 1.77E-46 |
| Paip2b | 1.510 | 6.80E-48 | 2.12E-46 |
| Hspb1 | 1.543 | 8.92E-48 | 2.78E-46 |
| Tyw1 | 1.448 | 1.02E-47 | 3.16E-46 |
| Tbc1d31 | 2.114 | 1.08E-47 | 3.34E-46 |
| Tmem39a | 1.946 | 1.23E-47 | 3.80E-46 |
| Rrbp1 | 1.185 | 1.23E-47 | 3.82E-46 |
| Rpl13a | 1.216 | 2.01E-47 | 6.14E-46 |
| Eif3d | 1.473 | 2.27E-47 | 6.91E-46 |
| Tlcd2 | -1.053 | 3.85E-47 | 1.16E-45 |
| Paqr7 | -1.343 | 4.22E-47 | 1.27E-45 |
| Ncald | -1.762 | 7.81E-47 | 2.35E-45 |
| Psmc5 | 1.119 | 8.28E-47 | 2.49E-45 |
| Crybg1 | 1.419 | 1.15E-46 | 3.43E-45 |
| Arf1 | 1.111 | 1.32E-46 | 3.94E-45 |
| Igfals | -1.053 | 1.38E-46 | 4.11E-45 |
| Rps12 | 1.283 | 1.39E-46 | 4.13E-45 |
| Anp32b | 1.282 | 2.07E-46 | 6.16E-45 |
| Col3a1 | 1.488 | 3.40E-46 | 1.01E-44 |
| Pum3 | 1.822 | 4.27E-46 | 1.26E-44 |
| Tubb6 | 2.120 | 6.48E-46 | 1.91E-44 |
| Fubp1 | 1.559 | 3.34E-45 | 9.71E-44 |
| Arf6 | 1.322 | 3.90E-45 | 1.13E-43 |
| Nomo1 | 1.196 | 5.05E-45 | 1.46E-43 |
| Atf6b | 1.470 | 5.30E-45 | 1.53E-43 |
| Lpin3 | 2.571 | 7.93E-45 | 2.28E-43 |
| Ttc39a | 3.441 | 1.69E-44 | 4.84E-43 |
| Ccnd1 | 1.745 | 3.90E-44 | 1.12E-42 |
| Col15a1 | 1.698 | 4.84E-44 | 1.38E-42 |
| Slc39a4 | -1.025 | 4.91E-44 | 1.40E-42 |
| Tnfrsf12a | 1.911 | 5.27E-44 | 1.50E-42 |
| Orm3 | 5.382 | 5.28E-44 | 1.50E-42 |
| Supt5 | 1.323 | 5.35E-44 | 1.52E-42 |
| Psmd6 | 1.160 | 5.56E-44 | 1.58E-42 |
| Slc7a1 | 2.925 | 1.07E-43 | 3.01E-42 |
| Nabp1 | 2.260 | 2.04E-43 | 5.67E-42 |
| Scfd2 | 2.207 | 2.21E-43 | 6.14E-42 |
| Ide | 1.316 | 2.36E-43 | 6.55E-42 |
| Ibtk | 1.599 | 2.51E-43 | 6.96E-42 |
| Nr0b2 | -1.815 | 5.54E-43 | 1.52E-41 |
| Tmem25 | -1.618 | 5.68E-43 | 1.56E-41 |
| Ptges3 | 1.202 | 6.20E-43 | 1.70E-41 |
| Tfb1m | 2.086 | 9.74E-43 | 2.65E-41 |
| Tspan33 | -1.436 | 1.52E-42 | 4.11E-41 |
| Smim14 | 1.403 | 1.78E-42 | 4.81E-41 |
| Smyd5 | 1.958 | 2.14E-42 | 5.77E-41 |
| Cela1 | -1.859 | 4.17E-42 | 1.12E-40 |
| Ipo4 | 1.704 | 4.39E-42 | 1.18E-40 |
| Hspa13 | 2.124 | 4.77E-42 | 1.28E-40 |
| Epg5 | 1.244 | 4.95E-42 | 1.33E-40 |
| Lgals8 | 1.388 | 5.26E-42 | 1.41E-40 |
| Mrpl17 | 1.434 | 6.73E-42 | 1.80E-40 |
| Serpine2 | -1.282 | 7.81E-42 | 2.08E-40 |
| Trim27 | 1.494 | 1.33E-41 | 3.53E-40 |
| Rad23b | 1.019 | 1.69E-41 | 4.47E-40 |
| Socs2 | -1.446 | 2.63E-41 | 6.93E-40 |
| Dhx40 | 1.668 | 3.41E-41 | 8.93E-40 |
| Rexo2 | 1.140 | 3.83E-41 | 1.00E-39 |
| Smox | 2.294 | 3.96E-41 | 1.03E-39 |
| Zfp395 | -1.154 | 7.11E-41 | 1.85E-39 |
| Sec23a | 1.055 | 9.06E-41 | 2.35E-39 |
| Zfp9 | 2.910 | 1.21E-40 | 3.12E-39 |
| Eef1b2 | 1.044 | 1.57E-40 | 4.03E-39 |
| Surf4 | 1.237 | 1.69E-40 | 4.34E-39 |
| Ufm1 | 1.243 | 2.13E-40 | 5.48E-39 |
| Hars | 1.209 | 2.32E-40 | 5.96E-39 |
| Cyp26b1 | 2.478 | 2.39E-40 | 6.12E-39 |
| Josd1 | 1.193 | 3.01E-40 | 7.71E-39 |
| Sesn2 | 1.689 | 3.20E-40 | 8.17E-39 |
| Itih4 | 1.403 | 3.38E-40 | 8.61E-39 |
| Per1 | 1.680 | 5.30E-40 | 1.35E-38 |
| Acnat2 | 1.121 | 7.72E-40 | 1.95E-38 |
| Fkbp5 | 1.281 | 9.42E-40 | 2.38E-38 |
| Gorasp2 | 1.276 | 9.71E-40 | 2.45E-38 |
| Psma5 | 1.314 | 1.03E-39 | 2.58E-38 |
| Slc35c2 | 1.568 | 1.14E-39 | 2.88E-38 |
| Cep170b | 1.398 | 1.16E-39 | 2.92E-38 |
| Snd1 | 1.204 | 1.36E-39 | 3.41E-38 |
| Tpm2 | -1.859 | 4.28E-39 | 1.07E-37 |
| Kcmf1 | 1.125 | 4.56E-39 | 1.14E-37 |
| Tox | 2.617 | 4.90E-39 | 1.22E-37 |
| Nop2 | 1.466 | 5.58E-39 | 1.38E-37 |
| Soat2 | 1.540 | 9.31E-39 | 2.31E-37 |
| Plekhb1 | -1.232 | 1.09E-38 | 2.68E-37 |
| Ngdn | 1.408 | 1.17E-38 | 2.89E-37 |
| Mthfd1l | 3.392 | 2.28E-38 | 5.59E-37 |
| Ufd1 | 1.405 | 2.58E-38 | 6.31E-37 |
| Atg13 | 1.420 | 2.68E-38 | 6.55E-37 |
| Ajuba | 1.754 | 2.92E-38 | 7.11E-37 |
| Pdp2 | -1.006 | 3.55E-38 | 8.62E-37 |
| Hspb8 | 1.162 | 4.95E-38 | 1.20E-36 |
| Vkorc1l1 | 1.216 | 5.43E-38 | 1.31E-36 |
| Chac1 | 3.290 | 5.99E-38 | 1.44E-36 |
| Spata5 | 1.968 | 6.62E-38 | 1.59E-36 |
| Fam210a | -1.066 | 7.03E-38 | 1.69E-36 |
| Herpud1 | 1.239 | 9.12E-38 | 2.18E-36 |
| Srsf3 | 1.281 | 9.52E-38 | 2.27E-36 |
| Dusp1 | -1.124 | 1.15E-37 | 2.73E-36 |
| Cep85 | 1.596 | 1.83E-37 | 4.34E-36 |
| Tbc1d16 | 1.553 | 2.01E-37 | 4.75E-36 |
| Heatr1 | 1.319 | 3.36E-37 | 7.92E-36 |
| Psmc3 | 1.161 | 3.74E-37 | 8.80E-36 |
| Golga5 | 1.339 | 4.31E-37 | 1.01E-35 |
| Srp14 | 1.314 | 4.53E-37 | 1.06E-35 |
| Ociad2 | -1.017 | 6.61E-37 | 1.54E-35 |
| Spcs3 | 1.509 | 8.09E-37 | 1.88E-35 |
| Cela2a | -5.071 | 1.04E-36 | 2.41E-35 |
| Mtor | 1.172 | 1.12E-36 | 2.59E-35 |
| Rarres1 | 1.253 | 1.22E-36 | 2.83E-35 |
| Polr3d | 2.060 | 1.25E-36 | 2.87E-35 |
| Fbxw9 | 1.773 | 1.37E-36 | 3.16E-35 |
| Cyp4a10 | 1.202 | 1.53E-36 | 3.52E-35 |
| Raet1d | -1.559 | 1.71E-36 | 3.92E-35 |
| Eprs | 1.603 | 1.82E-36 | 4.16E-35 |
| Wdr3 | 1.548 | 2.53E-36 | 5.76E-35 |
| Gpatch4 | 1.914 | 3.09E-36 | 7.03E-35 |
| Mettl1 | 2.216 | 3.38E-36 | 7.67E-35 |
| Mogs | 1.431 | 3.77E-36 | 8.54E-35 |
| Adck5 | -1.058 | 4.30E-36 | 9.69E-35 |
| Gnat1 | 3.699 | 5.08E-36 | 1.14E-34 |
| Rpl3 | 1.006 | 5.48E-36 | 1.23E-34 |
| Slco1a1 | -2.613 | 5.85E-36 | 1.31E-34 |
| Gjb2 | 1.067 | 6.30E-36 | 1.41E-34 |
| Fam114a1 | 1.520 | 6.64E-36 | 1.49E-34 |
| H2-Ab1 | -1.123 | 7.81E-36 | 1.75E-34 |
| Gdf15 | 2.382 | 1.21E-35 | 2.70E-34 |
| Rhbdd1 | 1.523 | 1.32E-35 | 2.93E-34 |
| Pnrc2 | 1.389 | 1.35E-35 | 3.00E-34 |
| Ufl1 | 1.830 | 1.46E-35 | 3.24E-34 |
| C3 | 1.233 | 1.49E-35 | 3.31E-34 |
| Bmf | -1.552 | 2.56E-35 | 5.68E-34 |
| Eif4ebp1 | 1.447 | 3.10E-35 | 6.85E-34 |
| Golga3 | 1.216 | 3.17E-35 | 6.98E-34 |
| Ddost | 1.201 | 3.58E-35 | 7.88E-34 |
| Ube2o | 1.494 | 4.37E-35 | 9.58E-34 |
| Atoh8 | 1.459 | 5.38E-35 | 1.18E-33 |
| Aatf | 1.693 | 5.63E-35 | 1.23E-33 |
| Slc41a3 | 3.183 | 5.81E-35 | 1.26E-33 |
| Npas2 | -2.358 | 5.89E-35 | 1.28E-33 |
| Rheb | 1.237 | 8.88E-35 | 1.92E-33 |
| Psmd14 | 1.071 | 9.07E-35 | 1.95E-33 |
| Fam118a | 3.439 | 1.63E-34 | 3.48E-33 |
| Alg12 | 1.921 | 1.74E-34 | 3.71E-33 |
| Stx18 | 1.291 | 1.91E-34 | 4.08E-33 |
| Shank2 | -1.069 | 1.95E-34 | 4.15E-33 |
| Slc23a1 | 1.297 | 2.47E-34 | 5.25E-33 |
| Sars | 1.143 | 2.58E-34 | 5.47E-33 |
| Ttc27 | 1.498 | 2.71E-34 | 5.75E-33 |
| Cxcl9 | -1.468 | 2.76E-34 | 5.84E-33 |
| Cdv3 | 1.116 | 4.15E-34 | 8.76E-33 |
| Hsp90b1 | 1.931 | 4.51E-34 | 9.50E-33 |
| Rit1 | 1.767 | 4.99E-34 | 1.05E-32 |
| Ube2j1 | 1.081 | 5.36E-34 | 1.12E-32 |
| Pes1 | 1.302 | 5.86E-34 | 1.23E-32 |
| Psmd11 | 1.019 | 6.83E-34 | 1.43E-32 |
| Bet1 | 1.318 | 7.11E-34 | 1.48E-32 |
| Eif6 | 1.558 | 1.09E-33 | 2.26E-32 |
| Nup210 | 1.566 | 1.71E-33 | 3.53E-32 |
| Pck2 | 2.167 | 1.79E-33 | 3.69E-32 |
| Nr2c2ap | 1.743 | 2.54E-33 | 5.23E-32 |
| Slc38a2 | 1.003 | 2.71E-33 | 5.56E-32 |
| Srrm4 | 3.104 | 3.51E-33 | 7.16E-32 |
| Rgs3 | -1.461 | 3.72E-33 | 7.58E-32 |
| Btg3 | 1.557 | 3.81E-33 | 7.76E-32 |
| Tymp | -1.147 | 4.42E-33 | 8.99E-32 |
| Tceal8 | 1.524 | 5.19E-33 | 1.05E-31 |
| Tmem248 | 1.207 | 5.59E-33 | 1.13E-31 |
| Gask1a | -2.623 | 6.03E-33 | 1.22E-31 |
| Camk1d | -1.020 | 6.04E-33 | 1.22E-31 |
| Pa2g4 | 1.005 | 6.34E-33 | 1.28E-31 |
| Slc34a2 | -2.762 | 6.42E-33 | 1.29E-31 |
| H2-Eb1 | -1.125 | 7.47E-33 | 1.49E-31 |
| Rwdd4a | 1.559 | 9.09E-33 | 1.81E-31 |
| Tcea1 | 1.499 | 1.05E-32 | 2.08E-31 |
| Plk2 | -1.485 | 1.16E-32 | 2.30E-31 |
| Tnfaip1 | 1.035 | 1.59E-32 | 3.15E-31 |
| Slc16a6 | 2.099 | 1.74E-32 | 3.45E-31 |
| Scfd1 | 1.904 | 2.06E-32 | 4.07E-31 |
| Treh | 3.295 | 2.42E-32 | 4.76E-31 |
| Wdr75 | 1.352 | 3.01E-32 | 5.91E-31 |
| Impact | 1.627 | 3.79E-32 | 7.43E-31 |
| Ipo5 | 1.081 | 3.86E-32 | 7.56E-31 |
| Grwd1 | 1.595 | 4.90E-32 | 9.57E-31 |
| Srgap3 | -2.354 | 5.35E-32 | 1.04E-30 |
| Nans | 1.776 | 5.65E-32 | 1.10E-30 |
| H3c14 | 1.570 | 6.10E-32 | 1.18E-30 |
| Trim46 | 5.374 | 7.00E-32 | 1.35E-30 |
| Zfp568 | 1.278 | 7.59E-32 | 1.47E-30 |
| Gne | 1.211 | 8.74E-32 | 1.68E-30 |
| Mettl7b | 1.227 | 9.38E-32 | 1.80E-30 |
| Fitm1 | -1.563 | 9.78E-32 | 1.88E-30 |
| Parn | 1.479 | 1.33E-31 | 2.55E-30 |
| Faf1 | 1.224 | 1.40E-31 | 2.67E-30 |
| Gnat2 | 3.086 | 1.55E-31 | 2.95E-30 |
| Cfd | -4.446 | 2.06E-31 | 3.90E-30 |
| Slc6a12 | 1.098 | 2.12E-31 | 4.01E-30 |
| Cdk18 | 1.247 | 2.33E-31 | 4.40E-30 |
| Nfxl1 | 1.763 | 3.42E-31 | 6.42E-30 |
| Lrit1 | -1.832 | 3.71E-31 | 6.96E-30 |
| Rplp2 | 1.311 | 5.31E-31 | 9.96E-30 |
| Ccdc86 | 1.598 | 5.72E-31 | 1.07E-29 |
| Rab3d | 1.642 | 5.84E-31 | 1.09E-29 |
| Kars | 1.180 | 6.16E-31 | 1.15E-29 |
| Sgk2 | 1.137 | 9.25E-31 | 1.72E-29 |
| Rplp0 | 1.105 | 1.08E-30 | 2.00E-29 |
| Llph | 1.289 | 1.10E-30 | 2.05E-29 |
| C2cd2l | 1.122 | 1.18E-30 | 2.18E-29 |
| Nelfe | 1.321 | 1.19E-30 | 2.21E-29 |
| Eif3g | 1.247 | 1.20E-30 | 2.21E-29 |
| Dnajc10 | 1.416 | 1.74E-30 | 3.19E-29 |
| Col27a1 | -1.383 | 1.79E-30 | 3.29E-29 |
| Marco | 1.418 | 2.09E-30 | 3.82E-29 |
| Ccdc134 | 2.212 | 2.81E-30 | 5.13E-29 |
| Mapk15 | -2.032 | 2.83E-30 | 5.17E-29 |
| Cited2 | -1.193 | 2.83E-30 | 5.17E-29 |
| Stard3nl | 1.832 | 2.96E-30 | 5.40E-29 |
| Cog6 | 1.177 | 3.10E-30 | 5.64E-29 |
| Ikbkg | 1.040 | 3.32E-30 | 6.02E-29 |
| Tubb4b | 1.190 | 3.65E-30 | 6.60E-29 |
| Ak6 | 1.099 | 3.76E-30 | 6.78E-29 |
| Psmc6 | 1.603 | 3.86E-30 | 6.95E-29 |
| Slc17a8 | -1.538 | 4.31E-30 | 7.75E-29 |
| Dpagt1 | 1.361 | 5.39E-30 | 9.68E-29 |
| Rpl14 | 1.013 | 5.71E-30 | 1.02E-28 |
| Rab3gap2 | 1.304 | 6.89E-30 | 1.23E-28 |
| Shc1 | 1.142 | 1.11E-29 | 1.98E-28 |
| Esr1 | 1.501 | 1.14E-29 | 2.03E-28 |
| Ica1 | 2.764 | 1.19E-29 | 2.12E-28 |
| Prmt3 | 1.397 | 1.37E-29 | 2.44E-28 |
| Tmem167b | 1.058 | 1.56E-29 | 2.76E-28 |
| Acta1 | -13.942 | 1.63E-29 | 2.89E-28 |
| Pex2 | 1.239 | 2.23E-29 | 3.93E-28 |
| Cgref1 | 5.173 | 2.61E-29 | 4.59E-28 |
| H1f2 | 1.065 | 3.01E-29 | 5.29E-28 |
| Maoa | 1.436 | 3.22E-29 | 5.64E-28 |
| Copg1 | 1.214 | 3.27E-29 | 5.72E-28 |
| Mylpf | -4.047 | 3.33E-29 | 5.80E-28 |
| G3bp1 | 1.003 | 4.72E-29 | 8.22E-28 |
| Utp20 | 1.531 | 5.22E-29 | 9.09E-28 |
| Eif2b3 | 1.716 | 5.41E-29 | 9.40E-28 |
| Cyp7a1 | 1.707 | 5.86E-29 | 1.02E-27 |
| Wdr36 | 1.243 | 7.36E-29 | 1.28E-27 |
| Pgd | 1.275 | 7.60E-29 | 1.31E-27 |
| Sdad1 | 1.705 | 8.73E-29 | 1.51E-27 |
| Ncl | 1.680 | 1.03E-28 | 1.78E-27 |
| Ugt2b37 | -3.479 | 1.05E-28 | 1.82E-27 |
| Blzf1 | 1.709 | 1.06E-28 | 1.82E-27 |
| Bco2 | -1.002 | 1.19E-28 | 2.04E-27 |
| Cyb561 | 1.730 | 1.24E-28 | 2.13E-27 |
| Rsl1d1 | 1.423 | 1.25E-28 | 2.13E-27 |
| Slc9a8 | 1.086 | 1.27E-28 | 2.18E-27 |
| Cyp21a1 | -3.732 | 1.59E-28 | 2.71E-27 |
| Copb1 | 1.288 | 1.68E-28 | 2.86E-27 |
| Myh14 | 1.427 | 2.08E-28 | 3.53E-27 |
| Kctd9 | 1.647 | 2.10E-28 | 3.55E-27 |
| Gale | 1.575 | 2.50E-28 | 4.23E-27 |
| Ddx49 | 1.356 | 2.66E-28 | 4.49E-27 |
| Ddx27 | 1.145 | 2.66E-28 | 4.49E-27 |
| Ubfd1 | 1.035 | 2.96E-28 | 4.98E-27 |
| Rab33b | 1.657 | 2.97E-28 | 4.99E-27 |
| Calu | 1.003 | 3.79E-28 | 6.35E-27 |
| Tmpo | 1.099 | 3.80E-28 | 6.36E-27 |
| Tes | 2.345 | 4.82E-28 | 8.02E-27 |
| Aldoa | 1.281 | 6.10E-28 | 1.01E-26 |
| Ppp2r3c | 1.666 | 6.25E-28 | 1.04E-26 |
| Ccdc120 | 3.208 | 7.11E-28 | 1.18E-26 |
| Wdr74 | 1.401 | 7.44E-28 | 1.23E-26 |
| Psmc4 | 1.143 | 7.55E-28 | 1.25E-26 |
| C77080 | 1.115 | 8.32E-28 | 1.38E-26 |
| Yipf2 | 1.345 | 8.85E-28 | 1.46E-26 |
| Ppan | 2.002 | 9.30E-28 | 1.53E-26 |
| Ptrh2 | 1.427 | 1.17E-27 | 1.92E-26 |
| Plcl2 | 1.459 | 1.27E-27 | 2.08E-26 |
| Spg21 | 1.378 | 1.37E-27 | 2.24E-26 |
| Pir | 1.182 | 2.08E-27 | 3.39E-26 |
| Tapbpl | 1.239 | 2.40E-27 | 3.90E-26 |
| Cog3 | 1.289 | 2.57E-27 | 4.17E-26 |
| Kctd15 | 1.840 | 2.72E-27 | 4.41E-26 |
| Ebna1bp2 | 1.377 | 2.91E-27 | 4.71E-26 |
| Ssr4 | 1.137 | 3.53E-27 | 5.69E-26 |
| Pard3b | 1.730 | 3.54E-27 | 5.70E-26 |
| Wwtr1 | 1.393 | 4.22E-27 | 6.79E-26 |
| Sf3b3 | 1.084 | 4.39E-27 | 7.04E-26 |
| Ccl6 | 1.306 | 5.01E-27 | 8.01E-26 |
| Yif1a | 1.316 | 5.13E-27 | 8.20E-26 |
| Vegfb | -1.285 | 5.94E-27 | 9.48E-26 |
| Srp54a | 2.792 | 6.28E-27 | 1.00E-25 |
| Slc13a2 | -3.796 | 6.89E-27 | 1.10E-25 |
| Mphosph10 | 2.032 | 7.00E-27 | 1.11E-25 |
| Alg2 | 1.211 | 7.56E-27 | 1.20E-25 |
| Lgr5 | -1.442 | 1.22E-26 | 1.93E-25 |
| Plaa | 1.607 | 1.39E-26 | 2.20E-25 |
| Psmd4 | 1.067 | 1.51E-26 | 2.37E-25 |
| Eif3b | 1.034 | 1.63E-26 | 2.56E-25 |
| Reps1 | 1.258 | 2.17E-26 | 3.40E-25 |
| Ube4b | 1.146 | 2.39E-26 | 3.73E-25 |
| Osbpl2 | 1.016 | 2.93E-26 | 4.56E-25 |
| Ero1a | 1.356 | 3.16E-26 | 4.92E-25 |
| Tuba1c | 1.088 | 4.08E-26 | 6.33E-25 |
| Apba3 | 1.058 | 4.14E-26 | 6.42E-25 |
| Rrp9 | 1.667 | 4.56E-26 | 7.06E-25 |
| Ampd2 | 1.160 | 4.76E-26 | 7.37E-25 |
| Fmo4 | 1.688 | 4.78E-26 | 7.39E-25 |
| Cilk1 | 1.310 | 5.27E-26 | 8.12E-25 |
| Zbtb21 | 1.667 | 5.39E-26 | 8.30E-25 |
| Cyp4a32 | 1.280 | 5.43E-26 | 8.35E-25 |
| Nudcd1 | 1.542 | 9.01E-26 | 1.38E-24 |
| Stat5a | -1.135 | 9.85E-26 | 1.50E-24 |
| Pwp1 | 1.220 | 1.02E-25 | 1.55E-24 |
| Rrp8 | 1.203 | 1.29E-25 | 1.96E-24 |
| Usp20 | 1.453 | 1.31E-25 | 1.99E-24 |
| Atp2a1 | -11.861 | 1.38E-25 | 2.09E-24 |
| Pus7 | 1.460 | 1.47E-25 | 2.22E-24 |
| Hcfc2 | 1.416 | 1.54E-25 | 2.32E-24 |
| Psmb4 | 1.070 | 1.63E-25 | 2.45E-24 |
| Utp4 | 1.235 | 1.63E-25 | 2.45E-24 |
| Trmt1 | 1.195 | 1.68E-25 | 2.52E-24 |
| Usp36 | 1.250 | 1.68E-25 | 2.52E-24 |
| Nuak2 | -1.148 | 1.76E-25 | 2.63E-24 |
| Dpy19l3 | -1.186 | 1.82E-25 | 2.73E-24 |
| Ppp1r14b | 1.500 | 1.99E-25 | 2.99E-24 |
| Fzd8 | -1.119 | 2.07E-25 | 3.10E-24 |
| Pnlip | -13.762 | 2.19E-25 | 3.28E-24 |
| Moxd1 | 4.581 | 2.50E-25 | 3.73E-24 |
| Cdk5rap3 | 1.230 | 2.59E-25 | 3.87E-24 |
| Cltb | 1.099 | 2.68E-25 | 4.00E-24 |
| Sys1 | 1.254 | 2.68E-25 | 4.00E-24 |
| Arsg | 1.486 | 3.03E-25 | 4.51E-24 |
| Pex11g | 1.202 | 3.33E-25 | 4.95E-24 |
| Icam1 | 1.253 | 3.69E-25 | 5.46E-24 |
| Pcp4l1 | -1.006 | 3.78E-25 | 5.60E-24 |
| Nipal1 | 2.929 | 4.39E-25 | 6.48E-24 |
| Pwp2 | 1.282 | 5.44E-25 | 7.99E-24 |
| Mthfd2 | 2.487 | 5.66E-25 | 8.31E-24 |
| Rrm2 | 1.929 | 6.74E-25 | 9.86E-24 |
| Gpr89 | 1.119 | 7.77E-25 | 1.13E-23 |
| Bzw2 | 1.615 | 7.77E-25 | 1.13E-23 |
| Eif2b4 | 1.200 | 8.23E-25 | 1.20E-23 |
| Dexi | -1.165 | 9.47E-25 | 1.38E-23 |
| Syt12 | 2.986 | 9.96E-25 | 1.45E-23 |
| Mrpl50 | 1.125 | 1.12E-24 | 1.63E-23 |
| Rpsa | 1.011 | 1.29E-24 | 1.87E-23 |
| Cnppd1 | 1.255 | 1.43E-24 | 2.06E-23 |
| Jpt2 | 1.050 | 1.50E-24 | 2.16E-23 |
| Rxrb | 1.207 | 1.57E-24 | 2.27E-23 |
| D630039A03Rik | -1.244 | 1.70E-24 | 2.44E-23 |
| Aimp1 | 1.227 | 1.75E-24 | 2.51E-23 |
| Cpeb1 | 2.792 | 1.92E-24 | 2.76E-23 |
| Cks1b | 1.946 | 2.36E-24 | 3.38E-23 |
| Acsl4 | 1.629 | 2.45E-24 | 3.49E-23 |
| Tmem120a | 1.119 | 2.54E-24 | 3.62E-23 |
| Atp1a2 | -2.428 | 2.59E-24 | 3.69E-23 |
| Mb | -13.226 | 2.59E-24 | 3.70E-23 |
| Urb2 | 1.373 | 2.70E-24 | 3.84E-23 |
| Sqle | 1.022 | 2.82E-24 | 4.01E-23 |
| Tkfc | 1.191 | 3.15E-24 | 4.47E-23 |
| Rplp1 | 1.009 | 3.45E-24 | 4.89E-23 |
| Mak16 | 1.556 | 3.46E-24 | 4.90E-23 |
| Mmrn2 | -1.080 | 5.45E-24 | 7.69E-23 |
| Slc37a4 | 1.021 | 5.68E-24 | 8.00E-23 |
| Bysl | 1.284 | 5.91E-24 | 8.32E-23 |
| Prmt7 | 1.598 | 6.51E-24 | 9.15E-23 |
| Impdh1 | 1.948 | 6.77E-24 | 9.51E-23 |
| Mab21l3 | 3.005 | 7.27E-24 | 1.02E-22 |
| Lrrc8d | 1.037 | 7.67E-24 | 1.08E-22 |
| Ppl | 1.507 | 8.04E-24 | 1.12E-22 |
| Psmb5 | 1.179 | 9.35E-24 | 1.31E-22 |
| Farsb | 1.153 | 9.38E-24 | 1.31E-22 |
| Vldlr | 2.550 | 9.42E-24 | 1.31E-22 |
| Pycr2 | 2.050 | 9.67E-24 | 1.35E-22 |
| Cope | 1.120 | 9.78E-24 | 1.36E-22 |
| Ints3 | 1.005 | 1.01E-23 | 1.40E-22 |
| Plekha5 | 1.211 | 1.03E-23 | 1.43E-22 |
| Nudcd2 | 1.264 | 1.23E-23 | 1.70E-22 |
| Slc39a11 | 1.180 | 1.28E-23 | 1.76E-22 |
| Arl14ep | 2.023 | 1.32E-23 | 1.82E-22 |
| Ckm | -12.098 | 1.39E-23 | 1.91E-22 |
| Rrs1 | 1.182 | 1.47E-23 | 2.02E-22 |
| Sec61b | 1.069 | 1.54E-23 | 2.12E-22 |
| Tex30 | 2.003 | 1.75E-23 | 2.41E-22 |
| Chmp2b | 1.233 | 1.90E-23 | 2.61E-22 |
| Ell2 | 1.335 | 2.00E-23 | 2.75E-22 |
| Timm21 | 1.308 | 2.08E-23 | 2.84E-22 |
| Tdg | 1.013 | 2.26E-23 | 3.09E-22 |
| Paqr3 | 1.695 | 2.62E-23 | 3.58E-22 |
| Btc | 3.170 | 2.73E-23 | 3.73E-22 |
| Srsf1 | 1.175 | 3.15E-23 | 4.28E-22 |
| Samd4 | 1.738 | 3.19E-23 | 4.33E-22 |
| Ube2k | 1.148 | 3.20E-23 | 4.35E-22 |
| Tmem209 | 1.404 | 3.28E-23 | 4.45E-22 |
| Ube2g2 | 1.237 | 3.45E-23 | 4.67E-22 |
| Tmem167 | 1.320 | 3.51E-23 | 4.75E-22 |
| Mdm2 | 1.207 | 4.62E-23 | 6.22E-22 |
| Lhfpl2 | 1.210 | 4.79E-23 | 6.45E-22 |
| Smo | 1.609 | 5.22E-23 | 7.01E-22 |
| Aen | 1.316 | 5.59E-23 | 7.50E-22 |
| AW209491 | 1.159 | 6.10E-23 | 8.17E-22 |
| Zc3h7a | 1.310 | 6.26E-23 | 8.37E-22 |
| Jun | 1.375 | 6.34E-23 | 8.47E-22 |
| Map7 | 1.108 | 6.66E-23 | 8.88E-22 |
| Tgtp2 | -1.617 | 6.86E-23 | 9.13E-22 |
| Nrg4 | 2.428 | 7.01E-23 | 9.32E-22 |
| Abcf3 | 1.006 | 8.94E-23 | 1.18E-21 |
| Srsf7 | 1.324 | 9.13E-23 | 1.21E-21 |
| Thumpd3 | 1.587 | 9.41E-23 | 1.24E-21 |
| Morc4 | 2.759 | 1.10E-22 | 1.45E-21 |
| Rps11 | 1.010 | 1.16E-22 | 1.53E-21 |
| Mcrip1 | 1.467 | 1.39E-22 | 1.82E-21 |
| Trmt2a | 1.266 | 1.47E-22 | 1.92E-21 |
| Cpa1 | -12.966 | 1.53E-22 | 2.00E-21 |
| Ung | 2.073 | 1.65E-22 | 2.15E-21 |
| Niban1 | 1.493 | 1.66E-22 | 2.16E-21 |
| Ppp1r1b | -1.955 | 1.83E-22 | 2.37E-21 |
| Mtm1 | 1.932 | 1.95E-22 | 2.53E-21 |
| Star | -2.560 | 2.21E-22 | 2.85E-21 |
| Tbc1d15 | 1.577 | 2.28E-22 | 2.94E-21 |
| Fam169b | 1.189 | 2.28E-22 | 2.95E-21 |
| Tssc4 | 1.735 | 2.53E-22 | 3.26E-21 |
| Nt5c2 | 1.284 | 2.56E-22 | 3.29E-21 |
| Shroom3 | 1.217 | 2.57E-22 | 3.30E-21 |
| Get4 | 1.510 | 2.71E-22 | 3.47E-21 |
| Jagn1 | 1.065 | 2.72E-22 | 3.49E-21 |
| Aldh1l2 | 4.175 | 2.92E-22 | 3.74E-21 |
| Rae1 | 1.107 | 3.22E-22 | 4.12E-21 |
| Mast2 | 1.135 | 3.23E-22 | 4.13E-21 |
| Tk1 | -1.092 | 3.48E-22 | 4.44E-21 |
| Fyb2 | 1.796 | 3.72E-22 | 4.72E-21 |
| Pprc1 | 1.190 | 3.87E-22 | 4.91E-21 |
| Adsl | 1.329 | 4.26E-22 | 5.39E-21 |
| Chpf | 1.865 | 5.41E-22 | 6.84E-21 |
| Tbrg4 | 1.078 | 5.51E-22 | 6.97E-21 |
| Polr3e | 1.403 | 5.53E-22 | 6.98E-21 |
| Tlr12 | -1.279 | 6.04E-22 | 7.62E-21 |
| Pdrg1 | 1.213 | 6.16E-22 | 7.75E-21 |
| Cyp17a1 | 1.971 | 6.42E-22 | 8.06E-21 |
| Rcc2 | 1.299 | 7.21E-22 | 9.04E-21 |
| Kdm6a | 1.254 | 7.67E-22 | 9.60E-21 |
| Smg5 | 1.054 | 8.39E-22 | 1.05E-20 |
| Golim4 | 1.587 | 8.70E-22 | 1.09E-20 |
| Tbpl1 | 1.661 | 9.09E-22 | 1.13E-20 |
| Mup13 | -2.699 | 1.00E-21 | 1.25E-20 |
| Eepd1 | 1.302 | 1.03E-21 | 1.27E-20 |
| Acbd3 | 1.325 | 1.06E-21 | 1.32E-20 |
| Pdilt | -1.629 | 1.08E-21 | 1.34E-20 |
| Asph | 1.136 | 1.10E-21 | 1.37E-20 |
| Eif2s1 | 1.161 | 1.13E-21 | 1.40E-20 |
| Ric8a | 1.345 | 1.14E-21 | 1.40E-20 |
| Noc2l | 1.144 | 1.31E-21 | 1.62E-20 |
| Pygm | -2.559 | 1.36E-21 | 1.68E-20 |
| Cdk7 | 1.299 | 1.43E-21 | 1.75E-20 |
| Eif1a | 1.308 | 1.76E-21 | 2.16E-20 |
| Mknk2 | 1.059 | 2.22E-21 | 2.72E-20 |
| Rapgef2 | 1.044 | 2.74E-21 | 3.34E-20 |
| Krtcap2 | 1.050 | 2.90E-21 | 3.53E-20 |
| Pgm2 | 1.560 | 2.91E-21 | 3.54E-20 |
| Amy2a1 | -14.459 | 3.27E-21 | 3.96E-20 |
| Fam83f | 3.109 | 4.03E-21 | 4.85E-20 |
| Mlkl | 1.926 | 4.21E-21 | 5.06E-20 |
| Polr1f | 1.879 | 4.98E-21 | 5.96E-20 |
| Cyp26a1 | 1.012 | 5.05E-21 | 6.05E-20 |
| Lrrfip1 | 1.168 | 5.37E-21 | 6.42E-20 |
| Ss18l2 | 1.331 | 5.85E-21 | 6.99E-20 |
| Pitx3 | -12.090 | 8.45E-21 | 1.01E-19 |
| Diablo | 1.101 | 9.06E-21 | 1.08E-19 |
| Samd4b | 1.025 | 9.24E-21 | 1.10E-19 |
| Olig1 | -2.904 | 9.52E-21 | 1.13E-19 |
| Myh1 | -9.570 | 1.01E-20 | 1.19E-19 |
| Cog8 | 1.122 | 1.05E-20 | 1.24E-19 |
| Exph5 | -1.280 | 1.07E-20 | 1.27E-19 |
| Psmb2 | 1.101 | 1.13E-20 | 1.33E-19 |
| Qtrt1 | 2.532 | 1.34E-20 | 1.57E-19 |
| Tmem11 | 1.075 | 1.38E-20 | 1.62E-19 |
| Arhgap24 | -1.170 | 1.70E-20 | 1.99E-19 |
| Try4 | -14.239 | 1.73E-20 | 2.02E-19 |
| Ddx18 | 1.149 | 1.76E-20 | 2.06E-19 |
| Parp2 | 1.772 | 1.79E-20 | 2.09E-19 |
| Cel | -12.535 | 1.81E-20 | 2.11E-19 |
| Rpl22l1 | 1.395 | 1.86E-20 | 2.17E-19 |
| Eif3j2 | 1.653 | 1.94E-20 | 2.26E-19 |
| Ap1m1 | 1.134 | 2.20E-20 | 2.56E-19 |
| Slc7a5 | 1.796 | 2.39E-20 | 2.78E-19 |
| Eif3j1 | 1.576 | 2.43E-20 | 2.82E-19 |
| Cd63 | 1.586 | 2.81E-20 | 3.25E-19 |
| Cdc34 | 1.102 | 2.89E-20 | 3.34E-19 |
| Atic | 1.069 | 2.98E-20 | 3.44E-19 |
| Notum | -1.019 | 3.34E-20 | 3.86E-19 |
| Myh2 | -9.857 | 3.36E-20 | 3.88E-19 |
| Cdc42se1 | 1.254 | 3.43E-20 | 3.95E-19 |
| Ccdc47 | 1.417 | 3.64E-20 | 4.19E-19 |
| Sult2a8 | -1.848 | 3.80E-20 | 4.37E-19 |
| Grk5 | -1.148 | 3.82E-20 | 4.38E-19 |
| Crym | 3.837 | 4.19E-20 | 4.81E-19 |
| Rps5 | 1.047 | 4.87E-20 | 5.56E-19 |
| Hmgcs1 | 1.095 | 5.04E-20 | 5.75E-19 |
| Nip7 | 1.141 | 5.44E-20 | 6.20E-19 |
| Edem3 | 1.400 | 6.25E-20 | 7.11E-19 |
| Mbnl2 | 1.586 | 6.90E-20 | 7.82E-19 |
| Anapc16 | 1.070 | 8.80E-20 | 9.94E-19 |
| Lemd2 | 1.133 | 1.06E-19 | 1.19E-18 |
| Vmn2r3 | 10.560 | 1.17E-19 | 1.31E-18 |
| Herpud2 | 1.063 | 1.20E-19 | 1.34E-18 |
| Riok2 | 1.173 | 1.21E-19 | 1.35E-18 |
| Aimp2 | 1.499 | 1.24E-19 | 1.39E-18 |
| Mcm6 | 1.627 | 1.38E-19 | 1.54E-18 |
| Nmd3 | 1.052 | 1.81E-19 | 2.01E-18 |
| Polr1b | 1.327 | 2.15E-19 | 2.38E-18 |
| Wdr46 | 1.231 | 2.19E-19 | 2.42E-18 |
| Nol6 | 1.239 | 2.55E-19 | 2.82E-18 |
| Smpd3 | 2.260 | 2.75E-19 | 3.03E-18 |
| 9130409I23Rik | 1.461 | 2.83E-19 | 3.12E-18 |
| Kbtbd12 | 3.379 | 3.35E-19 | 3.68E-18 |
| Umps | 1.168 | 3.49E-19 | 3.84E-18 |
| Lrrc28 | 1.134 | 3.51E-19 | 3.85E-18 |
| 2210010C04Rik | -12.224 | 3.86E-19 | 4.23E-18 |
| Golgb1 | 1.369 | 3.86E-19 | 4.23E-18 |
| Zfp280c | 2.124 | 5.08E-19 | 5.52E-18 |
| Elovl6 | 1.469 | 5.11E-19 | 5.55E-18 |
| Chchd4 | 1.332 | 6.39E-19 | 6.91E-18 |
| Alpl | 1.318 | 6.42E-19 | 6.94E-18 |
| Suco | 1.425 | 6.68E-19 | 7.21E-18 |
| C2cd5 | 1.731 | 6.78E-19 | 7.31E-18 |
| Slc30a7 | 1.216 | 8.51E-19 | 9.15E-18 |
| Tvp23b | 1.148 | 8.76E-19 | 9.42E-18 |
| Try5 | -14.136 | 1.10E-18 | 1.18E-17 |
| Fastk | 1.144 | 1.11E-18 | 1.19E-17 |
| Riok3 | 1.089 | 1.15E-18 | 1.23E-17 |
| Uhrf1 | 3.602 | 1.37E-18 | 1.46E-17 |
| Fads3 | 1.693 | 1.40E-18 | 1.50E-17 |
| Cebpb | 1.215 | 1.45E-18 | 1.55E-17 |
| Gm16286 | 1.055 | 1.49E-18 | 1.59E-17 |
| Prss2 | -13.538 | 1.51E-18 | 1.61E-17 |
| Mcm5 | 2.245 | 1.68E-18 | 1.78E-17 |
| Id1 | 1.343 | 1.70E-18 | 1.80E-17 |
| Npm1 | 1.470 | 2.05E-18 | 2.17E-17 |
| Elmod2 | 1.571 | 2.10E-18 | 2.22E-17 |
| Eif2d | 1.197 | 2.40E-18 | 2.53E-17 |
| Abhd4 | 1.215 | 2.51E-18 | 2.65E-17 |
| Rgs16 | 2.507 | 2.56E-18 | 2.70E-17 |
| Sowahb | 1.257 | 2.63E-18 | 2.76E-17 |
| Ppfibp1 | 1.534 | 2.67E-18 | 2.81E-17 |
| Cela3b | -12.359 | 2.71E-18 | 2.85E-17 |
| 1810055G02Rik | 1.227 | 2.98E-18 | 3.13E-17 |
| Arl2bp | 1.434 | 3.10E-18 | 3.25E-17 |
| Chpf2 | 1.148 | 3.15E-18 | 3.29E-17 |
| Thop1 | 1.372 | 3.46E-18 | 3.61E-17 |
| Rnf126 | 1.124 | 3.67E-18 | 3.83E-17 |
| Clstn3 | -1.888 | 3.98E-18 | 4.15E-17 |
| Cpb1 | -12.451 | 4.39E-18 | 4.57E-17 |
| Orc5 | 1.099 | 4.94E-18 | 5.13E-17 |
| Glce | 1.326 | 5.58E-18 | 5.77E-17 |
| Ubxn8 | 1.074 | 5.60E-18 | 5.79E-17 |
| Dph1 | 1.015 | 5.81E-18 | 6.01E-17 |
| Tomm5 | 1.290 | 5.90E-18 | 6.09E-17 |
| Gnl2 | 1.350 | 6.39E-18 | 6.59E-17 |
| Isg20 | 1.986 | 8.10E-18 | 8.33E-17 |
| Rpl35 | 1.029 | 8.52E-18 | 8.74E-17 |
| Heyl | -2.298 | 9.06E-18 | 9.26E-17 |
| Car1 | -1.990 | 9.06E-18 | 9.26E-17 |
| Pparg | 1.202 | 9.35E-18 | 9.55E-17 |
| Nat10 | 1.077 | 9.61E-18 | 9.81E-17 |
| Dnajc3 | 1.183 | 9.69E-18 | 9.89E-17 |
| Yod1 | 1.626 | 1.04E-17 | 1.06E-16 |
| Dnajc2 | 1.718 | 1.09E-17 | 1.11E-16 |
| Pef1 | 1.001 | 1.11E-17 | 1.13E-16 |
| Hspe1 | 1.069 | 1.28E-17 | 1.30E-16 |
| Gstp3 | 1.552 | 1.37E-17 | 1.39E-16 |
| Ranbp1 | 1.038 | 1.44E-17 | 1.46E-16 |
| Pla2g7 | 1.462 | 1.52E-17 | 1.54E-16 |
| Tasp1 | 2.085 | 1.52E-17 | 1.54E-16 |
| Prtn3 | 4.868 | 1.76E-17 | 1.78E-16 |
| Slc10a2 | 1.296 | 1.91E-17 | 1.92E-16 |
| Eif3a | 1.493 | 1.92E-17 | 1.93E-16 |
| Cwc27 | 2.694 | 1.99E-17 | 2.00E-16 |
| Itgam | 2.014 | 2.14E-17 | 2.14E-16 |
| Ccnb1 | 2.956 | 2.22E-17 | 2.22E-16 |
| Ckmt2 | -11.223 | 2.27E-17 | 2.27E-16 |
| Chil3 | 5.000 | 2.81E-17 | 2.80E-16 |
| Rnase1 | -12.054 | 2.86E-17 | 2.85E-16 |
| Rbm18 | 1.102 | 3.05E-17 | 3.03E-16 |
| Mios | 1.689 | 3.23E-17 | 3.21E-16 |
| Muc1 | -1.957 | 3.42E-17 | 3.39E-16 |
| Elac1 | 1.488 | 3.80E-17 | 3.76E-16 |
| Zfyve19 | 1.158 | 4.10E-17 | 4.04E-16 |
| Tnpo2 | 1.071 | 4.12E-17 | 4.06E-16 |
| Hsd3b1 | -8.323 | 5.33E-17 | 5.24E-16 |
| Lama4 | -1.042 | 5.72E-17 | 5.61E-16 |
| Brix1 | 1.328 | 5.80E-17 | 5.69E-16 |
| Slc45a4 | 1.353 | 6.14E-17 | 6.02E-16 |
| Gripap1 | 1.142 | 6.62E-17 | 6.48E-16 |
| Dis3 | 1.436 | 6.65E-17 | 6.50E-16 |
| Pnliprp1 | -11.897 | 7.23E-17 | 7.07E-16 |
| Nsmce1 | 1.286 | 7.42E-17 | 7.24E-16 |
| Tma16 | 1.992 | 8.54E-17 | 8.30E-16 |
| Dph5 | 1.557 | 8.71E-17 | 8.46E-16 |
| Ssbp1 | 1.221 | 9.57E-17 | 9.29E-16 |
| Trmt10a | 1.345 | 9.79E-17 | 9.49E-16 |
| Acot6 | 2.977 | 9.94E-17 | 9.64E-16 |
| Tnnt3 | -8.086 | 1.03E-16 | 9.93E-16 |
| Cebpg | 1.325 | 1.19E-16 | 1.15E-15 |
| Nfkbib | 1.143 | 1.26E-16 | 1.21E-15 |
| Timm10 | 1.214 | 1.50E-16 | 1.44E-15 |
| Amigo3 | 2.898 | 1.54E-16 | 1.48E-15 |
| Itpkc | 1.310 | 1.62E-16 | 1.55E-15 |
| Reg1 | -5.295 | 1.66E-16 | 1.58E-15 |
| Mst1 | 1.005 | 1.77E-16 | 1.69E-15 |
| Ears2 | 1.441 | 1.90E-16 | 1.81E-15 |
| Slc6a9 | 1.103 | 1.93E-16 | 1.83E-15 |
| Kdm4a | 1.015 | 1.97E-16 | 1.88E-15 |
| Myh4 | -9.015 | 2.09E-16 | 1.99E-15 |
| Tnnc2 | -11.991 | 2.16E-16 | 2.06E-15 |
| Sorbs2 | 1.130 | 2.25E-16 | 2.14E-15 |
| Thra | -1.052 | 2.25E-16 | 2.14E-15 |
| Hhipl2 | 4.940 | 2.28E-16 | 2.16E-15 |
| Slc7a11 | 8.392 | 2.45E-16 | 2.32E-15 |
| Golt1b | 1.580 | 2.50E-16 | 2.37E-15 |
| Tnni2 | -11.936 | 2.72E-16 | 2.57E-15 |
| Ints6 | 1.273 | 2.87E-16 | 2.70E-15 |
| Cyp11b1 | -9.929 | 2.88E-16 | 2.72E-15 |
| Pold2 | 1.310 | 2.96E-16 | 2.78E-15 |
| Polr1e | 1.600 | 3.01E-16 | 2.83E-15 |
| Kif20a | 2.495 | 3.07E-16 | 2.89E-15 |
| Tmem184c | 1.355 | 3.30E-16 | 3.10E-15 |
| Dhx57 | 1.251 | 3.78E-16 | 3.54E-15 |
| Kif16b | 1.256 | 4.01E-16 | 3.76E-15 |
| Arfgap1 | 1.139 | 4.12E-16 | 3.85E-15 |
| Gca | 1.915 | 4.84E-16 | 4.52E-15 |
| Ube2j2 | 1.102 | 6.43E-16 | 5.99E-15 |
| Pyroxd1 | 1.607 | 6.62E-16 | 6.16E-15 |
| Clcf1 | 3.294 | 6.67E-16 | 6.21E-15 |
| Myl1 | -11.602 | 6.98E-16 | 6.48E-15 |
| Mapre3 | 1.118 | 6.99E-16 | 6.48E-15 |
| Secisbp2 | 1.137 | 7.64E-16 | 7.07E-15 |
| Abca8b | 1.084 | 8.20E-16 | 7.58E-15 |
| Prc1 | 2.784 | 9.13E-16 | 8.42E-15 |
| Nle1 | 2.366 | 9.40E-16 | 8.67E-15 |
| Usp16 | 1.683 | 1.01E-15 | 9.27E-15 |
| Saa3 | 1.756 | 1.05E-15 | 9.66E-15 |
| Cntnap1 | 3.020 | 1.12E-15 | 1.02E-14 |
| Wnk4 | -1.923 | 1.17E-15 | 1.07E-14 |
| Plscr2 | 1.112 | 1.20E-15 | 1.10E-14 |
| Plin4 | -1.247 | 1.26E-15 | 1.15E-14 |
| Ccdc127 | 1.470 | 1.43E-15 | 1.31E-14 |
| Cspg5 | -1.985 | 1.62E-15 | 1.47E-14 |
| Mrto4 | 1.295 | 1.62E-15 | 1.47E-14 |
| Chrnb2 | -2.254 | 1.63E-15 | 1.48E-14 |
| Adpgk | 1.037 | 1.84E-15 | 1.67E-14 |
| Mospd1 | 1.554 | 1.86E-15 | 1.69E-14 |
| Tarbp2 | 1.265 | 1.88E-15 | 1.71E-14 |
| Invs | 1.113 | 2.01E-15 | 1.81E-14 |
| Unc5b | 1.521 | 2.14E-15 | 1.93E-14 |
| Ndor1 | 1.243 | 2.45E-15 | 2.20E-14 |
| Gbp10 | -1.834 | 2.62E-15 | 2.35E-14 |
| Flvcr1 | 1.829 | 2.71E-15 | 2.44E-14 |
| Kti12 | 1.050 | 2.86E-15 | 2.57E-14 |
| Tceal9 | 1.165 | 2.93E-15 | 2.62E-14 |
| Cpa2 | -11.102 | 3.00E-15 | 2.68E-14 |
| Pex12 | 1.178 | 3.04E-15 | 2.72E-14 |
| Arhgef26 | 1.158 | 3.06E-15 | 2.74E-14 |
| Nol9 | 1.082 | 3.31E-15 | 2.96E-14 |
| Trim24 | 1.420 | 3.43E-15 | 3.06E-14 |
| Clps | -12.081 | 3.47E-15 | 3.09E-14 |
| Zbtb18 | 1.119 | 3.50E-15 | 3.11E-14 |
| Clcn3 | 1.108 | 3.73E-15 | 3.32E-14 |
| Nup93 | 1.534 | 3.82E-15 | 3.40E-14 |
| Stam2 | 1.064 | 3.96E-15 | 3.51E-14 |
| Smurf1 | 1.024 | 4.02E-15 | 3.56E-14 |
| Magoh | 1.144 | 4.18E-15 | 3.70E-14 |
| Eif2s2 | 1.386 | 4.42E-15 | 3.91E-14 |
| Wdr4 | 1.071 | 4.73E-15 | 4.17E-14 |
| Ruvbl1 | 1.143 | 4.93E-15 | 4.34E-14 |
| Lrrc8a | 1.080 | 5.34E-15 | 4.69E-14 |
| Arhgap6 | -1.510 | 5.46E-15 | 4.79E-14 |
| Smim7 | 1.090 | 5.69E-15 | 4.99E-14 |
| S100a11 | 1.818 | 5.75E-15 | 5.04E-14 |
| Etf1 | 1.225 | 5.93E-15 | 5.19E-14 |
| Rpf2 | 1.572 | 6.05E-15 | 5.29E-14 |
| Bahcc1 | -1.013 | 7.96E-15 | 6.94E-14 |
| Tmem263 | 1.494 | 8.05E-15 | 7.01E-14 |
| Xpo4 | 1.234 | 1.01E-14 | 8.76E-14 |
| Qsox2 | 1.235 | 1.07E-14 | 9.26E-14 |
| Ctrl | -7.753 | 1.16E-14 | 1.00E-13 |
| Slc2a1 | 1.164 | 1.18E-14 | 1.02E-13 |
| Cc2d1b | 1.183 | 1.24E-14 | 1.07E-13 |
| Nop14 | 1.225 | 1.43E-14 | 1.23E-13 |
| Them7 | 1.151 | 1.59E-14 | 1.36E-13 |
| Fmod | -1.474 | 1.70E-14 | 1.46E-13 |
| Aprt | 1.197 | 1.77E-14 | 1.51E-13 |
| Tmem14a | -1.388 | 1.77E-14 | 1.51E-13 |
| Gar1 | 2.350 | 1.96E-14 | 1.68E-13 |
| Adck1 | 1.101 | 1.97E-14 | 1.68E-13 |
| Aatk | -1.744 | 2.02E-14 | 1.72E-13 |
| Lsg1 | 1.004 | 2.04E-14 | 1.74E-13 |
| Tasor2 | 1.714 | 2.19E-14 | 1.87E-13 |
| Rnf186 | -1.784 | 2.25E-14 | 1.91E-13 |
| Glt8d1 | 1.498 | 2.38E-14 | 2.02E-13 |
| Lrit2 | -2.195 | 2.76E-14 | 2.34E-13 |
| Clk4 | 1.272 | 2.78E-14 | 2.35E-13 |
| A2ml1 | 1.239 | 2.83E-14 | 2.39E-13 |
| Kansl2 | 1.013 | 2.92E-14 | 2.47E-13 |
| Tmem258 | 1.268 | 3.17E-14 | 2.67E-13 |
| Chd1l | 1.168 | 3.19E-14 | 2.68E-13 |
| Cep76 | 1.584 | 3.22E-14 | 2.71E-13 |
| EPPK1 | 1.123 | 3.34E-14 | 2.81E-13 |
| Bmper | 2.935 | 3.62E-14 | 3.03E-13 |
| Bcap29 | 1.550 | 4.00E-14 | 3.34E-13 |
| Asb3 | 1.314 | 4.12E-14 | 3.43E-13 |
| Farsa | 1.033 | 4.14E-14 | 3.44E-13 |
| Lad1 | 1.317 | 4.25E-14 | 3.54E-13 |
| Swsap1 | 1.307 | 4.71E-14 | 3.92E-13 |
| Ighm | -1.203 | 4.99E-14 | 4.14E-13 |
| Eif1ad | 1.011 | 5.10E-14 | 4.23E-13 |
| Tmprss2 | 1.503 | 5.31E-14 | 4.40E-13 |
| Hnrnpa1 | 1.082 | 5.66E-14 | 4.68E-13 |
| Cdc20 | 2.645 | 5.68E-14 | 4.69E-13 |
| Ticam1 | 1.038 | 5.74E-14 | 4.74E-13 |
| Fam187b | 1.528 | 5.78E-14 | 4.77E-13 |
| Slc22a3 | 1.697 | 6.29E-14 | 5.18E-13 |
| Rrp15 | 1.368 | 6.66E-14 | 5.47E-13 |
| Mycl | 1.490 | 7.12E-14 | 5.84E-13 |
| 2700097O09Rik | 2.118 | 7.53E-14 | 6.17E-13 |
| Nat9 | 1.091 | 8.27E-14 | 6.76E-13 |
| 2610002M06Rik | 1.242 | 8.47E-14 | 6.93E-13 |
| Zscan26 | 1.198 | 8.48E-14 | 6.93E-13 |
| Btg2 | 1.166 | 9.97E-14 | 8.12E-13 |
| Rbp1 | 1.059 | 1.00E-13 | 8.15E-13 |
| Muc3 | -3.883 | 1.07E-13 | 8.70E-13 |
| Champ1 | 1.283 | 1.15E-13 | 9.32E-13 |
| Mia3 | 1.244 | 1.26E-13 | 1.02E-12 |
| Unkl | 1.008 | 1.37E-13 | 1.11E-12 |
| Cdc25a | 1.372 | 1.43E-13 | 1.15E-12 |
| Spire2 | 4.709 | 1.65E-13 | 1.33E-12 |
| Cyp20a1 | 1.122 | 1.69E-13 | 1.36E-12 |
| Phf10 | 1.209 | 1.89E-13 | 1.52E-12 |
| Ppip5k2 | 1.199 | 1.90E-13 | 1.52E-12 |
| Zg16 | -11.483 | 2.24E-13 | 1.79E-12 |
| Tcap | -10.785 | 2.26E-13 | 1.81E-12 |
| Flad1 | 1.009 | 2.47E-13 | 1.97E-12 |
| Plekha1 | 1.243 | 2.71E-13 | 2.16E-12 |
| Ankrd23 | -2.201 | 2.77E-13 | 2.20E-12 |
| Pnliprp2 | -10.288 | 3.12E-13 | 2.47E-12 |
| Rabl3 | 1.153 | 3.17E-13 | 2.51E-12 |
| Pdzd11 | 1.111 | 3.19E-13 | 2.52E-12 |
| Rab23 | 1.660 | 3.28E-13 | 2.60E-12 |
| Shc4 | 4.387 | 3.50E-13 | 2.76E-12 |
| Rrp1b | 1.415 | 3.60E-13 | 2.84E-12 |
| Cdin1 | 1.809 | 3.61E-13 | 2.85E-12 |
| Dmtf1 | 1.348 | 3.79E-13 | 2.98E-12 |
| Kpna4 | 1.205 | 3.87E-13 | 3.05E-12 |
| Cyp11a1 | -7.489 | 4.03E-13 | 3.17E-12 |
| Fam135a | 2.129 | 4.42E-13 | 3.47E-12 |
| Abcb8 | 1.002 | 4.70E-13 | 3.68E-12 |
| Sap130 | 1.050 | 4.73E-13 | 3.70E-12 |
| Eef1e1 | 1.223 | 4.91E-13 | 3.84E-12 |
| Tsc22d2 | 1.481 | 4.95E-13 | 3.86E-12 |
| Tmem125 | 1.335 | 5.09E-13 | 3.96E-12 |
| Myh7 | -8.288 | 5.13E-13 | 4.00E-12 |
| Trim39 | 1.146 | 5.53E-13 | 4.30E-12 |
| Dpm2 | 1.133 | 5.66E-13 | 4.40E-12 |
| Rpp38 | 2.077 | 6.07E-13 | 4.70E-12 |
| Eid1 | 1.255 | 6.17E-13 | 4.77E-12 |
| H2ac18 | 1.021 | 6.31E-13 | 4.88E-12 |
| Abraxas1 | 1.519 | 6.63E-13 | 5.11E-12 |
| Mpzl3 | 1.124 | 6.66E-13 | 5.14E-12 |
| Il17ra | 1.025 | 7.41E-13 | 5.70E-12 |
| Mettl3 | 1.168 | 7.64E-13 | 5.88E-12 |
| Slc39a6 | 1.463 | 7.70E-13 | 5.92E-12 |
| Chga | -9.969 | 8.42E-13 | 6.46E-12 |
| A2m | 4.206 | 8.76E-13 | 6.70E-12 |
| Gm33543 | 1.505 | 9.50E-13 | 7.26E-12 |
| Usp48 | 1.013 | 9.52E-13 | 7.27E-12 |
| Ciart | 3.175 | 9.66E-13 | 7.37E-12 |
| Golga4 | 1.386 | 9.97E-13 | 7.60E-12 |
| Wars | 1.121 | 1.05E-12 | 7.99E-12 |
| Creb3 | 1.001 | 1.06E-12 | 8.03E-12 |
| Ankrd49 | 1.764 | 1.11E-12 | 8.40E-12 |
| Eef1akmt3 | 9.701 | 1.11E-12 | 8.42E-12 |
| Stag1 | 1.491 | 1.14E-12 | 8.65E-12 |
| Tubgcp2 | 1.445 | 1.22E-12 | 9.21E-12 |
| Eif5 | 1.099 | 1.22E-12 | 9.24E-12 |
| Mlh1 | 1.218 | 1.44E-12 | 1.09E-11 |
| Rassf6 | 1.137 | 1.68E-12 | 1.26E-11 |
| Cyp2b9 | 2.595 | 1.69E-12 | 1.27E-11 |
| Top2a | 2.538 | 1.70E-12 | 1.28E-11 |
| Pelo | 1.185 | 1.72E-12 | 1.29E-11 |
| Tmem165 | 1.128 | 1.73E-12 | 1.30E-11 |
| Flot1 | 1.093 | 1.74E-12 | 1.30E-11 |
| Bckdk | 1.118 | 1.89E-12 | 1.41E-11 |
| Mllt11 | 2.264 | 2.00E-12 | 1.49E-11 |
| Gm7694 | 1.319 | 2.06E-12 | 1.54E-11 |
| Mmachc | 1.159 | 2.12E-12 | 1.58E-11 |
| Ccdc186 | 1.478 | 2.27E-12 | 1.68E-11 |
| Slc35a4 | 1.175 | 2.31E-12 | 1.71E-11 |
| Eogt | 1.356 | 2.32E-12 | 1.72E-11 |
| Mta1 | 1.007 | 2.49E-12 | 1.84E-11 |
| Smndc1 | 1.023 | 2.54E-12 | 1.88E-11 |
| Fam47e | 1.205 | 2.68E-12 | 1.97E-11 |
| Pdgfa | 1.841 | 2.74E-12 | 2.02E-11 |
| Spring1 | 1.232 | 2.79E-12 | 2.05E-11 |
| H3c15 | 2.270 | 2.95E-12 | 2.16E-11 |
| E030018B13Rik | 2.576 | 3.00E-12 | 2.20E-11 |
| Tsr2 | 1.252 | 3.14E-12 | 2.30E-11 |
| Isyna1 | 1.394 | 3.18E-12 | 2.33E-11 |
| Nrap | -3.147 | 3.48E-12 | 2.55E-11 |
| Dennd4a | 1.034 | 3.66E-12 | 2.68E-11 |
| Taf1b | 1.781 | 3.78E-12 | 2.76E-11 |
| Gabrb3 | -2.609 | 3.92E-12 | 2.86E-11 |
| Ccdc130 | 1.349 | 3.93E-12 | 2.87E-11 |
| Arl8a | 1.062 | 4.05E-12 | 2.95E-11 |
| Mybpc1 | -8.740 | 4.11E-12 | 3.00E-11 |
| Fhl1 | -1.060 | 4.21E-12 | 3.06E-11 |
| Ercc6l2 | 1.117 | 4.27E-12 | 3.10E-11 |
| 1810030O07Rik | 1.198 | 4.30E-12 | 3.13E-11 |
| Tbce | 1.246 | 4.48E-12 | 3.25E-11 |
| Riox2 | 1.164 | 4.79E-12 | 3.48E-11 |
| Lrtm1 | 1.681 | 5.21E-12 | 3.77E-11 |
| Med7 | 1.111 | 5.33E-12 | 3.86E-11 |
| Cenpm | 3.711 | 6.02E-12 | 4.34E-11 |
| Lgals3 | 1.240 | 6.21E-12 | 4.48E-11 |
| Ccdc9 | 1.301 | 6.85E-12 | 4.93E-11 |
| Gtpbp4 | 1.175 | 7.49E-12 | 5.38E-11 |
| Mtln | 1.535 | 7.52E-12 | 5.40E-11 |
| Fip1l1 | 1.059 | 7.61E-12 | 5.46E-11 |
| Wdr12 | 1.225 | 7.84E-12 | 5.62E-11 |
| Ruvbl2 | 1.001 | 7.92E-12 | 5.67E-11 |
| Cxcl1 | 1.897 | 8.33E-12 | 5.96E-11 |
| Mdn1 | 1.469 | 8.42E-12 | 6.03E-11 |
| Cpne8 | 1.478 | 8.92E-12 | 6.37E-11 |
| Obscn | -6.555 | 9.42E-12 | 6.72E-11 |
| St7 | 1.197 | 1.08E-11 | 7.68E-11 |
| Bet1l | 1.088 | 1.19E-11 | 8.45E-11 |
| Fam89a | -1.086 | 1.21E-11 | 8.60E-11 |
| Tmem185b | 1.177 | 1.30E-11 | 9.19E-11 |
| Klk1 | -5.166 | 1.33E-11 | 9.41E-11 |
| D630045J12Rik | 1.387 | 1.45E-11 | 1.03E-10 |
| Eef1aknmt | 1.237 | 1.49E-11 | 1.05E-10 |
| Trmt9b | -1.147 | 1.60E-11 | 1.13E-10 |
| Ntmt1 | 1.498 | 1.62E-11 | 1.14E-10 |
| Hhipl1 | 3.196 | 1.71E-11 | 1.20E-10 |
| Dact1 | 1.879 | 1.74E-11 | 1.22E-10 |
| Fabp3 | -7.158 | 1.79E-11 | 1.26E-10 |
| Jade1 | 1.182 | 1.87E-11 | 1.31E-10 |
| Dtwd1 | 1.268 | 1.88E-11 | 1.32E-10 |
| Zfp518b | 1.663 | 2.00E-11 | 1.40E-10 |
| Uprt | 2.503 | 2.12E-11 | 1.48E-10 |
| Prss3 | -10.558 | 2.13E-11 | 1.48E-10 |
| Sprr1a | 11.158 | 2.15E-11 | 1.50E-10 |
| F13a1 | 3.284 | 2.22E-11 | 1.54E-10 |
| Ube2c | 2.528 | 2.32E-11 | 1.61E-10 |
| Dcaf1 | 1.084 | 2.33E-11 | 1.62E-10 |
| Siah2 | 1.026 | 2.38E-11 | 1.65E-10 |
| Mtmr11 | 2.021 | 2.57E-11 | 1.78E-10 |
| Josd2 | 1.217 | 2.59E-11 | 1.79E-10 |
| Fbxo4 | 1.072 | 2.63E-11 | 1.82E-10 |
| Gm6614 | 6.954 | 2.86E-11 | 1.97E-10 |
| Nmral1 | 1.306 | 3.09E-11 | 2.13E-10 |
| RPS6KA4 | 1.193 | 3.13E-11 | 2.15E-10 |
| Tle2 | -1.249 | 3.28E-11 | 2.25E-10 |
| Smarcb1 | 1.061 | 3.31E-11 | 2.27E-10 |
| Dkc1 | 1.697 | 3.34E-11 | 2.29E-10 |
| Areg | 10.441 | 3.50E-11 | 2.40E-10 |
| Vangl1 | 1.220 | 3.52E-11 | 2.41E-10 |
| Mvk | 1.000 | 3.66E-11 | 2.50E-10 |
| Igha | -1.085 | 3.99E-11 | 2.72E-10 |
| Gm8113 | 2.931 | 4.09E-11 | 2.78E-10 |
| Flywch1 | 1.048 | 4.32E-11 | 2.94E-10 |
| Gtf2a2 | 1.021 | 4.65E-11 | 3.15E-10 |
| Qtrt2 | 1.580 | 4.70E-11 | 3.19E-10 |
| Hspb7 | -3.553 | 4.88E-11 | 3.30E-10 |
| 2410002F23Rik | 1.368 | 4.90E-11 | 3.32E-10 |
| Ing5 | 1.159 | 4.91E-11 | 3.32E-10 |
| Rbl1 | 2.093 | 5.04E-11 | 3.41E-10 |
| Krr1 | 1.087 | 5.16E-11 | 3.49E-10 |
| Lins1 | 1.435 | 5.22E-11 | 3.52E-10 |
| Sycn | -11.263 | 5.28E-11 | 3.56E-10 |
| Rhof | 2.590 | 5.40E-11 | 3.64E-10 |
| Dlgap4 | 1.138 | 5.47E-11 | 3.69E-10 |
| Cebpd | 1.298 | 5.50E-11 | 3.70E-10 |
| Ccdc115 | 1.063 | 5.64E-11 | 3.79E-10 |
| Sall2 | -1.195 | 5.65E-11 | 3.80E-10 |
| Nlgn2 | -1.986 | 6.00E-11 | 4.03E-10 |
| Dll4 | -1.023 | 6.10E-11 | 4.09E-10 |
| Marveld2 | 1.177 | 6.22E-11 | 4.17E-10 |
| Usp1 | 1.254 | 6.59E-11 | 4.41E-10 |
| Dnajc21 | 1.250 | 6.75E-11 | 4.51E-10 |
| Pus1 | 1.132 | 7.19E-11 | 4.80E-10 |
| Tmem129 | 1.033 | 7.49E-11 | 4.99E-10 |
| Eef2k | 1.154 | 7.59E-11 | 5.05E-10 |
| Wdr73 | 1.178 | 7.62E-11 | 5.06E-10 |
| Zfp945 | 1.503 | 7.81E-11 | 5.19E-10 |
| Tatdn1 | 1.431 | 7.88E-11 | 5.23E-10 |
| Nat8f5 | 1.103 | 8.07E-11 | 5.35E-10 |
| Krcc1 | 1.380 | 8.32E-11 | 5.50E-10 |
| Gabpa | 1.214 | 9.42E-11 | 6.21E-10 |
| Itfg2 | 1.233 | 1.02E-10 | 6.69E-10 |
| Gtf3c5 | 1.169 | 1.05E-10 | 6.87E-10 |
| Hook1 | 1.254 | 1.07E-10 | 7.00E-10 |
| Sec61g | 1.107 | 1.12E-10 | 7.33E-10 |
| Ccna2 | 2.232 | 1.25E-10 | 8.16E-10 |
| Rad17 | 1.291 | 1.27E-10 | 8.31E-10 |
| Gstt2 | 1.052 | 1.27E-10 | 8.31E-10 |
| Noc3l | 1.325 | 1.37E-10 | 8.98E-10 |
| Mmp12 | 3.333 | 1.39E-10 | 9.07E-10 |
| Tdp1 | 1.621 | 1.44E-10 | 9.37E-10 |
| Rnf6 | 1.104 | 1.44E-10 | 9.42E-10 |
| Zfand2a | 1.109 | 1.49E-10 | 9.70E-10 |
| Csgalnact2 | 1.199 | 1.50E-10 | 9.74E-10 |
| Rps29 | 1.057 | 1.53E-10 | 9.96E-10 |
| Lrp2bp | -3.084 | 1.55E-10 | 1.01E-09 |
| Snapc4 | 1.234 | 1.71E-10 | 1.11E-09 |
| Alg8 | 1.437 | 1.73E-10 | 1.12E-09 |
| Gucy2c | 2.568 | 1.74E-10 | 1.12E-09 |
| Adm2 | 10.116 | 1.84E-10 | 1.19E-09 |
| Ifi27l2b | 1.494 | 2.01E-10 | 1.29E-09 |
| Actn2 | -6.539 | 2.14E-10 | 1.38E-09 |
| Zbtb11 | 1.242 | 2.25E-10 | 1.44E-09 |
| Ddr1 | 1.393 | 2.26E-10 | 1.45E-09 |
| Cdc73 | 1.130 | 2.65E-10 | 1.69E-09 |
| Amy2a3 | -11.348 | 2.80E-10 | 1.78E-09 |
| Amy2a2 | -11.348 | 2.80E-10 | 1.78E-09 |
| Amy2a4 | -11.348 | 2.80E-10 | 1.78E-09 |
| Pus3 | 1.722 | 2.82E-10 | 1.80E-09 |
| Fundc1 | 1.209 | 3.00E-10 | 1.91E-09 |
| Ryr1 | -5.358 | 3.23E-10 | 2.05E-09 |
| Rusf1 | 1.003 | 3.26E-10 | 2.06E-09 |
| Capn3 | -1.881 | 3.33E-10 | 2.11E-09 |
| Nek4 | 1.092 | 3.39E-10 | 2.14E-09 |
| Cnbd2 | 1.369 | 3.42E-10 | 2.16E-09 |
| Chchd5 | 1.079 | 3.46E-10 | 2.18E-09 |
| Exosc3 | 1.101 | 3.59E-10 | 2.26E-09 |
| Necab1 | 1.170 | 3.62E-10 | 2.28E-09 |
| Art3 | -1.576 | 3.84E-10 | 2.41E-09 |
| Polr1has | 1.198 | 3.94E-10 | 2.47E-09 |
| Hinfp | 1.085 | 4.06E-10 | 2.54E-09 |
| Nol8 | 2.645 | 4.16E-10 | 2.60E-09 |
| Med20 | 1.051 | 4.17E-10 | 2.61E-09 |
| Nob1 | 1.163 | 4.35E-10 | 2.72E-09 |
| Zfp146 | 1.314 | 4.39E-10 | 2.74E-09 |
| Arhgap12 | 1.236 | 4.52E-10 | 2.82E-09 |
| Cep192 | 1.825 | 4.55E-10 | 2.84E-09 |
| Pvalb | -9.849 | 4.71E-10 | 2.94E-09 |
| Syt3 | -1.136 | 4.78E-10 | 2.98E-09 |
| Endod1 | -1.099 | 5.13E-10 | 3.19E-09 |
| Utp18 | 1.105 | 5.16E-10 | 3.20E-09 |
| Mrpl47 | 1.108 | 5.28E-10 | 3.28E-09 |
| Dmbt1 | -2.823 | 5.36E-10 | 3.32E-09 |
| Dph6 | 1.339 | 5.61E-10 | 3.46E-09 |
| Cnot4 | 1.060 | 5.66E-10 | 3.49E-09 |
| Rec8 | -2.353 | 5.82E-10 | 3.58E-09 |
| Slco1a4 | 1.046 | 5.87E-10 | 3.62E-09 |
| Ttc37 | 1.335 | 6.10E-10 | 3.75E-09 |
| Rsad1 | 1.585 | 6.28E-10 | 3.86E-09 |
| Det1 | 1.282 | 6.30E-10 | 3.87E-09 |
| Serpinb1a | -1.465 | 6.64E-10 | 4.07E-09 |
| Ctrb1 | -8.228 | 6.81E-10 | 4.18E-09 |
| Sult1d1 | 1.114 | 7.13E-10 | 4.36E-09 |
| Atpsckmt | 1.070 | 7.33E-10 | 4.48E-09 |
| Pspc1 | 1.150 | 7.34E-10 | 4.49E-09 |
| Odr4 | 1.983 | 7.73E-10 | 4.72E-09 |
| Adipoq | -6.622 | 7.98E-10 | 4.87E-09 |
| Fcgbp | -3.129 | 8.01E-10 | 4.88E-09 |
| Tpx2 | 2.643 | 8.12E-10 | 4.94E-09 |
| Tmem230 | 1.018 | 8.20E-10 | 5.00E-09 |
| Dhrs13 | 1.455 | 8.70E-10 | 5.28E-09 |
| Polr2h | 1.150 | 8.95E-10 | 5.43E-09 |
| Nkiras1 | 1.016 | 9.19E-10 | 5.57E-09 |
| Iffo2 | 1.022 | 9.21E-10 | 5.58E-09 |
| Dusp12 | 1.088 | 9.46E-10 | 5.73E-09 |
| Qser1 | 1.131 | 9.52E-10 | 5.76E-09 |
| Aebp2 | 1.162 | 1.03E-09 | 6.21E-09 |
| Exosc1 | 1.124 | 1.07E-09 | 6.47E-09 |
| Cib3 | -3.501 | 1.18E-09 | 7.12E-09 |
| Syncrip | 1.129 | 1.25E-09 | 7.50E-09 |
| Dlg5 | 1.043 | 1.29E-09 | 7.76E-09 |
| Snupn | 1.340 | 1.29E-09 | 7.76E-09 |
| Gp2 | -6.173 | 1.29E-09 | 7.76E-09 |
| Ctps2 | 1.318 | 1.32E-09 | 7.94E-09 |
| Srl | -1.955 | 1.35E-09 | 8.07E-09 |
| Gps2 | 1.291 | 1.36E-09 | 8.13E-09 |
| Cebpz | 1.248 | 1.41E-09 | 8.45E-09 |
| Cyp2c39 | 1.751 | 1.45E-09 | 8.66E-09 |
| Tra2b | 1.009 | 1.48E-09 | 8.85E-09 |
| Npm3 | 1.220 | 1.54E-09 | 9.15E-09 |
| Clip1 | 1.041 | 1.75E-09 | 1.04E-08 |
| Nudt18 | 1.307 | 1.82E-09 | 1.08E-08 |
| Gtf2a1 | 1.135 | 1.82E-09 | 1.08E-08 |
| Cep83 | 1.577 | 1.93E-09 | 1.14E-08 |
| Ccl21a | -1.235 | 1.95E-09 | 1.15E-08 |
| Sult1e1 | 4.899 | 1.98E-09 | 1.17E-08 |
| Rfesd | 1.196 | 2.00E-09 | 1.18E-08 |
| Dxo | 1.104 | 2.01E-09 | 1.18E-08 |
| Aftph | 1.057 | 2.07E-09 | 1.22E-08 |
| Mpp7 | 1.240 | 2.14E-09 | 1.26E-08 |
| Rbm39 | 1.185 | 2.16E-09 | 1.27E-08 |
| Sclt1 | 1.886 | 2.17E-09 | 1.28E-08 |
| Serpina9 | -2.165 | 2.27E-09 | 1.33E-08 |
| Knstrn | 2.002 | 2.27E-09 | 1.34E-08 |
| Naa50 | 1.006 | 2.32E-09 | 1.36E-08 |
| Pdcl | 1.068 | 2.32E-09 | 1.36E-08 |
| Tax1bp1 | 1.194 | 2.41E-09 | 1.41E-08 |
| Pnpla8 | 1.095 | 2.45E-09 | 1.43E-08 |
| Prmt5 | 1.017 | 2.55E-09 | 1.49E-08 |
| Fnbp1l | 1.395 | 2.85E-09 | 1.66E-08 |
| Sstr2 | 1.560 | 2.87E-09 | 1.67E-08 |
| Ttn | -4.322 | 2.87E-09 | 1.67E-08 |
| Igdcc4 | 1.762 | 2.94E-09 | 1.71E-08 |
| Gm34653 | 9.388 | 3.00E-09 | 1.74E-08 |
| Rhbdd2 | 1.099 | 3.26E-09 | 1.88E-08 |
| D16Ertd472e | 1.044 | 3.47E-09 | 2.00E-08 |
| C330018D20Rik | 1.084 | 3.52E-09 | 2.03E-08 |
| Alg3 | 1.108 | 3.58E-09 | 2.06E-08 |
| Taf15 | 1.074 | 3.60E-09 | 2.07E-08 |
| Lims1 | 1.096 | 3.62E-09 | 2.08E-08 |
| Gyg | 1.218 | 3.64E-09 | 2.10E-08 |
| Fra10ac1 | 1.394 | 3.68E-09 | 2.12E-08 |
| Dusp8 | 1.419 | 3.81E-09 | 2.19E-08 |
| Cux2 | 2.951 | 3.98E-09 | 2.29E-08 |
| Phf14 | 1.319 | 4.28E-09 | 2.45E-08 |
| Trp53inp1 | 1.677 | 4.51E-09 | 2.58E-08 |
| Hmmr | 3.155 | 4.69E-09 | 2.67E-08 |
| Rora | 1.726 | 4.70E-09 | 2.68E-08 |
| Taf4b | 1.565 | 4.72E-09 | 2.69E-08 |
| Chgb | -9.630 | 4.79E-09 | 2.73E-08 |
| Pstpip2 | 1.039 | 5.07E-09 | 2.89E-08 |
| Zbtb7c | -1.436 | 5.43E-09 | 3.08E-08 |
| Zfp617 | 1.371 | 5.83E-09 | 3.30E-08 |
| Col5a2 | 1.267 | 6.42E-09 | 3.63E-08 |
| Ttll7 | 2.080 | 6.57E-09 | 3.71E-08 |
| Plin1 | -7.109 | 6.70E-09 | 3.77E-08 |
| Eef1a2 | -5.797 | 6.79E-09 | 3.82E-08 |
| Clec2h | -1.955 | 7.61E-09 | 4.27E-08 |
| Ahi1 | 2.042 | 7.77E-09 | 4.36E-08 |
| Mettl2 | 1.024 | 7.79E-09 | 4.36E-08 |
| Riok1 | 1.252 | 8.51E-09 | 4.76E-08 |
| Btbd10 | 1.519 | 8.54E-09 | 4.77E-08 |
| Sybu | 4.467 | 8.64E-09 | 4.83E-08 |
| Ptgfrn | 1.154 | 9.04E-09 | 5.05E-08 |
| Zfp324 | 1.213 | 9.40E-09 | 5.25E-08 |
| Rbis | 1.175 | 9.70E-09 | 5.40E-08 |
| Sptb | -1.555 | 9.81E-09 | 5.46E-08 |
| Cacna1s | -5.954 | 1.01E-08 | 5.64E-08 |
| Irak1bp1 | 2.159 | 1.05E-08 | 5.83E-08 |
| Cmya5 | -6.451 | 1.12E-08 | 6.23E-08 |
| Abracl | 1.066 | 1.13E-08 | 6.28E-08 |
| Eef1akmt4 | 1.338 | 1.15E-08 | 6.39E-08 |
| Rfx4 | -2.410 | 1.17E-08 | 6.50E-08 |
| Dclre1a | 1.044 | 1.30E-08 | 7.14E-08 |
| Srfbp1 | 1.270 | 1.32E-08 | 7.27E-08 |
| Ndufaf4 | 1.008 | 1.35E-08 | 7.41E-08 |
| Thoc1 | 1.164 | 1.40E-08 | 7.69E-08 |
| Scaf11 | 1.033 | 1.46E-08 | 7.99E-08 |
| Hltf | 1.376 | 1.48E-08 | 8.11E-08 |
| Arfip2 | 1.010 | 1.48E-08 | 8.11E-08 |
| Sh2b2 | 1.393 | 1.58E-08 | 8.60E-08 |
| 1810009J06Rik | -9.926 | 1.58E-08 | 8.64E-08 |
| Dus4l | 1.399 | 1.59E-08 | 8.69E-08 |
| Ldb3 | -4.501 | 1.67E-08 | 9.07E-08 |
| Cilp | -2.433 | 1.70E-08 | 9.26E-08 |
| Casq1 | -6.037 | 1.72E-08 | 9.35E-08 |
| Adam8 | 2.707 | 1.73E-08 | 9.39E-08 |
| Fbxo30 | 1.095 | 1.79E-08 | 9.73E-08 |
| Hnrnpa3 | 1.048 | 1.90E-08 | 1.03E-07 |
| Nphp1 | 1.754 | 2.09E-08 | 1.13E-07 |
| Ccdc66 | 1.839 | 2.12E-08 | 1.14E-07 |
| Snrpa1 | 1.068 | 2.15E-08 | 1.16E-07 |
| Siah1a | 1.299 | 2.16E-08 | 1.17E-07 |
| Plk1 | 1.660 | 2.20E-08 | 1.19E-07 |
| Cdt1 | 1.716 | 2.23E-08 | 1.20E-07 |
| Reg2 | -5.976 | 2.23E-08 | 1.20E-07 |
| Pigm | 1.035 | 2.23E-08 | 1.20E-07 |
| Pfn2 | 1.359 | 2.25E-08 | 1.21E-07 |
| Capn6 | 1.861 | 2.30E-08 | 1.23E-07 |
| Rnf2 | 1.145 | 2.32E-08 | 1.25E-07 |
| Tsr3 | 1.069 | 2.47E-08 | 1.33E-07 |
| Zfp386 | 1.530 | 2.51E-08 | 1.35E-07 |
| Lyar | 1.253 | 2.57E-08 | 1.38E-07 |
| Gins2 | 1.565 | 2.65E-08 | 1.42E-07 |
| Fntb | 1.655 | 2.72E-08 | 1.45E-07 |
| Cd14 | 1.356 | 2.85E-08 | 1.52E-07 |
| Dhps | 1.048 | 2.97E-08 | 1.58E-07 |
| Zbtb10 | 1.506 | 3.19E-08 | 1.70E-07 |
| Dusp4 | 1.655 | 3.20E-08 | 1.70E-07 |
| Scn8a | 2.682 | 3.39E-08 | 1.80E-07 |
| Nrf1 | 1.033 | 3.46E-08 | 1.83E-07 |
| Racgap1 | 2.376 | 3.51E-08 | 1.86E-07 |
| Urb1 | 1.149 | 3.68E-08 | 1.94E-07 |
| Lysmd3 | 1.535 | 3.69E-08 | 1.95E-07 |
| Dimt1 | 1.707 | 3.89E-08 | 2.05E-07 |
| Igfbp6 | -1.105 | 4.06E-08 | 2.13E-07 |
| Stx1b | -1.991 | 4.18E-08 | 2.19E-07 |
| Psmg2 | 1.055 | 4.21E-08 | 2.21E-07 |
| Pcdhgc4 | -4.778 | 4.25E-08 | 2.23E-07 |
| Foxn2 | 1.160 | 4.28E-08 | 2.24E-07 |
| Pwwp2a | 1.581 | 4.62E-08 | 2.42E-07 |
| Fry | 1.051 | 4.70E-08 | 2.45E-07 |
| Atr | 1.530 | 5.03E-08 | 2.62E-07 |
| Tbc1d8b | 1.296 | 5.16E-08 | 2.68E-07 |
| Slc15a5 | -1.477 | 5.34E-08 | 2.78E-07 |
| Timp1 | 3.624 | 5.35E-08 | 2.78E-07 |
| Scn4b | -6.877 | 5.60E-08 | 2.90E-07 |
| Fignl1 | 3.807 | 5.76E-08 | 2.98E-07 |
| S100a9 | 1.737 | 5.87E-08 | 3.04E-07 |
| Cdkn2aip | 1.144 | 5.97E-08 | 3.08E-07 |
| Fam3a | 1.027 | 6.04E-08 | 3.12E-07 |
| Ucp1 | -8.644 | 6.12E-08 | 3.16E-07 |
| Nrg1 | 2.900 | 6.12E-08 | 3.16E-07 |
| Alkbh1 | 1.291 | 6.13E-08 | 3.16E-07 |
| Fhod3 | -1.451 | 6.24E-08 | 3.22E-07 |
| Akr1b7 | 1.548 | 6.25E-08 | 3.22E-07 |
| Mmp28 | 2.268 | 7.06E-08 | 3.62E-07 |
| Alkbh2 | 1.259 | 7.61E-08 | 3.90E-07 |
| Mtrr | 1.067 | 7.64E-08 | 3.91E-07 |
| Slc1a4 | 1.080 | 7.64E-08 | 3.91E-07 |
| Cdk5rap1 | 1.164 | 7.77E-08 | 3.97E-07 |
| Ckap2l | 2.746 | 7.85E-08 | 4.01E-07 |
| Telo2 | 1.201 | 8.11E-08 | 4.14E-07 |
| Utp14b | 1.585 | 8.12E-08 | 4.14E-07 |
| Sult1c2 | -1.245 | 8.23E-08 | 4.19E-07 |
| Atp6v0d2 | 2.388 | 8.47E-08 | 4.30E-07 |
| Myl2 | -9.723 | 8.89E-08 | 4.51E-07 |
| Fanca | 2.322 | 9.06E-08 | 4.60E-07 |
| Katnal1 | 1.316 | 9.25E-08 | 4.69E-07 |
| Peg3 | 1.369 | 9.30E-08 | 4.72E-07 |
| Rgs4 | -1.787 | 9.47E-08 | 4.79E-07 |
| Cd2ap | 1.227 | 9.69E-08 | 4.90E-07 |
| Eef2kmt | 1.043 | 9.78E-08 | 4.94E-07 |
| Myl3 | -9.293 | 1.01E-07 | 5.07E-07 |
| Appl1 | 1.222 | 1.01E-07 | 5.11E-07 |
| Cdca3 | 1.818 | 1.05E-07 | 5.26E-07 |
| Lgals4 | -1.414 | 1.05E-07 | 5.28E-07 |
| Sytl1 | 1.447 | 1.08E-07 | 5.41E-07 |
| Ccnb2 | 2.432 | 1.08E-07 | 5.42E-07 |
| Rock2 | 2.099 | 1.11E-07 | 5.57E-07 |
| Nsun3 | 1.041 | 1.15E-07 | 5.75E-07 |
| BC048403 | 1.122 | 1.18E-07 | 5.88E-07 |
| Rundc3b | 2.661 | 1.18E-07 | 5.88E-07 |
| Slpi | 1.708 | 1.20E-07 | 5.98E-07 |
| Elfn1 | 7.662 | 1.25E-07 | 6.21E-07 |
| Dnajc12 | 1.020 | 1.36E-07 | 6.73E-07 |
| Armcx5 | 1.528 | 1.45E-07 | 7.19E-07 |
| Gtpbp8 | 1.142 | 1.45E-07 | 7.19E-07 |
| Gtdc1 | 1.173 | 1.45E-07 | 7.19E-07 |
| Atp2b2 | 1.380 | 1.47E-07 | 7.26E-07 |
| Zfp142 | 1.038 | 1.48E-07 | 7.31E-07 |
| Tmem182 | -7.589 | 1.52E-07 | 7.52E-07 |
| Lepr | 1.863 | 1.57E-07 | 7.71E-07 |
| Scg2 | -6.061 | 1.63E-07 | 8.02E-07 |
| Ipo7 | 1.105 | 1.65E-07 | 8.10E-07 |
| Thg1l | 1.399 | 1.65E-07 | 8.13E-07 |
| Srp54b | 2.684 | 1.69E-07 | 8.30E-07 |
| Scnm1 | 1.041 | 1.82E-07 | 8.92E-07 |
| Ier5 | 1.009 | 1.84E-07 | 9.02E-07 |
| Marveld3 | 2.005 | 1.85E-07 | 9.06E-07 |
| Gm5771 | -9.477 | 1.91E-07 | 9.34E-07 |
| Btaf1 | 1.079 | 1.95E-07 | 9.54E-07 |
| Cdk1 | 1.498 | 1.95E-07 | 9.54E-07 |
| Abr | 1.100 | 1.96E-07 | 9.57E-07 |
| Mnat1 | 1.394 | 2.02E-07 | 9.84E-07 |
| Slc10a7 | 1.085 | 2.13E-07 | 1.03E-06 |
| Klk1b4 | -1.494 | 2.33E-07 | 1.13E-06 |
| Stk11ip | 1.128 | 2.34E-07 | 1.13E-06 |
| Aptx | 1.187 | 2.44E-07 | 1.18E-06 |
| Nemf | 1.209 | 2.51E-07 | 1.21E-06 |
| Dbh | -7.731 | 2.65E-07 | 1.28E-06 |
| Snx16 | 1.318 | 2.66E-07 | 1.28E-06 |
| Gdf9 | 2.025 | 2.74E-07 | 1.32E-06 |
| Agap1 | 1.107 | 2.81E-07 | 1.35E-06 |
| Mtbp | 2.188 | 2.89E-07 | 1.39E-06 |
| Slc5a6 | 1.396 | 2.94E-07 | 1.41E-06 |
| Ring1 | 1.153 | 2.99E-07 | 1.43E-06 |
| Prmt1 | 1.026 | 3.00E-07 | 1.44E-06 |
| Zfyve16 | 1.341 | 3.05E-07 | 1.46E-06 |
| N6amt1 | 1.238 | 3.09E-07 | 1.48E-06 |
| Ptpn2 | 1.041 | 3.11E-07 | 1.49E-06 |
| Copz2 | 1.081 | 3.16E-07 | 1.51E-06 |
| Hgh1 | 1.248 | 3.20E-07 | 1.52E-06 |
| Zfp51 | 1.815 | 3.23E-07 | 1.54E-06 |
| Tle6 | -1.157 | 3.25E-07 | 1.55E-06 |
| Map3k6 | 1.686 | 3.37E-07 | 1.60E-06 |
| Wee1 | 1.114 | 3.41E-07 | 1.62E-06 |
| Rbmx | 1.030 | 3.47E-07 | 1.64E-06 |
| Krt19 | -1.003 | 3.55E-07 | 1.68E-06 |
| Ofd1 | 1.530 | 3.60E-07 | 1.70E-06 |
| Chchd6 | 1.555 | 3.61E-07 | 1.70E-06 |
| Zfp37 | 3.066 | 3.65E-07 | 1.72E-06 |
| Myot | -2.827 | 3.78E-07 | 1.78E-06 |
| Mettl4 | 1.234 | 3.81E-07 | 1.79E-06 |
| Kif2c | 3.252 | 3.90E-07 | 1.84E-06 |
| Chil1 | 2.807 | 3.92E-07 | 1.84E-06 |
| Bcl6b | -1.096 | 3.95E-07 | 1.86E-06 |
| Plekhm3 | 1.354 | 4.19E-07 | 1.96E-06 |
| Gtpbp3 | 1.023 | 4.64E-07 | 2.17E-06 |
| Acpp | 1.245 | 4.78E-07 | 2.23E-06 |
| Adcy1 | 1.791 | 4.97E-07 | 2.32E-06 |
| Fut1 | 4.934 | 5.07E-07 | 2.36E-06 |
| Lnpk | 1.162 | 5.23E-07 | 2.43E-06 |
| Il23a | 6.103 | 5.46E-07 | 2.53E-06 |
| Mgarp | -8.617 | 5.47E-07 | 2.54E-06 |
| Tbc1d19 | 1.719 | 5.58E-07 | 2.58E-06 |
| Prrg4 | 2.469 | 5.63E-07 | 2.60E-06 |
| Stap1 | 3.333 | 6.10E-07 | 2.81E-06 |
| Meiob | 1.453 | 6.10E-07 | 2.81E-06 |
| Pick1 | 1.027 | 6.27E-07 | 2.88E-06 |
| Dhx34 | 1.012 | 6.29E-07 | 2.89E-06 |
| Slc39a13 | 1.098 | 6.29E-07 | 2.89E-06 |
| Clca3a1 | 1.128 | 6.64E-07 | 3.04E-06 |
| Duox2 | -2.360 | 6.70E-07 | 3.07E-06 |
| Psph | 1.078 | 6.71E-07 | 3.07E-06 |
| Cabyr | -1.322 | 6.91E-07 | 3.16E-06 |
| Zrsr2 | 1.492 | 6.99E-07 | 3.20E-06 |
| Bco1 | 1.085 | 7.11E-07 | 3.25E-06 |
| Kdelr3 | 2.357 | 7.38E-07 | 3.37E-06 |
| Pde4d | 1.391 | 7.53E-07 | 3.43E-06 |
| Mybpc2 | -2.692 | 7.61E-07 | 3.46E-06 |
| Ces2b | -3.256 | 8.22E-07 | 3.74E-06 |
| Sgms1 | 1.059 | 8.60E-07 | 3.89E-06 |
| Ccl27a | -1.057 | 8.88E-07 | 4.02E-06 |
| Cdk20 | 1.114 | 9.12E-07 | 4.12E-06 |
| H2-T24 | -1.154 | 9.14E-07 | 4.13E-06 |
| Abitram | 1.197 | 9.46E-07 | 4.26E-06 |
| Xlr3a | 1.204 | 9.53E-07 | 4.29E-06 |
| Phgdh | 1.498 | 9.53E-07 | 4.29E-06 |
| Chil4 | -8.662 | 9.53E-07 | 4.29E-06 |
| Wnt5a | 1.576 | 9.60E-07 | 4.32E-06 |
| Smarcad1 | 1.397 | 9.77E-07 | 4.38E-06 |
| Scand1 | 1.064 | 9.96E-07 | 4.46E-06 |
| Cdr2 | 1.692 | 1.00E-06 | 4.48E-06 |
| Zswim7 | 1.141 | 1.06E-06 | 4.75E-06 |
| Ltbp2 | 1.155 | 1.07E-06 | 4.79E-06 |
| Cmss1 | 1.592 | 1.13E-06 | 5.01E-06 |
| Mki67 | 2.322 | 1.17E-06 | 5.19E-06 |
| Slc22a27 | 2.108 | 1.17E-06 | 5.20E-06 |
| Emp1 | 1.279 | 1.17E-06 | 5.21E-06 |
| Uty | 1.053 | 1.32E-06 | 5.84E-06 |
| Asic5 | 1.482 | 1.32E-06 | 5.84E-06 |
| Pus7l | 1.811 | 1.37E-06 | 6.07E-06 |
| Trnt1 | 1.089 | 1.49E-06 | 6.54E-06 |
| Ssb | 1.105 | 1.57E-06 | 6.90E-06 |
| Dmac2l | 1.261 | 1.61E-06 | 7.08E-06 |
| Smad7 | 1.286 | 1.71E-06 | 7.50E-06 |
| Xrcc4 | 1.239 | 1.71E-06 | 7.50E-06 |
| Celsr2 | -1.652 | 1.75E-06 | 7.65E-06 |
| Abce1 | 1.335 | 1.79E-06 | 7.82E-06 |
| Amn1 | 1.537 | 1.89E-06 | 8.23E-06 |
| Ddx31 | 1.018 | 1.97E-06 | 8.57E-06 |
| Nup43 | 1.293 | 2.01E-06 | 8.70E-06 |
| Tspyl4 | 1.170 | 2.07E-06 | 8.98E-06 |
| Cxcl5 | -3.447 | 2.09E-06 | 9.05E-06 |
| Sntg2 | -1.128 | 2.09E-06 | 9.06E-06 |
| Ermard | 1.137 | 2.15E-06 | 9.29E-06 |
| C1galt1 | 1.020 | 2.26E-06 | 9.73E-06 |
| Sh3bp4 | 1.208 | 2.28E-06 | 9.83E-06 |
| Nup37 | 1.301 | 2.34E-06 | 1.01E-05 |
| Sdr39u1 | 1.117 | 2.34E-06 | 1.01E-05 |
| Yae1d1 | 1.268 | 2.42E-06 | 1.04E-05 |
| Adrb3 | -2.170 | 2.47E-06 | 1.06E-05 |
| Rpap3 | 1.104 | 2.50E-06 | 1.07E-05 |
| Ascc3 | 1.556 | 2.55E-06 | 1.09E-05 |
| Tango6 | 1.020 | 2.64E-06 | 1.13E-05 |
| Tut4 | 1.105 | 2.65E-06 | 1.13E-05 |
| Cntfr | -1.040 | 2.72E-06 | 1.16E-05 |
| Wars2 | 1.130 | 2.78E-06 | 1.19E-05 |
| Pkp1 | 4.248 | 2.81E-06 | 1.20E-05 |
| Dppa2 | 8.050 | 2.94E-06 | 1.25E-05 |
| Pbrm1 | 1.001 | 2.98E-06 | 1.27E-05 |
| Eif5b | 1.051 | 3.10E-06 | 1.32E-05 |
| Get1 | 1.070 | 3.21E-06 | 1.36E-05 |
| Abcd2 | 1.162 | 3.22E-06 | 1.37E-05 |
| Rnpc3 | 1.276 | 3.26E-06 | 1.38E-05 |
| Adam12 | 2.046 | 3.36E-06 | 1.42E-05 |
| Fen1 | 1.268 | 3.42E-06 | 1.45E-05 |
| Foxred2 | 2.722 | 3.43E-06 | 1.45E-05 |
| Fbxo36 | 1.258 | 3.48E-06 | 1.47E-05 |
| Cdc6 | 3.939 | 3.55E-06 | 1.50E-05 |
| Lrif1 | 1.206 | 3.65E-06 | 1.54E-05 |
| Pycr1 | 2.069 | 3.66E-06 | 1.54E-05 |
| Exoc3l | -1.105 | 3.67E-06 | 1.55E-05 |
| Alkbh6 | 1.108 | 3.73E-06 | 1.57E-05 |
| Pclo | -1.492 | 3.83E-06 | 1.61E-05 |
| Hspbap1 | 1.321 | 4.24E-06 | 1.78E-05 |
| Pcbp3 | 1.925 | 4.46E-06 | 1.86E-05 |
| Rad1 | 1.062 | 4.47E-06 | 1.87E-05 |
| Ccl5 | -1.509 | 4.47E-06 | 1.87E-05 |
| Pgam2 | -4.958 | 4.53E-06 | 1.89E-05 |
| Tlr13 | 1.521 | 4.64E-06 | 1.94E-05 |
| Prom1 | -1.273 | 4.69E-06 | 1.96E-05 |
| Tmco6 | 1.188 | 4.95E-06 | 2.06E-05 |
| Ggct | 1.033 | 5.03E-06 | 2.09E-05 |
| Pla2g1b | -9.684 | 5.32E-06 | 2.20E-05 |
| Rpa2 | 1.031 | 5.47E-06 | 2.26E-05 |
| Naa15 | 1.116 | 5.55E-06 | 2.30E-05 |
| Zfp454 | -1.333 | 5.55E-06 | 2.30E-05 |
| Dbp | 1.536 | 5.62E-06 | 2.32E-05 |
| Gm10334 | -8.830 | 5.84E-06 | 2.41E-05 |
| Zfp719 | 1.293 | 5.85E-06 | 2.41E-05 |
| Slfn9 | 1.692 | 6.12E-06 | 2.52E-05 |
| Xpo1 | 1.030 | 6.30E-06 | 2.59E-05 |
| B4galnt2 | -3.054 | 6.38E-06 | 2.62E-05 |
| Ccne2 | 1.631 | 6.38E-06 | 2.62E-05 |
| Akr1cl | -7.679 | 6.46E-06 | 2.65E-05 |
| Poglut2 | 1.097 | 6.49E-06 | 2.66E-05 |
| Myoz1 | -5.209 | 6.50E-06 | 2.66E-05 |
| Birc5 | 2.125 | 7.10E-06 | 2.90E-05 |
| Prelid2 | 3.894 | 7.61E-06 | 3.10E-05 |
| Tspan8 | -1.459 | 7.67E-06 | 3.13E-05 |
| Rnaseh1 | 1.145 | 8.07E-06 | 3.28E-05 |
| Recql4 | 1.872 | 8.44E-06 | 3.42E-05 |
| Mettl17 | 1.072 | 8.48E-06 | 3.44E-05 |
| Morc3 | 1.078 | 8.68E-06 | 3.52E-05 |
| Gm17359 | 8.155 | 8.68E-06 | 3.52E-05 |
| Nupl2 | 1.259 | 8.82E-06 | 3.57E-05 |
| Lrrc15 | -2.632 | 8.87E-06 | 3.58E-05 |
| Pdia2 | -5.170 | 9.13E-06 | 3.68E-05 |
| Nkrf | 1.124 | 9.14E-06 | 3.69E-05 |
| Itsn2 | 1.012 | 9.44E-06 | 3.80E-05 |
| Plk4 | 2.307 | 9.49E-06 | 3.82E-05 |
| Rad51 | 2.425 | 9.72E-06 | 3.91E-05 |
| Rad51ap1 | 8.050 | 9.78E-06 | 3.93E-05 |
| Ppp3cc | 1.319 | 9.86E-06 | 3.96E-05 |
| Cables1 | 1.632 | 9.96E-06 | 4.00E-05 |
| Klhl41 | -3.365 | 1.01E-05 | 4.05E-05 |
| Tchh | 2.632 | 1.01E-05 | 4.06E-05 |
| Clk1 | 1.027 | 1.02E-05 | 4.08E-05 |
| Xirp2 | -5.714 | 1.02E-05 | 4.08E-05 |
| Fam184a | 1.154 | 1.07E-05 | 4.29E-05 |
| Rasl11b | -1.054 | 1.07E-05 | 4.29E-05 |
| Kif22 | 1.579 | 1.10E-05 | 4.40E-05 |
| Ckmt1 | -1.845 | 1.17E-05 | 4.63E-05 |
| Cd59b | -1.396 | 1.20E-05 | 4.77E-05 |
| Znrd2 | 1.098 | 1.21E-05 | 4.79E-05 |
| Mre11a | 1.989 | 1.25E-05 | 4.95E-05 |
| Ppp1r42 | 3.239 | 1.32E-05 | 5.21E-05 |
| Tmem35a | -5.000 | 1.35E-05 | 5.31E-05 |
| Gmds | 1.247 | 1.36E-05 | 5.35E-05 |
| Trip11 | 1.081 | 1.37E-05 | 5.42E-05 |
| Ogg1 | 1.301 | 1.39E-05 | 5.47E-05 |
| Dync2i2 | 1.129 | 1.44E-05 | 5.67E-05 |
| Tdrd3 | 1.145 | 1.48E-05 | 5.83E-05 |
| 3425401B19Rik | -5.907 | 1.51E-05 | 5.91E-05 |
| Cyp2a4 | 1.469 | 1.56E-05 | 6.10E-05 |
| Mphosph8 | 1.364 | 1.56E-05 | 6.11E-05 |
| Znhit6 | 1.300 | 1.56E-05 | 6.11E-05 |
| Tfpt | 1.024 | 1.58E-05 | 6.18E-05 |
| Zfp948 | 1.503 | 1.58E-05 | 6.19E-05 |
| Taf1a | 1.248 | 1.59E-05 | 6.21E-05 |
| Rnmt | 1.122 | 1.59E-05 | 6.22E-05 |
| Entpd7 | 1.119 | 1.61E-05 | 6.31E-05 |
| Zfx | 1.016 | 1.64E-05 | 6.40E-05 |
| Cd4 | -1.170 | 1.64E-05 | 6.42E-05 |
| Mad2l1 | 1.327 | 1.68E-05 | 6.54E-05 |
| Prpf40b | 1.123 | 1.68E-05 | 6.54E-05 |
| Rpl3l | -4.787 | 1.68E-05 | 6.54E-05 |
| Gpbp1 | 1.070 | 1.69E-05 | 6.58E-05 |
| Snrnp35 | 1.096 | 1.71E-05 | 6.65E-05 |
| Fbxw15 | 3.644 | 1.81E-05 | 7.04E-05 |
| Caap1 | 1.253 | 1.84E-05 | 7.12E-05 |
| Lratd1 | -1.235 | 1.84E-05 | 7.14E-05 |
| Ppp1r3g | 2.094 | 1.91E-05 | 7.41E-05 |
| 2700062C07Rik | 1.078 | 2.05E-05 | 7.89E-05 |
| Slc37a1 | 1.431 | 2.20E-05 | 8.44E-05 |
| Esf1 | 1.309 | 2.20E-05 | 8.44E-05 |
| Th | -7.845 | 2.21E-05 | 8.47E-05 |
| Taf5 | 1.102 | 2.25E-05 | 8.62E-05 |
| Smpx | -8.333 | 2.27E-05 | 8.70E-05 |
| Ccr1 | 2.443 | 2.29E-05 | 8.74E-05 |
| Smc2 | 1.530 | 2.33E-05 | 8.91E-05 |
| Bik | -1.197 | 2.34E-05 | 8.94E-05 |
| Ppm1h | 1.516 | 2.39E-05 | 9.12E-05 |
| Bclaf1 | 1.017 | 2.40E-05 | 9.14E-05 |
| Gal3st2 | -3.982 | 2.40E-05 | 9.16E-05 |
| Sypl2 | -5.322 | 2.52E-05 | 9.60E-05 |
| Il1rn | 1.119 | 2.55E-05 | 9.71E-05 |
| Matr3 | 1.007 | 2.59E-05 | 9.82E-05 |
| Shh | -2.841 | 2.59E-05 | 9.85E-05 |
| Marchf3 | 1.480 | 2.61E-05 | 9.90E-05 |
| Med31 | 1.375 | 2.65E-05 | 0.0001007 |
| Kcnk9 | -4.644 | 2.66E-05 | 0.000101 |
| Gm28043 | 1.032 | 2.79E-05 | 0.0001054 |
| Dnah1 | 4.644 | 2.83E-05 | 0.0001067 |
| Ptges | 2.921 | 2.88E-05 | 0.0001088 |
| Rps23rg1 | -1.655 | 3.21E-05 | 0.0001206 |
| Grhl1 | 1.446 | 3.23E-05 | 0.0001213 |
| C5ar1 | 1.577 | 3.25E-05 | 0.000122 |
| Cenpj | 1.499 | 3.28E-05 | 0.0001231 |
| Ckap2 | 2.599 | 3.28E-05 | 0.0001232 |
| Esco1 | 1.180 | 3.36E-05 | 0.0001257 |
| Tacc3 | 1.346 | 3.40E-05 | 0.0001271 |
| Zfp566 | 2.273 | 3.45E-05 | 0.0001289 |
| Napb | 1.751 | 3.52E-05 | 0.0001312 |
| Zzz3 | 1.050 | 3.70E-05 | 0.0001376 |
| Gm14295 | 1.539 | 3.73E-05 | 0.0001385 |
| Col6a3 | 1.169 | 3.80E-05 | 0.0001412 |
| Hrc | -4.600 | 4.03E-05 | 0.0001491 |
| Snhg11 | 1.841 | 4.04E-05 | 0.0001495 |
| Brd3os | 3.192 | 4.13E-05 | 0.0001527 |
| Casq2 | -2.212 | 4.18E-05 | 0.0001545 |
| Eml6 | 1.718 | 4.18E-05 | 0.0001545 |
| Abcc10 | 1.214 | 4.20E-05 | 0.0001552 |
| Hells | 1.283 | 4.22E-05 | 0.000156 |
| Eif4e3 | 1.025 | 4.24E-05 | 0.0001565 |
| Mcph1 | 1.350 | 4.47E-05 | 0.000165 |
| Synj2 | 1.269 | 4.63E-05 | 0.0001704 |
| Gabpb1 | 1.026 | 4.64E-05 | 0.0001707 |
| Myoz2 | -7.861 | 4.70E-05 | 0.0001729 |
| Smtnl1 | -7.322 | 4.72E-05 | 0.0001732 |
| Rbm48 | 1.078 | 4.77E-05 | 0.0001751 |
| Fmo3 | 3.369 | 5.02E-05 | 0.0001836 |
| Xrcc2 | 1.411 | 5.03E-05 | 0.0001842 |
| Igsf23 | 1.632 | 5.09E-05 | 0.0001863 |
| Gpx6 | -2.459 | 5.16E-05 | 0.0001885 |
| Smyd4 | 1.293 | 5.57E-05 | 0.000203 |
| Cwf19l2 | 1.131 | 5.61E-05 | 0.0002043 |
| Aoc3 | -1.695 | 5.61E-05 | 0.0002044 |
| Erc2 | 1.056 | 5.72E-05 | 0.000208 |
| Slc2a5 | 1.300 | 5.78E-05 | 0.0002101 |
| Mars2 | 1.058 | 6.14E-05 | 0.0002224 |
| Rpp30 | 1.103 | 6.31E-05 | 0.0002282 |
| Cep78 | 1.404 | 6.35E-05 | 0.0002298 |
| Ttll11 | 2.863 | 6.38E-05 | 0.0002308 |
| Actn3 | -3.000 | 6.42E-05 | 0.0002321 |
| Taf1d | 1.321 | 6.47E-05 | 0.0002336 |
| 4931428F04Rik | 1.741 | 6.54E-05 | 0.000236 |
| Aurkb | 2.450 | 6.58E-05 | 0.0002373 |
| 1810010H24Rik | 1.382 | 6.59E-05 | 0.0002375 |
| Slc25a32 | 1.005 | 6.68E-05 | 0.0002407 |
| Rev1 | 1.112 | 6.75E-05 | 0.0002428 |
| Ccdc30 | -1.061 | 6.78E-05 | 0.000244 |
| Zfp397 | 1.139 | 6.86E-05 | 0.0002467 |
| Spock2 | -1.123 | 6.98E-05 | 0.0002506 |
| 2210418O10Rik | 1.046 | 7.37E-05 | 0.000264 |
| Akap17b | 1.371 | 7.50E-05 | 0.0002685 |
| Ppp1r7 | 1.322 | 7.62E-05 | 0.0002725 |
| Trhde | -1.907 | 7.67E-05 | 0.0002742 |
| Srd5a2 | -1.051 | 7.83E-05 | 0.0002794 |
| Clic6 | -3.196 | 7.89E-05 | 0.0002813 |
| Chek1 | 2.158 | 7.90E-05 | 0.0002818 |
| Apobec2 | -7.570 | 7.91E-05 | 0.0002819 |
| Cwc22 | 1.047 | 8.10E-05 | 0.0002883 |
| Klhl31 | -4.459 | 8.19E-05 | 0.0002913 |
| Klf6 | 1.003 | 8.52E-05 | 0.0003021 |
| Gabrp | -2.865 | 8.92E-05 | 0.0003156 |
| Cd3eap | 1.005 | 8.99E-05 | 0.0003179 |
| H4c8 | 1.446 | 9.11E-05 | 0.000322 |
| Pard6a | 1.583 | 9.21E-05 | 0.0003254 |
| Clec4d | 3.047 | 9.49E-05 | 0.0003344 |
| Dtx1 | -1.415 | 9.59E-05 | 0.0003377 |
| Mcf2l | 2.200 | 9.62E-05 | 0.0003387 |
| Il33 | 1.272 | 9.68E-05 | 0.0003407 |
| Cyp2s1 | -1.554 | 9.71E-05 | 0.0003416 |
| S100a8 | 1.769 | 0.000102 | 0.0003573 |
| Shq1 | 1.002 | 0.000102 | 0.0003578 |
| Mipol1 | 1.435 | 0.000104 | 0.0003658 |
| Zfp608 | 1.302 | 0.000106 | 0.0003721 |
| Zfp13 | 1.981 | 0.000111 | 0.0003865 |
| F830016B08Rik | -1.154 | 0.000111 | 0.0003868 |
| Thbs1 | 1.059 | 0.000111 | 0.0003881 |
| Esrrg | -1.630 | 0.000112 | 0.0003917 |
| Slc6a16 | -2.769 | 0.000113 | 0.0003939 |
| Prpf4b | 1.404 | 0.000123 | 0.0004253 |
| Nsun6 | 1.416 | 0.000123 | 0.0004255 |
| Apol7c | -2.837 | 0.000123 | 0.0004269 |
| Cip2a | 2.105 | 0.000126 | 0.0004372 |
| Rasl2-9 | -3.553 | 0.000128 | 0.000442 |
| Kntc1 | 2.459 | 0.000135 | 0.000467 |
| Mtrf1 | 1.005 | 0.000138 | 0.0004761 |
| Gpx2 | 2.178 | 0.000138 | 0.0004771 |
| Kcnk3 | -1.788 | 0.000141 | 0.0004864 |
| St8sia3 | 2.371 | 0.000142 | 0.0004887 |
| Rps6ka3 | 1.063 | 0.000145 | 0.0004995 |
| Nrip2 | 2.418 | 0.000149 | 0.000512 |
| Pinx1 | 1.201 | 0.00015 | 0.0005133 |
| Wnt5b | 1.018 | 0.00015 | 0.0005134 |
| Cep126 | 3.722 | 0.000152 | 0.0005202 |
| Rif1 | 1.336 | 0.000155 | 0.0005301 |
| Setx | 1.029 | 0.000156 | 0.0005324 |
| Myo5a | 2.126 | 0.000157 | 0.0005345 |
| Csde1 | 1.088 | 0.000157 | 0.0005371 |
| Slc22a4 | 1.364 | 0.000159 | 0.0005428 |
| Scn4a | -4.322 | 0.00016 | 0.0005469 |
| Trbc2 | -1.569 | 0.000172 | 0.0005845 |
| Prkn | 1.580 | 0.000175 | 0.0005923 |
| Gpx7 | 1.314 | 0.000176 | 0.0005964 |
| Art1 | -7.322 | 0.000181 | 0.0006102 |
| Ltb | -1.296 | 0.000181 | 0.0006102 |
| Zfp119a | 1.392 | 0.000184 | 0.00062 |
| Amy2a5 | -13.483 | 0.000184 | 0.0006202 |
| Perm1 | -2.518 | 0.000192 | 0.0006445 |
| Arfgef3 | 2.874 | 0.000198 | 0.0006639 |
| Dtl | 2.293 | 0.000205 | 0.000687 |
| Zfp800 | 1.222 | 0.000209 | 0.0006987 |
| Lamb3 | 1.163 | 0.00021 | 0.0007009 |
| Bcdin3d | 1.348 | 0.000215 | 0.0007166 |
| E130311K13Rik | 1.056 | 0.000217 | 0.0007226 |
| Ktn1 | 1.049 | 0.000223 | 0.0007431 |
| Trdc | 3.931 | 0.000226 | 0.00075 |
| Ptrh1 | 1.793 | 0.000234 | 0.000776 |
| Ifi207 | 1.031 | 0.000238 | 0.0007901 |
| Prkab2 | 1.050 | 0.00024 | 0.0007949 |
| Cuzd1 | -7.229 | 0.000249 | 0.0008211 |
| Pdzk1ip1 | -1.356 | 0.000253 | 0.0008337 |
| Lrp2 | -1.104 | 0.000254 | 0.0008366 |
| Fam241b | 2.434 | 0.000268 | 0.00088 |
| Plaur | 1.665 | 0.000268 | 0.000881 |
| Ncapg | 2.824 | 0.00027 | 0.0008864 |
| Rbm26 | 1.032 | 0.000279 | 0.0009135 |
| Mup10 | -1.012 | 0.000279 | 0.0009135 |
| Cyp46a1 | -1.637 | 0.000279 | 0.0009142 |
| Alox5ap | 1.129 | 0.000279 | 0.000915 |
| Ly6c2 | -1.178 | 0.000281 | 0.0009195 |
| Tfap4 | 1.035 | 0.000283 | 0.000925 |
| Tyro3 | 1.644 | 0.000283 | 0.0009261 |
| Gm826 | -1.837 | 0.000295 | 0.000962 |
| Ppig | 1.068 | 0.000296 | 0.0009658 |
| Ccne1 | 1.400 | 0.000297 | 0.000969 |
| Tnnt1 | -1.317 | 0.000297 | 0.0009698 |
| Aunip | 5.011 | 0.000303 | 0.0009872 |
| Catsper2 | 1.613 | 0.00031 | 0.0010081 |
| Larp4 | 1.120 | 0.00031 | 0.0010085 |
| Bhlhb9 | 1.351 | 0.00032 | 0.0010377 |
| 3110001I22Rik | 1.501 | 0.000321 | 0.0010397 |
| Rnf225 | 2.389 | 0.000343 | 0.0011039 |
| 2700081O15Rik | -1.105 | 0.000344 | 0.0011069 |
| Rpgr | 1.592 | 0.000349 | 0.0011236 |
| Dlk1 | -4.824 | 0.000354 | 0.0011369 |
| Vcan | 3.138 | 0.000365 | 0.0011711 |
| Zfp691 | 1.615 | 0.000371 | 0.0011908 |
| Vcpkmt | 1.499 | 0.000378 | 0.0012126 |
| Muc4 | -2.000 | 0.000387 | 0.0012366 |
| Lzts1 | 2.963 | 0.000389 | 0.0012425 |
| Lrtm2 | 2.982 | 0.000393 | 0.0012539 |
| Kif4 | 2.209 | 0.000398 | 0.0012702 |
| Coa4 | 1.095 | 0.000401 | 0.0012786 |
| Piwil2 | 5.000 | 0.000402 | 0.0012822 |
| Amn | 4.923 | 0.000405 | 0.0012918 |
| Whrn | -1.222 | 0.000407 | 0.0012959 |
| Ak1 | -1.815 | 0.00041 | 0.0013058 |
| H4c18 | 9.388 | 0.000411 | 0.0013091 |
| Parm1 | -1.271 | 0.000414 | 0.0013164 |
| Ttk | 3.121 | 0.000416 | 0.0013223 |
| Card11 | -1.354 | 0.000428 | 0.0013576 |
| Six5 | 1.144 | 0.00043 | 0.0013639 |
| Trmt13 | 1.493 | 0.000433 | 0.0013725 |
| Ptpdc1 | 1.174 | 0.000437 | 0.0013848 |
| Gbp2b | -1.153 | 0.000438 | 0.0013861 |
| Dpep2 | 4.140 | 0.000438 | 0.0013861 |
| Ocstamp | 1.339 | 0.00045 | 0.0014235 |
| Slc26a2 | 1.026 | 0.000455 | 0.0014382 |
| Armcx6 | -1.501 | 0.000457 | 0.0014438 |
| Scimp | -1.337 | 0.000469 | 0.0014787 |
| Pf4 | 2.037 | 0.00047 | 0.0014804 |
| Synpo2 | -1.132 | 0.000472 | 0.0014852 |
| Fmn1 | 1.046 | 0.000473 | 0.001489 |
| Spag5 | 2.151 | 0.000481 | 0.0015107 |
| Flnc | -1.222 | 0.000483 | 0.001517 |
| Hspa1a | 1.113 | 0.000485 | 0.0015229 |
| Slc26a3 | -2.619 | 0.000492 | 0.0015429 |
| Tff2 | -2.763 | 0.000502 | 0.001573 |
| G6pc3 | 1.086 | 0.000512 | 0.0016015 |
| Trem2 | 1.431 | 0.000516 | 0.0016153 |
| Vps50 | 1.025 | 0.000521 | 0.0016288 |
| Melk | 2.933 | 0.000524 | 0.0016368 |
| Ntrk1 | -2.503 | 0.000533 | 0.0016623 |
| Sptssb | -6.845 | 0.000561 | 0.0017465 |
| Plk5 | -1.099 | 0.000587 | 0.0018234 |
| Ms4a4b | -1.785 | 0.000614 | 0.0018988 |
| Yme1l1 | 1.108 | 0.000619 | 0.0019149 |
| Bclaf3 | 1.355 | 0.000622 | 0.0019225 |
| Bag2 | 1.023 | 0.000635 | 0.0019598 |
| Anxa13 | -1.171 | 0.000638 | 0.0019665 |
| Exo1 | 2.609 | 0.00064 | 0.0019719 |
| Pde5a | 1.346 | 0.000641 | 0.0019766 |
| Sln | -7.276 | 0.000641 | 0.0019766 |
| Scaper | 1.121 | 0.000644 | 0.0019836 |
| Aqp4 | 1.449 | 0.000645 | 0.0019856 |
| Kif18b | 3.059 | 0.000656 | 0.002017 |
| Wdr25 | 1.486 | 0.000656 | 0.0020177 |
| Jsrp1 | -7.472 | 0.000662 | 0.0020347 |
| Ccdc189 | -2.000 | 0.000686 | 0.0021012 |
| Adat2 | 1.090 | 0.000688 | 0.0021066 |
| Nuf2 | 1.917 | 0.000701 | 0.0021445 |
| Cdca2 | 1.841 | 0.000719 | 0.002193 |
| Fermt1 | 1.713 | 0.000722 | 0.0022018 |
| Slc35f2 | 4.304 | 0.000744 | 0.002264 |
| Mmp7 | -1.228 | 0.00076 | 0.0023066 |
| Sult2a7 | 1.766 | 0.000771 | 0.0023384 |
| Atp10b | -3.248 | 0.000775 | 0.0023481 |
| Ctsj | 7.679 | 0.000788 | 0.0023859 |
| Cacna1h | -4.087 | 0.000792 | 0.0023986 |
| Brca1 | 1.569 | 0.000793 | 0.0023991 |
| Cfap300 | 3.513 | 0.000841 | 0.0025382 |
| Siva1 | 1.004 | 0.000842 | 0.0025421 |
| Cxcr6 | -1.190 | 0.000873 | 0.0026268 |
| Cckar | -6.022 | 0.000927 | 0.0027789 |
| Thbs4 | -2.047 | 0.000931 | 0.0027911 |
| Mbp | -1.469 | 0.001003 | 0.0029969 |
| Mms22l | 1.122 | 0.001008 | 0.0030101 |
| Slc51b | 2.359 | 0.001039 | 0.0030975 |
| Mylk4 | -5.229 | 0.001066 | 0.0031703 |
| Mtnr1a | -1.289 | 0.001076 | 0.0032007 |
| AU041133 | 1.367 | 0.001106 | 0.0032815 |
| Kif3a | 1.070 | 0.001129 | 0.0033423 |
| Myo18b | -1.926 | 0.001156 | 0.0034172 |
| Hsp90aa1 | 1.109 | 0.001166 | 0.0034424 |
| Myom2 | -5.022 | 0.001181 | 0.0034829 |
| Dnajc17 | 1.087 | 0.001197 | 0.0035247 |
| Olfm4 | -1.771 | 0.001197 | 0.0035262 |
| Adamts12 | 1.854 | 0.001198 | 0.0035284 |
| Mdc1 | 1.118 | 0.0012 | 0.0035314 |
| Trpm5 | -5.322 | 0.001235 | 0.0036276 |
| Zfp879 | -1.958 | 0.001238 | 0.0036371 |
| Glb1l2 | -4.369 | 0.001246 | 0.0036589 |
| Trim54 | -6.994 | 0.001262 | 0.0037001 |
| Myo15b | -1.840 | 0.001266 | 0.0037109 |
| Tshz3 | -1.109 | 0.001315 | 0.0038402 |
| Cdpf1 | 1.004 | 0.001335 | 0.0038941 |
| Bub1b | 1.347 | 0.001378 | 0.0040128 |
| Sh3pxd2b | 1.152 | 0.001391 | 0.0040498 |
| Nos1ap | 1.201 | 0.001399 | 0.0040679 |
| Myzap | -1.103 | 0.001412 | 0.0041007 |
| Clspn | 2.778 | 0.001417 | 0.0041129 |
| Gm37988 | -1.687 | 0.001459 | 0.0042276 |
| Txlnb | -3.755 | 0.001462 | 0.0042361 |
| Zbtb37 | 1.559 | 0.001491 | 0.0043159 |
| Flt3l | 1.180 | 0.001508 | 0.0043626 |
| Car9 | -1.719 | 0.00154 | 0.0044494 |
| Rab11fip5 | 1.150 | 0.001546 | 0.0044641 |
| Ms4a4a | 4.700 | 0.001583 | 0.0045618 |
| Cdkn3 | 3.072 | 0.001591 | 0.004581 |
| Cbln3 | -2.000 | 0.001591 | 0.0045816 |
| Sh2d7 | -5.907 | 0.001596 | 0.0045942 |
| Itprip | 1.120 | 0.001654 | 0.0047466 |
| Trdn | -6.229 | 0.001655 | 0.0047475 |
| Cxcl14 | 1.385 | 0.001657 | 0.0047528 |
| Gm45871 | 1.274 | 0.001683 | 0.0048212 |
| Cbr3 | 1.202 | 0.001733 | 0.0049553 |
| Pask | 1.445 | 0.001735 | 0.0049602 |
| Skap1 | -2.046 | 0.001747 | 0.0049916 |
| Btbd19 | 2.360 | 0.001765 | 0.0050385 |
| Haus6 | 1.344 | 0.001855 | 0.0052851 |
| Grap2 | -1.250 | 0.001883 | 0.0053538 |
| Tiparp | 1.040 | 0.001894 | 0.0053803 |
| Pdgfrl | -1.346 | 0.001909 | 0.0054198 |
| Ehf | -1.615 | 0.001952 | 0.0055348 |
| Ranbp2 | 1.237 | 0.001954 | 0.0055386 |
| Pcgf1 | 1.114 | 0.001979 | 0.005605 |
| Cenpi | 3.075 | 0.00201 | 0.0056828 |
| Cnmd | -1.841 | 0.002027 | 0.0057253 |
| Schip1 | -1.134 | 0.002041 | 0.0057578 |
| Ncapg2 | 1.968 | 0.002052 | 0.0057869 |
| Upf3b | 1.069 | 0.002055 | 0.0057908 |
| Zbtb45 | 1.331 | 0.002078 | 0.0058518 |
| Hoxb9 | -6.022 | 0.002087 | 0.0058764 |
| Retn | -3.392 | 0.002097 | 0.0059003 |
| Timeless | 1.089 | 0.002108 | 0.0059275 |
| Rb1cc1 | 1.338 | 0.002114 | 0.0059417 |
| Kctd13 | 1.332 | 0.002126 | 0.0059728 |
| Rtn2 | -1.534 | 0.002167 | 0.006079 |
| Afp | 2.252 | 0.002175 | 0.0060998 |
| Slc4a3 | -2.285 | 0.002183 | 0.0061192 |
| Ccl22 | -2.322 | 0.002208 | 0.0061839 |
| Vgll2 | -6.570 | 0.002254 | 0.0063021 |
| Tmem54 | -3.945 | 0.002257 | 0.0063099 |
| Zfp414 | 1.102 | 0.002269 | 0.0063398 |
| Marchf9 | 1.782 | 0.00227 | 0.0063433 |
| Bbs5 | 1.106 | 0.00228 | 0.0063668 |
| Tafa2 | 2.273 | 0.002283 | 0.0063737 |
| Mmp11 | 2.135 | 0.002299 | 0.0064143 |
| Hypk | 1.577 | 0.002308 | 0.0064358 |
| Cd3d | -2.013 | 0.00231 | 0.0064407 |
| Mycn | -2.147 | 0.002326 | 0.0064811 |
| Zbtb8a | 1.114 | 0.002341 | 0.0065201 |
| Trim72 | -3.485 | 0.00239 | 0.0066416 |
| Lysmd2 | 1.714 | 0.002413 | 0.0067006 |
| Fam216a | 1.034 | 0.002417 | 0.0067101 |
| Tmem107 | 1.434 | 0.002447 | 0.0067808 |
| Dlgap5 | 1.840 | 0.00246 | 0.0068132 |
| Sirpb1c | 2.232 | 0.002471 | 0.0068408 |
| Slc9a3 | -2.000 | 0.002478 | 0.0068583 |
| Sprn | 1.066 | 0.002511 | 0.0069389 |
| Mtus2 | 1.734 | 0.002521 | 0.006964 |
| Anln | 1.511 | 0.002577 | 0.0071097 |
| Chtf18 | 2.654 | 0.002604 | 0.0071803 |
| Orc1 | 4.248 | 0.002614 | 0.007206 |
| Uba6 | 1.034 | 0.002622 | 0.0072262 |
| Zfp763 | -1.094 | 0.002659 | 0.0073206 |
| Hcrtr2 | -3.322 | 0.002663 | 0.0073319 |
| Traf1 | -2.082 | 0.002684 | 0.0073841 |
| Ect2 | 1.334 | 0.002703 | 0.0074316 |
| Nexn | 1.219 | 0.002754 | 0.0075575 |
| Klhl15 | 1.216 | 0.002786 | 0.0076319 |
| Cox7a1 | -1.710 | 0.002826 | 0.0077339 |
| Zfp239 | -2.585 | 0.00283 | 0.0077449 |
| Dyrk3 | 1.414 | 0.002865 | 0.0078272 |
| Cyp11b2 | -6.644 | 0.002937 | 0.0080144 |
| Gm45713 | -8.822 | 0.002973 | 0.0081079 |
| Spint1 | -1.297 | 0.002986 | 0.0081414 |
| Arfgef1 | 1.014 | 0.002986 | 0.0081414 |
| Xcr1 | -1.652 | 0.003007 | 0.0081926 |
| Slc22a26 | 1.437 | 0.003034 | 0.0082556 |
| Cep290 | 1.585 | 0.003093 | 0.0084075 |
| Sgcg | -5.644 | 0.003094 | 0.0084081 |
| Cep135 | 1.029 | 0.003176 | 0.0086161 |
| H1f4 | 1.508 | 0.003187 | 0.0086418 |
| Cep295 | 1.409 | 0.003226 | 0.0087343 |
| Iqgap3 | 1.935 | 0.003277 | 0.0088623 |
| Tnf | -4.030 | 0.003302 | 0.0089274 |
| Tmem45a | -1.182 | 0.003305 | 0.0089335 |
| Pagr1a | 2.175 | 0.003318 | 0.0089639 |
| 2010106E10Rik | -6.845 | 0.003318 | 0.0089639 |
| Lrrc24 | 4.304 | 0.003338 | 0.0090163 |
| Acsbg1 | -1.881 | 0.003407 | 0.0091897 |
| Prss35 | -2.848 | 0.00341 | 0.0091961 |
| Dusp14 | 1.685 | 0.003431 | 0.0092462 |
| Rhox5 | 4.369 | 0.003461 | 0.0093245 |
| Mpp4 | -1.313 | 0.0035 | 0.0094168 |
| D130043K22Rik | 1.057 | 0.003545 | 0.0095351 |
| Wnt7b | -2.619 | 0.003561 | 0.0095735 |
| Jph2 | -1.841 | 0.00361 | 0.0096849 |
| Pdlim3 | -1.451 | 0.003639 | 0.0097528 |
| Itgb1bp2 | -4.109 | 0.003702 | 0.0099043 |
| Zfp446 | 1.215 | 0.003835 | 0.0102403 |
| Spats2l | 1.675 | 0.003843 | 0.010261 |
| Prss1 | -7.551 | 0.003861 | 0.0103033 |
| Kazald1 | -1.261 | 0.003882 | 0.0103538 |
| Slc36a2 | -3.426 | 0.003905 | 0.0104099 |
| Zfp521 | -1.263 | 0.003907 | 0.0104143 |
| Abcb1a | 1.165 | 0.003914 | 0.0104265 |
| Knl1 | 2.459 | 0.003926 | 0.0104574 |
| Zfp938 | 1.239 | 0.004007 | 0.010654 |
| Ncr1 | -4.059 | 0.004014 | 0.0106685 |
| Fam83e | -3.233 | 0.004046 | 0.0107477 |
| B3gntl1 | 1.026 | 0.004069 | 0.010798 |
| Ampd1 | -6.679 | 0.004082 | 0.0108262 |
| C2cd4d | -1.401 | 0.004102 | 0.010874 |
| Ncaph | 1.268 | 0.004107 | 0.0108839 |
| Ank1 | -1.879 | 0.004217 | 0.0111568 |
| Slc14a1 | -1.719 | 0.004224 | 0.0111713 |
| Zfp959 | 1.282 | 0.004279 | 0.0113165 |
| Ndnf | -3.644 | 0.004442 | 0.0117094 |
| Ddx3y | 1.149 | 0.004444 | 0.0117127 |
| Polq | 3.248 | 0.004518 | 0.011892 |
| Apol6 | -6.077 | 0.004578 | 0.0120359 |
| Dnah8 | -2.000 | 0.004597 | 0.0120742 |
| Hao2 | -3.585 | 0.004606 | 0.0120924 |
| Slc19a3 | 4.196 | 0.004636 | 0.0121598 |
| Engase | 1.075 | 0.004764 | 0.0124629 |
| Atg9b | 2.700 | 0.004812 | 0.0125794 |
| Pbk | 1.916 | 0.004892 | 0.0127814 |
| Ift43 | 1.093 | 0.00492 | 0.0128481 |
| Irak3 | 1.083 | 0.004927 | 0.0128626 |
| Rasal3 | -1.030 | 0.004954 | 0.0129269 |
| Adgrg7 | -2.268 | 0.004974 | 0.0129758 |
| Chaf1b | 1.340 | 0.005033 | 0.0131241 |
| Zfp612 | 1.029 | 0.005061 | 0.0131917 |
| BC016579 | 2.293 | 0.005118 | 0.0133276 |
| Msc | -2.288 | 0.00515 | 0.0134054 |
| Tmem45b | -2.143 | 0.005272 | 0.0136971 |
| Ttc26 | 1.855 | 0.005306 | 0.0137764 |
| Bub1 | 4.285 | 0.005333 | 0.0138348 |
| Lrrc51 | 1.126 | 0.005342 | 0.0138536 |
| Pik3c2g | 1.549 | 0.005364 | 0.0139062 |
| Cenpf | 2.000 | 0.005424 | 0.0140388 |
| Ms4a1 | -1.465 | 0.005442 | 0.0140766 |
| Rims4 | -2.524 | 0.00548 | 0.01417 |
| Nnt | 11.239 | 0.005619 | 0.0145079 |
| Ttll9 | 7.103 | 0.005661 | 0.0146046 |
| Clcn1 | -5.409 | 0.005755 | 0.0148261 |
| Unc13d | -2.620 | 0.005786 | 0.0148982 |
| Shcbp1 | 1.616 | 0.005801 | 0.014933 |
| A930007A09Rik | -2.237 | 0.005832 | 0.0149989 |
| Nxpe5 | 3.503 | 0.00601 | 0.0154353 |
| Wdr93 | 1.957 | 0.006034 | 0.0154947 |
| Zfp202 | 1.538 | 0.006048 | 0.0155198 |
| Lrrcc1 | 1.543 | 0.006053 | 0.0155291 |
| Alpk3 | -3.000 | 0.006197 | 0.0158768 |
| 1110002E22Rik | -2.000 | 0.006245 | 0.0159927 |
| Tub | -5.229 | 0.006261 | 0.0160308 |
| A1bg | 3.188 | 0.006305 | 0.016139 |
| Ssmem1 | 4.129 | 0.006339 | 0.0162198 |
| Veph1 | -2.087 | 0.00637 | 0.016285 |
| Ptprcap | -1.204 | 0.006483 | 0.0165501 |
| Wscd1 | -1.386 | 0.0065 | 0.0165849 |
| Ttll3 | -2.663 | 0.006621 | 0.0168744 |
| H1f3 | 1.663 | 0.006635 | 0.0169047 |
| Pls1 | 1.525 | 0.006638 | 0.0169103 |
| Esm1 | -1.194 | 0.006651 | 0.0169359 |
| Slc6a2 | -5.229 | 0.006682 | 0.0170108 |
| Ube2t | 2.300 | 0.00673 | 0.0171119 |
| Ppp1r14a | -1.188 | 0.00677 | 0.0171945 |
| Kif20b | 1.784 | 0.006944 | 0.0176066 |
| 1700020L24Rik | 2.300 | 0.006972 | 0.0176689 |
| Synpo2l | -4.170 | 0.007121 | 0.0180128 |
| Ccl19 | -1.101 | 0.007138 | 0.018053 |
| Anxa8 | 1.922 | 0.007141 | 0.018057 |
| Stil | 2.087 | 0.00718 | 0.0181431 |
| Gtse1 | 1.651 | 0.007225 | 0.0182536 |
| Spag4 | -1.139 | 0.007228 | 0.0182572 |
| Cdk5rap2 | 1.026 | 0.007252 | 0.0183124 |
| Lyrm7 | 1.081 | 0.007274 | 0.0183644 |
| Cyp4f39 | 1.047 | 0.00731 | 0.0184442 |
| Pif1 | 4.322 | 0.007402 | 0.0186471 |
| Myom3 | 1.248 | 0.007485 | 0.0188448 |
| Arhgap11a | 1.025 | 0.0076 | 0.0191152 |
| Map10 | -1.115 | 0.007613 | 0.0191449 |
| Strada | 1.120 | 0.00763 | 0.0191785 |
| Erp27 | 7.531 | 0.007679 | 0.0192978 |
| Diaph3 | 1.678 | 0.007711 | 0.0193684 |
| Lrrc30 | -6.409 | 0.007852 | 0.0196879 |
| Rad54b | 1.907 | 0.007896 | 0.019794 |
| Cyp3a44 | 3.907 | 0.00795 | 0.0199269 |
| Tmem171 | -2.170 | 0.007956 | 0.0199401 |
| Miox | 7.366 | 0.007967 | 0.0199587 |
| Rell2 | -2.202 | 0.007991 | 0.0200058 |
| Cttnbp2 | -2.914 | 0.008046 | 0.0201363 |
| Kif21b | -1.011 | 0.008113 | 0.0202881 |
| Lox | 1.343 | 0.008134 | 0.0203365 |
| Slit1 | -1.837 | 0.008152 | 0.0203772 |
| Ccdc34 | 1.950 | 0.008202 | 0.0204932 |
| Myoc | -3.350 | 0.008202 | 0.0204932 |
| Cphx1 | 1.328 | 0.008335 | 0.020796 |
| Gm12184 | 2.668 | 0.008393 | 0.0209232 |
| Il12rb1 | 1.326 | 0.008419 | 0.0209718 |
| Angpt2 | -1.257 | 0.008467 | 0.0210844 |
| Tff3 | 1.012 | 0.008546 | 0.0212546 |
| 1810046K07Rik | 1.971 | 0.008569 | 0.0213097 |
| Kdm8 | 1.032 | 0.008695 | 0.0216103 |
| Gm19426 | -2.053 | 0.008897 | 0.0220722 |
| Flrt1 | 1.202 | 0.009061 | 0.0224457 |
| Mgl2 | -1.401 | 0.009089 | 0.0225086 |
| Fbn2 | 4.129 | 0.009153 | 0.0226522 |
| Gm49359 | 1.209 | 0.009261 | 0.0228985 |
| Ercc6l | 2.064 | 0.009296 | 0.0229716 |
| Gnb1l | 1.465 | 0.009445 | 0.0232937 |
| Kcnc4 | -2.485 | 0.009769 | 0.0240215 |
| Bspry | 1.936 | 0.009771 | 0.0240224 |
| Igsf1 | -1.406 | 0.01007 | 0.0247101 |
| Cyp2c65 | -2.107 | 0.010121 | 0.0248211 |
| Retnlg | 1.432 | 0.010239 | 0.0250794 |
| Ndor1 | 2.917 | 0.010264 | 0.0251333 |
| Mylk2 | -2.678 | 0.01035 | 0.025326 |
| Akr1c21 | -2.644 | 0.010359 | 0.0253399 |
| Prickle4 | -1.923 | 0.010472 | 0.0255851 |
| Car4 | -7.366 | 0.010643 | 0.0259732 |
| Npy4r | -3.585 | 0.010651 | 0.0259869 |
| Tlr11 | -1.657 | 0.010667 | 0.0260199 |
| H4c14 | 2.708 | 0.010688 | 0.0260632 |
| Oip5 | 2.610 | 0.010708 | 0.0261032 |
| Rasgrf1 | -3.954 | 0.010777 | 0.0262646 |
| Duoxa2 | -3.143 | 0.010804 | 0.0263232 |
| Rab36 | -1.300 | 0.010833 | 0.0263815 |
| Trim63 | -6.022 | 0.01088 | 0.0264793 |
| Efcab7 | 2.848 | 0.0112 | 0.0271781 |
| Spata24 | 1.623 | 0.011219 | 0.027213 |
| Pla2g2d | -3.492 | 0.011228 | 0.0272266 |
| Ccdc163 | 1.234 | 0.011288 | 0.0273475 |
| Usp9x | 1.027 | 0.011361 | 0.0275006 |
| Kif9 | 1.475 | 0.011371 | 0.0275183 |
| Foxm1 | 1.044 | 0.011562 | 0.0279137 |
| Hey2 | -2.285 | 0.011584 | 0.0279564 |
| Efhd1 | -1.083 | 0.011613 | 0.0280185 |
| Ank2 | 1.158 | 0.01168 | 0.0281726 |
| Osgin2 | 1.483 | 0.011888 | 0.0286448 |
| Ssc4d | 1.097 | 0.011901 | 0.0286727 |
| Rab27b | -1.015 | 0.011946 | 0.0287625 |
| Rhbdl1 | -1.043 | 0.011975 | 0.0288156 |
| Gna15 | -1.273 | 0.012067 | 0.0289999 |
| Dmkn | 2.489 | 0.01215 | 0.029183 |
| Tnnc1 | -1.639 | 0.012184 | 0.0292596 |
| St6galnac3 | -1.422 | 0.012334 | 0.0295972 |
| Zfp322a | 1.064 | 0.012484 | 0.0299234 |
| Cdkn2b | -1.981 | 0.01251 | 0.0299804 |
| Rgs7bp | -1.040 | 0.012584 | 0.0301318 |
| Nhlrc1 | 1.187 | 0.012692 | 0.0303629 |
| Chmp4c | 1.112 | 0.012743 | 0.0304715 |
| Gm21188 | 4.087 | 0.012991 | 0.0309806 |
| Parpbp | 3.492 | 0.013048 | 0.0310983 |
| Pibf1 | 1.229 | 0.013067 | 0.0311307 |
| Bmp8b | 2.747 | 0.013073 | 0.0311348 |
| Espl1 | 1.648 | 0.013073 | 0.0311348 |
| Sh3rf2 | -2.202 | 0.01315 | 0.0312949 |
| Nusap1 | 1.534 | 0.013301 | 0.0316253 |
| Eme1 | 3.555 | 0.013353 | 0.0317361 |
| Cd209a | -1.061 | 0.013426 | 0.031889 |
| Smc6 | 1.036 | 0.013446 | 0.0319182 |
| Ppp1r1a | -6.607 | 0.0135 | 0.0320319 |
| Timp4 | -4.524 | 0.013502 | 0.0320323 |
| Hs6st2 | 5.322 | 0.013596 | 0.0322094 |
| Rad51b | 1.657 | 0.013617 | 0.0322527 |
| Tmem17 | 1.345 | 0.013732 | 0.0324785 |
| Ska3 | 2.433 | 0.013798 | 0.0326102 |
| Zfp760 | 1.250 | 0.013848 | 0.0327051 |
| Ush1g | -1.293 | 0.013989 | 0.0330164 |
| Igkv4-59 | -3.594 | 0.014006 | 0.0330451 |
| Cdc45 | 1.164 | 0.014066 | 0.0331834 |
| Lrrc26 | -6.607 | 0.014081 | 0.0332075 |
| Ankrd6 | -1.585 | 0.01409 | 0.0332245 |
| Eaf2 | -2.291 | 0.014142 | 0.0333333 |
| Gm14419 | 1.900 | 0.01416 | 0.0333704 |
| Zfp109 | 1.024 | 0.014555 | 0.0342124 |
| Hoxb2 | -1.080 | 0.014573 | 0.0342518 |
| Zfp951 | 1.525 | 0.01463 | 0.0343614 |
| Ngp | 1.353 | 0.01479 | 0.0347115 |
| Mmp8 | 1.939 | 0.014799 | 0.0347229 |
| Ppp4r4 | 1.115 | 0.014822 | 0.0347673 |
| Vmn2r20 | -1.737 | 0.014827 | 0.0347736 |
| Cdh11 | 1.250 | 0.014881 | 0.0348753 |
| Ovol1 | 2.755 | 0.015208 | 0.0356002 |
| Flrt2 | 1.307 | 0.01524 | 0.0356707 |
| Retnla | -3.615 | 0.01536 | 0.0359269 |
| Arhgap36 | -2.285 | 0.01542 | 0.0360355 |
| Jrkl | 1.158 | 0.015443 | 0.0360798 |
| Tcte2 | -2.020 | 0.01559 | 0.0363653 |
| Trim68 | 1.218 | 0.016182 | 0.0376077 |
| Serpina5 | 2.341 | 0.016202 | 0.0376373 |
| Kif14 | 2.248 | 0.016204 | 0.0376374 |
| Pwwp4c | 3.755 | 0.016218 | 0.0376651 |
| Tbc1d2 | 1.200 | 0.016472 | 0.038173 |
| Ifit1bl1 | -1.526 | 0.016555 | 0.0383551 |
| Mks1 | 1.178 | 0.01657 | 0.0383749 |
| Cacng6 | -5.781 | 0.01657 | 0.0383749 |
| Cdc25c | 3.000 | 0.016582 | 0.0383899 |
| Trarg1 | -2.170 | 0.016862 | 0.0389676 |
| 3110082I17Rik | 1.094 | 0.016888 | 0.0390177 |
| Ighg1 | 1.177 | 0.016933 | 0.0391039 |
| Nos2 | -2.632 | 0.016954 | 0.0391465 |
| Egr2 | 4.087 | 0.017091 | 0.0394142 |
| Hrob | 2.784 | 0.017158 | 0.0395405 |
| Bicdl2 | -3.369 | 0.017586 | 0.040424 |
| Prr18 | -3.087 | 0.017586 | 0.040424 |
| Aspm | 1.585 | 0.017819 | 0.0409207 |
| Atp8b4 | 1.044 | 0.01798 | 0.0412594 |
| Cend1 | 2.682 | 0.018214 | 0.0417228 |
| Dhrs7c | -6.679 | 0.018267 | 0.0418254 |
| Pcdhgb4 | -1.202 | 0.01834 | 0.0419637 |
| Mlf1 | -6.570 | 0.018738 | 0.0428212 |
| Cenpe | 2.202 | 0.01874 | 0.0428212 |
| Alpk1 | 1.266 | 0.018914 | 0.0431875 |
| Zfp112 | 1.274 | 0.019037 | 0.0434499 |
| Fxyd3 | -1.279 | 0.019118 | 0.0436118 |
| Tbx15 | -5.492 | 0.019199 | 0.0437587 |
| Cenpu | 2.471 | 0.019293 | 0.0439422 |
| Dctd | 4.392 | 0.019299 | 0.0439457 |
| Pkhd1l1 | 1.263 | 0.01951 | 0.044361 |
| C1ql3 | 1.549 | 0.019913 | 0.045168 |
| Zfp605 | 1.095 | 0.020189 | 0.045724 |
| Sbspon | -5.409 | 0.020349 | 0.046048 |
| 1810062G17Rik | -1.785 | 0.020581 | 0.0465231 |
| Tmprss4 | -1.481 | 0.020625 | 0.0466092 |
| Smim6 | -1.288 | 0.020658 | 0.0466643 |
| Gm21992 | 2.121 | 0.02066 | 0.0466643 |
| Ptp4a1 | -2.212 | 0.020893 | 0.0471502 |
| Taf9 | 1.098 | 0.020902 | 0.0471628 |
| Pknox2 | -1.601 | 0.020935 | 0.0472188 |
| Rsph9 | -1.338 | 0.021085 | 0.047531 |
| Ppil6 | -2.652 | 0.021191 | 0.0477304 |
| Rasl12 | -2.170 | 0.021425 | 0.0482111 |
| Foxj1 | -1.700 | 0.021473 | 0.0483066 |
| Car15 | -1.834 | 0.021556 | 0.0484805 |
| Sh3bgr | -7.229 | 0.02159 | 0.0485301 |
| Fancd2 | 2.054 | 0.021993 | 0.049342 |
| Jrk | 1.148 | 0.0221 | 0.049541 |
| Aplp1 | 1.193 | 0.022196 | 0.0497343 |
| Kcnt2 | 1.469 | 0.022316 | 0.0499916 |
| Lrrn1 | -2.459 | 0.022323 | 0.0499998 |
| Kcnj5 | -2.663 | 0.02253 | 0.0504136 |
| Cacna2d4 | 4.781 | 0.022637 | 0.050619 |
| Aph1c | 1.130 | 0.022848 | 0.0510643 |
| Pcdhga5 | -1.087 | 0.023059 | 0.0514942 |
| Cd8b1 | -1.744 | 0.023074 | 0.0515126 |
| BC055324 | 2.068 | 0.023098 | 0.0515522 |
| Il17rd | -1.170 | 0.023252 | 0.0518402 |
| Meox1 | -1.823 | 0.02346 | 0.0522324 |
| Lrrc75a | 1.409 | 0.023507 | 0.0523094 |
| Pimreg | 4.030 | 0.023568 | 0.0524377 |
| Ildr1 | -1.750 | 0.023668 | 0.05259 |
| Lat | -1.437 | 0.023669 | 0.05259 |
| Ctse | -1.204 | 0.023688 | 0.0526204 |
| Izumo1r | -2.415 | 0.024072 | 0.0534114 |
| Ska1 | 4.000 | 0.024498 | 0.0542351 |
| A530064D06Rik | 1.522 | 0.024554 | 0.0543518 |
| Ighv1-55 | -2.316 | 0.024575 | 0.0543835 |
| Efr3b | 1.527 | 0.02465 | 0.0545343 |
| Il17re | -1.314 | 0.024678 | 0.0545901 |
| Rasd1 | 1.382 | 0.024727 | 0.0546752 |
| Arc | -2.059 | 0.02498 | 0.0551536 |
| Nfasc | 1.175 | 0.025076 | 0.0553434 |
| Trbc1 | -1.236 | 0.025154 | 0.0555007 |
| Ptafr | 1.029 | 0.025158 | 0.0555029 |
| Gm14418 | 1.346 | 0.025195 | 0.0555771 |
| Ube2v2 | 1.033 | 0.025213 | 0.0556082 |
| Vmn1r90 | -2.585 | 0.02558 | 0.0563042 |
| Sptbn4 | 4.755 | 0.025801 | 0.056768 |
| Sema7a | -1.404 | 0.026021 | 0.0571937 |
| Carf | 1.269 | 0.02612 | 0.0573635 |
| Nat8l | -2.000 | 0.02632 | 0.0577636 |
| Itgb3bp | 1.555 | 0.026571 | 0.0582674 |
| Osr1 | -3.585 | 0.026595 | 0.0583128 |
| Misp | -1.534 | 0.026648 | 0.058421 |
| Gpr137c | -3.939 | 0.0268 | 0.0587068 |
| Ccdc14 | 1.449 | 0.026851 | 0.0588121 |
| Cidea | -1.984 | 0.026891 | 0.0588917 |
| Spata22 | 1.018 | 0.02714 | 0.0593807 |
| Pcdhga3 | -1.263 | 0.027151 | 0.0593968 |
| Plekhh1 | 1.436 | 0.027482 | 0.0600655 |
| Neurl1b | 1.563 | 0.027703 | 0.0605085 |
| Six1 | -4.781 | 0.028077 | 0.0612594 |
| Tnni1 | -6.570 | 0.02821 | 0.0615243 |
| Fcmr | -1.175 | 0.028216 | 0.0615299 |
| B3gnt4 | -6.229 | 0.028238 | 0.0615698 |
| Ugt1a2 | -5.781 | 0.028251 | 0.0615905 |
| Cenpq | 1.154 | 0.028259 | 0.0615906 |
| Cd3e | -1.339 | 0.028327 | 0.061729 |
| Acyp2 | 1.169 | 0.028443 | 0.0619347 |
| Stac3 | -3.222 | 0.028463 | 0.0619698 |
| Upf2 | 1.157 | 0.028717 | 0.0624561 |
| Wasf3 | -3.322 | 0.029216 | 0.0634419 |
| Slc9a2 | -3.322 | 0.029375 | 0.0637454 |
| Myadml2 | -5.714 | 0.029417 | 0.0638021 |
| Wfdc3 | 2.390 | 0.029607 | 0.0641618 |
| Fut2 | -2.115 | 0.029645 | 0.0642285 |
| Stc1 | -3.700 | 0.029755 | 0.0644421 |
| Cd46 | 1.948 | 0.029852 | 0.064627 |
| Sphk1 | -1.222 | 0.029862 | 0.0646287 |
| Fam189b | -1.428 | 0.02994 | 0.0647752 |
| Magohb | 1.185 | 0.030123 | 0.065145 |
| Tox3 | -2.807 | 0.030191 | 0.0652822 |
| Obp2a | -1.093 | 0.030384 | 0.0656311 |
| Mxd3 | 3.728 | 0.030807 | 0.0664055 |
| Slc2a4 | -1.019 | 0.030873 | 0.0665296 |
| B3galt2 | -3.644 | 0.030975 | 0.0667061 |
| Ighv1-82 | -2.463 | 0.031031 | 0.0668093 |
| Zfp683 | -2.678 | 0.031354 | 0.0674259 |
| Pkdrej | -1.663 | 0.031391 | 0.0674962 |
| Fam189a1 | -2.415 | 0.031564 | 0.0678239 |
| Noxo1 | -1.258 | 0.03181 | 0.0682801 |
| Fam107a | 1.858 | 0.032083 | 0.0688088 |
| Gm27029 | -1.835 | 0.032151 | 0.068922 |
| Aqp7 | -5.492 | 0.032268 | 0.069156 |
| Rad54l | 2.737 | 0.032457 | 0.0694964 |
| Dgkg | -3.170 | 0.0325 | 0.0695808 |
| 2310002L09Rik | -6.022 | 0.032557 | 0.0696919 |
| Klhl40 | -5.570 | 0.032585 | 0.0697444 |
| Hexdc | 1.006 | 0.032659 | 0.0698923 |
| Mst1r | -1.237 | 0.032731 | 0.0700199 |
| Nsg2 | -5.492 | 0.032761 | 0.0700665 |
| Nudt10 | 2.305 | 0.033009 | 0.0705315 |
| Npy | -3.021 | 0.033066 | 0.0706263 |
| Serpini2 | -6.229 | 0.033108 | 0.0706981 |
| Emb | 1.098 | 0.033355 | 0.0711783 |
| Lgals12 | -3.459 | 0.033372 | 0.0712061 |
| Cerkl | 1.617 | 0.033684 | 0.0718163 |
| Alms1 | 1.038 | 0.033756 | 0.0719224 |
| Dbil5 | -2.311 | 0.034104 | 0.0725976 |
| Zbtb25 | 1.338 | 0.034388 | 0.0731341 |
| Pou2af1 | -1.271 | 0.034391 | 0.0731341 |
| Rnf222 | -2.051 | 0.034598 | 0.0735158 |
| Psmc3ip | 2.151 | 0.034987 | 0.0742956 |
| Zfp105 | 1.555 | 0.035401 | 0.0750862 |
| Srcin1 | -1.222 | 0.035461 | 0.0752045 |
| Wscd2 | -2.415 | 0.035638 | 0.0755319 |
| Cavin4 | -5.781 | 0.03632 | 0.0769163 |
| Rasl10b | 1.659 | 0.036385 | 0.0770356 |
| Tctn1 | 1.005 | 0.036466 | 0.0771858 |
| Asprv1 | 2.722 | 0.036472 | 0.077188 |
| Grhl3 | -1.372 | 0.036574 | 0.0773457 |
| Ydjc | 1.680 | 0.036713 | 0.0776084 |
| Mep1b | -2.322 | 0.036909 | 0.0780024 |
| Ap1m2 | -1.311 | 0.037252 | 0.0786208 |
| Prkrip1 | 1.423 | 0.037319 | 0.0787391 |
| Sowaha | -3.170 | 0.037336 | 0.078763 |
| Stac | -3.087 | 0.037405 | 0.0788681 |
| Shisa3 | 1.837 | 0.037458 | 0.0789604 |
| Spdya | 1.490 | 0.037635 | 0.0792621 |
| Prkd1 | -2.907 | 0.037791 | 0.0795494 |
| Umod | -5.322 | 0.037933 | 0.0798086 |
| Cthrc1 | 7.077 | 0.038131 | 0.0802011 |
| Ddx25 | 2.907 | 0.038134 | 0.0802011 |
| Gnaz | -4.907 | 0.038151 | 0.0802267 |
| Ankrd37 | -1.470 | 0.038635 | 0.0811497 |
| Zfp994 | 1.010 | 0.038703 | 0.0812731 |
| Map3k9 | -1.348 | 0.038713 | 0.0812753 |
| Tmem221 | -1.705 | 0.038752 | 0.0813446 |
| Rgma | -1.031 | 0.038816 | 0.0814569 |
| Cntf | -1.360 | 0.03883 | 0.0814758 |
| Spdl1 | 2.000 | 0.038868 | 0.081546 |
| Gpr141 | 1.248 | 0.039202 | 0.0821486 |
| A630001G21Rik | -1.027 | 0.039416 | 0.082537 |
| Ndc80 | 2.132 | 0.039454 | 0.0826064 |
| Aldh1a3 | 1.745 | 0.039462 | 0.0826122 |
| Mybl1 | 1.102 | 0.039504 | 0.0826913 |
| Kcnd3 | -3.907 | 0.039526 | 0.0827151 |
| Gm14296 | 1.914 | 0.039948 | 0.0834817 |
| Ccn6 | -2.585 | 0.04005 | 0.0836732 |
| Cyp2d11 | -2.624 | 0.040206 | 0.0839678 |
| Ppp1r26 | -1.503 | 0.040301 | 0.0841219 |
| Zfp972 | -1.648 | 0.04054 | 0.0845579 |
| Tedc1 | 2.256 | 0.040835 | 0.0850859 |
| Kcnma1 | -1.737 | 0.041016 | 0.0854227 |
| Cspg4 | -1.737 | 0.041444 | 0.0861907 |
| Ighv1-72 | -3.027 | 0.041466 | 0.086227 |
| Slc44a4 | -1.044 | 0.041685 | 0.0866162 |
| Me3 | -1.637 | 0.041929 | 0.0870617 |
| Dntt | 1.207 | 0.0422 | 0.0875855 |
| Blk | -1.700 | 0.042462 | 0.0880746 |
| Acan | -4.129 | 0.042879 | 0.0888157 |
| Car6 | 6.366 | 0.0431 | 0.0892288 |
| Gsdme | 1.072 | 0.04346 | 0.0899056 |
| Rdh16f1 | 5.781 | 0.044291 | 0.09143 |
| Zfp449 | 1.706 | 0.044372 | 0.091575 |
| Gm3055 | -1.973 | 0.045171 | 0.0930134 |
| Lypd6 | -2.322 | 0.045449 | 0.0935388 |
| Chad | -1.716 | 0.045595 | 0.0937618 |
| Rslcan18 | 1.074 | 0.045874 | 0.0942242 |
| Capn13 | -3.000 | 0.046427 | 0.0952377 |
| Aqp12 | -6.814 | 0.046431 | 0.0952377 |
| Tnfrsf10b | 1.503 | 0.047082 | 0.0964763 |
| Zfp599 | -2.138 | 0.047127 | 0.096557 |
| Pcdhga2 | -4.781 | 0.048015 | 0.0982056 |
| Iglon5 | -2.044 | 0.048463 | 0.0989855 |
| Mesp1 | 6.607 | 0.048494 | 0.0990365 |
| Lag3 | -1.248 | 0.048619 | 0.099243 |
| Rab37 | -1.933 | 0.049064 | 0.1000133 |
| Themis | 1.044 | 0.04929 | 0.1004154 |
| Gli2 | -3.459 | 0.049525 | 0.1008417 |
| Macc1 | -4.644 | 0.049693 | 0.10112 |

| **Table S9**. The DEGs between the Model and BHP PM-SS group | | | |
| --- | --- | --- | --- |
| Symbol | log2(fc) | P Value | FDR |
| Sqstm1 | -1.939 | 0 | 0 |
| Sgk1 | -2.242 | 0 | 0 |
| Ddit4 | -2.887 | 0 | 0 |
| Igfbp1 | -2.163 | 0 | 0 |
| Ddit3 | -2.081 | 0 | 0 |
| Atf3 | -2.239 | 0 | 0 |
| Cyp2b10 | -2.837 | 0 | 0 |
| Fgf21 | -2.190 | 0 | 0 |
| Apoa4 | -2.493 | 0 | 0 |
| Trib3 | -2.385 | 0 | 0 |
| Srxn1 | -1.864 | 0 | 0 |
| Serpina6 | 1.463 | 0 | 0 |
| Cyp4a10 | -1.713 | 0 | 0 |
| G6pc | -1.486 | 0 | 0 |
| Gpt2 | -1.236 | 7.34E-296 | 7.99E-293 |
| Tat | -1.210 | 2.99E-290 | 3.05E-287 |
| Cdkn1a | -2.925 | 1.49E-285 | 1.44E-282 |
| Plk3 | -1.640 | 7.17E-273 | 6.51E-270 |
| Tubb2a | -1.796 | 2.17E-269 | 1.87E-266 |
| Ifrd1 | -1.834 | 2.98E-266 | 2.44E-263 |
| Cyp2a5 | -1.033 | 6.54E-238 | 5.09E-235 |
| Nr1d1 | 3.450 | 2.08E-237 | 1.54E-234 |
| Ddx28 | -1.852 | 3.54E-232 | 2.51E-229 |
| Mup17 | -1.557 | 5.95E-203 | 4.05E-200 |
| Eif4ebp3 | -2.441 | 5.83E-200 | 3.81E-197 |
| Atp2a2 | -1.059 | 2.23E-190 | 1.40E-187 |
| Arrdc3 | 1.834 | 1.52E-189 | 9.23E-187 |
| Cln8 | -1.266 | 3.66E-188 | 2.14E-185 |
| Mup15 | -1.150 | 6.21E-181 | 3.50E-178 |
| Tsku | -1.085 | 1.70E-178 | 9.24E-176 |
| Npc1 | -1.192 | 6.39E-177 | 3.37E-174 |
| Slc20a1 | -1.545 | 3.87E-169 | 1.97E-166 |
| Dbp | 3.258 | 1.09E-163 | 5.39E-161 |
| Inmt | 1.176 | 1.09E-162 | 5.26E-160 |
| Gm4756 | 1.640 | 9.85E-161 | 4.60E-158 |
| Cyp1a1 | -2.933 | 1.92E-160 | 8.70E-158 |
| Plpp3 | 1.238 | 1.13E-159 | 4.97E-157 |
| Adh4 | 1.300 | 2.07E-159 | 8.91E-157 |
| Tubb4b | -1.075 | 3.09E-157 | 1.29E-154 |
| Hsd3b3 | 1.214 | 7.60E-157 | 3.11E-154 |
| Cidec | -4.177 | 1.05E-153 | 4.20E-151 |
| Hsd17b6 | 1.319 | 2.45E-149 | 9.29E-147 |
| Slc3a2 | -1.145 | 5.23E-147 | 1.94E-144 |
| Lifr | 1.106 | 5.68E-146 | 2.06E-143 |
| Myc | -2.358 | 7.28E-142 | 2.53E-139 |
| C9orf72 | -1.812 | 1.64E-128 | 5.26E-126 |
| Ppp1r15a | -1.594 | 1.63E-127 | 5.11E-125 |
| Gfpt1 | -1.289 | 3.45E-126 | 1.06E-123 |
| Nfil3 | -1.653 | 1.28E-124 | 3.81E-122 |
| Gpat3 | -1.750 | 4.74E-122 | 1.38E-119 |
| Irs2 | -1.663 | 1.66E-119 | 4.75E-117 |
| Sik1 | -1.749 | 6.68E-117 | 1.88E-114 |
| Clint1 | -1.113 | 2.36E-116 | 6.52E-114 |
| Mthfr | -1.725 | 5.47E-116 | 1.49E-113 |
| Bhlha15 | -1.241 | 4.89E-115 | 1.29E-112 |
| Klhdc7a | 1.622 | 1.15E-114 | 2.99E-112 |
| Gpr146 | 1.298 | 2.04E-114 | 5.21E-112 |
| Adrm1 | -1.031 | 2.06E-112 | 5.10E-110 |
| Mybbp1a | -1.210 | 2.42E-111 | 5.81E-109 |
| Stk40 | -1.334 | 2.18E-109 | 5.08E-107 |
| Bdh1 | 1.091 | 2.22E-109 | 5.12E-107 |
| Rarres1 | -1.230 | 3.01E-109 | 6.84E-107 |
| Upp2 | 1.261 | 3.26E-107 | 7.20E-105 |
| Rorc | -1.237 | 4.83E-106 | 1.05E-103 |
| Mup1 | -1.264 | 7.73E-106 | 1.64E-103 |
| Chka | -1.574 | 1.97E-103 | 3.97E-101 |
| Dnajb9 | -1.048 | 4.35E-102 | 8.55E-100 |
| Cry1 | -1.920 | 5.07E-99 | 9.42E-97 |
| Mknk1 | -1.380 | 1.82E-97 | 3.34E-95 |
| Hspb1 | -1.346 | 1.10E-95 | 1.97E-93 |
| S1pr1 | 1.193 | 4.12E-95 | 7.31E-93 |
| Arl4a | -1.365 | 5.12E-95 | 9.00E-93 |
| St6gal1 | 1.174 | 1.65E-91 | 2.82E-89 |
| Abca8a | 1.180 | 2.73E-88 | 4.55E-86 |
| Stard4 | 1.042 | 1.64E-87 | 2.70E-85 |
| Aldoa | -1.009 | 3.24E-86 | 5.29E-84 |
| Rnase4 | 1.069 | 2.27E-85 | 3.67E-83 |
| Arntl | -1.896 | 9.93E-85 | 1.59E-82 |
| Ddx21 | -1.068 | 7.25E-81 | 1.11E-78 |
| Slc25a42 | 1.129 | 1.33E-80 | 1.99E-78 |
| Mideas | -1.555 | 3.65E-79 | 5.28E-77 |
| Arhgef2 | -1.587 | 3.13E-76 | 4.33E-74 |
| Rap1gap2 | -2.516 | 2.58E-74 | 3.40E-72 |
| Hspa1b | 1.182 | 1.33E-72 | 1.72E-70 |
| Abcg8 | 1.190 | 3.23E-72 | 4.13E-70 |
| Lipc | 1.036 | 1.11E-71 | 1.38E-69 |
| Camk1d | 1.115 | 3.92E-71 | 4.85E-69 |
| Hsd3b5 | 2.388 | 2.24E-70 | 2.75E-68 |
| Prune1 | -1.164 | 8.35E-69 | 9.95E-67 |
| Derl3 | 1.315 | 3.37E-68 | 3.96E-66 |
| Wdr43 | -1.411 | 4.15E-68 | 4.84E-66 |
| Pnrc1 | -1.042 | 4.37E-67 | 4.96E-65 |
| Klkb1 | 1.102 | 1.09E-65 | 1.18E-63 |
| Nop58 | -1.924 | 1.04E-64 | 1.09E-62 |
| Aldh18a1 | -2.149 | 1.85E-64 | 1.94E-62 |
| Rrp12 | -1.381 | 1.69E-62 | 1.70E-60 |
| Nop56 | -1.168 | 2.29E-62 | 2.29E-60 |
| Ypel5 | -1.113 | 1.42E-61 | 1.41E-59 |
| Cpeb2 | -1.205 | 2.63E-61 | 2.59E-59 |
| Cyp4a31 | -1.242 | 8.55E-60 | 8.31E-58 |
| Rusc1 | -1.239 | 6.81E-59 | 6.50E-57 |
| Cad | -1.178 | 1.27E-58 | 1.20E-56 |
| Arfgap3 | -1.123 | 2.47E-58 | 2.31E-56 |
| Acot3 | 2.133 | 7.12E-55 | 6.09E-53 |
| Irf2bp2 | -1.245 | 3.30E-54 | 2.76E-52 |
| Rassf1 | -1.552 | 7.57E-54 | 6.22E-52 |
| Impact | -1.784 | 2.35E-53 | 1.91E-51 |
| Nifk | -1.005 | 2.96E-52 | 2.31E-50 |
| Cyp4a32 | -1.159 | 4.09E-50 | 3.06E-48 |
| Trmt61a | -1.408 | 1.15E-49 | 8.47E-48 |
| Ngdn | -1.150 | 5.04E-49 | 3.61E-47 |
| Slc16a6 | -1.965 | 6.17E-49 | 4.40E-47 |
| Leap2 | -1.114 | 1.04E-48 | 7.34E-47 |
| H3c14 | -1.409 | 4.35E-48 | 3.04E-46 |
| Mcm10 | 1.069 | 4.95E-48 | 3.42E-46 |
| Pnrc2 | -1.042 | 1.96E-47 | 1.33E-45 |
| Zbtb21 | -1.434 | 5.01E-47 | 3.35E-45 |
| Slc2a1 | -1.334 | 6.73E-47 | 4.49E-45 |
| Myorg | 1.043 | 2.33E-46 | 1.52E-44 |
| Nat8f5 | -1.575 | 5.31E-46 | 3.42E-44 |
| Fam135a | -1.288 | 9.35E-46 | 5.96E-44 |
| Nop2 | -1.160 | 2.89E-45 | 1.82E-43 |
| Nceh1 | 1.127 | 6.84E-45 | 4.18E-43 |
| Plaa | -1.018 | 2.45E-44 | 1.46E-42 |
| Mafk | -1.239 | 1.55E-43 | 9.10E-42 |
| Mup12 | -1.041 | 9.31E-43 | 5.37E-41 |
| Cdcp1 | -1.643 | 2.09E-42 | 1.18E-40 |
| Slc7a1 | -2.007 | 2.34E-42 | 1.32E-40 |
| Lrrc58 | -1.043 | 1.15E-41 | 6.38E-40 |
| Mbnl2 | -1.105 | 3.62E-41 | 1.96E-39 |
| Sesn2 | -1.255 | 7.67E-41 | 4.08E-39 |
| Rock2 | -1.474 | 1.05E-40 | 5.52E-39 |
| Ctps | -1.295 | 1.45E-39 | 7.31E-38 |
| Mthfd2 | -1.751 | 1.91E-39 | 9.61E-38 |
| Kctd15 | -1.572 | 6.69E-39 | 3.30E-37 |
| Naa25 | -1.015 | 1.11E-38 | 5.45E-37 |
| Gtf2ird1 | -1.037 | 1.31E-38 | 6.43E-37 |
| Slc7a11 | -2.692 | 1.39E-38 | 6.76E-37 |
| Hamp2 | 1.369 | 2.37E-38 | 1.14E-36 |
| Nrep | 2.060 | 2.52E-38 | 1.21E-36 |
| Tubb6 | -1.311 | 2.80E-38 | 1.34E-36 |
| Tnfrsf12a | -1.218 | 4.23E-38 | 2.02E-36 |
| Lgalsl | -1.082 | 8.27E-38 | 3.88E-36 |
| 1700017B05Rik | -1.279 | 8.37E-37 | 3.83E-35 |
| Arl4d | -1.308 | 2.40E-36 | 1.08E-34 |
| Ncald | 1.612 | 5.23E-36 | 2.35E-34 |
| Flywch1 | -1.084 | 5.73E-36 | 2.56E-34 |
| Ciapin1 | -1.070 | 7.02E-36 | 3.10E-34 |
| Rora | -1.674 | 1.02E-35 | 4.47E-34 |
| Rras2 | -1.070 | 1.88E-35 | 8.21E-34 |
| Utp20 | -1.012 | 2.72E-35 | 1.18E-33 |
| Plekhb1 | 1.072 | 3.38E-35 | 1.45E-33 |
| Pwp2 | -1.147 | 7.11E-35 | 3.01E-33 |
| Nfxl1 | -1.097 | 9.43E-35 | 3.97E-33 |
| Saa3 | 1.296 | 1.90E-34 | 7.93E-33 |
| Fabp5 | 1.263 | 7.52E-34 | 3.04E-32 |
| Hid1 | -1.317 | 1.25E-33 | 5.00E-32 |
| Gadd45g | -1.661 | 4.34E-33 | 1.69E-31 |
| Tsr1 | -1.018 | 8.03E-33 | 3.10E-31 |
| Ube2o | -1.151 | 9.89E-33 | 3.79E-31 |
| Yrdc | -1.013 | 1.09E-32 | 4.15E-31 |
| Polr1f | -1.338 | 1.37E-31 | 4.96E-30 |
| Spring1 | -1.225 | 1.45E-31 | 5.22E-30 |
| Gm45753 | 1.160 | 1.58E-31 | 5.68E-30 |
| Spata5 | -1.358 | 2.80E-31 | 9.96E-30 |
| Bcl6 | 1.033 | 7.36E-31 | 2.56E-29 |
| Cxcl1 | 1.459 | 8.09E-31 | 2.80E-29 |
| Phlda1 | 1.141 | 8.37E-31 | 2.89E-29 |
| Tasor2 | -1.278 | 1.00E-30 | 3.44E-29 |
| Btg2 | -1.319 | 1.74E-30 | 5.91E-29 |
| Btg3 | -1.088 | 3.69E-30 | 1.25E-28 |
| Socs2 | 1.372 | 4.83E-30 | 1.62E-28 |
| Wipf3 | -1.061 | 5.30E-30 | 1.77E-28 |
| Notum | 1.264 | 9.24E-30 | 3.05E-28 |
| Ajuba | -1.141 | 9.41E-30 | 3.10E-28 |
| Pvr | -1.119 | 2.27E-29 | 7.36E-28 |
| Hsd3b2 | 1.090 | 5.52E-29 | 1.76E-27 |
| Rgs16 | 1.352 | 1.81E-28 | 5.64E-27 |
| Fibin | -1.409 | 4.83E-28 | 1.47E-26 |
| Mak16 | -1.049 | 8.62E-28 | 2.59E-26 |
| Pdk4 | -1.076 | 1.23E-27 | 3.65E-26 |
| Nr2c2ap | -1.114 | 2.97E-27 | 8.73E-26 |
| Mdn1 | -1.121 | 6.29E-27 | 1.82E-25 |
| Dixdc1 | 1.215 | 6.58E-27 | 1.90E-25 |
| Nqo1 | -1.026 | 8.56E-27 | 2.45E-25 |
| Nr0b2 | 1.243 | 9.25E-27 | 2.64E-25 |
| Usp20 | -1.107 | 3.20E-26 | 8.93E-25 |
| B3galt1 | -1.520 | 4.79E-26 | 1.33E-24 |
| Polr3e | -1.015 | 4.99E-26 | 1.38E-24 |
| Adgrf1 | 2.729 | 5.00E-26 | 1.38E-24 |
| Moxd1 | -2.979 | 7.04E-26 | 1.93E-24 |
| Gpnmb | -1.358 | 7.41E-26 | 2.03E-24 |
| Lrit1 | 1.780 | 7.44E-26 | 2.03E-24 |
| Pum3 | -1.083 | 8.55E-26 | 2.33E-24 |
| Bco2 | 1.107 | 9.18E-26 | 2.50E-24 |
| Ppan | -1.002 | 1.03E-25 | 2.80E-24 |
| Ccdc149 | -1.086 | 1.60E-25 | 4.31E-24 |
| Cyp2b9 | 1.500 | 2.78E-25 | 7.43E-24 |
| Pdrg1 | -1.017 | 8.14E-25 | 2.12E-23 |
| Rnf186 | 2.423 | 1.21E-24 | 3.13E-23 |
| Tbc1d31 | -1.041 | 3.07E-24 | 7.76E-23 |
| D630039A03Rik | 1.404 | 3.50E-24 | 8.83E-23 |
| Cyp26b1 | -1.313 | 6.17E-24 | 1.55E-22 |
| Kctd9 | -1.097 | 4.72E-23 | 1.14E-21 |
| Mthfd1l | -1.628 | 7.41E-23 | 1.77E-21 |
| Skil | -1.040 | 1.02E-22 | 2.43E-21 |
| Arl14ep | -1.164 | 1.90E-22 | 4.46E-21 |
| Nfkbiz | 1.169 | 2.18E-22 | 5.11E-21 |
| Usp16 | -1.012 | 2.95E-22 | 6.83E-21 |
| Ccnb1 | -2.895 | 3.19E-22 | 7.37E-21 |
| Mnt | -1.206 | 4.14E-22 | 9.49E-21 |
| Samd4 | -1.293 | 9.89E-22 | 2.22E-20 |
| E030018B13Rik | -3.037 | 1.38E-21 | 3.08E-20 |
| Onecut1 | 1.009 | 2.38E-21 | 5.24E-20 |
| Nol8 | -2.053 | 6.12E-21 | 1.33E-19 |
| Muc1 | 2.421 | 6.66E-21 | 1.44E-19 |
| Dnajc2 | -1.098 | 8.29E-21 | 1.77E-19 |
| Trp53inp1 | -1.242 | 1.99E-20 | 4.18E-19 |
| Cyp17a1 | -1.290 | 2.72E-20 | 5.68E-19 |
| Pde4b | -1.017 | 3.21E-20 | 6.68E-19 |
| Lipg | 1.354 | 1.08E-19 | 2.18E-18 |
| Mlkl | -1.397 | 2.09E-19 | 4.14E-18 |
| Syt1 | 1.129 | 3.03E-19 | 5.95E-18 |
| Plekhh3 | -1.124 | 3.99E-19 | 7.81E-18 |
| Lad1 | -1.054 | 5.61E-19 | 1.08E-17 |
| Bmf | 1.291 | 6.55E-19 | 1.25E-17 |
| Cdc20 | -2.596 | 7.25E-19 | 1.38E-17 |
| Itpkc | -1.020 | 8.88E-19 | 1.69E-17 |
| Fyb2 | -1.134 | 9.32E-19 | 1.77E-17 |
| Nxpe2 | 1.259 | 9.89E-19 | 1.88E-17 |
| Zfp9 | -1.232 | 1.48E-18 | 2.78E-17 |
| Shroom3 | -1.093 | 2.10E-18 | 3.92E-17 |
| Ccdc186 | -1.128 | 2.29E-18 | 4.27E-17 |
| Unc5b | -1.320 | 4.88E-18 | 8.90E-17 |
| Ccdc134 | -1.238 | 7.13E-18 | 1.29E-16 |
| Paqr3 | -1.123 | 8.20E-18 | 1.47E-16 |
| Tmem263 | -1.133 | 3.11E-17 | 5.33E-16 |
| Kif20a | -1.813 | 4.32E-17 | 7.36E-16 |
| Mab21l3 | -1.650 | 6.10E-17 | 1.03E-15 |
| Tppp | 1.109 | 8.21E-17 | 1.37E-15 |
| Pycr2 | -1.188 | 1.12E-16 | 1.84E-15 |
| Gfod1 | -1.164 | 1.16E-16 | 1.90E-15 |
| Rrp9 | -1.012 | 1.30E-16 | 2.12E-15 |
| Ccdc120 | -1.659 | 1.46E-16 | 2.37E-15 |
| Ighg2b | 1.571 | 1.61E-16 | 2.61E-15 |
| Cpeb1 | -1.367 | 2.52E-16 | 4.04E-15 |
| Tmtc2 | 1.089 | 2.70E-16 | 4.32E-15 |
| Rnd1 | -1.015 | 2.79E-16 | 4.45E-15 |
| Sucnr1 | 1.189 | 3.05E-16 | 4.85E-15 |
| Suco | -1.057 | 4.33E-16 | 6.83E-15 |
| Polr1e | -1.148 | 4.80E-16 | 7.54E-15 |
| Rnf152 | 1.033 | 1.26E-15 | 1.92E-14 |
| Spire2 | -3.026 | 2.31E-15 | 3.44E-14 |
| Slc39a6 | -1.248 | 2.98E-15 | 4.42E-14 |
| Dclk3 | 1.224 | 4.48E-15 | 6.53E-14 |
| Dsg1c | 1.340 | 5.43E-15 | 7.86E-14 |
| D630045J12Rik | -1.121 | 5.61E-15 | 8.11E-14 |
| Treh | -1.371 | 1.11E-14 | 1.56E-13 |
| Yod1 | -1.018 | 1.13E-14 | 1.60E-13 |
| Tlr12 | 1.248 | 1.64E-14 | 2.28E-13 |
| 2610528J11Rik | 1.315 | 1.89E-14 | 2.62E-13 |
| Nt5e | -1.136 | 2.46E-14 | 3.38E-13 |
| Sult1c2 | 1.967 | 3.11E-14 | 4.24E-13 |
| Frat1 | -1.125 | 4.54E-14 | 6.11E-13 |
| Clcf1 | -2.189 | 4.60E-14 | 6.19E-13 |
| Mllt11 | -1.851 | 4.69E-14 | 6.31E-13 |
| Rrm2 | -1.005 | 4.74E-14 | 6.36E-13 |
| H3c15 | -1.594 | 4.77E-14 | 6.40E-13 |
| Btc | -1.696 | 7.71E-14 | 1.02E-12 |
| Vmn2r3 | -1.205 | 8.07E-14 | 1.07E-12 |
| Ccne2 | -1.997 | 1.83E-13 | 2.37E-12 |
| Il18 | 1.031 | 2.28E-13 | 2.92E-12 |
| Chac1 | -1.182 | 2.52E-13 | 3.22E-12 |
| Ube2c | -2.036 | 8.00E-13 | 9.80E-12 |
| Fam222a | -1.525 | 9.01E-13 | 1.10E-11 |
| Tmem25 | 1.165 | 1.30E-12 | 1.56E-11 |
| Kbtbd12 | -1.794 | 1.80E-12 | 2.14E-11 |
| Zfp280c | -1.286 | 2.75E-12 | 3.24E-11 |
| Lrit2 | 2.247 | 3.48E-12 | 4.05E-11 |
| Dact1 | -1.169 | 3.51E-12 | 4.09E-11 |
| Cntnap1 | -1.730 | 3.54E-12 | 4.12E-11 |
| Fam118a | -1.128 | 4.04E-12 | 4.68E-11 |
| Gar1 | -1.654 | 4.26E-12 | 4.92E-11 |
| Dusp8 | -1.211 | 5.50E-12 | 6.29E-11 |
| Knstrn | -2.192 | 6.68E-12 | 7.57E-11 |
| Prc1 | -1.869 | 7.21E-12 | 8.15E-11 |
| Igkc | 1.091 | 1.01E-11 | 1.13E-10 |
| Dqx1 | 1.364 | 1.44E-11 | 1.59E-10 |
| Uhrf1 | -2.038 | 1.85E-11 | 2.02E-10 |
| Sult1e1 | -4.755 | 1.91E-11 | 2.08E-10 |
| Lgals4 | 1.980 | 2.03E-11 | 2.19E-10 |
| Ccna2 | -1.742 | 2.83E-11 | 3.04E-10 |
| Hspbap1 | -1.309 | 2.89E-11 | 3.09E-10 |
| Qtrt1 | -1.313 | 5.08E-11 | 5.32E-10 |
| Cd24a | 1.112 | 5.84E-11 | 6.07E-10 |
| Top2a | -1.595 | 6.21E-11 | 6.43E-10 |
| Fcgbp | 3.322 | 7.22E-11 | 7.43E-10 |
| Rhof | -1.935 | 1.32E-10 | 1.32E-09 |
| Akr1b7 | -1.037 | 1.77E-10 | 1.76E-09 |
| Nle1 | -1.328 | 2.38E-10 | 2.33E-09 |
| Rfx4 | 2.665 | 2.44E-10 | 2.39E-09 |
| Hmmr | -2.640 | 2.47E-10 | 2.42E-09 |
| Pcdhgc4 | 5.426 | 2.82E-10 | 2.75E-09 |
| Lysmd3 | -1.083 | 3.15E-10 | 3.06E-09 |
| Unc93a2 | 1.187 | 6.15E-10 | 5.80E-09 |
| Grem2 | 1.139 | 7.51E-10 | 7.05E-09 |
| Fam89a | 1.141 | 7.64E-10 | 7.15E-09 |
| Tnfrsf19 | 1.075 | 8.11E-10 | 7.57E-09 |
| Akap17b | -1.093 | 1.00E-09 | 9.28E-09 |
| Cdk1 | -1.249 | 1.53E-09 | 1.39E-08 |
| Dusp4 | -1.341 | 1.76E-09 | 1.61E-08 |
| Muc3 | 3.285 | 2.07E-09 | 1.87E-08 |
| Cdca3 | -1.360 | 2.23E-09 | 2.00E-08 |
| Plk1 | -2.251 | 2.34E-09 | 2.09E-08 |
| Ttll7 | -1.165 | 2.65E-09 | 2.36E-08 |
| Ldlrad4 | 1.250 | 2.94E-09 | 2.60E-08 |
| Sprr1a | -2.588 | 3.83E-09 | 3.36E-08 |
| Myom3 | -2.511 | 6.31E-09 | 5.44E-08 |
| Cep192 | -1.177 | 7.57E-09 | 6.45E-08 |
| Adcy1 | -1.684 | 7.61E-09 | 6.48E-08 |
| Ahi1 | -1.320 | 8.48E-09 | 7.18E-08 |
| Lonrf3 | -1.664 | 8.51E-09 | 7.19E-08 |
| Racgap1 | -2.004 | 1.21E-08 | 1.01E-07 |
| Ccnb2 | -2.137 | 1.62E-08 | 1.33E-07 |
| Ckmt1 | 2.439 | 1.74E-08 | 1.43E-07 |
| Aifm3 | 1.048 | 1.89E-08 | 1.54E-07 |
| Alpk1 | 1.350 | 1.97E-08 | 1.60E-07 |
| Acot6 | -1.400 | 1.98E-08 | 1.61E-07 |
| Fam83f | -1.278 | 2.03E-08 | 1.64E-07 |
| Nek1 | -1.022 | 2.39E-08 | 1.92E-07 |
| Nek2 | -1.158 | 2.52E-08 | 2.02E-07 |
| Parp2 | -1.020 | 2.86E-08 | 2.26E-07 |
| Dus4l | -1.040 | 3.33E-08 | 2.62E-07 |
| Ces4a | 1.014 | 3.69E-08 | 2.88E-07 |
| Asic5 | -1.326 | 3.78E-08 | 2.95E-07 |
| Zbtb10 | -1.079 | 4.14E-08 | 3.21E-07 |
| Ccdc66 | -1.473 | 5.63E-08 | 4.32E-07 |
| Cyp2a4 | -1.553 | 6.13E-08 | 4.69E-07 |
| Zfp948 | -1.457 | 6.55E-08 | 4.99E-07 |
| Ppp1r3g | -1.705 | 7.92E-08 | 5.98E-07 |
| Srgap3 | 1.435 | 8.44E-08 | 6.36E-07 |
| Caap1 | -1.150 | 9.79E-08 | 7.31E-07 |
| Gjc3 | -1.757 | 1.05E-07 | 7.80E-07 |
| Ckap2 | -2.837 | 1.15E-07 | 8.53E-07 |
| Hinfp | -1.161 | 1.17E-07 | 8.67E-07 |
| Bmper | -1.387 | 1.18E-07 | 8.73E-07 |
| Adm2 | -2.385 | 1.27E-07 | 9.39E-07 |
| Taf4b | -1.071 | 1.29E-07 | 9.52E-07 |
| Rundc3b | -1.833 | 1.31E-07 | 9.66E-07 |
| Arhgap6 | 1.176 | 1.69E-07 | 1.23E-06 |
| Gabrp | 3.550 | 1.76E-07 | 1.28E-06 |
| Dmbt1 | 2.837 | 1.96E-07 | 1.41E-06 |
| Zfp51 | -1.137 | 2.59E-07 | 1.84E-06 |
| Tacc3 | -1.402 | 3.43E-07 | 2.41E-06 |
| Cwc27 | -1.120 | 4.02E-07 | 2.80E-06 |
| Hhipl2 | -1.240 | 5.27E-07 | 3.59E-06 |
| Amigo3 | -1.234 | 5.32E-07 | 3.63E-06 |
| Mmp7 | 2.060 | 5.58E-07 | 3.79E-06 |
| Rpp38 | -1.064 | 5.80E-07 | 3.93E-06 |
| Prkab2 | -1.042 | 5.86E-07 | 3.96E-06 |
| Nexn | -1.447 | 5.88E-07 | 3.97E-06 |
| Wfdc2 | 1.267 | 5.97E-07 | 4.03E-06 |
| Bag2 | -1.083 | 6.36E-07 | 4.28E-06 |
| Igdcc4 | -1.427 | 6.37E-07 | 4.28E-06 |
| Gdf9 | -1.475 | 6.88E-07 | 4.60E-06 |
| Trim46 | -1.280 | 7.88E-07 | 5.22E-06 |
| Tgtp2 | 1.056 | 8.97E-07 | 5.90E-06 |
| Slfn9 | -1.209 | 1.07E-06 | 6.95E-06 |
| Hps6 | 1.052 | 1.15E-06 | 7.45E-06 |
| Tchh | -2.291 | 1.38E-06 | 8.86E-06 |
| Ckap2l | -1.794 | 1.45E-06 | 9.27E-06 |
| Cdc45 | -1.606 | 1.55E-06 | 9.87E-06 |
| Irak1bp1 | -1.373 | 2.15E-06 | 1.34E-05 |
| Cyp4f39 | -1.770 | 2.16E-06 | 1.34E-05 |
| Cela2a | 1.517 | 2.40E-06 | 1.48E-05 |
| Aurka | -1.224 | 2.45E-06 | 1.51E-05 |
| Clec2h | 1.809 | 2.52E-06 | 1.55E-05 |
| Adamdec1 | 1.636 | 2.53E-06 | 1.55E-05 |
| Gm6614 | -1.215 | 2.95E-06 | 1.80E-05 |
| Tpx2 | -1.314 | 3.23E-06 | 1.95E-05 |
| Duox2 | 2.632 | 3.48E-06 | 2.09E-05 |
| Ccl21a | 1.124 | 3.56E-06 | 2.14E-05 |
| Uprt | -1.192 | 3.83E-06 | 2.29E-05 |
| Nuf2 | -2.402 | 4.24E-06 | 2.51E-05 |
| Zfp37 | -2.096 | 4.24E-06 | 2.51E-05 |
| Il23a | -2.677 | 4.69E-06 | 2.76E-05 |
| Sntg2 | 1.249 | 6.50E-06 | 3.75E-05 |
| Fbxo32 | -1.139 | 6.99E-06 | 4.02E-05 |
| Timp1 | 1.076 | 7.23E-06 | 4.13E-05 |
| Muc4 | 2.503 | 7.72E-06 | 4.40E-05 |
| Dppa2 | -5.143 | 8.75E-06 | 4.94E-05 |
| Krt19 | 1.067 | 8.79E-06 | 4.96E-05 |
| Hells | -2.139 | 8.83E-06 | 4.98E-05 |
| Serpinb1a | 1.141 | 1.01E-05 | 5.61E-05 |
| Odf3b | 1.402 | 1.42E-05 | 7.70E-05 |
| Rad51b | 1.304 | 1.65E-05 | 8.89E-05 |
| Cdr2 | -1.151 | 1.67E-05 | 8.99E-05 |
| Sptssb | 7.966 | 1.72E-05 | 9.23E-05 |
| Cip2a | -1.757 | 1.92E-05 | 0.000102042 |
| Cux2 | -1.350 | 1.93E-05 | 0.000102608 |
| Zbtb8a | -1.114 | 1.97E-05 | 0.000104767 |
| H4c8 | -1.108 | 2.03E-05 | 0.000107735 |
| Hhipl1 | -1.237 | 2.45E-05 | 0.000128616 |
| Cpt1b | -2.401 | 3.10E-05 | 0.000160108 |
| Epcam | 1.180 | 3.16E-05 | 0.000163408 |
| Areg | -1.490 | 3.34E-05 | 0.000171393 |
| Nusap1 | -1.669 | 3.38E-05 | 0.000173321 |
| Rab36 | 2.014 | 3.44E-05 | 0.000176145 |
| B4galnt2 | 2.862 | 3.45E-05 | 0.000176443 |
| Tspan8 | 1.549 | 4.31E-05 | 0.000216786 |
| Myo15b | 2.019 | 4.54E-05 | 0.000227349 |
| Neurl1b | -1.563 | 4.65E-05 | 0.000232733 |
| Ncaph | -1.392 | 5.64E-05 | 0.000279293 |
| Birc5 | -1.736 | 5.80E-05 | 0.000286438 |
| Hirip3 | -1.019 | 6.68E-05 | 0.000326114 |
| Klf1 | 1.050 | 6.91E-05 | 0.000335834 |
| Ccl7 | 2.721 | 6.95E-05 | 0.000337663 |
| Fut1 | -1.172 | 8.08E-05 | 0.000388786 |
| Kif2c | -1.799 | 9.41E-05 | 0.000447325 |
| Ptk6 | 1.965 | 9.58E-05 | 0.000454326 |
| Kif22 | -1.484 | 0.000100114 | 0.000473775 |
| H1f4 | -1.850 | 0.000104029 | 0.000491162 |
| Fam83d | -2.126 | 0.000119625 | 0.000558819 |
| Fignl1 | -1.625 | 0.00013005 | 0.000604235 |
| Slc9a3 | 2.222 | 0.000137219 | 0.000634479 |
| Kif4 | -1.962 | 0.000144801 | 0.000667269 |
| Gal3st2 | 3.868 | 0.000146287 | 0.000673735 |
| Ctse | 1.860 | 0.000146588 | 0.000674935 |
| Ncapg | -2.239 | 0.000154433 | 0.000708059 |
| Fam110c | 1.404 | 0.000156172 | 0.000714828 |
| Prrg4 | -1.487 | 0.000181192 | 0.000817439 |
| Ect2 | -1.376 | 0.000183333 | 0.000826642 |
| Ehf | 1.897 | 0.00018614 | 0.000838607 |
| Lrtm2 | -2.496 | 0.000187639 | 0.000844659 |
| Cxcl5 | 2.746 | 0.000192993 | 0.00086767 |
| Nat8f6 | -1.776 | 0.00021162 | 0.000945518 |
| Npas2 | -1.342 | 0.00021311 | 0.000951711 |
| Clic6 | 3.196 | 0.000215197 | 0.000959719 |
| Olfm4 | 2.979 | 0.000226984 | 0.001007337 |
| Stap1 | -1.620 | 0.000231491 | 0.001026503 |
| Acod1 | 2.218 | 0.000242942 | 0.001073201 |
| Adgrg7 | 2.967 | 0.000243664 | 0.001076102 |
| Cbr3 | -1.053 | 0.000250646 | 0.001105439 |
| Flrt1 | -1.231 | 0.000261117 | 0.001146362 |
| H4c17 | -2.745 | 0.000295526 | 0.001284651 |
| Cdca5 | -2.485 | 0.0003014 | 0.00130879 |
| Reg1 | 2.807 | 0.000313143 | 0.001354801 |
| Rad54b | -2.322 | 0.000325309 | 0.00139996 |
| Mrnip | 1.163 | 0.000345168 | 0.001482298 |
| Pbk | -1.943 | 0.000400219 | 0.001699502 |
| Esco2 | -1.826 | 0.000406576 | 0.001725598 |
| Haus6 | -1.017 | 0.000409051 | 0.001733852 |
| Fam107a | -2.858 | 0.000434648 | 0.001833311 |
| Pde5a | -1.059 | 0.000449306 | 0.001890256 |
| Sptb | 1.087 | 0.00047246 | 0.001981033 |
| Rad51c | -1.230 | 0.000480329 | 0.002009388 |
| Zfp605 | -1.045 | 0.0005358 | 0.002224352 |
| Shh | 2.565 | 0.00058454 | 0.002401264 |
| Elfn1 | -1.948 | 0.000594057 | 0.002437095 |
| St6galnac3 | 2.454 | 0.000619236 | 0.002532126 |
| Mst1r | 2.026 | 0.000638972 | 0.002605003 |
| Tyro3 | -1.147 | 0.000662034 | 0.002690965 |
| Zfp791 | 1.293 | 0.00066719 | 0.002711247 |
| Ttll11 | -1.785 | 0.000677702 | 0.002747812 |
| Ltf | 1.187 | 0.000688824 | 0.002788755 |
| Duoxa2 | 4.196 | 0.000725723 | 0.002927263 |
| Cckar | 6.366 | 0.000786474 | 0.003147736 |
| Mtbp | -1.009 | 0.00079183 | 0.003163445 |
| Ccdc30 | 1.064 | 0.000845355 | 0.003360847 |
| Chek1 | -1.355 | 0.000907685 | 0.003587694 |
| Ttk | -2.121 | 0.000932892 | 0.003670455 |
| Unc13d | 1.492 | 0.000948591 | 0.003725042 |
| Mbp | 1.507 | 0.000961777 | 0.003774103 |
| Gm49359 | -1.162 | 0.000996482 | 0.003903724 |
| Gm14295 | 1.072 | 0.001048901 | 0.004087511 |
| Gm49340 | -2.896 | 0.001121953 | 0.004350398 |
| Lingo4 | -1.869 | 0.00114641 | 0.004441013 |
| Nhlrc1 | -1.057 | 0.001149504 | 0.004450887 |
| Actg2 | 1.290 | 0.001150737 | 0.004454606 |
| Unc93a | 6.366 | 0.001159922 | 0.00448485 |
| Capn3 | 1.023 | 0.001179636 | 0.004555682 |
| Slc44a4 | 1.870 | 0.001186463 | 0.004577718 |
| BC055324 | -2.245 | 0.001193357 | 0.004601059 |
| Tnf | 4.605 | 0.001263085 | 0.004838489 |
| Arhgap11a | -1.042 | 0.001268512 | 0.004854161 |
| Esrrg | 1.585 | 0.001287555 | 0.004918965 |
| Lratd1 | 1.127 | 0.001318731 | 0.005029838 |
| Spag5 | -1.437 | 0.001330665 | 0.0050718 |
| Arfgef3 | -1.652 | 0.001332303 | 0.005076859 |
| Ighd | 1.121 | 0.001336878 | 0.005091917 |
| Cfd | 1.731 | 0.001372413 | 0.005218746 |
| Large2 | 2.013 | 0.001487926 | 0.005617462 |
| Iqgap3 | -1.563 | 0.001491189 | 0.005628479 |
| Rgs1 | -1.332 | 0.001503063 | 0.00566937 |
| Pglyrp1 | 2.049 | 0.001555069 | 0.005853365 |
| Kif18b | -1.907 | 0.001570262 | 0.005905109 |
| Aunip | -3.011 | 0.001574401 | 0.005916588 |
| Ncmap | 4.939 | 0.001605513 | 0.006019659 |
| BC016579 | -2.293 | 0.001645374 | 0.006152171 |
| Aurkb | -1.332 | 0.00164728 | 0.006157088 |
| Nrg1 | -1.149 | 0.001727133 | 0.006422593 |
| Mbnl3 | -1.891 | 0.001729763 | 0.006430909 |
| Prr15l | 1.227 | 0.001739127 | 0.006461309 |
| Bhlhe41 | 1.601 | 0.00177608 | 0.006579147 |
| Raver2 | 1.649 | 0.00180607 | 0.006672086 |
| Pdzk1ip1 | 1.231 | 0.001817356 | 0.006710743 |
| Cenpf | -1.737 | 0.00194061 | 0.00713041 |
| Lrrc14b | -1.926 | 0.0020409 | 0.007475367 |
| Plet1 | 1.016 | 0.002084306 | 0.007618979 |
| Amn | -2.923 | 0.002137263 | 0.007789901 |
| Fbxw15 | -1.354 | 0.00215806 | 0.007858686 |
| Vil1 | 2.051 | 0.00223758 | 0.008124717 |
| Kntc1 | -1.322 | 0.002255307 | 0.008181812 |
| Anln | -1.151 | 0.00229271 | 0.008307572 |
| Ltb | 1.242 | 0.002339289 | 0.008460177 |
| Sftpd | 1.457 | 0.002373305 | 0.008571811 |
| Igkv2-137 | 4.263 | 0.00242446 | 0.008729553 |
| Spint1 | 1.466 | 0.002433131 | 0.008756913 |
| Anxa13 | 1.275 | 0.002505301 | 0.008988931 |
| Lpcat4 | 1.159 | 0.002644817 | 0.009431508 |
| Ccdc57 | 1.298 | 0.0028009 | 0.009927426 |
| Dtl | -1.329 | 0.002854594 | 0.010098017 |
| Chil4 | 7.050 | 0.002911137 | 0.010271348 |
| Clspn | -2.585 | 0.002913766 | 0.010278402 |
| Ptp4a1 | 1.503 | 0.002955187 | 0.010413274 |
| Lmntd2 | 1.758 | 0.003258194 | 0.011360909 |
| Ypel4 | -4.248 | 0.003358454 | 0.011663201 |
| Accs | 1.045 | 0.003403149 | 0.011793346 |
| Gm7298 | 1.967 | 0.0034209 | 0.011852349 |
| Gm43951 | -6.714 | 0.003423117 | 0.011857514 |
| Zfp566 | -1.146 | 0.003519449 | 0.012145918 |
| H4c3 | -3.270 | 0.00355481 | 0.01224876 |
| Cep126 | -1.722 | 0.003564154 | 0.012278367 |
| Ccl2 | 1.460 | 0.003602521 | 0.012389638 |
| Nudt10 | -2.692 | 0.003608051 | 0.012403434 |
| Unc79 | 3.000 | 0.003646686 | 0.012525708 |
| Tmem229a | 1.241 | 0.003844718 | 0.013123134 |
| Acta1 | 6.937 | 0.004135737 | 0.014019795 |
| Smim27 | 1.124 | 0.004144087 | 0.014039362 |
| Misp | 2.208 | 0.004469158 | 0.015009902 |
| Tmprss4 | 1.954 | 0.004796993 | 0.01595027 |
| Aqp5 | 7.155 | 0.004980271 | 0.016485856 |
| Lrrc24 | -3.134 | 0.005032637 | 0.016642338 |
| Slc9a4 | 2.954 | 0.005043819 | 0.016669193 |
| Scube1 | -1.562 | 0.005156555 | 0.0170108 |
| Chrnb2 | 1.090 | 0.005162661 | 0.017024067 |
| Myot | -6.276 | 0.005560195 | 0.018213624 |
| Cyp2g1 | 2.147 | 0.005580729 | 0.018273561 |
| Fancd2 | -1.667 | 0.005617303 | 0.018367547 |
| Fam83e | 3.070 | 0.005689502 | 0.018562752 |
| Hapln1 | -2.585 | 0.005840164 | 0.018993614 |
| Synpo2 | 1.206 | 0.005950274 | 0.019320944 |
| Serpina5 | -2.441 | 0.005967881 | 0.019370417 |
| Hkdc1 | 1.833 | 0.006003212 | 0.019473485 |
| Tmem54 | 3.907 | 0.006187633 | 0.020000233 |
| Ly6c2 | 1.060 | 0.006767595 | 0.021647822 |
| Slc14a1 | 1.585 | 0.006886719 | 0.021990116 |
| Olfr648 | 1.843 | 0.006987193 | 0.022297866 |
| Trpm5 | 5.644 | 0.00708055 | 0.022573771 |
| Flrt3 | 2.188 | 0.007109776 | 0.022658076 |
| Rps23rg1 | 1.322 | 0.007323549 | 0.023271249 |
| Krt80 | 1.117 | 0.007468299 | 0.023657578 |
| Dnah1 | -1.322 | 0.00754761 | 0.023885655 |
| Fmo3 | -1.299 | 0.007591485 | 0.024010553 |
| Sgo2a | -2.280 | 0.007622486 | 0.024094607 |
| Dnase1 | -4.102 | 0.007843617 | 0.024721845 |
| Rec8 | 1.295 | 0.007998521 | 0.025161534 |
| Ccdc189 | 2.128 | 0.008034644 | 0.025250854 |
| Rasl2-9 | 2.861 | 0.008068525 | 0.025337837 |
| Rab7b | 1.022 | 0.008083743 | 0.025370994 |
| Dtx1 | 1.158 | 0.008142326 | 0.025535236 |
| Slc26a3 | 1.948 | 0.008355374 | 0.026118139 |
| Degs2 | 2.064 | 0.00837643 | 0.026153929 |
| Aqp4 | -1.038 | 0.008448285 | 0.026353101 |
| Wnt7b | 2.716 | 0.008482172 | 0.026453752 |
| Cldn34c1 | 2.729 | 0.008695118 | 0.027050741 |
| Azin2 | 1.230 | 0.008945896 | 0.027751643 |
| Arhgap36 | 2.492 | 0.009051301 | 0.028032396 |
| Eme1 | -2.970 | 0.009319966 | 0.028726597 |
| Kcnd3 | 4.492 | 0.009358865 | 0.028830174 |
| Syn3 | 1.085 | 0.009364899 | 0.028832453 |
| Gm10309 | 4.129 | 0.009572298 | 0.02941555 |
| Aspm | -1.198 | 0.0095835 | 0.029433362 |
| Ptgir | 1.752 | 0.009704268 | 0.029781875 |
| Abhd1 | 1.053 | 0.010056334 | 0.030752577 |
| Trdc | -1.683 | 0.010146611 | 0.030982522 |
| Prune2 | 1.322 | 0.010152105 | 0.030993232 |
| Chrna4 | -2.397 | 0.010209573 | 0.031145394 |
| Abcc12 | 1.112 | 0.010241103 | 0.031224086 |
| Igkv12-44 | 3.709 | 0.010424464 | 0.031718019 |
| Aplnr | 4.248 | 0.010779237 | 0.032675749 |
| Ift81 | -1.127 | 0.010838319 | 0.032824392 |
| Knl1 | -1.874 | 0.010886586 | 0.032958354 |
| H4c14 | -2.222 | 0.010892431 | 0.032969938 |
| - | 3.322 | 0.011003717 | 0.033269798 |
| Kif20b | -1.096 | 0.011142669 | 0.033658773 |
| Ube2t | -1.654 | 0.011169944 | 0.033728689 |
| Car9 | 1.448 | 0.011306296 | 0.034096298 |
| Atp2a1 | 6.129 | 0.011555414 | 0.034764132 |
| Cnn1 | 1.074 | 0.011604889 | 0.034906549 |
| Adamts12 | -1.162 | 0.011622461 | 0.034952969 |
| Lrrc39 | -1.339 | 0.011644643 | 0.035000347 |
| Ssmem1 | -3.129 | 0.012275867 | 0.036628047 |
| Lzts1 | -1.762 | 0.012653854 | 0.03764585 |
| Celsr2 | 1.363 | 0.01275835 | 0.03794982 |
| Nr4a2 | -1.363 | 0.012825268 | 0.038121104 |
| Fcmr | 1.482 | 0.01285895 | 0.038200366 |
| Ms4a1 | 1.444 | 0.012959477 | 0.038436095 |
| Gp2 | 2.939 | 0.01340695 | 0.03960505 |
| Gm20547 | 7.077 | 0.013563998 | 0.040025555 |
| Slc7a6 | -1.322 | 0.013829612 | 0.040713741 |
| Plekhs1 | 2.485 | 0.013844453 | 0.040750086 |
| Nlgn2 | 1.014 | 0.013911079 | 0.040909341 |
| Spink4 | 3.242 | 0.014073641 | 0.041313029 |
| Slc16a3 | 1.137 | 0.014099239 | 0.041380735 |
| Fosb | 2.263 | 0.014459578 | 0.042339429 |
| Dnah7b | 3.322 | 0.014532512 | 0.042522501 |
| Prom1 | 1.027 | 0.014595147 | 0.042675198 |
| Col4a6 | 1.387 | 0.014693567 | 0.042924555 |
| Scimp | 1.009 | 0.014716419 | 0.042983625 |
| Gm19426 | 2.075 | 0.014765181 | 0.043110633 |
| A630001G21Rik | 1.469 | 0.014865914 | 0.043358251 |
| Vmn1r81 | 3.322 | 0.01514874 | 0.044080836 |
| AA467197 | 3.585 | 0.015277585 | 0.044400396 |
| Gabrb3 | 1.104 | 0.015361595 | 0.044604872 |
| Il7r | 1.490 | 0.015459783 | 0.044866053 |
| Rgs4 | 1.042 | 0.015521176 | 0.044988279 |
| Mup6 | -1.942 | 0.015639141 | 0.045281995 |
| Il4i1 | 1.957 | 0.015726122 | 0.045485469 |
| Cilp | 1.350 | 0.015812923 | 0.045671838 |
| Ctrb1 | 2.110 | 0.015876423 | 0.045830932 |
| Efcab7 | -1.801 | 0.015899442 | 0.045873063 |
| Clec4e | 1.527 | 0.01591169 | 0.04588409 |
| Igkv17-127 | 3.716 | 0.015967625 | 0.046029138 |
| Rd3 | 1.408 | 0.01604396 | 0.046224716 |
| Slc19a3 | -2.322 | 0.016115689 | 0.04640682 |
| Dnai1 | 1.080 | 0.016402929 | 0.047089652 |
| Ddx25 | -2.907 | 0.01652221 | 0.04737701 |
| Crybb3 | 1.367 | 0.016685617 | 0.047761504 |
| Tlcd5 | -1.585 | 0.016795094 | 0.048016214 |
| Gm17359 | -1.475 | 0.017136295 | 0.048914645 |
| 1700020L24Rik | -1.261 | 0.018126575 | 0.051391136 |
| Pagr1a | -1.791 | 0.018355379 | 0.051994707 |
| Osbpl10 | -1.138 | 0.01842331 | 0.052169041 |
| Pcdhga2 | 4.781 | 0.018552676 | 0.052471695 |
| Polq | -1.926 | 0.018652165 | 0.05268922 |
| Batf | 1.226 | 0.018994553 | 0.053526826 |
| Nos2 | 2.485 | 0.019170655 | 0.053939741 |
| Card14 | 3.322 | 0.019477526 | 0.054645192 |
| Gipc2 | 1.493 | 0.019478388 | 0.054645192 |
| Cend1 | -2.267 | 0.019555097 | 0.054832144 |
| Ranbp3l | 1.888 | 0.019581959 | 0.054888622 |
| Sez6l2 | 2.585 | 0.019756173 | 0.055348456 |
| Cyp2c65 | 2.182 | 0.019853444 | 0.055573314 |
| Gna15 | 1.415 | 0.019906916 | 0.055674551 |
| Mycn | 1.865 | 0.020021269 | 0.055939635 |
| Rell2 | 1.900 | 0.021238182 | 0.058836058 |
| Fut2 | 1.459 | 0.021645707 | 0.059791051 |
| Dmrt2 | -1.415 | 0.021954502 | 0.060511009 |
| A930007A09Rik | 2.147 | 0.021958257 | 0.06051115 |
| Atp10b | 2.459 | 0.023007395 | 0.063009021 |
| Slc4a9 | 1.914 | 0.023072211 | 0.063165349 |
| Ighv1-78 | 8.253 | 0.023305585 | 0.063676206 |
| 4933411K16Rik | 1.208 | 0.024076326 | 0.065420967 |
| Osr1 | 3.222 | 0.024465907 | 0.066314097 |
| Ccdc88a | -1.455 | 0.024708667 | 0.066850081 |
| Ighv1-15 | 8.344 | 0.02473985 | 0.066912282 |
| Ccr7 | 1.263 | 0.024805372 | 0.067022917 |
| Klf5 | 1.126 | 0.024986034 | 0.06738845 |
| Slc6a16 | 2.000 | 0.025406202 | 0.068284916 |
| Pcdhgb4 | 1.293 | 0.025510087 | 0.068530304 |
| Igsf1 | 1.678 | 0.025652514 | 0.068844993 |
| Slc9a2 | 3.459 | 0.025877842 | 0.069347184 |
| Il17re | 1.410 | 0.025928983 | 0.069461442 |
| Igkv5-48 | 2.921 | 0.026100887 | 0.069841785 |
| Stx1b | 1.102 | 0.026549821 | 0.070868935 |
| Prr15 | 1.408 | 0.027165304 | 0.072252102 |
| Apol7c | 2.100 | 0.027346449 | 0.072639283 |
| Ankrd55 | -1.546 | 0.027452884 | 0.072910148 |
| Rhbdl1 | 1.126 | 0.027473694 | 0.072953554 |
| Zfp239 | 2.280 | 0.027810109 | 0.073738994 |
| Gtse1 | -1.032 | 0.028046799 | 0.074318331 |
| Ankub1 | 1.794 | 0.028081181 | 0.074385304 |
| Prr11 | -1.333 | 0.028182453 | 0.074641464 |
| Zfp185 | 1.259 | 0.028608875 | 0.075623719 |
| A630076J17Rik | 7.626 | 0.029066708 | 0.07663553 |
| Vsig2 | 2.285 | 0.029219321 | 0.077000619 |
| Sgpp2 | 1.337 | 0.029315685 | 0.077229644 |
| Ankrd6 | 1.755 | 0.030065752 | 0.078976382 |
| Serpinb2 | 2.206 | 0.030367678 | 0.079654204 |
| Cbln3 | 1.538 | 0.030742396 | 0.080482016 |
| Cdca7l | 1.191 | 0.030905011 | 0.080842955 |
| Slc51b | -1.034 | 0.031099992 | 0.081274912 |
| Cnmd | 1.470 | 0.03127899 | 0.081651258 |
| Rad51ap1 | -1.084 | 0.031373104 | 0.081857694 |
| Gm49450 | 3.544 | 0.03145237 | 0.082006593 |
| Bcl2a1d | 1.593 | 0.032218321 | 0.083755331 |
| Ulk4 | -3.807 | 0.032837923 | 0.085135741 |
| Grid1 | -1.387 | 0.033069834 | 0.085628279 |
| Thsd4 | 1.180 | 0.033348312 | 0.086239991 |
| Saxo2 | -2.189 | 0.033362964 | 0.086264225 |
| Mettl7a2 | 2.644 | 0.033457302 | 0.086452403 |
| Sprr2a2 | 1.237 | 0.03365633 | 0.086826884 |
| Cds1 | 1.100 | 0.033811603 | 0.087134619 |
| Pgap4 | 1.495 | 0.035121306 | 0.089942258 |
| Dnaaf4 | 1.970 | 0.035154166 | 0.089984089 |
| Reg3g | 6.994 | 0.035192131 | 0.090053046 |
| Myo1a | 1.807 | 0.03531632 | 0.090342532 |
| Glb1l2 | 3.115 | 0.035747928 | 0.091317929 |
| Ccr5 | 1.169 | 0.036277633 | 0.092526372 |
| Fxyd3 | 1.115 | 0.036727425 | 0.093454706 |
| Bspry | -1.209 | 0.036849236 | 0.093720866 |
| Nudt11 | -1.311 | 0.036881019 | 0.093787098 |
| Gpr132 | 1.716 | 0.036951447 | 0.093922334 |
| Frem3 | 4.129 | 0.036959006 | 0.093926932 |
| Gpr17 | 2.322 | 0.037308858 | 0.094698182 |
| Ndnf | 3.322 | 0.037807388 | 0.095710754 |
| 1700030J22Rik | 1.646 | 0.037881231 | 0.095867976 |
| Vegfd | -1.349 | 0.038050178 | 0.09622397 |
| Adra2a | 3.807 | 0.038393663 | 0.096879654 |
| Noxo1 | 1.454 | 0.038573219 | 0.097242609 |
| Gm20878 | 2.844 | 0.038732874 | 0.097569813 |
| Csgalnact1 | -1.064 | 0.039229062 | 0.098652395 |
| Efcab11 | -3.322 | 0.039319957 | 0.098806432 |
| Spag4 | 1.100 | 0.03942279 | 0.098987197 |
| Gpr84 | 1.227 | 0.039535181 | 0.099208404 |
| Adrb3 | 1.322 | 0.03956931 | 0.099278797 |
| Tspoap1 | 3.170 | 0.039890981 | 0.09994771 |
| Prg2 | 3.524 | 0.039934524 | 0.100021613 |
| Shisa7 | -2.170 | 0.040392803 | 0.100941843 |
| Dnm3 | 1.389 | 0.041010091 | 0.102171854 |
| Olfr344 | -1.700 | 0.04110315 | 0.102369165 |
| Msc | 1.404 | 0.041143358 | 0.102441379 |
| Il1f9 | 1.495 | 0.041849065 | 0.103881832 |
| Gm3604 | -6.022 | 0.042039603 | 0.104243922 |
| Tarm1 | 2.921 | 0.042407604 | 0.105028899 |
| Adam12 | -1.069 | 0.043279188 | 0.106815049 |
| Trim80 | -1.191 | 0.043375799 | 0.107004991 |
| Rnase13 | 2.241 | 0.04338748 | 0.107017647 |
| Carmil3 | -4.322 | 0.043450756 | 0.107125196 |
| Dclk2 | 1.619 | 0.043776961 | 0.107766793 |
| Msmp | 1.014 | 0.044587587 | 0.109564203 |
| Wscd1 | 1.236 | 0.044621266 | 0.109630471 |
| Dgkh | 1.061 | 0.045066203 | 0.110607196 |
| Pcdhb4 | -2.459 | 0.045240071 | 0.110978249 |
| Egfl8 | 6.748 | 0.0452759 | 0.111021571 |
| Egfl6 | 5.229 | 0.04538963 | 0.111267257 |
| Gm49368 | 2.585 | 0.045549258 | 0.111591584 |
| Pcdhga3 | 1.341 | 0.04666159 | 0.1139578 |
| Reg2 | 2.778 | 0.047223486 | 0.115089193 |
| Oit1 | 2.109 | 0.047542588 | 0.115748487 |
| Lrmda | 1.345 | 0.047838035 | 0.116405961 |
| Trarg1 | 2.059 | 0.047920373 | 0.116569818 |
| Tox3 | 3.273 | 0.048049246 | 0.116795239 |
| Glt8d2 | -2.624 | 0.048052204 | 0.116795239 |
| S1pr4 | 1.083 | 0.048338321 | 0.117368433 |

| Table S10. The DEGs between the control and BHP PM-SS group | | | |
| --- | --- | --- | --- |
| Symbol | log2(fc) | P Value | FDR |
| Apcs | 4.245 | 0 | 0 |
| Lcn2 | 7.197 | 0 | 0 |
| Mt2 | 6.910 | 0 | 0 |
| Mt1 | 5.232 | 0 | 0 |
| Hsd3b5 | -3.690 | 0 | 0 |
| Elovl3 | -5.514 | 0 | 0 |
| Orm1 | 2.612 | 0 | 0 |
| Saa2 | 8.597 | 0 | 0 |
| Orm2 | 6.684 | 0 | 0 |
| Ppp1r3c | -2.754 | 0 | 0 |
| Selenbp2 | -3.754 | 0 | 0 |
| Mup14 | -2.273 | 0 | 0 |
| Mup11 | -2.857 | 0 | 0 |
| Mup7 | -2.645 | 0 | 0 |
| Saa1 | 7.129 | 0 | 0 |
| Mup19 | -3.265 | 0 | 0 |
| Mup16 | -2.303 | 0 | 0 |
| Mup1 | -2.758 | 0 | 0 |
| Mup9 | -2.564 | 0 | 0 |
| Mup2 | -3.582 | 0 | 0 |
| Mup12 | -3.020 | 0 | 0 |
| Mup15 | -3.829 | 0 | 0 |
| Mup17 | -4.669 | 0 | 0 |
| Trib3 | 5.240 | 1.22E-302 | 8.40E-300 |
| Glul | -2.419 | 2.32E-301 | 1.53E-298 |
| Fgl1 | 2.741 | 1.95E-291 | 1.24E-288 |
| Car3 | -2.195 | 7.80E-281 | 4.77E-278 |
| Plpp5 | 3.721 | 3.28E-268 | 1.94E-265 |
| Ctsl | 2.038 | 1.02E-261 | 5.83E-259 |
| Cyp7b1 | -1.640 | 4.69E-256 | 2.58E-253 |
| Ces1d | -1.479 | 1.47E-243 | 7.81E-241 |
| Cyp4a12b | -2.944 | 1.48E-234 | 7.63E-232 |
| Hao1 | -1.483 | 4.65E-214 | 2.32E-211 |
| Cyp4v3 | -1.079 | 6.90E-213 | 3.35E-210 |
| Nupr1 | 5.423 | 4.80E-212 | 2.26E-209 |
| Etnppl | -1.761 | 1.87E-206 | 8.58E-204 |
| Cyp2a5 | 1.928 | 7.80E-202 | 3.48E-199 |
| Scd1 | -1.228 | 4.11E-195 | 1.79E-192 |
| Mup20 | -1.398 | 5.61E-190 | 2.38E-187 |
| C6 | -1.436 | 7.18E-189 | 2.96E-186 |
| Keg1 | -1.597 | 2.15E-188 | 8.66E-186 |
| Tsc22d1 | -1.713 | 8.82E-188 | 3.47E-185 |
| Cry1 | -2.501 | 3.47E-187 | 1.33E-184 |
| Serpina3n | 2.669 | 1.35E-180 | 5.08E-178 |
| Mup22 | -1.461 | 6.49E-174 | 2.38E-171 |
| Itih3 | 2.220 | 1.71E-171 | 6.15E-169 |
| Srd5a1 | -1.681 | 1.39E-167 | 4.87E-165 |
| Mup3 | -1.506 | 2.24E-167 | 7.70E-165 |
| Ugt2b1 | -1.946 | 1.21E-162 | 4.07E-160 |
| Gclc | -1.041 | 1.40E-161 | 4.61E-159 |
| Serpina6 | 2.101 | 1.65E-160 | 5.35E-158 |
| Cyp2c40 | -2.942 | 1.80E-160 | 5.71E-158 |
| Cyp4a12a | -1.657 | 2.74E-158 | 8.55E-156 |
| Oat | -1.171 | 1.08E-153 | 3.31E-151 |
| Fgf21 | 6.211 | 2.18E-151 | 6.56E-149 |
| Pnrc1 | -1.395 | 1.55E-150 | 4.57E-148 |
| Ddit3 | 3.207 | 1.69E-149 | 4.88E-147 |
| Nr1d1 | 2.812 | 4.93E-143 | 1.38E-140 |
| Fkbp11 | 3.535 | 5.98E-135 | 1.64E-132 |
| Gstm3 | 1.909 | 1.95E-128 | 5.27E-126 |
| Dbp | 4.794 | 6.48E-127 | 1.72E-124 |
| Rbm3 | 2.239 | 2.85E-125 | 7.46E-123 |
| Cyp2a22 | 4.401 | 2.98E-123 | 7.70E-121 |
| Slc1a2 | -1.056 | 7.72E-123 | 1.96E-120 |
| Prg4 | 1.820 | 8.93E-123 | 2.23E-120 |
| Serp1 | 1.650 | 3.27E-120 | 8.06E-118 |
| Mup18 | -1.632 | 3.55E-120 | 8.62E-118 |
| Dio1 | -1.377 | 9.39E-120 | 2.25E-117 |
| Mup8 | -2.394 | 1.17E-119 | 2.77E-117 |
| Alas1 | 2.116 | 1.79E-118 | 4.16E-116 |
| Atf3 | 4.349 | 4.81E-116 | 1.10E-113 |
| Hpx | 2.078 | 7.26E-116 | 1.64E-113 |
| Slc22a28 | -3.486 | 5.13E-115 | 1.14E-112 |
| Ces2c | -2.209 | 1.33E-114 | 2.92E-112 |
| Serpina12 | -2.534 | 2.84E-114 | 6.16E-112 |
| Bhlha15 | 6.911 | 4.77E-113 | 1.02E-110 |
| Serpina1e | -1.450 | 2.31E-112 | 4.90E-110 |
| Cyp2b10 | 5.889 | 1.23E-111 | 2.57E-109 |
| Hspa5 | 1.695 | 6.80E-110 | 1.40E-107 |
| Lrrc59 | 1.779 | 8.56E-110 | 1.74E-107 |
| Hp | 2.008 | 1.04E-109 | 2.09E-107 |
| G6pc | -1.046 | 2.10E-109 | 4.18E-107 |
| Tmed3 | 2.644 | 8.90E-107 | 1.75E-104 |
| Nr1d2 | 2.894 | 1.03E-104 | 2.01E-102 |
| Sec61a1 | 1.778 | 1.52E-102 | 2.91E-100 |
| Ugt1a9 | -1.298 | 4.64E-102 | 8.81E-100 |
| Stt3a | 1.563 | 1.68E-101 | 3.15E-99 |
| Gldc | -1.217 | 5.04E-101 | 9.35E-99 |
| Cyp2c67 | -1.198 | 1.79E-100 | 3.28E-98 |
| Gmppb | 2.243 | 1.51E-99 | 2.73E-97 |
| Slc30a10 | -1.409 | 1.50E-98 | 2.69E-96 |
| Clpx | -1.313 | 2.19E-98 | 3.89E-96 |
| Slc25a25 | -1.011 | 5.00E-98 | 8.77E-96 |
| Ces1f | -1.032 | 5.60E-98 | 9.74E-96 |
| Tram1 | 1.292 | 4.44E-97 | 7.63E-95 |
| Lrg1 | 2.179 | 5.51E-96 | 9.28E-94 |
| Srxn1 | 1.632 | 7.17E-96 | 1.20E-93 |
| Clptm1l | 1.462 | 2.61E-95 | 4.30E-93 |
| Chka | -1.377 | 7.64E-95 | 1.25E-92 |
| Susd4 | -2.013 | 1.48E-94 | 2.40E-92 |
| Ces1e | -1.116 | 1.56E-94 | 2.51E-92 |
| Saa3 | 3.052 | 6.78E-94 | 1.08E-91 |
| Tubb2a | -1.391 | 7.96E-94 | 1.25E-91 |
| Sdf2l1 | 1.818 | 1.25E-92 | 1.95E-90 |
| Serpina7 | 2.466 | 1.84E-92 | 2.84E-90 |
| Arntl | -1.855 | 4.52E-92 | 6.91E-90 |
| Klhdc7a | 2.072 | 9.16E-92 | 1.39E-89 |
| Abcc4 | 2.904 | 4.16E-91 | 6.24E-89 |
| Steap4 | 1.318 | 7.95E-91 | 1.18E-88 |
| Ssr1 | 1.500 | 1.47E-90 | 2.17E-88 |
| Acot1 | -1.786 | 1.59E-90 | 2.33E-88 |
| Rorc | -1.239 | 4.45E-89 | 6.44E-87 |
| Srm | 2.037 | 1.44E-88 | 2.07E-86 |
| Stbd1 | 1.332 | 5.24E-88 | 7.45E-86 |
| Ifrd1 | 2.250 | 8.05E-88 | 1.14E-85 |
| Gstm4 | 2.011 | 1.25E-87 | 1.76E-85 |
| Serpina3m | 1.402 | 2.08E-87 | 2.89E-85 |
| Acaa1b | -1.272 | 1.24E-86 | 1.71E-84 |
| Gm4952 | -1.051 | 4.84E-86 | 6.61E-84 |
| Asns | 3.150 | 6.49E-86 | 8.78E-84 |
| Selenos | 1.569 | 6.68E-86 | 8.96E-84 |
| Nme6 | 2.596 | 5.64E-85 | 7.50E-83 |
| Mup21 | -1.801 | 4.17E-84 | 5.51E-82 |
| Psat1 | 4.419 | 2.95E-83 | 3.86E-81 |
| Ces3b | -1.408 | 5.02E-81 | 6.33E-79 |
| Trabd | 1.446 | 8.73E-80 | 1.08E-77 |
| Preb | 1.410 | 1.47E-79 | 1.82E-77 |
| Pgm3 | 2.098 | 6.72E-79 | 8.22E-77 |
| Txndc5 | 1.473 | 6.69E-78 | 8.06E-76 |
| Slc22a30 | -1.166 | 9.69E-78 | 1.16E-75 |
| Ppard | -2.194 | 1.74E-75 | 2.07E-73 |
| Slc38a10 | 1.636 | 5.62E-75 | 6.63E-73 |
| Manf | 1.593 | 1.04E-74 | 1.21E-72 |
| Dhdds | 1.949 | 8.47E-74 | 9.85E-72 |
| Dusp1 | -1.569 | 3.85E-73 | 4.44E-71 |
| Itih4 | 1.859 | 6.64E-73 | 7.61E-71 |
| Uba5 | 2.279 | 7.35E-71 | 8.31E-69 |
| Pla2g12a | 2.171 | 8.88E-71 | 9.98E-69 |
| Tmem62 | 1.836 | 1.28E-70 | 1.43E-68 |
| Kyat1 | 1.510 | 1.34E-70 | 1.48E-68 |
| Cyp2f2 | -1.296 | 2.86E-70 | 3.14E-68 |
| Acta1 | -7.005 | 2.96E-70 | 3.24E-68 |
| Fmo4 | 2.416 | 4.86E-69 | 5.24E-67 |
| Mcfd2 | 1.236 | 5.49E-68 | 5.88E-66 |
| Ces2e | -1.210 | 6.24E-68 | 6.65E-66 |
| Irf2bp2 | -1.441 | 6.97E-68 | 7.37E-66 |
| Dnajb9 | 1.452 | 3.59E-66 | 3.75E-64 |
| Srpr | 1.340 | 1.58E-65 | 1.63E-63 |
| Yipf5 | 1.561 | 5.14E-65 | 5.27E-63 |
| Creld2 | 1.629 | 8.15E-65 | 8.30E-63 |
| Rgs16 | 3.859 | 4.65E-64 | 4.71E-62 |
| Rnf125 | -1.456 | 8.41E-64 | 8.47E-62 |
| Derl1 | 1.408 | 1.05E-63 | 1.05E-61 |
| Nfil3 | -1.223 | 1.49E-63 | 1.47E-61 |
| Tmed9 | 1.565 | 1.66E-63 | 1.63E-61 |
| Gmppa | 1.958 | 1.33E-62 | 1.29E-60 |
| Ikbke | 2.048 | 2.40E-62 | 2.31E-60 |
| Ccnd1 | 2.047 | 6.59E-62 | 6.29E-60 |
| Slc17a9 | 2.878 | 1.81E-61 | 1.72E-59 |
| Armcx3 | 2.639 | 4.82E-61 | 4.55E-59 |
| Smim14 | 1.499 | 5.87E-61 | 5.51E-59 |
| Cxcl1 | 3.357 | 7.75E-60 | 7.23E-58 |
| Pmm1 | 2.575 | 1.02E-59 | 9.44E-58 |
| Irs2 | -1.224 | 3.25E-59 | 2.98E-57 |
| Alas2 | -1.056 | 1.07E-58 | 9.79E-57 |
| Edem2 | 1.530 | 1.20E-58 | 1.09E-56 |
| Npc1 | 1.309 | 1.50E-58 | 1.35E-56 |
| Plk3 | 1.508 | 2.11E-58 | 1.89E-56 |
| Acot2 | 3.097 | 3.16E-58 | 2.82E-56 |
| Soat2 | 1.718 | 6.90E-58 | 6.09E-56 |
| Msmo1 | 1.230 | 1.94E-57 | 1.70E-55 |
| Cyp2c69 | -3.765 | 9.14E-57 | 7.98E-55 |
| Hmgn2 | 1.897 | 1.38E-56 | 1.20E-54 |
| Srprb | 1.386 | 1.60E-56 | 1.38E-54 |
| Nr4a1 | -3.025 | 2.91E-56 | 2.49E-54 |
| Extl1 | 2.439 | 3.02E-56 | 2.57E-54 |
| Cyb5r1 | 2.449 | 5.01E-56 | 4.22E-54 |
| Nars | 1.244 | 3.26E-55 | 2.73E-53 |
| Ang | 1.362 | 3.69E-55 | 3.08E-53 |
| Depp1 | -1.087 | 5.29E-55 | 4.37E-53 |
| Cad | 1.807 | 6.42E-55 | 5.28E-53 |
| Fbxw9 | 2.043 | 5.06E-54 | 4.08E-52 |
| Nucb2 | 2.596 | 1.06E-53 | 8.45E-52 |
| Arf4 | 1.207 | 1.42E-53 | 1.12E-51 |
| Rab3d | 1.854 | 2.09E-53 | 1.65E-51 |
| Srp9 | 1.264 | 4.50E-53 | 3.52E-51 |
| Tmem214 | 1.459 | 7.46E-53 | 5.81E-51 |
| Ssr2 | 1.325 | 2.92E-52 | 2.25E-50 |
| Spcs2 | 1.116 | 4.85E-52 | 3.72E-50 |
| Gstm2 | 1.486 | 1.72E-51 | 1.31E-49 |
| Anxa7 | 1.077 | 3.87E-51 | 2.93E-49 |
| Gnat2 | 3.682 | 4.50E-51 | 3.39E-49 |
| Tbrg1 | 1.411 | 2.58E-50 | 1.94E-48 |
| Atp2a1 | -5.732 | 3.46E-50 | 2.58E-48 |
| Hapln4 | 2.049 | 7.98E-50 | 5.94E-48 |
| Ssr3 | 1.401 | 3.22E-49 | 2.37E-47 |
| Dpp3 | 1.405 | 9.88E-49 | 7.22E-47 |
| Atf5 | 1.569 | 1.27E-48 | 9.24E-47 |
| P4hb | 1.278 | 4.33E-48 | 3.11E-46 |
| Serpina10 | 1.307 | 4.75E-48 | 3.40E-46 |
| Rnase4 | 1.245 | 8.45E-48 | 6.01E-46 |
| Anxa2 | 1.665 | 1.43E-47 | 1.02E-45 |
| Hyou1 | 1.327 | 1.51E-47 | 1.06E-45 |
| Pabpc4 | 1.405 | 1.89E-47 | 1.33E-45 |
| Apoa4 | 1.455 | 1.94E-47 | 1.36E-45 |
| Mthfr | -1.087 | 2.02E-47 | 1.41E-45 |
| Tef | 1.240 | 2.12E-47 | 1.47E-45 |
| Gtpbp2 | 1.547 | 2.56E-47 | 1.77E-45 |
| Serpine2 | -1.498 | 4.55E-47 | 3.13E-45 |
| Npas2 | -3.700 | 5.71E-47 | 3.91E-45 |
| Ostc | 1.308 | 1.19E-46 | 8.07E-45 |
| Odc1 | 1.142 | 6.96E-46 | 4.69E-44 |
| Erlin1 | 1.264 | 8.15E-46 | 5.47E-44 |
| Gpat3 | 2.399 | 1.39E-45 | 9.25E-44 |
| Arfgap3 | 1.794 | 4.34E-45 | 2.86E-43 |
| Derl3 | 2.075 | 1.78E-44 | 1.16E-42 |
| Cyp2b9 | 4.095 | 2.22E-44 | 1.45E-42 |
| Sec24d | 1.345 | 2.60E-44 | 1.68E-42 |
| Slco1a4 | 1.848 | 5.96E-44 | 3.84E-42 |
| Fndc3b | 1.495 | 6.18E-44 | 3.97E-42 |
| Gosr2 | 1.040 | 6.96E-44 | 4.45E-42 |
| Dnajc12 | 1.778 | 7.74E-44 | 4.93E-42 |
| Gadd45a | 2.945 | 8.82E-44 | 5.58E-42 |
| Morf4l2 | 1.117 | 1.46E-43 | 9.17E-42 |
| Lss | 1.233 | 5.45E-43 | 3.39E-41 |
| Slc35b1 | 1.289 | 6.86E-43 | 4.26E-41 |
| Hdlbp | 1.014 | 1.12E-42 | 6.87E-41 |
| Slc6a12 | 1.257 | 1.23E-42 | 7.54E-41 |
| Slc17a8 | -2.287 | 1.71E-42 | 1.04E-40 |
| Pdk4 | -1.396 | 1.85E-42 | 1.12E-40 |
| Nxpe2 | -1.467 | 1.93E-42 | 1.17E-40 |
| Por | 1.453 | 5.32E-42 | 3.20E-40 |
| Slc39a11 | 1.494 | 9.00E-42 | 5.38E-40 |
| Lman1 | 1.160 | 4.24E-41 | 2.51E-39 |
| Slc3a2 | 1.527 | 4.68E-41 | 2.76E-39 |
| Sec61b | 1.274 | 5.08E-41 | 2.99E-39 |
| Hmgcs1 | 1.367 | 9.80E-41 | 5.74E-39 |
| Spcs3 | 1.599 | 1.26E-40 | 7.29E-39 |
| Marchf5 | 1.043 | 3.39E-40 | 1.93E-38 |
| Acot11 | 2.007 | 4.56E-40 | 2.58E-38 |
| Ctrb1 | -6.117 | 5.20E-40 | 2.93E-38 |
| Avpi1 | 1.595 | 6.60E-40 | 3.70E-38 |
| Slc3a1 | 1.222 | 7.19E-40 | 4.02E-38 |
| Fdps | 1.306 | 1.45E-39 | 8.05E-38 |
| Myh1 | -6.763 | 2.40E-39 | 1.32E-37 |
| Tjp3 | -1.119 | 2.68E-39 | 1.47E-37 |
| Cyp7a1 | 1.968 | 4.24E-39 | 2.32E-37 |
| Adora1 | 1.482 | 8.21E-39 | 4.42E-37 |
| Plscr2 | 1.515 | 2.67E-38 | 1.42E-36 |
| Mettl9 | 1.221 | 3.57E-38 | 1.90E-36 |
| Cyb561 | 2.030 | 4.10E-38 | 2.17E-36 |
| Mme | -2.462 | 2.16E-37 | 1.12E-35 |
| Yipf2 | 1.504 | 2.99E-37 | 1.54E-35 |
| Yif1a | 1.488 | 5.46E-37 | 2.80E-35 |
| Krtcap2 | 1.349 | 5.53E-37 | 2.83E-35 |
| Calu | 1.122 | 1.28E-36 | 6.41E-35 |
| Hmgcr | 1.132 | 2.12E-36 | 1.05E-34 |
| Ank3 | 1.573 | 3.72E-36 | 1.84E-34 |
| Slc39a7 | 1.315 | 4.97E-36 | 2.44E-34 |
| Maoa | 1.802 | 5.78E-36 | 2.83E-34 |
| Pdia4 | 1.046 | 1.71E-35 | 8.28E-34 |
| Srp72 | 1.202 | 2.11E-35 | 1.02E-33 |
| Cela1 | -1.637 | 2.69E-35 | 1.30E-33 |
| Dnajc10 | 1.446 | 6.40E-35 | 3.05E-33 |
| Asap2 | -1.154 | 8.32E-35 | 3.95E-33 |
| Nbas | 1.303 | 1.25E-34 | 5.92E-33 |
| Cope | 1.209 | 1.58E-34 | 7.41E-33 |
| Gale | 1.671 | 1.42E-33 | 6.55E-32 |
| Ift20 | 1.237 | 1.45E-33 | 6.69E-32 |
| Gnpnat1 | 1.183 | 1.77E-33 | 8.13E-32 |
| Hamp | 1.306 | 2.20E-33 | 1.01E-31 |
| Rexo2 | 1.029 | 3.86E-33 | 1.75E-31 |
| Gfpt1 | 1.634 | 4.79E-33 | 2.16E-31 |
| Arcn1 | 1.293 | 6.71E-33 | 3.01E-31 |
| Mvp | 1.325 | 7.30E-33 | 3.27E-31 |
| Tceal9 | 1.636 | 9.37E-33 | 4.17E-31 |
| Cyp4a14 | 1.062 | 1.08E-32 | 4.77E-31 |
| Gnat1 | 3.672 | 1.51E-32 | 6.65E-31 |
| Ckm | -6.689 | 1.51E-32 | 6.65E-31 |
| Nol3 | 3.797 | 1.54E-32 | 6.74E-31 |
| Slc35c2 | 1.507 | 1.64E-32 | 7.15E-31 |
| Fam114a1 | 1.551 | 1.75E-32 | 7.62E-31 |
| Sqle | 1.056 | 2.05E-32 | 8.87E-31 |
| Ypel2 | -1.081 | 2.20E-32 | 9.47E-31 |
| Dpagt1 | 1.359 | 2.41E-32 | 1.03E-30 |
| Pyroxd2 | 1.266 | 2.71E-32 | 1.16E-30 |
| Slc34a2 | -2.493 | 3.16E-32 | 1.35E-30 |
| Arf1 | 1.015 | 4.27E-32 | 1.82E-30 |
| Rint1 | 1.399 | 4.28E-32 | 1.82E-30 |
| Cdk5rap3 | 1.430 | 6.09E-32 | 2.58E-30 |
| 2010003K11Rik | 1.843 | 7.64E-32 | 3.23E-30 |
| Jpt2 | 1.059 | 1.05E-31 | 4.39E-30 |
| Htatip2 | 1.312 | 1.05E-31 | 4.39E-30 |
| Mfsd2a | 2.049 | 1.15E-31 | 4.80E-30 |
| Ddost | 1.225 | 1.18E-31 | 4.94E-30 |
| Orm3 | 4.714 | 1.61E-31 | 6.70E-30 |
| Nans | 1.687 | 1.79E-31 | 7.44E-30 |
| 6430548M08Rik | 1.493 | 3.27E-31 | 1.35E-29 |
| Gpnmb | 4.122 | 3.96E-31 | 1.63E-29 |
| Fitm1 | -1.415 | 5.85E-31 | 2.40E-29 |
| Lars | 1.337 | 1.10E-30 | 4.49E-29 |
| Cyp21a1 | -3.902 | 1.56E-30 | 6.32E-29 |
| Ttc39a | 2.954 | 1.65E-30 | 6.69E-29 |
| Thnsl2 | 1.195 | 2.28E-30 | 9.19E-29 |
| Dynll1 | 1.750 | 4.78E-30 | 1.91E-28 |
| C3 | 1.194 | 9.10E-30 | 3.60E-28 |
| Srp54c | 2.262 | 1.14E-29 | 4.48E-28 |
| Ugt2b37 | -3.246 | 1.27E-29 | 4.98E-28 |
| Ifrd2 | 1.095 | 1.48E-29 | 5.81E-28 |
| Gars | 1.011 | 1.53E-29 | 6.00E-28 |
| Gadd45g | -1.302 | 2.98E-29 | 1.16E-27 |
| Sucnr1 | -1.053 | 4.50E-29 | 1.75E-27 |
| Bfar | 1.336 | 4.76E-29 | 1.84E-27 |
| Calr | 1.111 | 6.27E-29 | 2.42E-27 |
| Fdft1 | 1.067 | 6.55E-29 | 2.53E-27 |
| Isyna1 | 2.083 | 6.72E-29 | 2.59E-27 |
| Mvk | 1.387 | 1.04E-28 | 3.97E-27 |
| Col1a1 | 1.564 | 1.18E-28 | 4.51E-27 |
| Chid1 | 1.057 | 1.83E-28 | 6.91E-27 |
| Mylpf | -3.179 | 2.60E-28 | 9.76E-27 |
| Tpm2 | -1.489 | 3.82E-28 | 1.43E-26 |
| Septin11 | 1.517 | 5.98E-28 | 2.22E-26 |
| Cfd | -2.715 | 9.03E-28 | 3.33E-26 |
| Nelfe | 1.332 | 1.43E-27 | 5.26E-26 |
| Arl4d | -1.117 | 1.86E-27 | 6.79E-26 |
| Ufm1 | 1.142 | 2.92E-27 | 1.06E-25 |
| Tspyl2 | 1.668 | 3.15E-27 | 1.14E-25 |
| Tbc1d16 | 1.295 | 4.52E-27 | 1.63E-25 |
| Fam222a | -2.131 | 5.48E-27 | 1.96E-25 |
| Pex2 | 1.141 | 9.33E-27 | 3.33E-25 |
| Igsf11 | 1.227 | 1.16E-26 | 4.12E-25 |
| Uso1 | 1.418 | 1.32E-26 | 4.69E-25 |
| Gpr146 | 1.044 | 1.52E-26 | 5.37E-25 |
| Hamp2 | 1.639 | 2.90E-26 | 1.02E-24 |
| Atf6 | 1.194 | 4.11E-26 | 1.44E-24 |
| Ssr4 | 1.146 | 4.50E-26 | 1.57E-24 |
| Tspan33 | -1.007 | 5.72E-26 | 1.99E-24 |
| Marco | 1.515 | 5.80E-26 | 2.01E-24 |
| Chil3 | 5.915 | 7.24E-26 | 2.51E-24 |
| Per3 | 1.951 | 7.73E-26 | 2.68E-24 |
| Mup13 | -2.646 | 9.91E-26 | 3.41E-24 |
| Ide | 1.053 | 1.03E-25 | 3.52E-24 |
| Cacfd1 | 1.206 | 1.35E-25 | 4.60E-24 |
| Spcs1 | 1.062 | 1.99E-25 | 6.75E-24 |
| Tsku | 1.057 | 2.55E-25 | 8.61E-24 |
| Ccl9 | 1.002 | 2.56E-25 | 8.65E-24 |
| Fads3 | 1.701 | 3.72E-25 | 1.25E-23 |
| Scfd2 | 1.749 | 4.42E-25 | 1.48E-23 |
| Parn | 1.401 | 4.99E-25 | 1.67E-23 |
| Prtn3 | 5.810 | 6.61E-25 | 2.20E-23 |
| Ddx56 | 1.309 | 8.18E-25 | 2.72E-23 |
| Rpl13a | 1.025 | 9.59E-25 | 3.17E-23 |
| S100a9 | 2.681 | 9.90E-25 | 3.27E-23 |
| Rpl12 | 1.027 | 1.13E-24 | 3.72E-23 |
| Gramd1c | 1.775 | 1.35E-24 | 4.43E-23 |
| Slc13a2 | -2.878 | 1.47E-24 | 4.81E-23 |
| Ss18l2 | 1.420 | 1.76E-24 | 5.74E-23 |
| Grk5 | -1.032 | 1.90E-24 | 6.18E-23 |
| Surf4 | 1.023 | 2.95E-24 | 9.50E-23 |
| Mettl7b | 1.078 | 4.23E-24 | 1.35E-22 |
| Tceal8 | 1.333 | 4.68E-24 | 1.49E-22 |
| Itgam | 2.187 | 8.33E-24 | 2.62E-22 |
| Srsf3 | 1.112 | 8.39E-24 | 2.64E-22 |
| Tfb1m | 1.660 | 1.18E-23 | 3.70E-22 |
| Cela2a | -3.554 | 2.04E-23 | 6.38E-22 |
| Wfs1 | 1.380 | 2.38E-23 | 7.42E-22 |
| Tox | 2.086 | 2.96E-23 | 9.21E-22 |
| Phospho1 | -1.517 | 3.67E-23 | 1.14E-21 |
| Gmds | 2.007 | 4.65E-23 | 1.44E-21 |
| Celsr1 | -1.046 | 5.47E-23 | 1.68E-21 |
| Tmem97 | 1.107 | 7.75E-23 | 2.37E-21 |
| Crym | 3.924 | 9.21E-23 | 2.81E-21 |
| Wipf3 | 1.755 | 9.23E-23 | 2.81E-21 |
| Spg21 | 1.263 | 1.25E-22 | 3.79E-21 |
| Krt18 | 1.007 | 2.05E-22 | 6.16E-21 |
| Cpa1 | -12.966 | 2.08E-22 | 6.26E-21 |
| Pomgnt1 | 1.155 | 2.93E-22 | 8.79E-21 |
| Srp19 | 1.453 | 3.92E-22 | 1.17E-20 |
| Ubxn8 | 1.114 | 5.20E-22 | 1.54E-20 |
| Slc23a1 | 1.112 | 8.61E-22 | 2.54E-20 |
| Fibin | 3.238 | 8.71E-22 | 2.56E-20 |
| Spp1 | 1.046 | 8.97E-22 | 2.64E-20 |
| Col27a1 | -1.289 | 9.87E-22 | 2.89E-20 |
| Eif4ebp1 | 1.141 | 1.12E-21 | 3.26E-20 |
| Tsen2 | 1.634 | 1.41E-21 | 4.11E-20 |
| Ccdc149 | 2.231 | 1.88E-21 | 5.47E-20 |
| Gask1a | -1.743 | 2.13E-21 | 6.15E-20 |
| Palmd | 1.134 | 2.31E-21 | 6.64E-20 |
| Asph | 1.153 | 2.52E-21 | 7.24E-20 |
| Ank | 1.003 | 2.79E-21 | 8.01E-20 |
| Mb | -7.817 | 3.91E-21 | 1.12E-19 |
| Fabp5 | 1.441 | 4.33E-21 | 1.24E-19 |
| Pard3b | 1.428 | 6.14E-21 | 1.74E-19 |
| Arhgef26 | 1.233 | 7.13E-21 | 2.01E-19 |
| Abcd2 | 1.944 | 9.90E-21 | 2.79E-19 |
| Col15a1 | 1.295 | 1.06E-20 | 2.98E-19 |
| Cyp39a1 | 1.027 | 1.37E-20 | 3.85E-19 |
| Il18bp | 1.088 | 1.44E-20 | 4.03E-19 |
| Gsta1 | 2.076 | 1.85E-20 | 5.16E-19 |
| Tmem258 | 1.591 | 2.21E-20 | 6.13E-19 |
| Cel | -12.535 | 2.32E-20 | 6.44E-19 |
| Cnppd1 | 1.052 | 2.34E-20 | 6.50E-19 |
| Atp1a2 | -2.070 | 2.58E-20 | 7.15E-19 |
| Mrpl17 | 1.136 | 2.96E-20 | 8.17E-19 |
| Try4 | -7.917 | 3.19E-20 | 8.79E-19 |
| Pnlip | -9.632 | 4.14E-20 | 1.13E-18 |
| Rusc1 | 1.865 | 4.31E-20 | 1.18E-18 |
| Il1rn | 1.994 | 5.61E-20 | 1.53E-18 |
| A2m | 4.807 | 6.58E-20 | 1.79E-18 |
| Tmem120a | 1.025 | 7.69E-20 | 2.08E-18 |
| Mapk15 | -1.620 | 8.06E-20 | 2.18E-18 |
| Pstpip2 | 1.308 | 9.14E-20 | 2.47E-18 |
| Cep85 | 1.088 | 9.36E-20 | 2.53E-18 |
| Pck2 | 1.708 | 1.03E-19 | 2.78E-18 |
| Ccl6 | 1.179 | 1.52E-19 | 4.05E-18 |
| Kars | 1.005 | 1.66E-19 | 4.41E-18 |
| Slc20a1 | 1.052 | 1.72E-19 | 4.58E-18 |
| Ctrl | -12.660 | 2.38E-19 | 6.28E-18 |
| Hsd3b1 | -9.323 | 2.76E-19 | 7.24E-18 |
| Amy2a1 | -11.137 | 3.18E-19 | 8.33E-18 |
| Gstp3 | 1.625 | 4.39E-19 | 1.14E-17 |
| Aldh1l2 | 3.878 | 4.46E-19 | 1.16E-17 |
| 2210010C04Rik | -12.224 | 4.78E-19 | 1.24E-17 |
| Arhgef2 | 1.165 | 5.13E-19 | 1.33E-17 |
| Hspa13 | 1.555 | 5.37E-19 | 1.38E-17 |
| Mrps18b | 1.003 | 5.78E-19 | 1.49E-17 |
| Pygm | -2.368 | 6.10E-19 | 1.57E-17 |
| Lpin3 | 1.894 | 8.21E-19 | 2.09E-17 |
| Fus | 1.226 | 1.01E-18 | 2.56E-17 |
| Star | -2.850 | 1.18E-18 | 2.98E-17 |
| Try5 | -14.136 | 1.34E-18 | 3.37E-17 |
| Fst | -1.621 | 1.55E-18 | 3.88E-17 |
| Prss2 | -13.538 | 1.89E-18 | 4.71E-17 |
| Mettl1 | 1.731 | 2.01E-18 | 5.00E-17 |
| Ccdc167 | 1.244 | 2.14E-18 | 5.30E-17 |
| Slc5a6 | 1.774 | 2.18E-18 | 5.40E-17 |
| Slc16a5 | 1.560 | 2.22E-18 | 5.49E-17 |
| Slco1a1 | -1.844 | 2.69E-18 | 6.63E-17 |
| Adgrf1 | -1.050 | 2.75E-18 | 6.77E-17 |
| Tenm3 | 1.075 | 2.84E-18 | 6.97E-17 |
| Cela3b | -12.359 | 3.32E-18 | 8.12E-17 |
| Lmna | 1.060 | 3.39E-18 | 8.26E-17 |
| Nat9 | 1.178 | 4.40E-18 | 1.07E-16 |
| Smim7 | 1.120 | 5.11E-18 | 1.23E-16 |
| Trim46 | 4.095 | 5.12E-18 | 1.23E-16 |
| Angptl6 | 1.322 | 5.63E-18 | 1.35E-16 |
| Syt1 | 1.558 | 5.69E-18 | 1.36E-16 |
| Hid1 | 1.877 | 6.85E-18 | 1.63E-16 |
| Pitx3 | -8.768 | 6.90E-18 | 1.64E-16 |
| Ppp1r1b | -1.520 | 7.21E-18 | 1.71E-16 |
| Lgr5 | -1.100 | 1.21E-17 | 2.85E-16 |
| Tapbpl | 1.076 | 1.43E-17 | 3.36E-16 |
| Gm3776 | 1.861 | 1.96E-17 | 4.54E-16 |
| Cgref1 | 4.196 | 2.73E-17 | 6.27E-16 |
| Atp6v0d2 | 3.177 | 3.13E-17 | 7.13E-16 |
| Ckmt2 | -11.223 | 3.17E-17 | 7.21E-16 |
| Rnase1 | -12.054 | 3.40E-17 | 7.70E-16 |
| Per1 | 1.224 | 3.45E-17 | 7.80E-16 |
| Dhrs7 | 1.045 | 3.68E-17 | 8.31E-16 |
| Ppl | 1.355 | 4.41E-17 | 9.94E-16 |
| Nup210 | 1.128 | 5.16E-17 | 1.15E-15 |
| 1810058I24Rik | 1.168 | 6.66E-17 | 1.49E-15 |
| Paip2b | 1.013 | 7.59E-17 | 1.69E-15 |
| Acot3 | 1.365 | 7.70E-17 | 1.71E-15 |
| Pnliprp1 | -11.897 | 8.94E-17 | 1.98E-15 |
| Tnnt3 | -7.845 | 8.99E-17 | 1.99E-15 |
| Nudcd2 | 1.101 | 1.25E-16 | 2.74E-15 |
| Smox | 1.674 | 1.36E-16 | 2.96E-15 |
| Cpb1 | -10.129 | 1.38E-16 | 3.00E-15 |
| A2ml1 | 1.305 | 1.51E-16 | 3.27E-15 |
| Golga1 | 1.361 | 1.70E-16 | 3.69E-15 |
| Leap2 | 1.218 | 1.72E-16 | 3.71E-15 |
| Dhx40 | 1.159 | 1.92E-16 | 4.15E-15 |
| Nmral1 | 1.526 | 2.30E-16 | 4.96E-15 |
| Gdf15 | 1.728 | 2.78E-16 | 5.95E-15 |
| Myh4 | -9.015 | 2.83E-16 | 6.06E-15 |
| Ppp2r3c | 1.362 | 3.61E-16 | 7.68E-15 |
| Cyp11b1 | -9.929 | 3.81E-16 | 8.08E-15 |
| Ermp1 | 1.012 | 4.65E-16 | 9.82E-15 |
| Cd63 | 1.435 | 4.84E-16 | 1.02E-14 |
| Srrm4 | 2.270 | 5.41E-16 | 1.14E-14 |
| Rhbdd1 | 1.071 | 5.66E-16 | 1.18E-14 |
| Cp | 1.167 | 5.88E-16 | 1.23E-14 |
| Slc22a27 | 2.892 | 6.14E-16 | 1.28E-14 |
| Myl1 | -11.602 | 1.00E-15 | 2.06E-14 |
| Arsg | 1.063 | 1.19E-15 | 2.45E-14 |
| Ubxn4 | 1.178 | 1.22E-15 | 2.50E-14 |
| Slc41a3 | 2.234 | 1.27E-15 | 2.60E-14 |
| Trmt6 | 1.290 | 1.73E-15 | 3.51E-14 |
| Eif2d | 1.101 | 2.02E-15 | 4.07E-14 |
| Myh2 | -7.535 | 3.02E-15 | 6.05E-14 |
| Cpa2 | -11.102 | 3.47E-15 | 6.92E-14 |
| Smyd5 | 1.294 | 3.59E-15 | 7.15E-14 |
| Timp1 | 4.699 | 3.78E-15 | 7.50E-14 |
| Cyp11a1 | -8.811 | 3.92E-15 | 7.76E-14 |
| Syt12 | 2.385 | 3.93E-15 | 7.77E-14 |
| Clps | -12.081 | 4.45E-15 | 8.74E-14 |
| Ipo4 | 1.100 | 5.65E-15 | 1.10E-13 |
| Nfkbiz | 1.286 | 5.73E-15 | 1.12E-13 |
| Sys1 | 1.065 | 6.18E-15 | 1.20E-13 |
| Olig1 | -1.925 | 6.34E-15 | 1.23E-13 |
| St7 | 1.314 | 6.85E-15 | 1.32E-13 |
| Them7 | 1.163 | 7.25E-15 | 1.40E-13 |
| Aig1 | 1.102 | 7.47E-15 | 1.43E-13 |
| Ifi27l2b | 1.639 | 8.46E-15 | 1.61E-13 |
| Aimp1 | 1.042 | 1.03E-14 | 1.95E-13 |
| Rhbg | -1.049 | 1.15E-14 | 2.18E-13 |
| Vmn2r3 | 9.355 | 1.20E-14 | 2.26E-13 |
| Aprt | 1.199 | 1.29E-14 | 2.44E-13 |
| Cyp1a1 | 2.161 | 1.36E-14 | 2.55E-13 |
| Socs3 | 1.247 | 1.38E-14 | 2.59E-13 |
| Dnajc3 | 1.129 | 2.48E-14 | 4.58E-13 |
| Usp2 | 1.203 | 2.51E-14 | 4.63E-13 |
| Cpne8 | 1.796 | 2.57E-14 | 4.73E-13 |
| Iffo2 | 1.068 | 2.59E-14 | 4.76E-13 |
| Cyp20a1 | 1.138 | 2.64E-14 | 4.85E-13 |
| Gpcpd1 | 1.308 | 3.09E-14 | 5.66E-13 |
| Lgals3 | 1.290 | 3.99E-14 | 7.25E-13 |
| Nqo1 | 1.349 | 4.12E-14 | 7.47E-13 |
| Nabp1 | 1.540 | 4.25E-14 | 7.70E-13 |
| Loxl4 | -1.634 | 4.39E-14 | 7.92E-13 |
| Lrtm1 | 1.972 | 5.91E-14 | 1.05E-12 |
| Myc | 1.683 | 6.55E-14 | 1.17E-12 |
| Trmt61a | 1.627 | 9.17E-14 | 1.62E-12 |
| Obscn | -4.970 | 1.34E-13 | 2.34E-12 |
| Pdilt | -1.096 | 1.53E-13 | 2.68E-12 |
| Srp54a | 1.959 | 1.59E-13 | 2.78E-12 |
| F13a1 | 3.569 | 1.77E-13 | 3.06E-12 |
| Slc18a1 | 1.264 | 1.93E-13 | 3.34E-12 |
| Ung | 1.852 | 2.13E-13 | 3.67E-12 |
| Fam47e | 1.174 | 2.14E-13 | 3.70E-12 |
| Timm21 | 1.074 | 2.27E-13 | 3.91E-12 |
| Chac1 | 2.108 | 2.59E-13 | 4.42E-12 |
| Mknk1 | 1.007 | 2.70E-13 | 4.60E-12 |
| Eprs | 1.069 | 2.72E-13 | 4.63E-12 |
| Zg16 | -11.483 | 2.84E-13 | 4.81E-12 |
| Coq8b | 1.094 | 3.28E-13 | 5.54E-12 |
| Pnliprp2 | -10.288 | 3.85E-13 | 6.49E-12 |
| Abcb1b | -1.807 | 4.65E-13 | 7.81E-12 |
| Gm33543 | 1.472 | 5.01E-13 | 8.40E-12 |
| Tvp23b | 1.071 | 5.28E-13 | 8.81E-12 |
| Gpatch4 | 1.226 | 6.79E-13 | 1.13E-11 |
| Aen | 1.010 | 7.06E-13 | 1.17E-11 |
| 9130409I23Rik | 1.276 | 7.94E-13 | 1.31E-11 |
| S100a8 | 2.495 | 9.55E-13 | 1.57E-11 |
| Chga | -9.969 | 9.99E-13 | 1.64E-11 |
| Golt1a | 1.318 | 1.23E-12 | 2.00E-11 |
| Snx10 | 1.150 | 1.24E-12 | 2.02E-11 |
| Slpi | 1.986 | 1.42E-12 | 2.31E-11 |
| Lama3 | -1.320 | 1.50E-12 | 2.44E-11 |
| Pfkfb3 | 1.020 | 1.81E-12 | 2.91E-11 |
| Vldlr | 2.104 | 1.99E-12 | 3.19E-11 |
| Fam118a | 2.311 | 2.08E-12 | 3.33E-11 |
| Nrg4 | 1.842 | 2.29E-12 | 3.65E-11 |
| Wee1 | 1.355 | 2.56E-12 | 4.06E-11 |
| Nphp1 | 1.971 | 2.61E-12 | 4.14E-11 |
| S100a11 | 1.575 | 2.91E-12 | 4.59E-11 |
| Rbmx | 1.193 | 3.59E-12 | 5.64E-11 |
| Get4 | 1.213 | 4.01E-12 | 6.27E-11 |
| Ddit4 | 1.243 | 4.14E-12 | 6.46E-11 |
| Heyl | -1.605 | 4.41E-12 | 6.86E-11 |
| Mybpc1 | -8.740 | 5.21E-12 | 8.04E-11 |
| Tnnc2 | -6.862 | 5.84E-12 | 9.00E-11 |
| Tes | 1.723 | 5.86E-12 | 9.02E-11 |
| Mmp12 | 3.590 | 5.86E-12 | 9.02E-11 |
| Tmprss2 | 1.421 | 6.02E-12 | 9.25E-11 |
| Il13ra1 | 1.054 | 6.17E-12 | 9.48E-11 |
| Tnni2 | -6.292 | 6.55E-12 | 1.00E-10 |
| Ric8a | 1.008 | 6.78E-12 | 1.04E-10 |
| Nrap | -3.147 | 7.73E-12 | 1.17E-10 |
| Wnk4 | -1.483 | 8.09E-12 | 1.23E-10 |
| Glt8d1 | 1.441 | 8.28E-12 | 1.25E-10 |
| Slc37a1 | 1.979 | 1.20E-11 | 1.80E-10 |
| Klk1 | -5.166 | 1.24E-11 | 1.86E-10 |
| Acsl4 | 1.174 | 1.24E-11 | 1.86E-10 |
| Elmod2 | 1.317 | 1.25E-11 | 1.87E-10 |
| Alpk1 | 2.615 | 1.30E-11 | 1.94E-10 |
| Bckdk | 1.046 | 1.30E-11 | 1.94E-10 |
| Tcea1 | 1.028 | 1.60E-11 | 2.37E-10 |
| Ica1 | 1.868 | 2.80E-11 | 4.05E-10 |
| Ttn | -4.322 | 2.83E-11 | 4.08E-10 |
| Prss3 | -10.558 | 2.84E-11 | 4.09E-10 |
| Acpp | 1.445 | 2.84E-11 | 4.10E-10 |
| Ryr1 | -3.358 | 2.90E-11 | 4.17E-10 |
| Tmem184c | 1.194 | 3.08E-11 | 4.41E-10 |
| Adamtsl2 | -1.058 | 3.71E-11 | 5.27E-10 |
| Dusp12 | 1.223 | 3.76E-11 | 5.34E-10 |
| Nipal1 | 2.066 | 4.31E-11 | 6.10E-10 |
| Tex30 | 1.468 | 4.91E-11 | 6.90E-10 |
| Scfd1 | 1.420 | 5.56E-11 | 7.78E-10 |
| Sycn | -11.263 | 6.13E-11 | 8.54E-10 |
| Ciart | 2.683 | 6.40E-11 | 8.89E-10 |
| Ermard | 1.249 | 6.53E-11 | 9.06E-10 |
| Myh7 | -6.966 | 7.64E-11 | 1.05E-09 |
| Gp2 | -3.235 | 8.98E-11 | 1.24E-09 |
| Rbis | 1.251 | 9.59E-11 | 1.32E-09 |
| Tcap | -5.656 | 9.99E-11 | 1.37E-09 |
| Fibp | 1.057 | 1.03E-10 | 1.41E-09 |
| Rhbdd2 | 1.037 | 1.26E-10 | 1.71E-09 |
| Alg12 | 1.251 | 1.27E-10 | 1.72E-09 |
| Zfp330 | 1.187 | 1.43E-10 | 1.92E-09 |
| Tasp1 | 1.828 | 1.43E-10 | 1.92E-09 |
| Myot | -9.103 | 1.50E-10 | 2.02E-09 |
| Ubiad1 | 1.108 | 1.57E-10 | 2.11E-09 |
| Smpd3 | 1.708 | 1.62E-10 | 2.16E-09 |
| Pgm2 | 1.204 | 1.75E-10 | 2.33E-09 |
| Aatk | -1.312 | 1.98E-10 | 2.64E-09 |
| Abracl | 1.314 | 2.05E-10 | 2.72E-09 |
| Impdh1 | 1.393 | 2.31E-10 | 3.06E-09 |
| Cdin1 | 1.648 | 3.04E-10 | 3.96E-09 |
| Amy2a3 | -11.348 | 3.27E-10 | 4.24E-09 |
| Amy2a2 | -11.348 | 3.27E-10 | 4.24E-09 |
| Amy2a4 | -11.348 | 3.27E-10 | 4.24E-09 |
| Sec61g | 1.140 | 3.56E-10 | 4.61E-09 |
| Marveld2 | 1.075 | 4.30E-10 | 5.52E-09 |
| Eef1akmt3 | 8.951 | 4.56E-10 | 5.83E-09 |
| Csf3r | 1.495 | 5.05E-10 | 6.44E-09 |
| Pvalb | -9.849 | 6.68E-10 | 8.46E-09 |
| Ufl1 | 1.124 | 7.51E-10 | 9.44E-09 |
| Stard3nl | 1.178 | 7.83E-10 | 9.85E-09 |
| Ddx52 | 1.085 | 8.75E-10 | 1.09E-08 |
| Alg6 | 1.451 | 9.10E-10 | 1.14E-08 |
| Hspb7 | -2.894 | 9.36E-10 | 1.17E-08 |
| Adam8 | 2.907 | 9.41E-10 | 1.17E-08 |
| Thbs1 | 1.410 | 9.82E-10 | 1.22E-08 |
| Reg1 | -2.488 | 1.00E-09 | 1.24E-08 |
| Copz2 | 1.087 | 1.11E-09 | 1.37E-08 |
| Fabp3 | -6.284 | 1.16E-09 | 1.43E-08 |
| Sult2a7 | 2.603 | 1.21E-09 | 1.49E-08 |
| Actn2 | -5.217 | 1.38E-09 | 1.70E-08 |
| Ildr2 | 1.082 | 1.44E-09 | 1.76E-08 |
| Lonrf3 | -1.653 | 1.60E-09 | 1.96E-08 |
| Gm7694 | 1.279 | 1.75E-09 | 2.13E-08 |
| Zfp9 | 1.678 | 1.77E-09 | 2.16E-08 |
| Ppp1r14b | 1.029 | 1.85E-09 | 2.25E-08 |
| Krcc1 | 1.295 | 2.11E-09 | 2.55E-08 |
| Aptx | 1.074 | 2.23E-09 | 2.69E-08 |
| Slc22a26 | 2.292 | 2.23E-09 | 2.69E-08 |
| Sult2a8 | -1.331 | 2.34E-09 | 2.81E-08 |
| Klf10 | 1.034 | 2.46E-09 | 2.95E-08 |
| Grem2 | 1.582 | 2.52E-09 | 3.02E-08 |
| Slc13a5 | 1.608 | 2.52E-09 | 3.02E-08 |
| Nudt18 | 1.171 | 2.57E-09 | 3.07E-08 |
| Igfbp6 | -1.237 | 2.60E-09 | 3.11E-08 |
| Rap1gap2 | 1.679 | 2.65E-09 | 3.17E-08 |
| Car1 | -1.207 | 2.66E-09 | 3.17E-08 |
| Hsp90b1 | 1.422 | 2.82E-09 | 3.36E-08 |
| Dmac2l | 1.525 | 3.09E-09 | 3.66E-08 |
| Sytl1 | 1.553 | 3.26E-09 | 3.85E-08 |
| Zdhhc12 | 1.034 | 3.95E-09 | 4.64E-08 |
| Dclre1a | 1.081 | 4.28E-09 | 5.00E-08 |
| Pla2g7 | 1.048 | 4.50E-09 | 5.25E-08 |
| Tbc1d31 | 1.074 | 4.56E-09 | 5.31E-08 |
| Rad51b | 2.961 | 4.70E-09 | 5.46E-08 |
| Hectd2 | -1.235 | 4.79E-09 | 5.56E-08 |
| Ccdc47 | 1.017 | 4.94E-09 | 5.73E-08 |
| Chgb | -9.630 | 5.42E-09 | 6.25E-08 |
| C2cd5 | 1.193 | 7.00E-09 | 8.00E-08 |
| Eef1a2 | -5.797 | 7.36E-09 | 8.37E-08 |
| Cyp26b1 | 1.165 | 7.84E-09 | 8.92E-08 |
| Cmss1 | 1.639 | 7.86E-09 | 8.94E-08 |
| Gm8113 | 2.613 | 9.30E-09 | 1.05E-07 |
| Ighg1 | 1.900 | 9.95E-09 | 1.11E-07 |
| Shc4 | 3.652 | 1.12E-08 | 1.25E-07 |
| Adipoq | -3.493 | 1.13E-08 | 1.25E-07 |
| Mospd1 | 1.142 | 1.22E-08 | 1.35E-07 |
| Flt3l | 1.653 | 1.30E-08 | 1.42E-07 |
| Pvr | 1.103 | 1.38E-08 | 1.51E-07 |
| Reg2 | -3.198 | 1.42E-08 | 1.55E-07 |
| Rsad1 | 1.397 | 1.42E-08 | 1.55E-07 |
| Ctbs | 1.071 | 1.57E-08 | 1.71E-07 |
| Srl | -1.616 | 1.76E-08 | 1.91E-07 |
| 1810009J06Rik | -9.926 | 1.78E-08 | 1.92E-07 |
| Polr3gl | 1.129 | 1.88E-08 | 2.03E-07 |
| Tmtc2 | 1.154 | 1.99E-08 | 2.13E-07 |
| Ctps2 | 1.101 | 2.04E-08 | 2.19E-07 |
| Nagk | 1.045 | 2.09E-08 | 2.23E-07 |
| Slc39a10 | -1.392 | 2.13E-08 | 2.27E-07 |
| Ctps | 1.156 | 2.18E-08 | 2.32E-07 |
| Nsmce1 | 1.010 | 2.18E-08 | 2.32E-07 |
| Rassf1 | 1.072 | 2.19E-08 | 2.33E-07 |
| Cib3 | -2.743 | 2.19E-08 | 2.33E-07 |
| Plin1 | -3.524 | 2.22E-08 | 2.36E-07 |
| Myh14 | 1.060 | 2.49E-08 | 2.63E-07 |
| Eef2kmt | 1.151 | 2.52E-08 | 2.66E-07 |
| Ankrd23 | -1.757 | 2.57E-08 | 2.71E-07 |
| Serpina9 | -1.973 | 3.16E-08 | 3.31E-07 |
| Amn1 | 1.657 | 3.25E-08 | 3.41E-07 |
| 1110065P20Rik | 1.046 | 3.34E-08 | 3.49E-07 |
| Clstn3 | -1.185 | 3.54E-08 | 3.69E-07 |
| Mtln | 1.348 | 3.74E-08 | 3.89E-07 |
| Spock2 | -1.684 | 4.17E-08 | 4.33E-07 |
| Treh | 1.924 | 4.22E-08 | 4.38E-07 |
| Hhipl2 | 3.700 | 4.83E-08 | 4.99E-07 |
| Dct | -1.541 | 4.94E-08 | 5.10E-07 |
| Alkbh6 | 1.243 | 5.10E-08 | 5.26E-07 |
| Smo | 1.020 | 5.34E-08 | 5.50E-07 |
| Casq1 | -3.922 | 5.75E-08 | 5.91E-07 |
| Gm34653 | 8.959 | 5.85E-08 | 6.00E-07 |
| Chil1 | 2.872 | 6.18E-08 | 6.33E-07 |
| Cspg5 | -1.188 | 6.83E-08 | 6.93E-07 |
| Ccr1 | 2.852 | 8.00E-08 | 8.03E-07 |
| Dusp23 | 1.053 | 8.03E-08 | 8.05E-07 |
| Aimp2 | 1.030 | 9.10E-08 | 9.05E-07 |
| Idi1 | 1.084 | 9.36E-08 | 9.30E-07 |
| Fos | -1.400 | 9.61E-08 | 9.54E-07 |
| Chrnb2 | -1.164 | 1.09E-07 | 1.07E-06 |
| Cmya5 | -5.129 | 1.12E-07 | 1.11E-06 |
| N6amt1 | 1.191 | 1.20E-07 | 1.18E-06 |
| Myl2 | -9.723 | 1.22E-07 | 1.19E-06 |
| Fmod | -1.057 | 1.25E-07 | 1.22E-06 |
| Myl3 | -9.293 | 1.42E-07 | 1.37E-06 |
| Morc4 | 1.822 | 1.51E-07 | 1.46E-06 |
| Gbp10 | -1.018 | 1.65E-07 | 1.59E-06 |
| Tmem181a | 1.072 | 1.66E-07 | 1.59E-06 |
| Ldb3 | -5.960 | 1.74E-07 | 1.66E-06 |
| Eef1akmt4 | 1.165 | 1.83E-07 | 1.74E-06 |
| Ptgfrn | 1.117 | 1.87E-07 | 1.78E-06 |
| Gm6614 | 5.739 | 2.02E-07 | 1.92E-06 |
| Tmem182 | -7.589 | 2.09E-07 | 1.98E-06 |
| Prkn | 1.836 | 2.21E-07 | 2.08E-06 |
| BC024139 | 1.302 | 2.41E-07 | 2.25E-06 |
| Gm5771 | -9.477 | 2.44E-07 | 2.27E-06 |
| Amigo2 | 1.039 | 2.48E-07 | 2.31E-06 |
| Chpf | 1.164 | 3.38E-07 | 3.09E-06 |
| Dbh | -7.731 | 3.41E-07 | 3.12E-06 |
| Emp1 | 1.307 | 3.42E-07 | 3.13E-06 |
| Cacna1s | -4.954 | 3.59E-07 | 3.26E-06 |
| Zfp324 | 1.107 | 4.02E-07 | 3.62E-06 |
| Tmem39a | 1.007 | 4.47E-07 | 4.02E-06 |
| Ngp | 2.234 | 4.90E-07 | 4.38E-06 |
| Pycr1 | 2.337 | 5.06E-07 | 4.50E-06 |
| Myoz1 | -8.531 | 5.06E-07 | 4.51E-06 |
| Eif2b3 | 1.010 | 5.26E-07 | 4.67E-06 |
| Cwc27 | 1.574 | 5.45E-07 | 4.84E-06 |
| B3galt1 | 1.022 | 5.57E-07 | 4.94E-06 |
| Chchd6 | 1.545 | 5.62E-07 | 4.98E-06 |
| Mthfd1l | 1.765 | 5.90E-07 | 5.21E-06 |
| Scand1 | 1.055 | 5.92E-07 | 5.22E-06 |
| Mthfsd | 1.013 | 6.26E-07 | 5.51E-06 |
| Bcap29 | 1.221 | 6.31E-07 | 5.55E-06 |
| Cenpm | 3.023 | 6.46E-07 | 5.66E-06 |
| Ppp3cc | 1.404 | 6.47E-07 | 5.67E-06 |
| Mgarp | -8.617 | 6.93E-07 | 6.05E-06 |
| Cks1b | 1.152 | 6.99E-07 | 6.10E-06 |
| Amy2a5 | -10.576 | 7.05E-07 | 6.15E-06 |
| Pdia2 | -8.077 | 7.39E-07 | 6.43E-06 |
| Endod1 | -1.183 | 7.97E-07 | 6.91E-06 |
| Xlr3a | 1.065 | 1.05E-06 | 8.97E-06 |
| Areg | 8.951 | 1.14E-06 | 9.63E-06 |
| Elac1 | 1.018 | 1.34E-06 | 1.12E-05 |
| Cabyr | -1.409 | 1.39E-06 | 1.17E-05 |
| Sirpb1c | 2.930 | 1.40E-06 | 1.17E-05 |
| Polr3d | 1.067 | 1.62E-06 | 1.35E-05 |
| Scn4b | -5.555 | 1.74E-06 | 1.44E-05 |
| Fam172a | 1.162 | 1.86E-06 | 1.54E-05 |
| Ces2b | -2.426 | 1.92E-06 | 1.58E-05 |
| Phgdh | 1.481 | 1.99E-06 | 1.63E-05 |
| Cep41 | 1.111 | 2.07E-06 | 1.70E-05 |
| Ripor2 | 1.556 | 2.24E-06 | 1.83E-05 |
| Gabrb3 | -1.504 | 2.29E-06 | 1.87E-05 |
| Gins2 | 1.375 | 2.31E-06 | 1.88E-05 |
| Alg8 | 1.090 | 2.52E-06 | 2.04E-05 |
| Lrp2bp | -1.563 | 2.68E-06 | 2.17E-05 |
| Cd14 | 1.102 | 2.75E-06 | 2.22E-05 |
| Miip | 1.073 | 2.90E-06 | 2.33E-05 |
| Art3 | -1.055 | 2.95E-06 | 2.38E-05 |
| Zbtb7c | -1.051 | 3.01E-06 | 2.42E-05 |
| Vcan | 3.322 | 3.27E-06 | 2.62E-05 |
| Pgam2 | -3.589 | 3.36E-06 | 2.69E-05 |
| Actn3 | -5.170 | 3.46E-06 | 2.76E-05 |
| Tdp1 | 1.261 | 3.54E-06 | 2.82E-05 |
| Meiob | 1.301 | 3.54E-06 | 2.82E-05 |
| Odr4 | 1.695 | 3.87E-06 | 3.07E-05 |
| Smim8 | 1.033 | 4.24E-06 | 3.34E-05 |
| Dqx1 | 1.358 | 5.66E-06 | 4.39E-05 |
| Nrip2 | 2.893 | 5.67E-06 | 4.39E-05 |
| Mfap4 | -1.325 | 5.81E-06 | 4.49E-05 |
| Spata22 | 1.511 | 5.91E-06 | 4.56E-05 |
| Lrrc15 | -2.495 | 5.99E-06 | 4.62E-05 |
| Pla2g1b | -9.684 | 6.17E-06 | 4.74E-05 |
| 1810046K07Rik | 2.824 | 6.23E-06 | 4.79E-05 |
| Pdzrn3 | 1.005 | 6.42E-06 | 4.92E-05 |
| Wasf1 | -1.092 | 6.79E-06 | 5.19E-05 |
| Ighg2b | 1.187 | 6.92E-06 | 5.28E-05 |
| Pdgfa | 1.318 | 7.27E-06 | 5.50E-05 |
| Plk5 | -1.617 | 7.27E-06 | 5.50E-05 |
| D130043K22Rik | 1.396 | 7.31E-06 | 5.53E-05 |
| Gm10334 | -8.830 | 7.34E-06 | 5.55E-05 |
| Akr1cl | -7.679 | 8.39E-06 | 6.28E-05 |
| Mybpc2 | -2.933 | 9.20E-06 | 6.82E-05 |
| Trdmt1 | 1.195 | 9.48E-06 | 7.02E-05 |
| Mmp9 | 1.463 | 1.00E-05 | 7.36E-05 |
| Slc15a5 | -1.095 | 1.03E-05 | 7.53E-05 |
| Tlr13 | 1.551 | 1.04E-05 | 7.65E-05 |
| Unc93a2 | 1.220 | 1.07E-05 | 7.85E-05 |
| Flvcr2 | 1.102 | 1.08E-05 | 7.90E-05 |
| Retnlg | 2.106 | 1.16E-05 | 8.44E-05 |
| Xirp2 | -5.714 | 1.25E-05 | 9.08E-05 |
| Zswim7 | 1.016 | 1.26E-05 | 9.12E-05 |
| Sybu | 3.650 | 1.26E-05 | 9.13E-05 |
| Angel1 | 1.040 | 1.28E-05 | 9.31E-05 |
| Slc7a11 | 5.700 | 1.45E-05 | 0.000104028 |
| Prelid2 | 3.749 | 1.48E-05 | 0.000106208 |
| Eif4e3 | 1.099 | 1.56E-05 | 0.0001119 |
| Myom1 | -1.064 | 1.75E-05 | 0.000123806 |
| Fbxo36 | 1.179 | 1.79E-05 | 0.000125996 |
| Nos1ap | 1.369 | 1.89E-05 | 0.000132519 |
| Cd4 | -1.244 | 1.96E-05 | 0.000136847 |
| Scg2 | -5.061 | 1.96E-05 | 0.000137349 |
| Atl1 | 1.540 | 2.01E-05 | 0.000140547 |
| Rpl3l | -4.787 | 2.11E-05 | 0.000146353 |
| Limd1 | 1.251 | 2.17E-05 | 0.000150558 |
| Wnt5b | 1.061 | 2.28E-05 | 0.000157949 |
| Atg10 | 1.239 | 2.30E-05 | 0.000159071 |
| Th | -7.845 | 2.58E-05 | 0.000176631 |
| Ucp1 | -3.737 | 2.60E-05 | 0.000177901 |
| Wdyhv1 | 1.024 | 2.62E-05 | 0.000179281 |
| Isg20 | 1.176 | 2.65E-05 | 0.000180861 |
| Klk1b4 | -1.121 | 2.76E-05 | 0.000188357 |
| Tha1 | 1.142 | 3.12E-05 | 0.00021085 |
| Kcnk9 | -4.644 | 3.37E-05 | 0.000226582 |
| Hrc | -3.600 | 3.64E-05 | 0.000243461 |
| Mppe1 | 1.032 | 3.83E-05 | 0.000255186 |
| Slc22a3 | 1.083 | 4.08E-05 | 0.000270849 |
| Plek | 1.083 | 4.14E-05 | 0.000274487 |
| Ergic2 | 1.029 | 4.18E-05 | 0.000276662 |
| Fam83f | 1.830 | 4.46E-05 | 0.000294305 |
| Utp14b | 1.320 | 4.50E-05 | 0.000296749 |
| Slc22a4 | 1.380 | 4.52E-05 | 0.000297798 |
| Xrcc4 | 1.047 | 4.66E-05 | 0.000306276 |
| Mphosph10 | 1.050 | 5.45E-05 | 0.000353217 |
| Cxcr2 | 1.976 | 5.46E-05 | 0.000353893 |
| Smtnl1 | -7.322 | 6.03E-05 | 0.000387237 |
| Myoz2 | -7.861 | 6.23E-05 | 0.000399124 |
| Hapln1 | -3.129 | 6.28E-05 | 0.000402306 |
| Catsper2 | 1.633 | 7.17E-05 | 0.000455171 |
| Thbs4 | -3.369 | 7.50E-05 | 0.000474791 |
| Tbc1d19 | 1.366 | 8.42E-05 | 0.000528365 |
| Pkd1 | 1.070 | 8.74E-05 | 0.000546599 |
| Mcm5 | 1.286 | 9.04E-05 | 0.000564668 |
| S100a4 | 1.949 | 9.47E-05 | 0.000588937 |
| Cdt1 | 1.284 | 9.52E-05 | 0.000591757 |
| Zfp386 | 1.262 | 9.56E-05 | 0.000594111 |
| Cxcr6 | -1.435 | 0.000100089 | 0.000619598 |
| Gucy2c | 1.966 | 0.000103391 | 0.000638601 |
| Klhl31 | -4.459 | 0.00010436 | 0.000644348 |
| Mmp8 | 2.555 | 0.000106623 | 0.00065562 |
| Stag1 | 1.024 | 0.000108139 | 0.00066371 |
| Cfh | 1.141 | 0.000111772 | 0.000683462 |
| Ak1 | -1.556 | 0.000111835 | 0.000683596 |
| Klhl41 | -2.439 | 0.000115845 | 0.000705497 |
| Ppm1h | 1.574 | 0.000121206 | 0.000735975 |
| Vangl1 | 1.010 | 0.000128406 | 0.000777688 |
| Serpine1 | 1.838 | 0.000137316 | 0.000827402 |
| Smco4 | 1.147 | 0.000149325 | 0.000894868 |
| Uhrf1 | 1.565 | 0.000155314 | 0.000927723 |
| Dpep2 | 4.366 | 0.000159223 | 0.000948673 |
| Rec8 | -1.058 | 0.000159284 | 0.000948696 |
| Perm1 | -2.655 | 0.000160868 | 0.000956745 |
| Fen1 | 1.040 | 0.000162297 | 0.000964901 |
| 3425401B19Rik | -4.585 | 0.000163305 | 0.000969844 |
| Ccl7 | 3.635 | 0.000163707 | 0.000971881 |
| Siva1 | 1.034 | 0.00017643 | 0.001042543 |
| Irak3 | 1.324 | 0.000183119 | 0.001077822 |
| Fam110c | 1.599 | 0.000186984 | 0.001097831 |
| Dync2i2 | 1.020 | 0.000190047 | 0.001113047 |
| Alox5ap | 1.039 | 0.000201599 | 0.001174867 |
| Klf1 | 1.389 | 0.000226775 | 0.001307795 |
| E130311K13Rik | 1.048 | 0.000227447 | 0.001310239 |
| Ssc4d | 1.360 | 0.00023196 | 0.001332518 |
| Art1 | -7.322 | 0.000235262 | 0.001350079 |
| Smpx | -3.552 | 0.000237596 | 0.00136205 |
| Akr1c21 | -7.551 | 0.000239926 | 0.001373501 |
| Faap100 | 1.073 | 0.000243959 | 0.001395623 |
| C5ar1 | 1.058 | 0.000244893 | 0.00140048 |
| Mup6 | -2.258 | 0.000248745 | 0.001420539 |
| Rab11fip5 | 1.267 | 0.000249795 | 0.00142555 |
| Cuzd1 | -7.229 | 0.00025085 | 0.001430584 |
| Rtn2 | -1.455 | 0.00025317 | 0.001443315 |
| Clec4e | 3.919 | 0.000259467 | 0.001476152 |
| Ccl5 | -1.227 | 0.000260147 | 0.001479005 |
| Trhde | -2.170 | 0.000274882 | 0.001554214 |
| Adra1a | 1.039 | 0.000276778 | 0.001563861 |
| Casq2 | -2.290 | 0.000277597 | 0.001567951 |
| Scn8a | 2.111 | 0.00028026 | 0.001580831 |
| Sypl2 | -4.322 | 0.000285216 | 0.001604402 |
| Il33 | 1.272 | 0.000292449 | 0.00164173 |
| Alpk3 | -5.322 | 0.000300289 | 0.001681171 |
| Slc16a3 | 2.288 | 0.000301566 | 0.001686031 |
| Slc2a5 | 1.138 | 0.000310654 | 0.001729231 |
| Cpeb1 | 1.425 | 0.000310851 | 0.001729742 |
| Clec4d | 2.913 | 0.000316356 | 0.001756235 |
| Cttnbp2 | -2.708 | 0.000319836 | 0.001773763 |
| Nfe2 | 1.955 | 0.000340058 | 0.001873952 |
| 2700097O09Rik | 1.278 | 0.000351626 | 0.001931897 |
| Tmem35a | -2.885 | 0.000357117 | 0.001957503 |
| Ptk6 | 2.112 | 0.000364311 | 0.001992021 |
| Lox | 1.608 | 0.000415781 | 0.002243313 |
| Emilin2 | 1.359 | 0.000420975 | 0.002267633 |
| Atp8b4 | 1.322 | 0.000421142 | 0.002267793 |
| Esco2 | -1.750 | 0.000425819 | 0.002289243 |
| Dlk1 | -4.824 | 0.000428245 | 0.002301537 |
| Myo18b | -2.248 | 0.000428849 | 0.002303283 |
| Amigo3 | 1.664 | 0.000432096 | 0.002317707 |
| Sprr1a | 8.570 | 0.000435751 | 0.002334281 |
| Ccdc120 | 1.548 | 0.000451962 | 0.002415631 |
| Cpsf4l | 1.433 | 0.000464837 | 0.002479629 |
| Vmn2r20 | -4.644 | 0.000485273 | 0.002575606 |
| Cdc6 | 3.020 | 0.000488978 | 0.002594161 |
| Pkhd1l1 | 1.678 | 0.000496787 | 0.002631363 |
| 1810010H24Rik | 1.225 | 0.000497056 | 0.002631748 |
| Sclt1 | 1.426 | 0.00049861 | 0.002637631 |
| Cilp | -1.082 | 0.00050501 | 0.002668921 |
| Marveld3 | 1.406 | 0.000527653 | 0.002777919 |
| Cphx1 | 1.483 | 0.000530178 | 0.002788543 |
| Tnnt1 | -1.238 | 0.000534635 | 0.002810197 |
| Mmp28 | 1.677 | 0.000546996 | 0.002871512 |
| Spata24 | 1.800 | 0.000590941 | 0.003088459 |
| Adm2 | 7.731 | 0.000607843 | 0.003169767 |
| Acot6 | 1.577 | 0.000618841 | 0.003220594 |
| Eml6 | 1.438 | 0.000641807 | 0.003326919 |
| Arl6 | 1.084 | 0.000671427 | 0.003465225 |
| Hhipl1 | 1.959 | 0.000682657 | 0.003515494 |
| Ccr2 | 1.305 | 0.000683136 | 0.00351686 |
| Rnf225 | 2.222 | 0.000688814 | 0.003542778 |
| Mtm1 | 1.002 | 0.000694032 | 0.003564035 |
| Lepr | 1.376 | 0.000726699 | 0.00371679 |
| Kbtbd12 | 1.585 | 0.000751684 | 0.003826799 |
| Dmkn | 3.000 | 0.00075691 | 0.003851028 |
| Orc1 | 4.555 | 0.000774976 | 0.003936879 |
| S100a6 | 1.097 | 0.000804863 | 0.004071165 |
| Foxred2 | 2.115 | 0.000805358 | 0.00407242 |
| Sema3b | 1.707 | 0.000817037 | 0.004122639 |
| Jsrp1 | -7.472 | 0.000842127 | 0.004233714 |
| Ccne1 | 1.053 | 0.000853998 | 0.004282963 |
| Abcc12 | 2.012 | 0.000859065 | 0.004304451 |
| Kcnk3 | -1.745 | 0.000877377 | 0.004384231 |
| Lrp11 | 1.023 | 0.000897736 | 0.004467034 |
| 4930579G24Rik | -1.037 | 0.000937635 | 0.004643177 |
| Mylk2 | -5.000 | 0.001019826 | 0.005005151 |
| Zfp414 | 1.199 | 0.001032568 | 0.00506167 |
| Fbxo40 | -2.104 | 0.001033004 | 0.005062302 |
| Itgae | -1.752 | 0.001104454 | 0.005372577 |
| Fut1 | 3.763 | 0.001120681 | 0.005443496 |
| Accs | 1.466 | 0.001138294 | 0.005517687 |
| Bst1 | 1.668 | 0.001139506 | 0.00552194 |
| Mark1 | -1.219 | 0.001147998 | 0.005553308 |
| Fanca | 1.650 | 0.00116556 | 0.00563306 |
| Dcdc2c | 2.164 | 0.001182832 | 0.005703433 |
| Btc | 1.474 | 0.001185105 | 0.005712725 |
| Mylk4 | -5.229 | 0.001278731 | 0.006121149 |
| Stau2 | 1.257 | 0.001282045 | 0.006135233 |
| Tert | 1.017 | 0.00133517 | 0.006361804 |
| Qtrt1 | 1.219 | 0.001338464 | 0.006372522 |
| Ms4a6d | 1.025 | 0.001353226 | 0.006436695 |
| Snhg11 | 1.392 | 0.001356379 | 0.006447973 |
| Camp | 8.299 | 0.001374594 | 0.006513936 |
| Trem2 | 1.272 | 0.001448723 | 0.006820194 |
| Lingo4 | -1.869 | 0.001461988 | 0.006874802 |
| Aldh1a2 | 1.428 | 0.001477256 | 0.006938692 |
| Trim54 | -6.994 | 0.0015375 | 0.007188935 |
| Crybb3 | 2.537 | 0.001601099 | 0.007458847 |
| Fam241b | 2.036 | 0.001642932 | 0.007621468 |
| 9530077C05Rik | 2.952 | 0.001679587 | 0.007774033 |
| Cdpf1 | 1.009 | 0.001721155 | 0.007943077 |
| Myoc | -4.672 | 0.001721882 | 0.007943077 |
| Nr4a3 | -2.485 | 0.001803697 | 0.008283459 |
| Rbl1 | 1.230 | 0.001838152 | 0.008432314 |
| Tnnc1 | -2.245 | 0.00186053 | 0.008525491 |
| Pdpn | 1.440 | 0.001875253 | 0.008585808 |
| Taf1b | 1.022 | 0.00194599 | 0.008874052 |
| Trdn | -6.229 | 0.001998277 | 0.009096099 |
| Prr5 | 1.032 | 0.00206768 | 0.009373255 |
| Ly6c1 | -1.235 | 0.002074907 | 0.009400856 |
| Mterf2 | 2.052 | 0.002097528 | 0.00949813 |
| Zc3h12d | 1.121 | 0.002146204 | 0.009678731 |
| Gpx6 | -1.638 | 0.002150259 | 0.009694368 |
| Slc35f2 | 4.066 | 0.002155702 | 0.009714411 |
| St8sia3 | 2.091 | 0.002168759 | 0.009769769 |
| Abcb1a | 1.154 | 0.002175828 | 0.00979627 |
| Hypk | 1.555 | 0.00219268 | 0.009864072 |
| Ctsj | 7.409 | 0.00230843 | 0.010311739 |
| Ppp1r42 | 2.415 | 0.002450569 | 0.010878972 |
| Pkp1 | 3.379 | 0.002495457 | 0.011057431 |
| Pf4 | 1.782 | 0.002524226 | 0.011178908 |
| Emb | 1.380 | 0.002548672 | 0.011284142 |
| Hoxb9 | -6.022 | 0.002571766 | 0.011380289 |
| Bmper | 1.548 | 0.002674721 | 0.011801083 |
| Vgll2 | -6.570 | 0.002766905 | 0.012155828 |
| Mtmr11 | 1.120 | 0.00279614 | 0.012267943 |
| Efhd1 | -1.322 | 0.002849329 | 0.012474787 |
| Lnx1 | 2.256 | 0.002851767 | 0.012482149 |
| 4931428F04Rik | 1.278 | 0.002889646 | 0.012624518 |
| 1110002E22Rik | -3.585 | 0.002903133 | 0.012676733 |
| Pcbp3 | 1.247 | 0.002955781 | 0.012886177 |
| Pwwp2a | 1.023 | 0.002967542 | 0.012930622 |
| Timp4 | -5.524 | 0.003116922 | 0.013520857 |
| Mab21l3 | 1.355 | 0.00312672 | 0.013548468 |
| Fermt1 | 1.503 | 0.00312739 | 0.013548468 |
| Myom3 | -1.263 | 0.00314816 | 0.013627719 |
| Dimt1 | 1.114 | 0.003244481 | 0.013986 |
| Asprv1 | 3.446 | 0.003250312 | 0.014007477 |
| Sln | -3.954 | 0.003310108 | 0.014239155 |
| Ptrh1 | 1.473 | 0.003323667 | 0.01428913 |
| Igsf23 | 1.306 | 0.003403782 | 0.014592688 |
| Slc22a29 | 1.909 | 0.003441099 | 0.014733545 |
| Cyp11b2 | -6.644 | 0.00344308 | 0.014738205 |
| Nat8f7 | -1.971 | 0.003454266 | 0.014779632 |
| Clip4 | -1.355 | 0.003470259 | 0.014827632 |
| Myzap | -1.042 | 0.003510704 | 0.01498106 |
| Apobec2 | -3.441 | 0.003516588 | 0.014994543 |
| Cd69 | -2.218 | 0.003589916 | 0.015263847 |
| Acod1 | 2.041 | 0.00362175 | 0.01538731 |
| Tppp3 | -1.218 | 0.003644412 | 0.015459725 |
| Sgcg | -5.644 | 0.003720331 | 0.015745363 |
| Fabp7 | 1.119 | 0.003743224 | 0.015834137 |
| Krt80 | 1.752 | 0.003788092 | 0.016003427 |
| Scn4a | -2.322 | 0.003855184 | 0.016261904 |
| Txlnb | -2.755 | 0.003861098 | 0.01627853 |
| Piwil2 | 4.459 | 0.00393817 | 0.016559931 |
| Trim72 | -2.637 | 0.003958942 | 0.016627355 |
| Gpr35 | 1.585 | 0.004008822 | 0.016806922 |
| Prss35 | -2.848 | 0.004027931 | 0.016878463 |
| 2010106E10Rik | -6.845 | 0.004062093 | 0.016991426 |
| Pls1 | 1.608 | 0.004129068 | 0.017241001 |
| Itgb1bp2 | -4.524 | 0.004130234 | 0.017241509 |
| Gm12184 | 2.927 | 0.00418285 | 0.017430303 |
| Cep78 | 1.143 | 0.00429349 | 0.01785086 |
| 3110001I22Rik | 1.199 | 0.004293505 | 0.01785086 |
| Spindoc | -1.027 | 0.004316385 | 0.017936955 |
| Ogn | -1.027 | 0.004363581 | 0.018105737 |
| Ampd1 | -6.679 | 0.004492008 | 0.018573265 |
| Elfn1 | 5.714 | 0.004558834 | 0.018826 |
| Prss1 | -7.551 | 0.004573839 | 0.018878521 |
| Mphosph6 | 1.057 | 0.004655723 | 0.01917814 |
| Gm21188 | 4.700 | 0.004677354 | 0.019248033 |
| Lrrc75a | 1.574 | 0.004708534 | 0.019361866 |
| Ncr1 | -4.059 | 0.0047939 | 0.019675481 |
| Ccl2 | 1.933 | 0.004812071 | 0.019743365 |
| Ank1 | -3.227 | 0.005026579 | 0.020546873 |
| Nfasc | 1.395 | 0.00503341 | 0.020569701 |
| Melk | 2.769 | 0.005045749 | 0.020615026 |
| Ift22 | 1.473 | 0.005060671 | 0.02066065 |
| Apol6 | -6.077 | 0.005091243 | 0.020775193 |
| Grhl3 | -2.138 | 0.005142477 | 0.020979068 |
| Kdelr3 | 1.553 | 0.005307409 | 0.021603876 |
| Trim68 | 1.290 | 0.00547799 | 0.022221526 |
| Nat8f6 | -1.388 | 0.005547666 | 0.022465049 |
| Slc4a9 | 3.615 | 0.005548908 | 0.022465049 |
| Mfap3l | -1.396 | 0.005589715 | 0.022591481 |
| Hcrtr2 | -2.585 | 0.005598398 | 0.0226155 |
| Rab27a | 1.054 | 0.005603542 | 0.022630743 |
| Pi16 | -1.174 | 0.005652649 | 0.022801181 |
| 3110082I17Rik | 1.174 | 0.005712946 | 0.023010671 |
| Nle1 | 1.037 | 0.005790757 | 0.02326988 |
| Acsbg1 | -2.042 | 0.005836283 | 0.023427434 |
| Myom2 | -3.700 | 0.005876343 | 0.023542447 |
| Nmnat3 | 1.031 | 0.006008109 | 0.023959874 |
| Sprr2a2 | 2.119 | 0.006057471 | 0.024133407 |
| Kazald1 | -1.261 | 0.006106864 | 0.024300874 |
| Itgb3bp | 1.644 | 0.006140811 | 0.024401923 |
| Retnla | -7.937 | 0.006177969 | 0.024536586 |
| Dnah1 | 3.322 | 0.00629914 | 0.024975698 |
| Syne4 | 1.034 | 0.006368107 | 0.025224916 |
| Gm14548 | 2.129 | 0.006384513 | 0.025271504 |
| Atp1a3 | 1.585 | 0.006433927 | 0.025430722 |
| Ift43 | 1.045 | 0.006747371 | 0.02656154 |
| Mgl2 | -1.780 | 0.006790572 | 0.026712495 |
| Srp54b | 1.808 | 0.006799867 | 0.026736679 |
| Mmp11 | 1.632 | 0.006892958 | 0.02704153 |
| Kcna5 | -5.570 | 0.006900726 | 0.027055566 |
| Lrrn4 | 1.354 | 0.006907256 | 0.027074738 |
| Clcn1 | -5.409 | 0.00693632 | 0.027169311 |
| Nrg1 | 1.751 | 0.006966002 | 0.027253244 |
| Crygn | -1.241 | 0.006989052 | 0.027336947 |
| Miox | 7.551 | 0.007141113 | 0.027865702 |
| Wscd2 | -4.000 | 0.00722027 | 0.028128047 |
| Myo5a | 1.817 | 0.00737918 | 0.028638991 |
| Hao2 | -3.100 | 0.007408885 | 0.028747519 |
| Cyp2c39 | 1.067 | 0.007465342 | 0.028905439 |
| Nnt | 10.842 | 0.007568304 | 0.02924726 |
| AI413582 | 1.250 | 0.007569567 | 0.02924726 |
| Cerkl | 1.918 | 0.007748613 | 0.029848252 |
| Capg | 1.027 | 0.007832684 | 0.030101865 |
| Ecrg4 | -7.180 | 0.007898845 | 0.03032084 |
| Uprt | 1.312 | 0.007975298 | 0.030578767 |
| Gcnt1 | 1.544 | 0.008086237 | 0.03093946 |
| Nlrp1b | 4.644 | 0.008174469 | 0.031211951 |
| Kif9 | 2.026 | 0.00827804 | 0.031563612 |
| Bhlhe41 | 1.816 | 0.008453217 | 0.032164696 |
| Lrrc30 | -6.409 | 0.008568802 | 0.032559474 |
| Gm52968 | 6.409 | 0.008581879 | 0.032586665 |
| Evi2a | 1.053 | 0.008623524 | 0.032707187 |
| Cd177 | 2.755 | 0.008676287 | 0.03286955 |
| Ank2 | 1.381 | 0.008689813 | 0.032913239 |
| Trank1 | -3.907 | 0.008921403 | 0.033666827 |
| Ms4a4a | 4.115 | 0.009144405 | 0.034366975 |
| Mre11a | 1.004 | 0.009178412 | 0.034455566 |
| Msmp | 2.090 | 0.009185031 | 0.034472578 |
| Brd3os | 2.241 | 0.009245204 | 0.034674766 |
| Jph2 | -1.519 | 0.009290248 | 0.034835793 |
| Cox7a1 | -1.341 | 0.009391816 | 0.035176696 |
| Tbc1d2 | 1.183 | 0.009396701 | 0.03518701 |
| Ptgir | 2.193 | 0.00949714 | 0.035482637 |
| Trim5 | -1.105 | 0.00969171 | 0.036095223 |
| Ovol3 | 8.129 | 0.009891071 | 0.036796212 |
| Gdf3 | 2.744 | 0.010239689 | 0.037930731 |
| Cabcoco1 | 1.335 | 0.01051516 | 0.038820503 |
| Wdr93 | 1.795 | 0.010729534 | 0.039526522 |
| Ppil6 | -3.290 | 0.010835315 | 0.039833235 |
| Flrt2 | 1.307 | 0.010857995 | 0.039897031 |
| Gpx7 | 1.214 | 0.010916569 | 0.040078398 |
| Dnase1 | -3.585 | 0.011002491 | 0.040340022 |
| Cfap300 | 2.858 | 0.0111784 | 0.040876056 |
| B3gnt9 | 1.318 | 0.011324693 | 0.041310359 |
| Zfp968-ps | 1.193 | 0.011609792 | 0.042238357 |
| Plaur | 1.211 | 0.011660758 | 0.042386417 |
| Unc79 | 4.000 | 0.011692422 | 0.042464118 |
| Rad51ap1 | 6.966 | 0.011877474 | 0.043060409 |
| A1bg | 3.078 | 0.012391183 | 0.04466778 |
| Cntnap1 | 1.290 | 0.012404212 | 0.044685477 |
| Cd8b1 | -2.366 | 0.012585241 | 0.045258621 |
| Itgbl1 | -1.385 | 0.012746651 | 0.04573945 |
| Trim63 | -6.022 | 0.012773882 | 0.045814989 |
| Ptafr | 1.014 | 0.012956872 | 0.046403027 |
| Gnb1l | 1.324 | 0.013342562 | 0.047516328 |
| Cxcl2 | 3.445 | 0.013371261 | 0.047597997 |
| Fbxl22 | -1.204 | 0.013484832 | 0.047918352 |
| Shisa2 | -1.311 | 0.013572588 | 0.048127884 |
| Cidea | -2.420 | 0.013610002 | 0.048239832 |
| Hmcn2 | -1.193 | 0.014056069 | 0.049533766 |
| Gzmk | -3.678 | 0.014085874 | 0.049628209 |
| Rpgr | 1.202 | 0.014278756 | 0.050211343 |
| Lilr4b | 1.185 | 0.014361851 | 0.050471299 |
| Eda2r | 1.848 | 0.01438091 | 0.050484546 |
| Chaf1b | 1.063 | 0.014651297 | 0.051324615 |
| Klhl38 | -3.755 | 0.014766577 | 0.051706509 |
| Cdhr2 | 1.566 | 0.014837256 | 0.051898961 |
| Car15 | -2.036 | 0.014976234 | 0.052351814 |
| Hebp2 | 2.399 | 0.015076726 | 0.052633779 |
| Ppp1r1a | -6.607 | 0.015093801 | 0.052662439 |
| Aldh1a3 | 1.390 | 0.015449984 | 0.053712201 |
| Cxcl14 | 1.170 | 0.015916345 | 0.055136144 |
| Gm17359 | 6.679 | 0.015944054 | 0.055220543 |
| Zfp13 | 1.473 | 0.016045944 | 0.05550357 |
| Irx1 | -1.046 | 0.01626475 | 0.056084174 |
| Sirpb1b | 1.343 | 0.016285907 | 0.0561454 |
| Ankub1 | 2.700 | 0.016503459 | 0.056800523 |
| Meig1 | -2.363 | 0.017184894 | 0.058753904 |
| Rpp38 | 1.013 | 0.017316388 | 0.059152089 |
| Rhox5 | 3.907 | 0.017319273 | 0.059152089 |
| Sspo | 1.415 | 0.017323979 | 0.059155923 |
| Sh3rf2 | -1.939 | 0.017683905 | 0.060235458 |
| Lrrcc1 | 1.485 | 0.018176522 | 0.06164634 |
| Fam229b | 2.719 | 0.018265795 | 0.061910957 |
| Il1f9 | 2.632 | 0.018305784 | 0.062008308 |
| Igf2bp2 | 1.008 | 0.0184156 | 0.062341924 |
| Cacna1h | -2.503 | 0.018476012 | 0.062533613 |
| Serpinb2 | 3.585 | 0.018540682 | 0.062688236 |
| Eif5a2 | -1.363 | 0.018671678 | 0.063079476 |
| Anxa8 | 1.734 | 0.018786345 | 0.063376084 |
| Cacng6 | -5.781 | 0.019291816 | 0.064803232 |
| Csgalnact1 | -1.043 | 0.019348123 | 0.064939522 |
| Nrm | 1.412 | 0.019461213 | 0.065252768 |
| Cux2 | 1.601 | 0.019516571 | 0.065375192 |
| Zfp185 | 1.896 | 0.019660668 | 0.065774595 |
| Ier5l | -1.304 | 0.019917129 | 0.066538204 |
| Recql4 | 1.154 | 0.020241924 | 0.067432236 |
| Dhrs7c | -6.679 | 0.020333841 | 0.067697464 |
| H3c2 | -7.512 | 0.020333841 | 0.067697464 |
| Dbil5 | -2.924 | 0.020341566 | 0.067709526 |
| Mcemp1 | 2.291 | 0.020701602 | 0.068755471 |
| Tub | -3.907 | 0.020760852 | 0.068896818 |
| Zbtb45 | 1.427 | 0.020809461 | 0.069005342 |
| Tbx15 | -5.492 | 0.020831694 | 0.069062503 |
| Fbxw15 | 2.290 | 0.020893059 | 0.069238137 |
| Scube1 | -1.441 | 0.020908602 | 0.069265461 |
| Catsperd | 1.295 | 0.020956374 | 0.069378332 |
| Cd209f | -1.435 | 0.021053225 | 0.069657064 |
| Ncam2 | 1.282 | 0.021714177 | 0.071494671 |
| Ccl8 | 8.155 | 0.021772295 | 0.071662798 |
| Rsph9 | -1.032 | 0.021892256 | 0.072014575 |
| Foxj1 | -1.826 | 0.021909939 | 0.072058385 |
| Gm9733 | 7.861 | 0.022088753 | 0.072516468 |
| Nme5 | 7.662 | 0.022501269 | 0.073607282 |
| Nptx1 | 3.322 | 0.022645186 | 0.073931586 |
| Gm10309 | 3.544 | 0.022780503 | 0.074343961 |
| Slfn4 | 2.000 | 0.023850682 | 0.077135115 |
| Grid1 | -1.206 | 0.023956381 | 0.077401143 |
| Fignl1 | 2.182 | 0.024188485 | 0.078059401 |
| Il1r2 | 2.385 | 0.024353464 | 0.078510367 |
| Gm14440 | -2.585 | 0.024354929 | 0.078510367 |
| Tff2 | -1.254 | 0.024360814 | 0.078510367 |
| Nxph4 | 6.409 | 0.024369328 | 0.078520219 |
| Greb1 | -2.322 | 0.024422948 | 0.078631604 |
| Tmem107 | 1.206 | 0.024947721 | 0.080024651 |
| Gsg1l | -1.784 | 0.02499968 | 0.080160171 |
| Kcnt2 | 1.469 | 0.025150303 | 0.080518034 |
| Car4 | -4.044 | 0.025440933 | 0.081290848 |
| Nme4 | 1.281 | 0.025906295 | 0.082554128 |
| Ptges | 2.000 | 0.026082765 | 0.0830844 |
| Tpx2 | 1.328 | 0.026185558 | 0.083379664 |
| Apba2 | -2.059 | 0.026394193 | 0.083963031 |
| Tmem171 | -1.500 | 0.026746647 | 0.084790157 |
| Mtbp | 1.179 | 0.027136182 | 0.085860169 |
| Ly6f | 3.145 | 0.027754587 | 0.087465713 |
| Pclaf | 1.714 | 0.027927982 | 0.087893822 |
| Ccdc57 | 1.252 | 0.028301244 | 0.088899075 |
| Igkv2-137 | 3.061 | 0.028458412 | 0.08931888 |
| Clec9a | -1.138 | 0.028524969 | 0.089499666 |
| Chtf18 | 1.745 | 0.028642555 | 0.089851527 |
| Nlrp1a | 2.363 | 0.028916641 | 0.090573655 |
| Mmp27 | 1.290 | 0.029052528 | 0.090947523 |
| Tarm1 | 4.143 | 0.029116316 | 0.091095392 |
| Cd300lf | 1.309 | 0.029371544 | 0.091720103 |
| Rad51 | 1.798 | 0.029527089 | 0.092083914 |
| Gm28035 | 1.138 | 0.029747662 | 0.092666774 |
| Gpx2 | 1.443 | 0.029802994 | 0.092769123 |
| Vcpkmt | 1.051 | 0.030518413 | 0.09463918 |
| B3gnt4 | -6.229 | 0.030817466 | 0.095429256 |
| Cfap126 | 1.193 | 0.031050029 | 0.095945346 |
| Tmem130 | -3.000 | 0.031214281 | 0.096380733 |
| Il7 | 2.737 | 0.031520877 | 0.097218321 |
| Tmod4 | -2.713 | 0.03164407 | 0.09756183 |
| Tubb1 | 3.215 | 0.031714984 | 0.097762209 |
| Atg9b | 2.392 | 0.031820466 | 0.098032449 |
| Yy2 | 1.615 | 0.032155959 | 0.098826299 |
| Pif1 | 3.858 | 0.032183354 | 0.098876056 |
| Trpc3 | -1.415 | 0.03226108 | 0.099094034 |
| Zfp879 | -1.073 | 0.032306345 | 0.099214612 |
| Six1 | -4.781 | 0.032393256 | 0.099389083 |
| Pimreg | 3.841 | 0.032741521 | 0.100308501 |
| Moxd1 | 1.602 | 0.032771809 | 0.100382667 |
| Olfr648 | 1.761 | 0.033172765 | 0.101347584 |
| Apc2 | -1.874 | 0.034033202 | 0.103497543 |
| B3galt2 | -3.644 | 0.03421001 | 0.103920381 |
| Soat1 | 1.229 | 0.034317896 | 0.104171441 |
| Fam83d | -1.126 | 0.034637705 | 0.104943789 |
| Lmod2 | -2.459 | 0.034916996 | 0.10564039 |
| Bub1 | 3.585 | 0.03495592 | 0.105719408 |
| 2310002L09Rik | -6.022 | 0.035292391 | 0.106597401 |
| Tmem28 | 1.263 | 0.035324028 | 0.106637356 |
| Exo1 | 2.000 | 0.035539083 | 0.107168996 |
| ccdc198 | 1.298 | 0.035651568 | 0.107450005 |
| Veph1 | -1.280 | 0.035719551 | 0.107614924 |
| Map10 | -1.022 | 0.035769476 | 0.107706375 |
| Serpini2 | -6.229 | 0.035918701 | 0.10807611 |
| Usp13 | -3.000 | 0.035924996 | 0.10807611 |
| Tnfsf14 | 1.814 | 0.03621743 | 0.108737911 |
| C130050O18Rik | 1.447 | 0.036589756 | 0.109596675 |
| Asb4 | -1.043 | 0.037193562 | 0.111203497 |
| Pcdhga5 | -1.181 | 0.037300183 | 0.111441553 |
| Chil4 | -1.612 | 0.037428832 | 0.111748385 |
| Gpr55 | -1.290 | 0.037526555 | 0.111976039 |
| Slc6a2 | -2.907 | 0.037882761 | 0.112794298 |
| Cym | 7.050 | 0.038047331 | 0.113202638 |
| Wdr25 | 1.260 | 0.038112919 | 0.113359948 |
| Actg2 | 1.184 | 0.038113935 | 0.113359948 |
| Cavin4 | -5.781 | 0.038120964 | 0.113360432 |
| Siglecf | 1.729 | 0.038226402 | 0.113633038 |
| Fam83a | 2.149 | 0.038419909 | 0.114146607 |
| Scube3 | -1.585 | 0.038937432 | 0.115331362 |
| Rassf10 | 3.170 | 0.040324945 | 0.118759336 |
| Ssc5d | -1.181 | 0.040357906 | 0.118835212 |
| Ano5 | -2.459 | 0.040411016 | 0.118970376 |
| Pla2g2d | -2.907 | 0.041203826 | 0.120887648 |
| Ntrk1 | -1.043 | 0.041208703 | 0.120887648 |
| Tafa2 | 1.737 | 0.041683226 | 0.122039806 |
| Wdr86 | 1.902 | 0.041720727 | 0.122064363 |
| Stac | -3.087 | 0.042367487 | 0.123518844 |
| Ccdc163 | 1.200 | 0.042545947 | 0.123869092 |
| Mcf2l | 1.466 | 0.042624685 | 0.124027768 |
| Gm15922 | 1.798 | 0.04288799 | 0.124706009 |
| Afp | 1.503 | 0.042905099 | 0.124733789 |
| Ccl17 | -3.278 | 0.04321084 | 0.125556312 |
| H4c18 | 8.430 | 0.043435184 | 0.126030736 |
| Lag3 | -1.373 | 0.043690732 | 0.126594242 |
| Tox2 | -1.459 | 0.043806341 | 0.126840176 |
| Il23a | 3.426 | 0.044028002 | 0.127370302 |
| Kcnc4 | -1.485 | 0.044581395 | 0.128723124 |
| Plod2 | 1.077 | 0.044604306 | 0.128744247 |
| Fam163a | -3.585 | 0.044718137 | 0.128982608 |
| Lysmd2 | 1.263 | 0.044872878 | 0.129361135 |
| Kcnj5 | -1.441 | 0.045126581 | 0.129903021 |
| Fmo3 | 2.070 | 0.045355164 | 0.13040992 |
| H2al1m | -2.982 | 0.045423876 | 0.130567233 |
| Mettl7a2 | 3.059 | 0.045508201 | 0.130713362 |
| Tspoap1 | 3.170 | 0.046100401 | 0.132091976 |
| Egr2 | 3.858 | 0.046189046 | 0.132284197 |
| Slc4a5 | 3.000 | 0.046378624 | 0.132659577 |
| Acan | -4.129 | 0.046849509 | 0.133728457 |
| Aqp12 | -6.814 | 0.046919451 | 0.133881807 |
| Diaph3 | 1.617 | 0.046938523 | 0.133913082 |
| Dlec1 | 2.100 | 0.047148324 | 0.134488394 |
| Slc36a2 | -1.726 | 0.048017809 | 0.136567447 |
| Nxpe5 | 2.585 | 0.04848924 | 0.137694764 |
| Gm27029 | -1.456 | 0.048500065 | 0.13769519 |
| Syn2 | -2.170 | 0.048802186 | 0.138321733 |
| Xlr3b | 1.594 | 0.049369675 | 0.139594679 |
| Nkain2 | 4.907 | 0.049468427 | 0.139826011 |

**Table S11** Sixty differential metabolites identified from different groups.

| No. | Compound | Ion | Model vs Control | | | | | BHP PM-SS vs Model | | | | |
| --- | --- | --- | --- | --- | --- | --- | --- | --- | --- | --- | --- | --- |
|  |  |  | VIP | Log_FC | *p* value | ^*^FDR | Trend | VIP | Log_FC | *p* value | ^*^FDR | Trend |
| 1 | Glycerophosphocholine | M+H | 12.03 | -1.00 | 6.20E-06 | 5.90E-05 | Down | 27.26 | 2.46 | 7.53E-05 | 1.57E-03 | UP |
| 2 | Betaine | M+H | 10.93 | -0.39 | 5.81E-06 | 5.58E-05 | Down | 17.39 | 0.65 | 2.65E-04 | 4.04E-03 | UP |
| 3 | D-pyroglutamic acid | M+H | 9.89 | -1.22 | 2.82E-14 | 8.73E-12 | Down | 6.78 | 0.81 | 2.14E-03 | 1.95E-02 | UP |
| 4 | Cysteinylglycine | M+H | 6.48 | 1.21 | 1.58E-11 | 1.19E-09 | UP | 5.18 | -0.50 | 1.08E-03 | 1.19E-02 | Down |
| 5 | L-Glutamate | M+H | 6.19 | 1.56 | 2.05E-14 | 6.79E-12 | UP | 4.13 | -0.53 | 3.23E-03 | 2.65E-02 | Down |
| 6 | L-Saccharopine | M+H | 6.15 | -0.29 | 6.61E-05 | 4.51E-04 | Down | 5.53 | 0.29 | 2.51E-02 | 1.13E-01 | UP |
| 7 | L-propionylcarnitine | M+H | 5.49 | -1.34 | 6.81E-15 | 2.80E-12 | Down | 4.89 | 0.58 | 3.14E-07 | 2.59E-05 | Down |
| 8 | Uracil | M+H | 4.68 | -0.48 | 1.53E-05 | 1.29E-04 | Down | 3.21 | 0.56 | 4.97E-02 | 1.73E-01 | UP |
| 9 | 2-aminoadipic acid | M+H | 3.96 | 2.67 | 1.26E-17 | 6.05E-14 | UP | 4.08 | -1.54 | 2.65E-10 | 1.69E-07 | Down |
| 10 | 2-piperidone | M+H | 3.41 | 3.70 | 2.58E-10 | 1.21E-08 | UP | 3.63 | -2.84 | 1.05E-08 | 2.25E-06 | UP |
| 11 | Acetylcarnitine | M+H | 3.34 | -1.34 | 4.74E-12 | 4.54E-10 | Down | 3.21 | 1.00 | 8.04E-09 | 1.88E-06 | UP |
| 12 | G-guanidinobutyrate | M+H | 3.16 | 0.38 | 2.20E-04 | 1.25E-03 | UP | 5.02 | -0.82 | 8.19E-06 | 3.18E-04 | Down |
| 13 | Creatinine | M+H | 2.44 | 0.73 | 3.15E-06 | 3.35E-05 | UP | 1.86 | -0.75 | 8.52E-03 | 5.31E-02 | Down |
| 14 | S-adenosyl-l-homocysteine | M+H | 2.37 | -0.45 | 1.63E-02 | 4.55E-02 | Down | 3.43 | -0.48 | 1.47E-03 | 1.49E-02 | UP |
| 15 | Arginine | M+H | 2.35 | -0.91 | 1.29E-08 | 3.10E-07 | Down | 1.46 | 0.72 | 4.50E-02 | 1.63E-01 | UP |
| 16 | L-Lysine | M+H | 2.33 | -0.47 | 2.81E-05 | 2.19E-04 | Down | 2.10 | 0.55 | 3.91E-02 | 1.49E-01 | UP |
| 17 | L-methionine | M+H | 1.98 | -0.64 | 1.69E-06 | 1.99E-05 | Down | 1.65 | 0.68 | 2.54E-02 | 1.14E-01 | UP |
| 18 | Isopimaric acid | M+H | 1.98 | -1.50 | 3.81E-06 | 3.93E-05 | Down | 2.42 | 1.53 | 3.76E-06 | 1.83E-04 | UP |
| 19 | L-palmitoylcarnitine | M+H | 1.85 | 1.09 | 2.45E-10 | 1.17E-08 | UP | 1.77 | -0.66 | 2.71E-05 | 7.51E-04 | Down |
| 20 | Anserine | M+H | 1.74 | -0.94 | 3.95E-06 | 4.06E-05 | Down | 1.50 | -0.78 | 1.38E-05 | 4.57E-04 | UP |
| 21 | Malonyl-l-carnitine | M+H | 1.73 | 0.34 | 8.51E-06 | 7.76E-05 | UP | 1.57 | -0.20 | 4.89E-02 | 1.71E-01 | Down |
| 22 | N-acetyl-d-galactosamine | M+H | 1.72 | -1.33 | 4.63E-11 | 3.00E-09 | Down | 1.89 | 0.45 | 1.52E-08 | 2.97E-06 | UP |
| 23 | Ectoine | M+H | 1.50 | 0.65 | 1.70E-08 | 3.93E-07 | UP | 1.60 | -0.57 | 2.11E-04 | 3.39E-03 | Down |
| 24 | 15-oxo-5z,8z,11z,13e-eicosatetraenoic acid | M+H | 1.49 | -1.77 | 2.75E-13 | 4.80E-11 | Down | 1.10 | 1.17 | 9.96E-05 | 1.96E-03 | UP |
| 25 | Salmeterol | M+H | 1.45 | 1.80 | 1.40E-13 | 2.99E-11 | UP | 1.20 | -0.67 | 8.42E-06 | 3.24E-04 | Down |
| 26 | Keracyanin | M+H | 1.42 | -0.93 | 5.67E-10 | 2.27E-08 | Down | 1.39 | 0.65 | 2.39E-05 | 6.79E-04 | UP |
| 27 | Glutathione disulfide | M+H | 1.38 | -0.78 | 6.04E-04 | 2.93E-03 | Down | 1.57 | 0.56 | 8.56E-04 | 1.00E-02 | UP |
| 28 | γ-glu-cys | M+H | 1.11 | 0.74 | 1.01E-08 | 2.50E-07 | UP | 1.26 | -0.55 | 6.58E-06 | 2.70E-04 | Down |
| 29 | Pro-phe | M+H | 1.02 | 0.74 | 1.66E-07 | 2.80E-06 | UP | 1.31 | -0.93 | 1.17E-06 | 7.36E-05 | Down |
| 30 | Taurocholate | M-H | 43.14 | 2.32 | 9.54E-13 | 2.33E-10 | UP | 16.90 | -0.84 | 1.26E-02 | 3.42E-02 | Down |
| 31 | Glutathione | M-H | 14.71 | -0.73 | 9.23E-08 | 2.58E-06 | Down | 15.79 | 0.66 | 4.02E-07 | 4.45E-06 | UP |
| 32 | Adenosine 5'-diphosphoribose | M-H | 12.11 | -1.92 | 2.18E-05 | 2.23E-04 | Down | 18.85 | 1.84 | 1.48E-11 | 8.63E-10 | UP |
| 33 | Arachidonic acid | M-H | 7.90 | -0.67 | 2.18E-05 | 2.23E-04 | Down | 10.37 | 0.52 | 3.87E-08 | 6.21E-07 | UP |
| 34 | UDP-alpha-D-galactose | M-H | 7.89 | -2.98 | 2.79E-13 | 1.02E-10 | Down | 5.27 | 2.64 | 7.08E-06 | 5.34E-05 | UP |
| 35 | Udp-N-acetylglucosamine | M-H | 6.67 | 0.65 | 2.94E-06 | 4.17E-05 | UP | 9.61 | -0.71 | 3.76E-11 | 1.86E-09 | Down |
| 36 | Glutamine | M-H | 6.00 | -2.02 | 1.78E-12 | 3.62E-10 | Down | 3.93 | 1.68 | 1.72E-05 | 1.16E-04 | UP |
| 37 | UDP-glucuronate | M-H | 5.17 | -0.55 | 6.98E-05 | 5.75E-04 | Down | 8.06 | 0.31 | 4.86E-12 | 3.50E-10 | UP |
| 38 | N-Acetyl-D-glucosamine 6-phosphate | M-H | 4.95 | -1.20 | 7.44E-11 | 7.58E-09 | Down | 5.48 | 0.56 | 1.05E-10 | 4.37E-09 | UP |
| 39 | Leucine | M-H | 4.70 | 0.67 | 2.79E-02 | 7.61E-02 | UP | 11.34 | -0.53 | 5.57E-06 | 4.32E-05 | Down |
| 40 | Taurocholic acid | M-H | 4.24 | 5.53 | 2.30E-16 | 6.32E-13 | UP | 4.27 | -1.06 | 1.68E-11 | 9.58E-10 | Down |
| 41 | L-tyrosine | M-H | 4.07 | 1.08 | 2.84E-03 | 1.20E-02 | UP | 8.98 | -0.66 | 2.11E-14 | 5.17E-12 | Down |
| 42 | N-acetyl-l-glutamate | M-H | 4.02 | 0.97 | 3.12E-07 | 6.84E-06 | UP | 6.08 | -0.77 | 3.56E-13 | 4.72E-11 | Down |
| 43 | Phenylalanine | M-H | 3.95 | 0.74 | 2.20E-02 | 6.27E-02 | UP | 9.54 | -0.41 | 9.11E-12 | 5.86E-10 | Down |
| 44 | D-Glucosamine 6-phosphate | M-H | 3.59 | 1.34 | 3.24E-09 | 1.63E-07 | UP | 4.10 | -0.79 | 1.04E-09 | 2.87E-08 | Down |
| 45 | N-Glycoloyl-neuraminate | M-H | 3.35 | -2.28 | 1.97E-13 | 7.73E-11 | Down | 4.36 | 0.59 | 3.06E-14 | 7.32E-12 | UP |
| 46 | Uridine 5'-diphosphate | M-H | 2.94 | -1.40 | 2.85E-05 | 2.78E-04 | Down | 3.40 | 2.03 | 5.03E-04 | 2.14E-03 | UP |
| 47 | Uridine 5'-monophosphate | M-H | 2.89 | -1.32 | 1.02E-12 | 2.43E-10 | Down | 3.39 | 0.95 | 4.20E-13 | 5.37E-11 | UP |
| 48 | beta-D-Fructose | M-H | 2.85 | 0.69 | 4.80E-02 | 1.17E-01 | UP | 8.30 | -0.60 | 2.26E-07 | 2.75E-06 | Down |
| 49 | Dihydrothymine | M-H | 2.71 | 1.78 | 2.37E-12 | 4.57E-10 | UP | 1.65 | -0.56 | 1.92E-05 | 1.28E-04 | Down |
| 50 | Pantothenate | M-H | 2.27 | -1.49 | 1.22E-05 | 1.39E-04 | Down | 3.56 | 1.57 | 5.26E-07 | 5.65E-06 | UP |
| 51 | DL-2-Aminoadipic acid | M-H | 2.17 | 2.66 | 6.00E-15 | 6.00E-12 | UP | 2.60 | -1.40 | 7.25E-14 | 1.39E-11 | Down |
| 52 | D-aspartic acid | M-H | 1.79 | -0.58 | 2.11E-04 | 1.43E-03 | Down | 3.18 | 0.63 | 1.87E-12 | 1.66E-10 | UP |
| 53 | L-dihydroorotate | M-H | 1.71 | -0.86 | 5.47E-03 | 2.03E-02 | Down | 2.62 | 0.61 | 6.12E-09 | 1.31E-07 | UP |
| 54 | Valine | M-H | 1.65 | -0.64 | 1.01E-03 | 5.22E-03 | Down | 2.72 | 0.59 | 4.20E-08 | 6.66E-07 | UP |
| 55 | 3-hydroxyglutaric acid | M-H | 1.61 | -0.71 | 1.11E-02 | 3.62E-02 | Down | 3.53 | 0.82 | 1.71E-12 | 1.53E-10 | UP |
| 56 | Cdp-ethanolamine | M-H | 1.54 | 1.08 | 3.11E-08 | 1.08E-06 | UP | 1.90 | -0.81 | 1.11E-09 | 3.03E-08 | Down |
| 57 | D-proline | M-H | 1.52 | -0.52 | 7.50E-03 | 2.64E-02 | Down | 3.34 | 0.64 | 8.26E-13 | 8.90E-11 | UP |
| 58 | Cysteineglutathione disulfide | M-H | 1.25 | -1.32 | 1.46E-04 | 1.05E-03 | Down | 1.49 | 0.96 | 1.11E-03 | 4.25E-03 | UP |
| 59 | Adenylosuccinate | M-H | 1.21 | -2.41 | 8.08E-07 | 1.47E-05 | Down | 2.15 | 2.81 | 1.99E-12 | 1.72E-10 | UP |
| 60 | DL-serine | M-H | 1.18 | -0.74 | 2.21E-03 | 9.83E-03 | Down | 2.04 | 0.67 | 6.17E-12 | 4.32E-10 | UP |

^*^False discovery rate (FDR) from two-sided unpaired t-test <0.05.


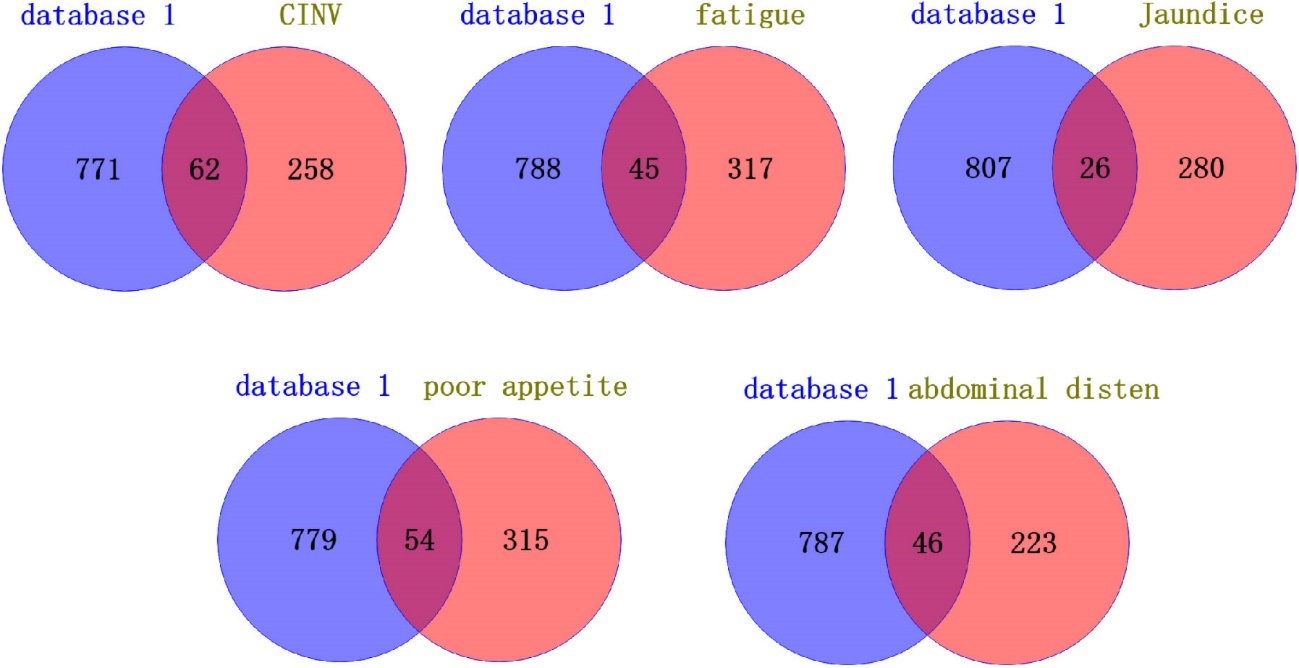


Figure. S1 The number of targets in database 1, database 2, and the overlapped database.


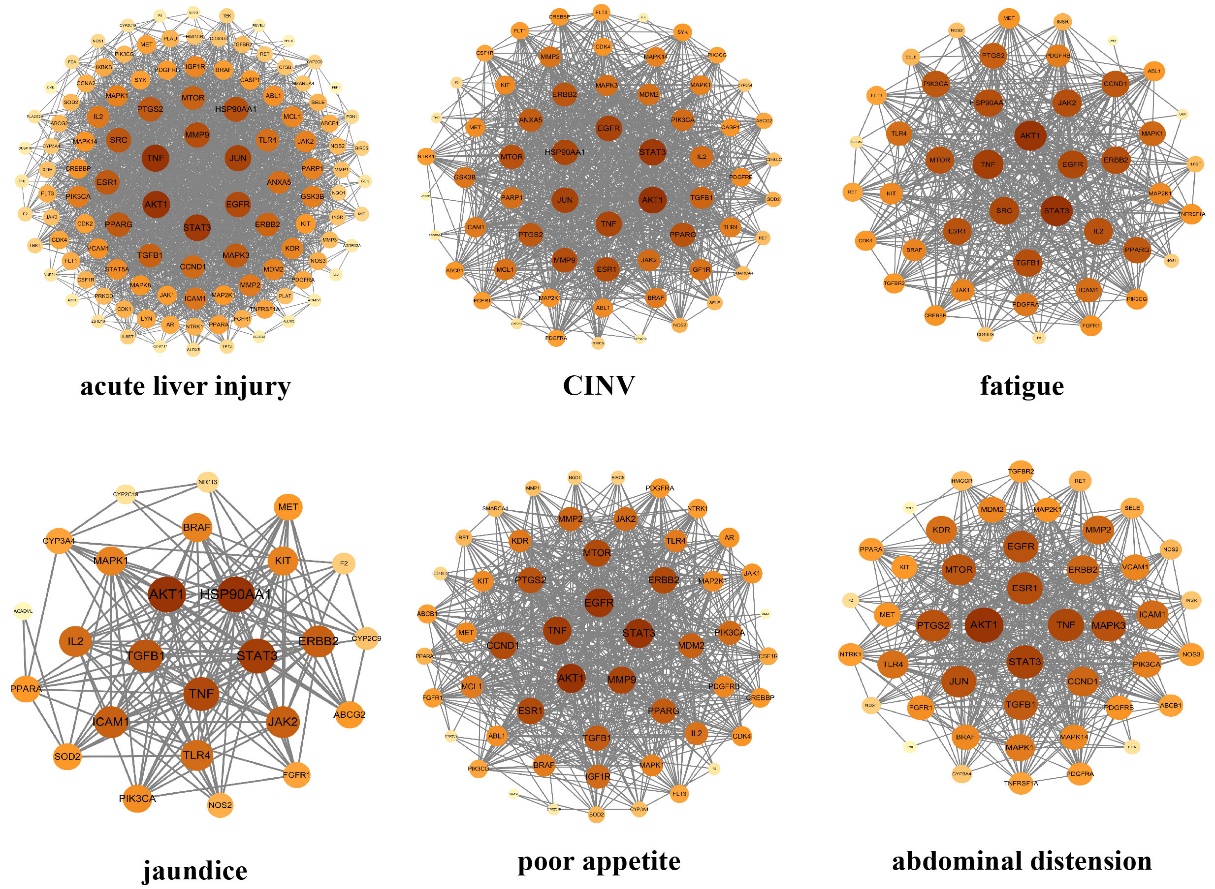


Figure. S2 Protein-protein interaction analysis for targets associated with acute liver injury, CINV, fatigue, jaundice, poor appetite and abdominal distension. The size of the node was related to its degree value, and the greater the degree value was, the larger the node.


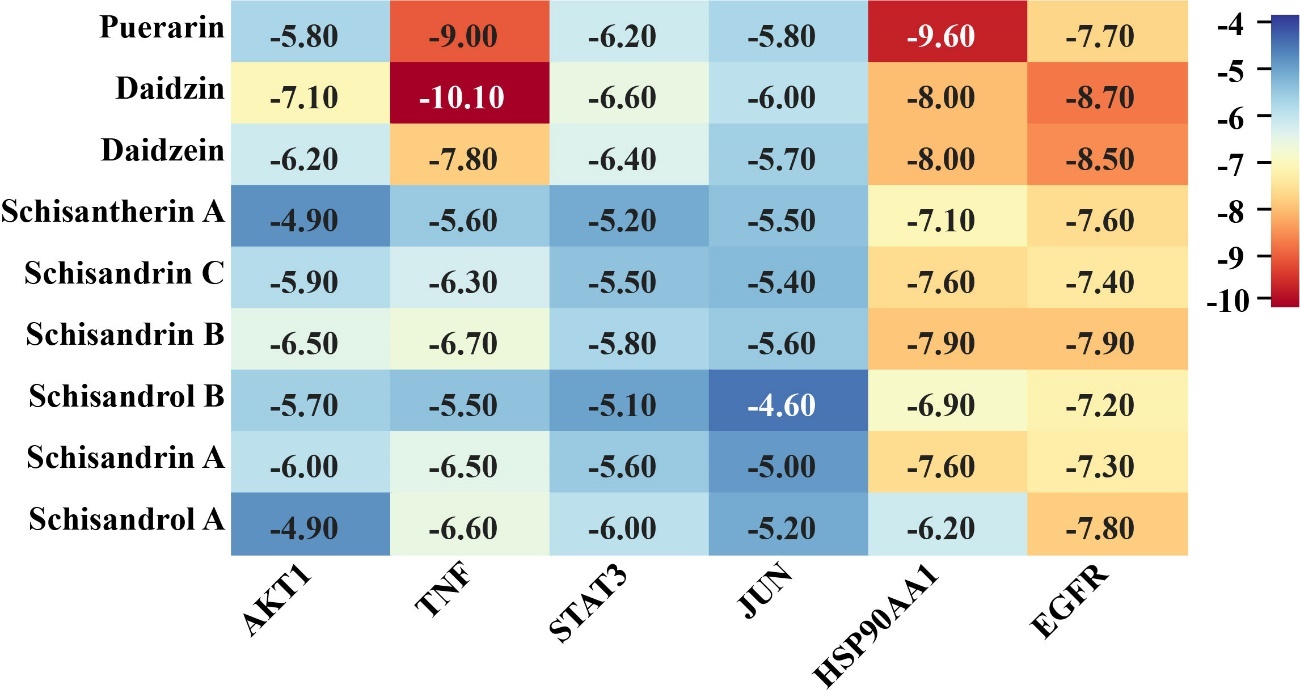


Figure. S3 Molecular Docking scores heat map.


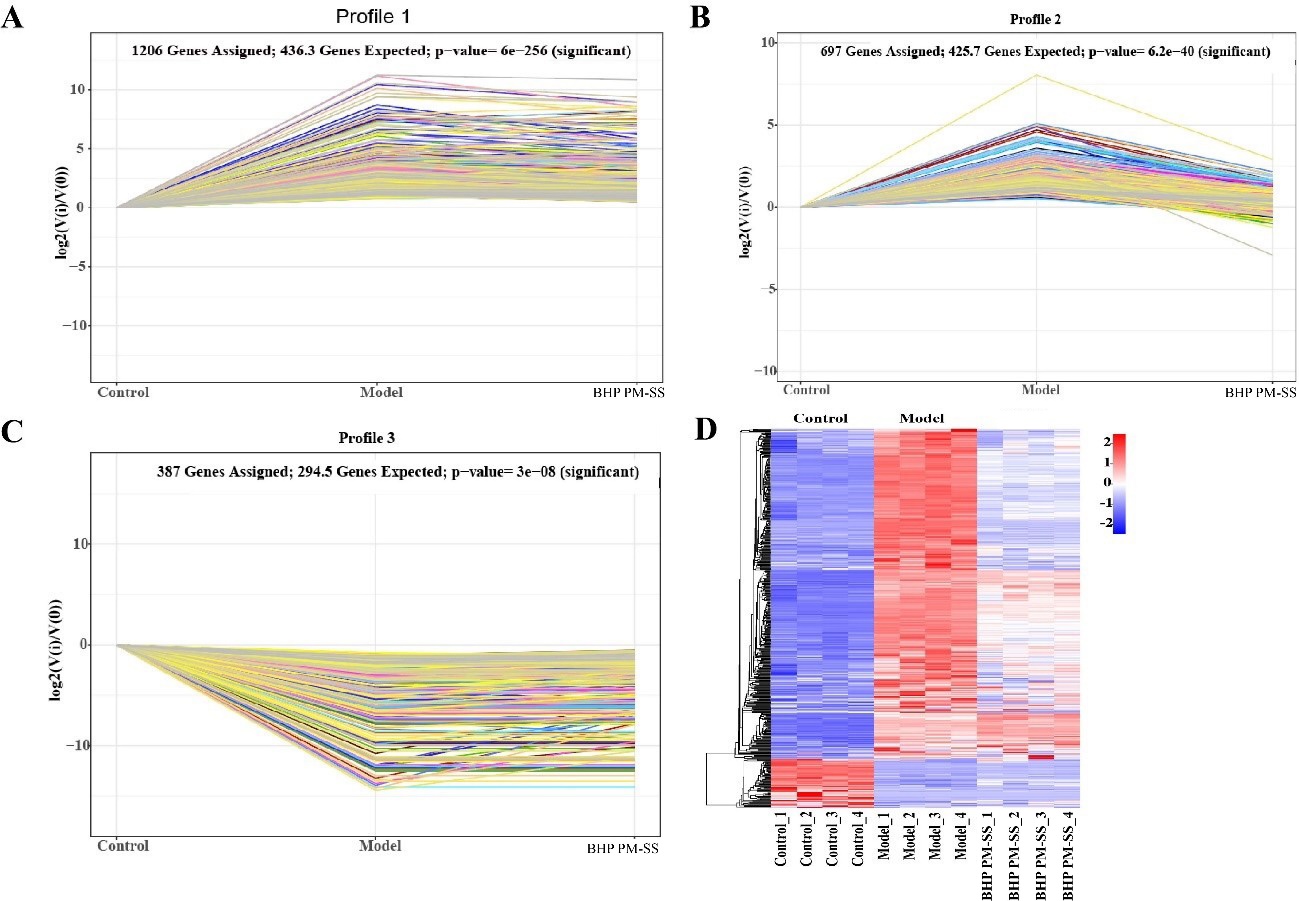


Figure. S4 (A, B and C) STEM clustering of DEGs expression patterns. Each box shows a clustering of gene expression pattern. The curve denotes gene expression tendency in different sample subgroups. The p value and number of clustered time series genes is marked in the top corner of each cluster box; (D) Heatmap of DEGs between the three groups.


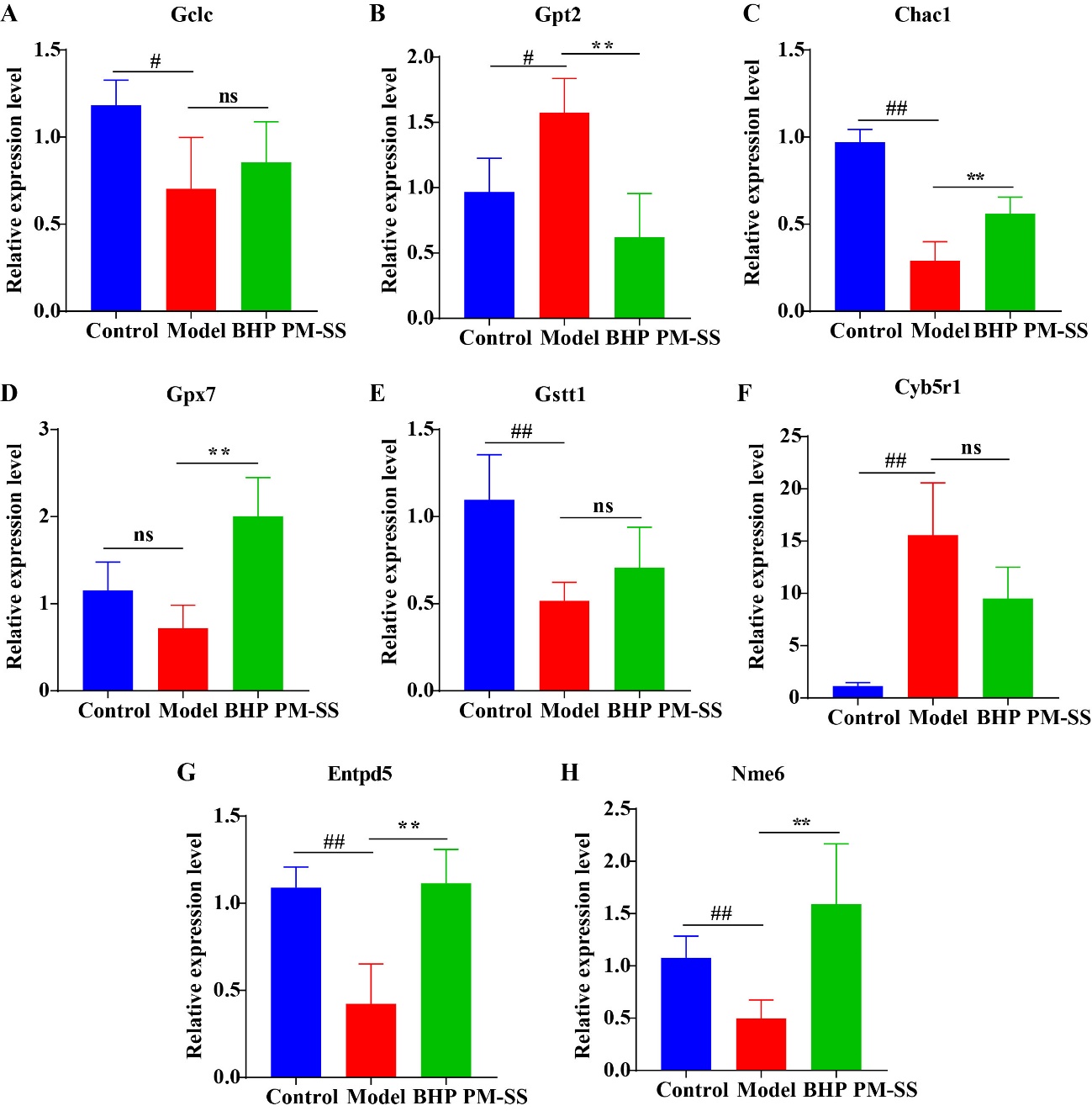


Figure. S5 RT-PCR analysis of the expression of 8 candidate unigenes related to Glutathione metabolism, Pyrimidine metabolism, Arginine biosynthesis and Amino sugar and nucleotide sugar metabolism in liver tissues of control, model, and BHP PM-SS mice. Four biological replicates and three technical replicates were performed. ^#^*p* < 0.05 or ^##^*p* < 0.01, compared to the control group; ^**^*p* < 0.01 compared to the Model group.
